# Understanding Conformation Importance in Data-Driven Property Prediction Models

**DOI:** 10.1021/acs.jcim.5c00018

**Published:** 2025-03-18

**Authors:** Yu Hamakawa, Tomoyuki Miyao

**Affiliations:** † Graduate School of Science and Technology, Nara Institute of Science and Technology, 8916-5 Takayama-cho, Ikoma, Nara 630-0192, Japan; ‡ Data Science Center, Nara Institute of Science and Technology, 8916-5 Takayama-cho, Ikoma, Nara 630-0192, Japan

## Abstract

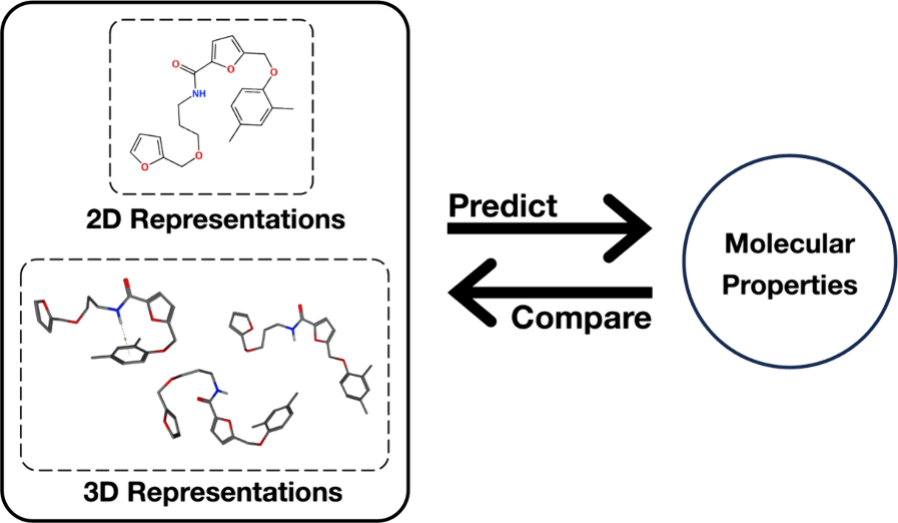

The prediction of molecular properties is essential in chemoinformatics and has many applications in drug discovery and materials design. Molecular representations play a key role in the prediction models to achieve high prediction accuracy. Nevertheless, appropriate molecular descriptors, including the utilization of conformational information, have been unclear due to a lack of systematic analysis of property prediction models and control. This study investigates the influence of using multiple conformers in machine learning-based property prediction, comparing two- and three-dimensional descriptors using three independent data sets: a large-scale quantum mechanical property, a medium-scale melting point, and small-scale enantioselective chemical reaction data sets. One unique aspect of this study is creating these carefully controlled data sets for models’ performance evaluation in conformational diversity and the target property’s dependence on conformation. Our findings show that using all available conformers as simple data augmentation consistently achieves high prediction accuracy among aggregation approaches, followed by mean aggregation. Furthermore, Uni-Mol, an end-to-end prediction model utilizing atomic coordinates and elements, combined with the ground-truth conformation, significantly outperformed traditional 2D and 3D descriptors and predicted conformational-sensitive properties with high accuracy. Although the prediction accuracy of the Uni-Mol model significantly decreased using the wrong conformers, it still outperformed two-dimensional extended connectivity fingerprints, which showed higher prediction accuracy than most of the tested 3D descriptors.

## 1 Introduction

Molecular property prediction based on its structural characteristics is a central issue in chemoinformatics with wide-ranging applications, from drug discovery to materials design. A property prediction model is created with a machine learning (ML) algorithm that takes molecular descriptors as input, which are numerical representations of a molecule.^1^ Although designing appropriate molecular descriptors is essential and experts embody their hypotheses in descriptors, many analyses use universally applicable descriptors for the chemical structure of a molecule, irrespective of target properties. These descriptors include a collection of descriptors from the literature and molecular fingerprints, such as extended connectivity fingerprints (ECFPs).^2^ ECFPs have been successfully used in the virtual screening of compounds^3,4^ and property predictions.^5^

Molecular descriptors can be classified into two-dimensional (2D) and three-dimensional (3D). 2D descriptors are calculated based on the chemical graph of a molecule, while 3D descriptors are based on the conformation of a molecule. It is believed that 3D descriptors should be better than 2D descriptors because molecules exist in the 3D space. However, in retrospective validations of property and activity predictions, the relative merits of 2D or 3D descriptors were not consistent across different targets and data sets.^6−12^ Additionally, many neural network models using Simplified Molecular Input Line Entry System (SMILES) strings or chemical graphs as input, i.e., 2D, have shown high prediction accuracy for physicochemical properties and biological activities, such as solubility, lipophilicity, toxicity, and even for quantum mechanics (QM)-based properties.^13−18^ However, recent studies conducting extensive benchmark calculations revealed that these representation learning methods showed limited performance compared with traditional machine learning models.^19,20^ Further clarification is required to find suitable descriptors, even within a category of 2D representations. Although neural networks utilizing the 3D coordinates of atom elements have recently shown comparative prediction accuracy for these properties,^21,22^ dominant approaches for property prediction still depend on 2D descriptors (representations).

One possible reason for the insufficient prediction accuracy for 3D representations is that the conformer(s) employed in the model is not directly related to the target property or activity. For 3D descriptor calculations, optimized molecular conformation based on molecular force field or QM calculation is frequently used. However, the optimized conformation sometimes differs from the influential conformation due to modeling limitations (e.g., vacuum in QM). Besides, the thermodynamic properties are determined by an ensemble of conformers.

Apart from relying on a single conformer, an ensemble of conformers can be used to calculate sets of descriptors, which are aggregated into a set of descriptors for the compound.^23^ For the aggregation, a weighting scheme based on conformer existence probabilities was proposed.^24^ In the simplest case, sets of descriptors are aggregated by averaging their values (i.e., equal weighting). Furthermore, without aggregation, these descriptor sets can be regarded for a set of instances, and modeling can be conducted by giving the instances the same objective variable value.^25^ This modeling approach is the most straightforward multi-instance learning (MIL) method, where instances correspond to conformers and multiple instances are simultaneously used for training a model. MIL is an emerging approach to handle various conformers without aggregating descriptor values. In particular, neural network-based approaches have been proposed to automatically extract important conformers for target properties.^25,26^ For example, a sum pooling method with a multilayer perceptron and a weighted sum scheme based on a self-attention architecture was proposed. The self-attention-based architecture can learn conformer weights from a training data set.^27^

To understand the difference between 2D and 3D molecular descriptors and different 3D descriptor aggregation methods, including MIL approaches, in property prediction models, a large-scale data set of conformer-dependent properties with a ground-state conformation is necessary. Otherwise, any conclusions would be “dependent on the data set”. In this respect, previous studies for comparing molecular representations are not sufficient.

Herein, we compiled QM property data sets extracted from the PubChemQC PM6 database,^28^ where only conformationally diverse small molecules were included with the ground-truth conformation for six QM properties, for which conformational dependency would be quantified. In this study, tested 2D molecular descriptors were ECFP and topological pharmacophore fingerprints. 3D descriptors were ones calculated by Molecular Operating Environment (MOE),^29^ Pmapper representing pharmacophore triplets (quadruplets),^30^ 3D-molecule representation of structures based on electron diffraction (3D-MoRSE),^31−33^ many-body tensor representation (MBTR).^34^ These 3D representations were combined with several aggregation methods to build property prediction models. As a 2D neural network approach, a pretrained graph neural network model by Molecular Contrastive Learning of Representations via Graph Neural Networks (MolCLR)^17^ was utilized. As 3D-based neural networks, the geometry-enhanced molecular representation learning method (GEM)^21^ and Uni-Mol^22^ were employed. GEM is based on atom-bond and bond-angle graphs extracted from 3D spatial structures. A Uni-Mol model takes atomic 3D coordinates and element types as the input. These models were pretrained using a massive number of molecular structure data. Furthermore, the importance of conformers is discussed based on prediction accuracy differences by adjusting similarity to the ground-truth conformations. Statistical hypothetical testing was conducted to derive fair conclusions.

Findings derived from the large-scale QM data set were discussed through two case studies: one using a medium-scale data set of melting points (MP) of small molecules and the other using small-scale data sets for modeling catalyst structure–reactivity relationships. Both cases assumed a practical situation in which conformer contributions to the target properties were unknown.

## 2 Materials and Methods

### 2.1 Data Sets

Four data sets were prepared to build and evaluate property prediction models using various molecular representations. A PubChemQC PM6 data set (PQC data set) was manually compiled from the PubChemQC PM6 database, focusing on conformational diversity. Similarly, a melting point data set (MP data set) was created from the Jean-Claude Bradley Double Plus Good (Highly Curated and Validated) Melting Points Data set, containing conformationally diverse compounds. APTC-1 and APTC-2 data sets were extracted from the reference^35^ without any modification.

#### 2.1.1 PubChemQC PM6 Data Set (PQC Data Set)

The PubChemQC PM6 database^28^ contains over 221 million molecules extracted from the PubChem database.^36^ Each molecule in the database was structurally optimized using the PM6 method,^37^ and nine quantum chemistry (QC) properties using the PM6 Hamiltonian are stored along with the optimized coordinates. In this study, six properties were selected as targets: the dipole moment (Debye), HOMO energy (HOMO) (eV), LUMO energy (LUMO) (eV), HOMO–LUMO energy gap (HOMO–LUMO Gap) (eV), total energy (SCF Energy) (eV), and enthalpy (Hartree). The conformers (atom coordinates) with which the PM6 property values were calculated are called “ground-truth”. A subset of the PubChemQC PM6 database was extracted to contain only conformationally diverse compounds. This subset of the data set was termed the PQC data set and was further used for machine learning (ML) model evaluation.

The extraction procedure started by downloading 509,453 compounds from the CHONsubset of the PubChemQC PM6 database, where molecules consisted of hydrogen, carbon, nitrogen, and oxygen with a molecular weight of less than 500. Original compound data were recorded in JSON format and converted to SDF format. To select conformationally flexible compounds, the 509,453 compounds were sorted by the number of rotatable bonds in descending order, and the top 100,000 compounds were extracted. After generating and optimizing the conformers for these 100,000 compounds, a final data set of the 97,696 compounds forming 644,648 conformers was prepared as the PQC data set. Since one compound had a missing value for enthalpy, 97,695 compounds forming 644,642 conformers were used to predict values for all properties.

#### 2.1.2 Melting Point Data Set (MP Data Set)

The Jean-Claude Bradley Double Plus Good (Highly Curated and Validated) Melting Points Data set^38^ is a highly reliable data set, consisting of 3041 melting point measurements. Each compound was measured at least two times, and the range of the melting point values was between 0.01 and 5 °C, which was extracted from the 28,645 melting point measurements in the Jean-Claude Bradley Open Melting Point Data set.^39^ The average of the MP (degree Celsius) is used as the target property. The melting point of a compound reflects the strength of the intermolecular interaction and packing (symmetry) in the crystal lattice and conformational flexibility in the liquid phase. The conformation in the crystal lattice may not be a local optimum in a vacuum, and it may not be possible to identify a single conformation essential for predicting the MP. Like the preparation for the PQC data set, compounds with diverse conformers were further extracted from the data set, resulting in 195 compounds. The curation process involves removing the compounds containing chiral carbons and *cis*–*trans* isomers, the compounds comprising elements other than hydrogen, carbon, nitrogen, oxygen, and sulfur, and the compounds with less than six rotatable bonds. The remaining 195 compounds contained 9703 conformers in total.

#### 2.1.3 Asymmetric Phase Transfer Catalysts Data Sets (APTC-1 and APTC-2 Data Sets)^40,41^

The APTC-1 and APTC-2 data sets consist of the enantioselective alkylation reactions for the α-carbon of a glycine derivative, each using an APTC (ammonium ion catalyst). Since the catalysts were the source of selectivity, catalyst conformation was essential to predict selectivity.^35,42−44^ Each data record consists of the SMILES^45^ string of a catalyst and ΔΔ*G*^‡^ (kcal/mol) of the reaction. The difference between APTC-1 and APTC-2 is the scaffolds of the catalysts: cinchona alkaloid-based for APTC-1 and dibenzo[*b*,*f*]azepine for APTC-2. The sizes of the data sets are quite small: 88 catalysts (reactions) for APTC-1 and 40 catalysts for APTC-2. Like the MP data set, the true conformer(s) were unknown. After generating conformers for each compound, the total number of training samples became 4371 conformers for 88 catalysts for APTC-1 and 1864 conformers for 40 catalysts for APTC-2. A discussion on whether ML models can accurately predict experimental ΔΔ*G*^‡^ values using multiple conformers is provided in this study.

### 2.2 Conformer Generation

In the PQC data set, the OMEGA toolkit (version 2023.2.3) from OpenEye^46,47^ was used for conformer generation with the following settings: the maximum number of conformers was 40, and the root-mean-square deviation of atomic positions (RMSD) threshold was 2.0 Å to sample diverse conformers.^48^ The compounds having uncertain stereocenters were removed. It resulted in 97,695 compounds and a total of 644,642 conformers.

In the MP, APTC-1, and APTC-2 data sets, conformers were generated using the same protocol used in the previous study to construct ML models for the APTC-1 and APTC-2 data sets.^35^ The RDKit (version 2023.9.1)^49^ conformer generator ETKDG^50^ was used, with parameters of the maximum number of conformers being 50, an energy window of 50, and force field UFF.^51^ When the RDKit conformer generator failed to generate conformers, a systematic conformer generator from the Open Babel package^52^ was used, and the total energy of conformers was recalculated using the MMFF94 force field^53^ implemented in RDKit. It resulted in 9703 conformers for 195 compounds in the MP data set, 4371 conformers for the 88 catalysts in the APTC-1 data set, and 1864 conformers for the 40 catalysts in the APTC-2 data set. Molecular properties in the MP, APTC-1, and APTC-2 data sets are likely to be determined by an ensemble of (un)stable conformers. Thus, the force field-based optimization using the RDKit library seemed sufficient. Conformational diversity for each data set is discussed in the next section.

### 2.3 Structural Optimization and Conformational Diversity

**Figure 1:**
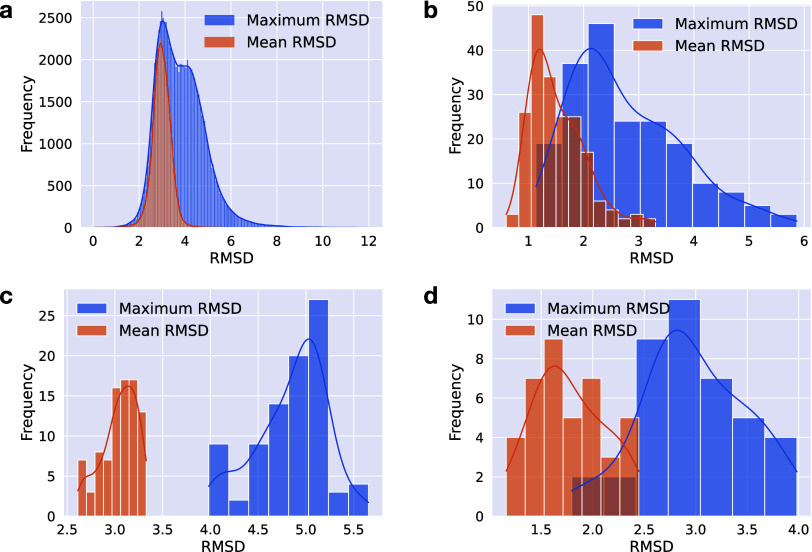
Compounds’ conformational diversity in the PQC, MP, APTC-1, and APTC-2 data sets. Two histograms of the maximum and average compound-wise RMSD (Å) values among conformers are provided in blue and orange, respectively, for the PQC data set (a), the MP data set (b), the APTC-1 data set (c), and the APTC-2 data set (d). An exhaustive pairwise comparison was conducted to derive the statistics. Hydrogen atoms were included in the RMSD calculation.

Each generated conformer by the OMEGA toolkit for the PQC data set was further optimized using a semiempirical molecular orbital method with the PM6 Hamiltonian in MOPAC (version 22.1.1).^54^ Structural optimization using the semiempirical method was not performed for the MP, APTC-1, and APTC-2 data sets, because the target properties for these data sets may depend on an ensemble of multiple (un)stable conformers rather than a single stable conformer. Figure 1 shows the histograms of RMSDs for the mean and maximum pairwise RMSD values among the conformers. RMSDs were calculated for all atoms, including hydrogens after rotationally and translationally moved to give the minimum RMSD using the OEChem toolkit.^55^ In the PQC data set, 5481 compounds had single conformers, and the RMSD calculation was performed for 92,214 compounds that had two or more conformers. The average of the maximum RMSD value among conformers in the PQC data set was 3.82 Å (mean was 2.98 Å), 2.77 Å (mean was 1.52 Å) in the MP data set, 4.85 Å (mean was 3.05 Å) in the APTC-1 data set, and 2.97 Å (mean was 1.77 Å) in the APTC-2 data set. Thus, all data sets consisted of compounds with diverse conformers. For the PQC data set, the distributions of the number of rotatable bonds, the distribution of the average RMSDs from the ground-truth conformers, and the number of generated conformers per compound are reported in Figure S1 of the Supporting Information. For the MP, APTC-1, and APTC-2 data sets, the same information is reported in Figure S2.

**Figure 2:**
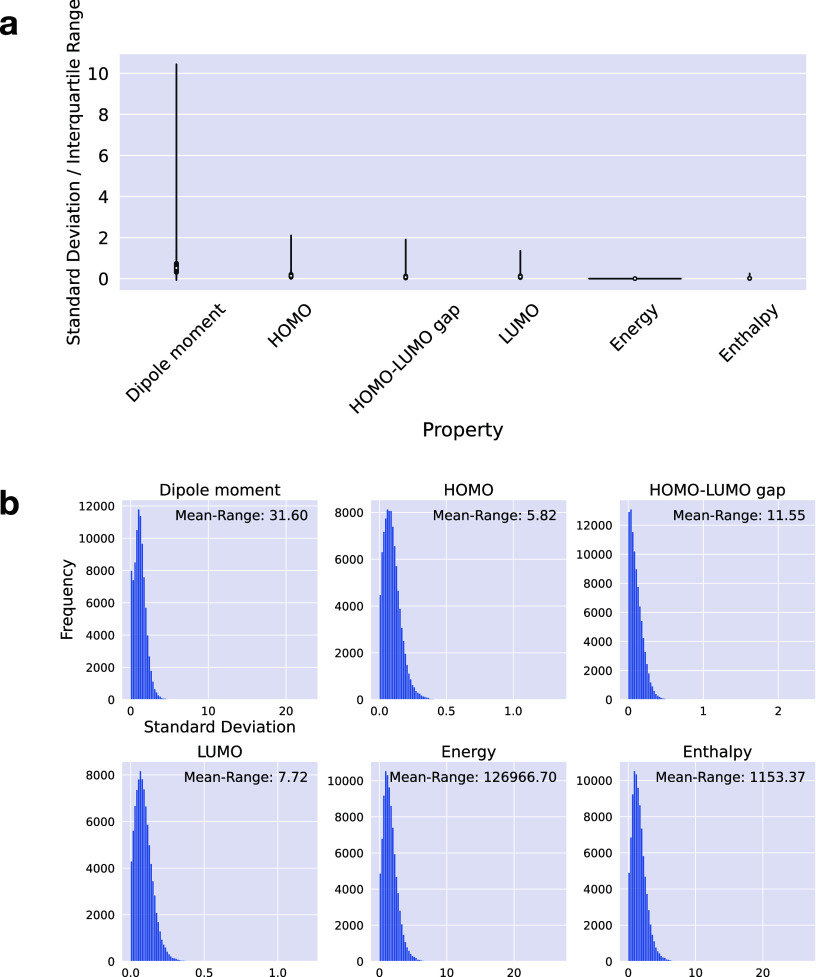
Visualization of the variation of the six properties depending on conformation differences. (a) Property-wise violin plots of the standard deviations are scaled by the interquartile ranges of the property values. For each compound, a scaled standard deviation is calculated for multiple conformers. (b) Histograms of the standard deviations before scaling. A mean-range value in each histogram plot is the range of the average property values. The unit of dipole moment is Debye, HOMO, LUMO, and HOMO–LUMO gap are eV, and energy and enthalpy are kcal/mol.

Diversity in QC properties depending on the conformation is shown in Figure 2. The same properties as for the ground-truth conformation were calculated with MOPAC. Note that the units of properties recorded in the PQC data set and those extracted from MOPAC were different. In MOPAC, the unit of the dipole moment was Debye, HOMO, LUMO, and HOMO–LUMO Gap were in eV, and the energy and the enthalpy were in kcal/mol. Figure 2a shows the distributions of the scaled standard deviations for the six selected properties depending on the conformers. A standard deviation value corresponded to one compound, whose conformers were used to derive property values, and the statistic was calculated. These standard deviation values were divided by the interquartile range to understand the conformational diversity of the QC property values. Distributions before the scaling operation are shown in Figure 2b. The dipole moment was highly conformation-dependent with significant variations due to conformational coordinates. On the other hand, the energy and enthalpy showed low conformational dependency.

### 2.4 Molecular Descriptors

Four types of 3D descriptors (MOE, Pmapper, 3D-MoRSE, and MBTR descriptors) and two types of 2D descriptors (ECFP and 2D pharmacophore fingerprints (2D PFP)) were used.

#### 2.4.1 MOE Descriptors

MOE Descriptors were the 3D descriptors calculated by the MOE software (version 2022.02)^29^ and consisted of 117 descriptors. The descriptors implemented in MOE primarily pertain to drug discovery, focusing on molecular interaction, such as the polar surface area and molecular shape. All 117 descriptors are internal coordinate dependent. The descriptors based on the semiempirical molecular orbital method, such as AM1, were excluded. For the MP, APTC-1, and APTC-2 data sets, prediction models were constructed with all 117 descriptors, while for the PQC data sets, models were built with the 116 descriptors excluding ‘dipole’. Brief descriptions of the used descriptors are listed in Table S1.

#### 2.4.2 Pmapper Descriptors

Pmapper descriptors count the number of 3D pharmacophore quadruplets inside a molecule.^30^ A set of descriptors is an integer vector consisting of quadruplets and their frequencies. Following the previous research protocol for building enantioselectivity prediction models,^35^ triplets instead of quadruplets were used, and any atoms except for five- and six-membered ones and the centers of five- or six-membered rings were specified as vertices of triplets. When *cis*–*trans* isomers are involved, triplets cannot distinguish the isomers. As in the original research, all distances between atoms in triplets were binned at an interval of 1 Å. The atom triplets are standardized so that unique combinations are stored.

For the PQC data set, 1028 unique atom triplets were generated (a 1028-dimensional vector) for conformers produced by OMEGA, and 864 for ground-truth conformations. Similarly, 575 atom triplets were generated for the MP data set, 1202 for the APTC-1 data set, and 1556 for the APTC-2 data set. A brief description of the specification of vertices in Pmapper descriptors for each data set is shown in Table S2.

#### 2.4.3 3D-MoRSE Descriptors

3D-MoRSE descriptors convey information about atomic coordinates based on the transformation function utilized in the electron diffraction study.^31−33^ The general formula of the descriptor is provided in eq 1.
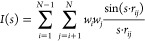
1where *N* represents the number of atoms, *w_i_* and *w_j_* denote the weights for atoms *i* and *j*, *r_ij_* is the distance between these atoms, and *s* is a parameter, typically taking one value from the set {1,···,32 Å}. Different weight types are employed to capture various characteristics of atomic coordinates. Recently, 3D-MoRSE descriptors were employed for accurately predicting the electronic coupling of organic semiconductors in both crystalline and amorphous phases.^56^ In this study, 160 3D-MoRSE descriptors were calculated by the Mordred package.^57^ The types of weights in 3D-MoRSE are listed in Table S3.

#### 2.4.4 Many-Body Tensor Representation (MBTR)

MBTR encodes a 3D structure through a set of element-specified geometrical distributions specified by degrees.^34^ A degree in the MBTR represents the number of elements involved, and the geometry function is provided as a parameter for each degree. For example, at degree 2, two elements are focused, and the distribution can be created by applying the geometry function (e.g., the inverse of distance). A set of distributions forms the MBTR descriptors. In this study, the DScribe package^58^ was utilized for calculating the MBTR descriptors. The parameters of the MBTR descriptors were the same as those used in the previous study that developed a hierarchical learning model to predict the enantioselectivity of the asymmetric hydrogenations of olefins.^59^ These parameters are listed in Table S4. For the PQC data set, 540-dimension descriptors are generated, 950 dimensions for the MP data set, and 2310 dimensions for the APTCs data set.

#### 2.4.5 Extended Connectivity Fingerprints and 2D Pharmacophore Fingerprints

As 2D descriptors, ECFP and 2D PFP were used. ECFP is an atom-environment-based fingerprint, capturing characteristics of substructures on the structural formula. The bond diameter was set to 4 (ECFP4), and the vector length was 2048 for the PQC and MP data sets and 1024 for the APTCs data sets. For the PQC data set and the MP data set, ECFP was calculated using an in-house program with the help of the OEChem toolkit^55^ from OpenEye, while for the APTCs data sets, the RDKit Morgan fingerprint modules were used. For ECFP, count vector formats were tested for the PQC and MP data sets, while bit and count vector formats were tested for the APTCs data sets. The count and bit format focus on the presence or absence and frequency of a substructure, respectively.

2D PFP is a fingerprint that represents topological pharmacophores using triplets of pharmacophore features with topological distances. Pmapper is a geometrical extension of 2D PFP, the same atom invariants as in the Pmapper descriptor were used: aromatic atoms of five- or six-membered rings and other atoms.^35^ 2D PFP was only calculated for the APTCs data sets, aiming to interpret the merit of Pmapper descriptors. The same pharmacophoric features using the RDKit Pharm2D modules with custom pharmacophore features. 2048-Dimension vectors were generated for the APTCs data sets. The definitions of the used custom pharmacophore features and bins are listed in Table S5.

#### 2.4.6 Aggregation Methods of Conformer Descriptors

3D descriptors are conformation-dependent, taking different values for different conformers. An aggregation method should be applied to derive a numerical vector for one compound. Several approaches were proposed to aggregate a set of descriptor values for multiple conformers.^24,60,61^ The tested aggregation methods in this study are listed below.

1. Boltzmann weight: this method assigns weights to conformer descriptors based on their probabilities of existence, which are calculated based on the Boltzmann distribution. The sum of the existence probabilities of all conformers is normalized to 1. The absolute temperature of the system in the Boltzmann distribution was set to 298 K. The conformer energies were PM6 energies derived with MOPAC for the PQC data set and the MMFF94 force field energies^53^ with RDKit for the MP and APTCs data sets.
  Formally, the existing probability of the *i*-th conformer *p_i_* is denoted as
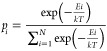
2where *N* is the number of conformers for a certain compound, *E_i_* is the total energy of the *i*-th conformer, *k* is Boltzmann constant, and *T* is the absolute temperature of the system. Then, the aggregated descriptor vector for a compound **x**_Agg_ is denoted as
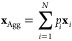
3where **x**_*i*_ is the calculated descriptor vector of the *i*-th conformer.
2. Mean: this method calculates the mean of the descriptor values for all conformers (no weight is assumed). Formally, the aggregated descriptor for a certain compound **x**_Agg_ is denoted as
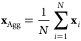
4where *N* is the number of conformers for a certain compound and **x**_*i*_ is the calculated descriptor of the *i*-th conformer.
3. Global minimum: this method extracts the descriptor vector of the conformer with the lowest energy, indicating the descriptors for the most stable conformer. Formally, the aggregated descriptor for a certain compound **x**_Agg_ is denoted as
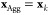
5
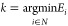
6where **x**_*k*_ is the calculated descriptor of the *k*-th conformer and *E_i_* is the total energy of the *i*-th conformer.
4. Random: this method selects the descriptor values for one randomly sampled conformer.
5. RMSD Max (Min): this method selects the descriptor vector for the conformer showing the maximum (minimum) RMSD to the ground-truth conformation (only tested for the PQC data set). Formally, the RMSD of the *i*-th conformer RMSD_*i*_ is denoted as
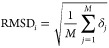
7where *M* is the number of atoms constituting the *i*-th conformer, and δ_*j*_ is the Euclidean distance between heavy atom *j* and reference atom of the ground-truth conformation. Then, the aggregated descriptor **x**_Agg_ for a certain compound is denoted as
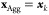
8
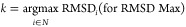
9
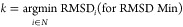
10where **x**_*k*_ is the calculated descriptor of the *k*-th conformer and *N* is the number of conformers for a certain compound.
6. Nonaggregation: this method simply uses the descriptor vectors for all conformers. During training, the objective variable values for the conformers are that of the compound: this means that multiple conformers of a compound take a single property value. When predicting the property value for a test compound, the predicted values for the conformers are averaged, and the averaged value becomes the model’s output.

Aggregation methods of Random and RMSD Max (Min) were used as a control.

### 2.5 Machine-Learning (ML) and Neural Network Models

To evaluate molecular representation and aggregation methods fairly, several ML methods and previously proposed property prediction modeling architectures were tested. As linear regression methods, partial least-squares regression (PLS)^62^ and elastic net^63^ were adopted, and as nonlinear regression methods, random forest (RF)^64,65^ and support vector machine (SVM).^66^ PLS, elastic net, and SVM were used for only the APTCs data sets. In addition to traditional modeling approaches, recently proposed multi-instance learning (MIL) approaches for handling multiple conformers were used.^35^ MIL assigns a shared label to multiple instances, treating them as a single bag for learning. The models proposed in the previous study combined a MIL model with a conformer weight subnetwork to identify important conformers for prediction. In previous research,^35^ the term Instance-Wrapper was used for the method identical to nonaggregation. The only difference is the base ML model: ours is RF, and the previous research one^35^ is multilayer perceptron (MLP). Thus, in this study, the term nonaggregation is used to represent Instance-Wrapper for consistency.

As molecular representation learning methods, one 2D-based and two 3D-based neural networks were employed. A graph neural network (GNN)^67,68^ pretrained using MolCLR was the 2D-based model, termed MolCLR.^17^ GEM,^21^ taking 3D molecular graphs as input, Uni-Mol^22^ as SE(3) Transformer architecture,^18^ taking 3D atomic coordinates and element types as input, were employed as 3D-based neural networks.

MolCLR is a GNN-based contrastive learning method,^69,70^ in which a node represents an atom and an edge a covalent bond between atoms. The model was pretrained to distinguish sets of pseudomolecular graphs augmented from molecular graphs. GEM is a 3D-based GNN method that converts a 3D spatial structure to two molecular graphs: atom-bond and bond-angle graphs. The atom-bond graph is identical to the molecular graph in 2D-based GNN. The bond-angle graph represents geometrical information where a node represents a covalent bond and an edge is the angle between connected nodes (connected covalent bonds). Furthermore, Uni-Mol^22^ was tested due to its high prediction ability for the various QSAR and QSPR benchmarking data sets. The architecture of Uni-Mol is based on the Transformer network, where the attention mechanism reflects discrete atom interactions. An atomic environment is represented in the pair matrix, which uses Euclidean distances of atom pairs, followed by a pair-type aware Gaussian kernel.^71^ The pair matrix also participates in the attention mechanism and serves as the positional encoding for atoms.

The pretrained models provided by the authors for MolCLR, GEM, and Uni-Mol were employed in this study.^72−74^ These models were fine-tuned for the four data sets: PQC, MP, APTC-1, and APTC-2. The MolCLR model had been pretrained on about 10 million unlabeled molecules sampled from PubChem,^36^ the GEM model on about 20 million unlabeled molecules samples from ZINC15,^75^ and the Uni-Mol on about 19 million unlabeled molecules with 209 million conformation samples from the ZINC15 and ChEMBL databases.^76^ Although the source of the PQC data set was PubChem, all the pretraining procedures used unlabeled (i.e., self-supervised) data. Thus, no potential bias to improve the prediction accuracy was included before the fine-tuning stage.

Table S6 lists the hyperparameters for the models. For MIL, MolCLR, GEM, Uni-Mol, Elastic Net, PLS, and SVM, default hyperparameter values were used. For RF, Optuna^77^ tuned the hyperparameters for the PQC data set analysis and fixed them as defaults for the MP, APTC-1, and APTC-2 data set analyses.

### 2.6 Training and Test Data Splitting

For the PQC data set, five-times three-fold cross-validations (CV) with different random seeds (15 test sets in total) were conducted to evaluate the prediction accuracy of the modeling approaches. Similarly, for the MP data set, five-time five-fold cross-validations (CV) with different random seeds (25 test sets in total) were conducted. In each fold, compounds were randomly split into training (80%) and test (20%) sets, and models were optimized and trained only using the training data set; test compounds were the target for the property prediction. The training data set for RF was further divided into training and validation sets (80:20) for hyperparameter tuning for the PQC data set. Uni-Mol used a 5-fold CV for model selection and early stopping.

In the previous study, the APTCs data sets were used to evaluate multi-instance NN models. Only one pair of training and test data sets was used for each APTC.^35^ Although the train-test splitting ensured that substrates in the test data set were not included in the training data set, the APTC-1 and APTC-2 data sets were small, with 88 and 40 catalysts, respectively. This small size might impair fair evaluations of prediction accuracy, e.g., unexpected overfitting of the test set.

In this study, different validation strategies were used. For the APTC-1 data set, five times 5-fold cross-validations (CV) with different random seeds (25 test sets in total) were conducted. For the APTC-2 data set, leave-one-out cross-validation (LOOCV) was used.

### 2.7 Evaluation Metrics

As evaluation metrics for the prediction accuracy of ML models, the coefficient of determination (*R*^2^), the mean absolute error (MAE), and the root mean squared error (RMSE) were used. These metrics are defined by the following equations:
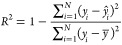
11
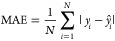
12
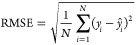
13where *N* is the number of data, *ŷ*_*i*_ is the predicted value for the *i*-th data, *y̅* is the average of the observed values of *y_i_* {*i* = 1,···,*n*}.

Except for the APTC-2 data set, these evaluation metrics were calculated for each training or test data set, and the average and standard deviation were derived from all of the training or test data sets. For the APTC-2 data set, where LOOCV was performed, these evaluation metrics values were calculated once by treating a total of 40 LOOCV-based prediction values as a single data set.

### 2.8 Statistical Testing for Performance Evaluation

This study compares multiple descriptors, 3D descriptor aggregation methods, and ML models for the four types of data sets including six QC properties, MP, and enantioselectivity. Statistical hypothesis testing was conducted on the prediction accuracy based on a previously reported study on molecular property prediction.^19^ The distribution of the prediction accuracy values for test data sets was checked by plotting them. The PQC, MP, APTC-1, and APTC-2 data sets contained 15, 25, 25 test sets, and 40 test points, respectively; each distribution consisted of 15, 25, 25, and 40 data points. The distribution of each prediction accuracy did not follow the normality and equal-variance assumptions. Thus, nonparametric tests were employed.

While previous studies used the Mann–Whitney *U* test^78^—a common method for unpaired nonparametric tests—we employed the Wilcoxon signed-rank test^79^—a standard method for paired nonparametric tests—since two accuracy values for the same test and training data sets could be compared. Then, we tested the difference in prediction accuracy between the pair of modeling approaches. The modeling approaches were organized in several tables, and the tested results were reported table-wise. The significance level was set to 0.05 (a two-sided *p*-value). Furthermore, no counteract correction to the multiple comparison problems was conducted, such as the Bonferroni correction.^80^ Models for comparison were selected with specific intentions such as aggregation methods and descriptors. The number of selected modeling approaches might disrupt the balance of the correction, making it easier or harder for significant differences to emerge.

An analysis of variance (ANOVA),^81^ in addition to hypothesis testing, was conducted in several cases where the data set size was small, and the variation of the evaluation metric values for the test data sets was large.

## 3 Results and Discussion

### 3.1 Study Design

The objectives of this study are to clarify (1) the circumstances where 3D molecular representation is better than 2D, along with understanding the importance of “ground-truth” conformation, (2) the best aggregation method for producing a single descriptor vector from 3D molecular descriptor sets corresponding to multiple conformers; (3) whether the MIL approaches are effective or not. Three types of data sets (the total is four data sets) were used for these purposes: the PQC, MP, and APTCs data sets.

**Figure 3:**
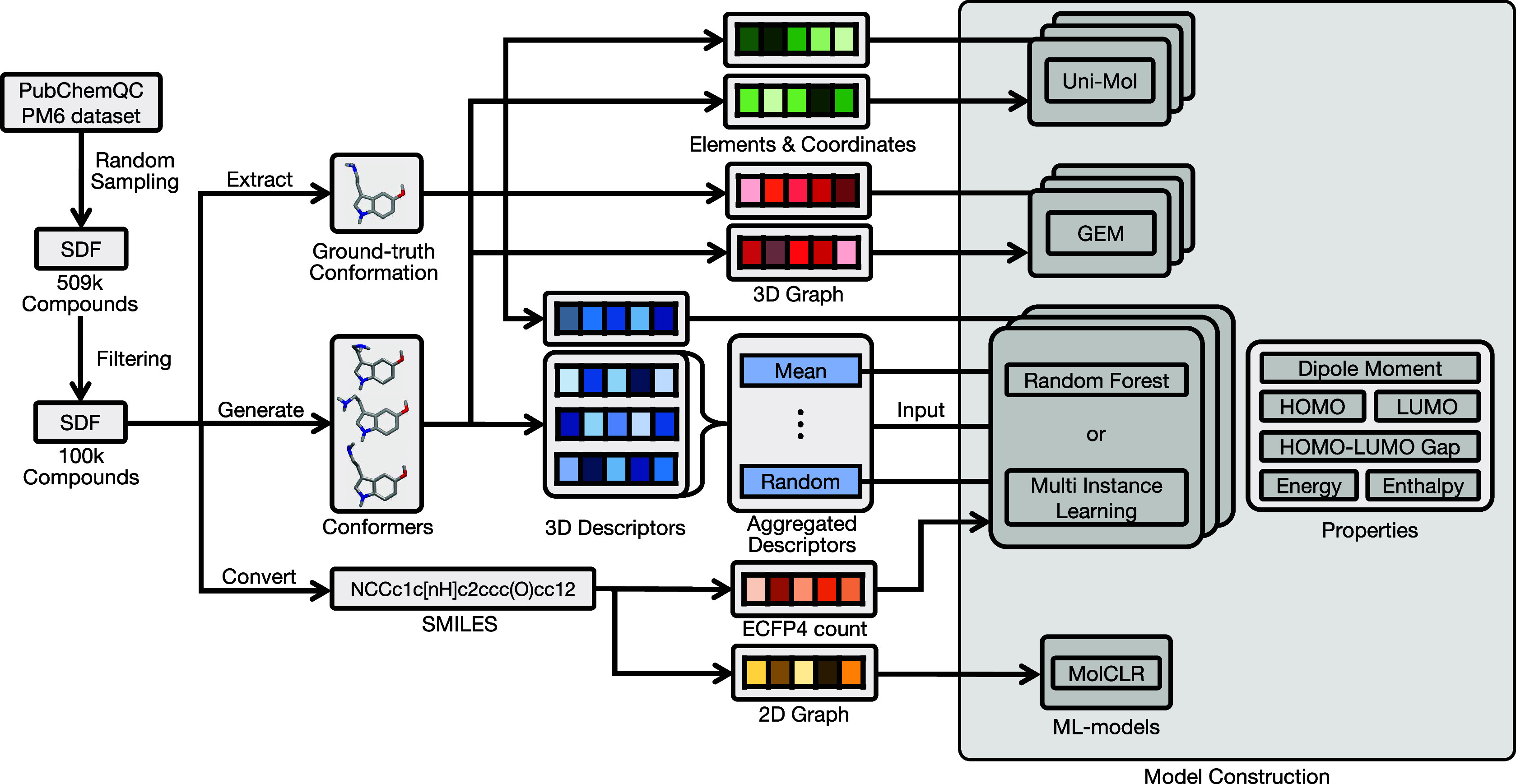
Modeling workflow using the PQC data set. The PQC data set contains the coordinates of compounds optimized by the PM6 Hamiltonian and properties: Dipole moment (Debye), HOMO (eV), LUMO (eV), HOMO–LUMO Gap (eV), Energy (eV), and Enthalpy (Hartree). After randomly sampling molecules from a subdata set containing only hydrogen, carbon, oxygen, and nitrogen atoms and with a molecular weight of 500 or less, the top 100,000 molecules in terms of the highest number of rotatable bonds were extracted. 3D descriptors were MOE, Pmapper 3D-MoRSE, and MBTR, and the 2D descriptor was ECFP4 count format. These 3D descriptors were calculated for generated multiple conformers and aggregated separately using the six aggregation methods as well as for the ground-truth conformation.

Using the PQC data set where six QC properties are annotated for each compound, we directly tested the three hypotheses above by comparing the prediction accuracy. The PQC data set consisted of random compounds with diverse conformers (the average of maximum RMSD between conformers was 3.82 Å, Figure 1), with the “ground-truth” conformation with which the property values were calculated. The whole experimental procedure using the PQC data set is presented in Figure 3. This data set enabled a fair evaluation of the importance of the conformation. Using around 100,000 diverse compounds with the six QM properties, various molecular representations were calculated and used as inputs of ML models. The input molecular representations were 3D descriptors using the ground-truth conformation, 3D descriptors with (non) aggregation methods, ECFP for RF and MIL, 2D and 3D molecular graphs for MolCLR and GEM respectively, and atomic elements and coordinates for Uni-Mol.

The MP data set and APTCs data sets (APTC-1 and APTC-2) were used to discuss findings from the PQC data set. The MP data set is a medium-scale experimental data set with 195 compounds, and the relation between the objective variable (melting point) and local minimum conformers was unknown. Finally, The APTCs data sets are small, with 88 and 40 reactions for APTC-1 and APTC-2, respectively, and the relation between the objective variable (ΔΔ*G*^‡^) and the stable catalyst conformer is also unknown, let alone experimentally identifying influential conformers to the reaction. Various modeling approaches used in analyzing the PQC data set were tested for these real application-oriented data sets.

### 3.2 Analysis with the PQC Data Set

#### 3.2.1 Aggregation Methods for 3D Descriptors

The prediction performance of the RF models for the six QC properties using various aggregation methods for the 3D descriptors (MOE, Pmapper, 3D-MoRSE, and MBTR) is summarized in Table 1. In this table, performance with the nonaggregation method, i.e., using the descriptors for all conformers of a compound as a set of augmented instances, the ground-truth conformation, and ECFP4 count are also reported.

**Table 1:** Averaged Prediction Accuracy (*R*^2^) of Random Forest (RF) Models for 15 Test Sets of the PQC Data Set Six Property Predictions^a^

			dipole moment	HOMO	HOMO–LUMO gap	LUMO	energy	enthalpy
MOE	aggregation method	Boltzmann weight	0.431 (0.008)	0.712 (0.003)	0.856 (0.002)	0.894 (0.001)	0.879 (0.003)	0.912 (0.007)
		mean	0.452 (0.008)	0.745 (0.002)	0.869 (0.002)	0.904 (0.002)	0.894 (0.003)	0.923 (0.002)
		global minimum	0.422 (0.008)	0.700 (0.003)	0.848 (0.005)	0.889 (0.002)	0.872 (0.003)	0.908 (0.002)
		RMSD max.	0.418 (0.006)	0.690 (0.003)	0.843 (0.002)	0.884 (0.002)	0.867 (0.003)	0.904 (0.002)
	nonaggregation		0.455 (0.006)	0.761 (0.003)	0.880 (0.002)	0.909 (0.002)	0.907 (0.007)	0.933 (0.006)
	ground-truth		0.498 (0.008)	0.672 (0.004)	0.865 (0.002)	0.877 (0.004)	0.849 (0.015)	0.901 (0.002)
Pmapper	aggregation method	Boltzmann weight	0.311 (0.006)	0.529 (0.006)	0.803 (0.004)	0.739 (0.004)	0.678 (0.005)	0.599 (0.004)
		mean	0.329 (0.006)	0.561 (0.005)	0.817 (0.004)	0.760 (0.005)	0.706 (0.005)	0.643 (0.005)
		global minimum	0.302 (0.009)	0.513 (0.007)	0.794 (0.004)	0.724 (0.004)	0.664 (0.005)	0.572 (0.005)
		RMSD max.	0.295 (0.007)	0.487 (0.007)	0.785 (0.004)	0.713 (0.005)	0.654 (0.006)	0.564 (0.004)
	nonaggregation		0.352 (0.007)	0.676 (0.114)	0.829 (0.010)	0.775 (0.003)	0.719 (0.005)	0.661 (0.004)
	ground-truth		0.358 (0.007)	0.535 (0.006)	0.813 (0.005)	0.741 (0.005)	0.669 (0.005)	0.581 (0.004)
3D-MoRSE	aggregation method	Boltzmann weight	0.330 (0.005)	0.649 (0.003)	0.837 (0.002)	0.845 (0.003)	0.739 (0.005)	0.770 (0.004)
		mean	0.359 (0.006)	0.696 (0.003)	0.858 (0.002)	0.868 (0.003)	0.793 (0.003)	0.816 (0.003)
		global minimum	0.314 (0.006)	0.618 (0.003)	0.824 (0.003)	0.830 (0.003)	0.709 (0.008)	0.745 (0.004)
		RMSD max.	0.309 (0.005)	0.615 (0.004)	0.817 (0.003)	0.823 (0.003)	0.706 (0.004)	0.739 (0.003)
	nonaggregation		0.338 (0.004)	0.679 (0.003)	0.849 (0.002)	0.865 (0.006)	0.789 (0.004)	0.809 (0.006)
	ground-truth		0.337 (0.007)	0.632 (0.003)	0.824 (0.003)	0.837 (0.004)	0.708 (0.005)	0.740 (0.003)
MBTR	aggregation method	Boltzmann weight	0.476 (0.006)	0.801 (0.002)	0.913 (0.002)	0.940 (0.001)	0.960 (0.001)	0.968 (0.001)
		mean	0.487 (0.007)	0.814 (0.002)	0.919 (0.002)	0.943 (0.001)	0.963 (0.001)	0.970 (0.001)
		global minimum	0.472 (0.007)	0.795 (0.002)	0.910 (0.002)	0.937 (0.001)	0.957 (0.001)	0.966 (0.004)
		RMSD max.	0.471 (0.007)	0.790 (0.002)	0.909 (0.001)	0.936 (0.001)	0.955 (0.001)	0.966 (0.001)
	nonaggregation		0.489 (0.006)	0.822 (0.007)	0.925 (0.002)	0.946 (0.002)	**0.968 (0.001)**	**0.976 (0.001)**
	ground-truth		**0.543 (0.007)**	0.809 (0.008)	0.918 (0.002)	0.940 (0.001)	0.954 (0.002)	0.966 (0.001)
ECFP4 count			0.516 (0.006)	**0.867 (0.005)**	**0.939 (0.001)**	**0.956 (0.001)**	0.960 (0.002)	0.941 (0.002)

a For each average prediction accuracy, the standard deviation is provided in the parentheses. All methods used MOE 116 descriptors, Pmapper 1028 descriptors for generated conformers and 864 for ground-truth conformations, 3D-MoRSE 160 descriptors, MBTR 540 descriptors, or ECFP4 count to build models. The best performance value per target is highlighted in bold, and the second-best is underlined.

Overall, the ECFP4 count (2D representation) and MBTR nonaggregation showed consistently high prediction accuracy across all properties. MBTR ground-truth outperformed ECFP4 for the dipole moment. MBTR, based on the atomic coordinate information without specific abstraction like the MOE descriptors, was better than the other 3D descriptors for the QC property prediction.

The structural-formula representation of ECFP4 sufficiently predicted the QC properties of compounds with high conformational diversity, particularly HOMO, HOMO–LUMO gap, and LUMO. *R*^2^ values for the dipole moment and HOMO were slightly lower than for other properties by the ECFP count. The *R*^2^ value for the dipole moment was 0.516, and that for HOMO was 0.867. This may be due to the conformational dependence of the dipole moment and the HOMO energy, as shown in Figure 2a.

However, even for these conformation-dependent target properties, the ECFP count exhibited relatively high accuracy: for the dipole moment, the descriptor was ranked as second, and for HOMO it was ranked as the top in Table 1. The 3D descriptors: MOE, 3D-MoRSE, and MBTR did not fully capture target properties. Among the 3D descriptors, MBTR was the best, followed by MOE, 3D-MoRSE, and Pmapper, irrespective of aggregation methods. It should be noted that most of the prediction models fitted training data sets sufficiently using RF as a regression method, supported by high *R*^2^ values for the training data sets. Even models using Pmapper for the dipole moment reached an *R*^2^ value of 0.9. It may be difficult to determine an appropriate representation in advance without introducing further validation, e.g., CV. Ground-truth conformers seemed meaningful only for the dipole moment prediction and using the MBTR descriptor (average *R*^2^: 0.543), which outperformed the nonaggregated one (0.489) and the ECFP count descriptors (0.516).

Among the aggregation methods tested, the mean aggregation was better than other methods, including using the Boltzmann weight or global minimum. Furthermore, this method gave higher prediction accuracy than using the ground-truth conformation to generate MBTR descriptor values for all properties, except for the dipole moment. However, the nonaggregation method consistently performed better than the mean aggregation, likely due to incorporating augmented descriptor values for multiple conformers into the training data set. This suggests that the simple mean aggregation method is preferable when using under-represented 3D descriptors.

The prediction accuracy *R*^2^ values for all training data sets of the PQC data set are provided from Tables S7 to S32, and the prediction accuracy *R*^2^, MAE, and RMSE for all test data sets are from Tables S51 to S127. The hypothesis testing results for the Table 1 results are provided from Figures S8 to S13.

#### 3.2.2 Multi-Instance Learning (MIL) Models

MIL models were constructed using 3D descriptors for multiple conformers as input. As a representative, a performance overview of the MIL models in combination with the MOE descriptors is shown in Table 2. For the six QC properties, the simple nonaggregation method consistently outperformed other MIL methods. In particular, the advantage of the Bag-Net using the attention mechanism algorithm, which weights important conformers based on the attention scores, was not observed against the nonaggregation models. This relatively simple data-augmentation technique worked consistently better than other sophisticated MIL methods, in addition to consistently outperforming their counterpart method of using single descriptor values as input. Comparing the prediction accuracy between MLP and RF, RF showed higher prediction accuracy for the conformationally sensitive properties: the dipole moment and HOMO.

**Table 2:** Comparison of Averaged Prediction Accuracy (*R*^2^) for 15 Test Sets of the PQC Data Set Six Property Predictions Using Muti-Instance Learning (MIL) Models and MOE 116 Descriptors^a^

		dipole moment	HOMO	HOMO–LUMO gap	LUMO	energy	enthalpy
MIL	nonaggregation	0.388 (0.072)	0.737 (0.012)	0.872 (0.029)	**0.916 (0.010)**	**0.956 (0.004)**	**0.936 (0.024)**
	Bag-Wrapper	0.300 (0.090)	0.663 (0.055)	0.851 (0.009)	0.880 (0.115)	0.914 (0.110)	0.931 (0.040)
	Instance-Net	–0.053 (1.361)	0.686 (0.045)	0.851 (0.014)	0.878 (0.127)	0.936 (0.033)	0.918 (0.060)
	Bag-Net	0.157 (0.475)	0.683 (0.049)	0.851 (0.013)	0.870 (0.150)	0.908 (0.091)	0.929 (0.039)
	Bag-AttentionNet	0.347 (0.064)	0.686 (0.035)	0.855 (0.014)	0.898 (0.032)	0.933 (0.026)	0.924 (0.024)
single conformation	MLP (mean)	–0.055 (1.174)	0.688 (0.022)	0.813 (0.162)	0.835 (0.246)	0.748 (0.502)	0.928 (0.059)
	MLP (ground-truth)	0.319 (0.156)	0.564 (0.130)	0.831 (0.028)	0.291 (1.481)	0.312 (1.556)	0.772 (0.340)
RF	nonaggregation	0.455 (0.006)	**0.761 (0.003)**	**0.880 (0.002)**	0.909 (0.002)	0.907 (0.007)	0.933 (0.006)
	mean	0.452 (0.008)	0.745 (0.002)	0.869 (0.002)	0.904 (0.002)	0.894 (0.003)	0.923 (0.002)
	ground-truth	**0.498 (0.008)**	0.672 (0.004)	0.865 (0.002)	0.877 (0.004)	0.849 (0.015)	0.901 (0.002)

a The RF models using nonaggregation and mean aggregation were also reported as comparison. The standard deviations are provided in parentheses. The best accuracy value per target is highlighted in bold, and the second-best is underlined.

On the other hand, the neural network architecture was better than RF for the relatively conformationally insensitive properties of the energy and the enthalpy. The findings presented above were also consistent when using Pmapper, 3D-MoRSE, or MBTR as a set of molecular descriptors. However, the prediction accuracy using Pmapper, 3D-MoRSE, or MBTR was consistently lower than that using the MOE descriptors. The results of hypothesis testing corresponding to Table 2 are reported in Figures S14–S19.

#### 3.2.3 MolCLR, GEM, and Uni-Mol Models

Three state-of-the-art neural network architectures (MolCLR, GEM, and Uni-Mol) were tested. The inputs of MolCLR, GEM, and Uni-Mol are 2D molecular graphs, 3D molecular graphs, and atomic elements and coordinates, respectively. For each data set in three-times 5-fold CV validation, MolCLR, GEM, and Uni-Mol models were fine-tuned from the pretrained one downloaded from the refs (72−74).

In the Uni-Mol models, three conformer types were tested in combination with the same recorded compound property values: the ground-truth, global minimum, and RMSD maximum (RMSD max.). In the GEM models, in addition to three conformation types, the nonaggregation method was also tested. As shown in Table 3, Uni-Mol models showed significantly higher prediction accuracy than the rest of the conventional descriptor-based approaches. Using the ground-truth conformers to train the Uni-Mol model gave the best prediction accuracy for all of the tested properties. Specifically, for the dipole moment prediction, whose values highly depended on conformation, the Uni-Mol model using the ground-truth conformation reached an average *R*^2^ value of 0.933. For the less conformation-dependent properties, energy, and enthalpy, the Uni-Mol models reached 0.98 and 0.99, irrespective of conformation types.

**Table 3:** Averaged Prediction Accuracy (*R*^*2*^) of the GEM, Uni-Mol, and MolCLR Models for 15 Test Sets of the PQC Data Set Six Property Predictions^a^

		dipole moment	HOMO	HOMO–LUMO gap	LUMO	energy	enthalpy
GEM	ground-truth	0.499 (0.010)	0.917 (0.002)	0.961 (0.002)	0.971 (0.002)	0.996 (0.001)	0.996 (0.001)
	global min. (2.25 Å)	0.472 (0.009)	0.898 (0.004)	0.955 (0.001)	0.966 (0.003)	0.996 (0.001)	0.996 (0.001)
	RMSD max. (2.91 Å)	0.467 (0.009)	0.896 (0.004)	0.954 (0.002)	0.965 (0.003)	0.996 (0.001)	0.996 (0.001)
	nonaggregation	0.500 (0.007)	0.915 (0.002)	0.963 (0.001)	0.973 (0.001)	**0.997 (0.000)**	**0.997 (0.001)**
Uni-Mol	ground-truth	**0.933 (0.003)**	**0.986 (0.001)**	**0.964 (0.001)**	**0.986 (0.001)**	0.987 (0.002)	0.993 (0.001)
	global min. (2.25 Å)	0.544 (0.006)	0.938 (0.001)	0.944 (0.001)	0.973 (0.001)	0.987 (0.002)	0.993 (0.001)
	RMSD max. (2.91 Å)	0.548 (0.006)	0.940 (0.001)	0.945 (0.001)	0.973 (0.001)	0.986 (0.002)	0.993 (0.001)
MolCLR		0.512 (0.009)	0.924 (0.002)	0.963 (0.001)	0.977 (0.001)	0.978 (0.001)	0.979 (0.001)
RF using ECFP4 count		0.516 (0.006)	0.867 (0.005)	0.939 (0.001)	0.956 (0.001)	0.960 (0.002)	0.941 (0.002)

a The RF model using the ECFP4 count is also reported as a comparison. The standard deviations are provided in parentheses. The RMSD is calculated from the ground-truth conformation and the average RMSD value for all compounds in the parentheses of the title column. The best accuracy value per target is highlighted in bold, and the second-best value is underlined

The high accuracy for the dipole moment using the ground-truth conformation had drastically dropped to around 0.54 when trained on the wrong conformers (Global min and RMSD max.). However, even when using the wrong conformers, the prediction accuracy was higher than that of the 2D ECFP4 count. This performance was comparable with MBTR in combination with RF using the ground-truth conformation (Table 1), suggesting the usefulness of this method, even when using nonaccurate conformers. This finding was consistent with several property and activity prediction benchmark results, such as toxicity and activity prediction,^22^ where ground-truth conformation was unknown.

Comparing the performance of Uni-Mol models with the RF models using ECFP for the properties in Table 1, Uni-Mol models, including those using nonaccurate single conformers, were generally superior. Our benchmark calculation revealed that the Uni-Mol architecture led to QC property prediction models with high accuracy, in particular, for the two properties: the dipole moment and HOMO.

Among 2D-based methods, MolCLR outperformed the ECFP count in combination with RF for the five properties except for the dipole moment, indicating that MolCLR achieved a better 2D representation than ECFP for the QC property prediction.

GEM, a 3D representation-based GNN, performed equally to Uni-Mol for the two least conformationally dependent properties, energy, and enthalpy. However, for the other four properties, it performed worse than MolCLR: a 2D representation. GEM with the ground-truth conformation showed performance similar to that of the nonaggregation approach. This indicated that GEM could not extract the 3D information responsible for property value generation. The results of hypothesis testing for the methods in Table 3 are reported from Figures S20 to S25.

To summarize models’ prediction accuracies for the PQC data set, Uni-Mol with the ground-truth conformation gave the best prediction accuracy for the dipole moment, HOMO, HOMO–LUMO gap, and LUMO, while for the energy and enthalpy, GEM using the nonaggregation method showed the best prediction accuracy. However, GEM was not sensitive to conformational differences for these two target properties, as even using the RMSD max conformation reached similar prediction accuracy values (Table 3). For each target property, the scatter plot of the predicted values derived by the best-performed model against the true (calculated) values is provided in Figure S4. Flat lines appearing in Figure S4 for Uni-Mol, corresponding to predicting the same property values, were due to a robust scaler implemented in Scikit-learn to scale predictions to be robust against outliers.

#### 3.2.4 Prediction Accuracy for Extrapolated Regions of Chemical Space

**Figure 4:**
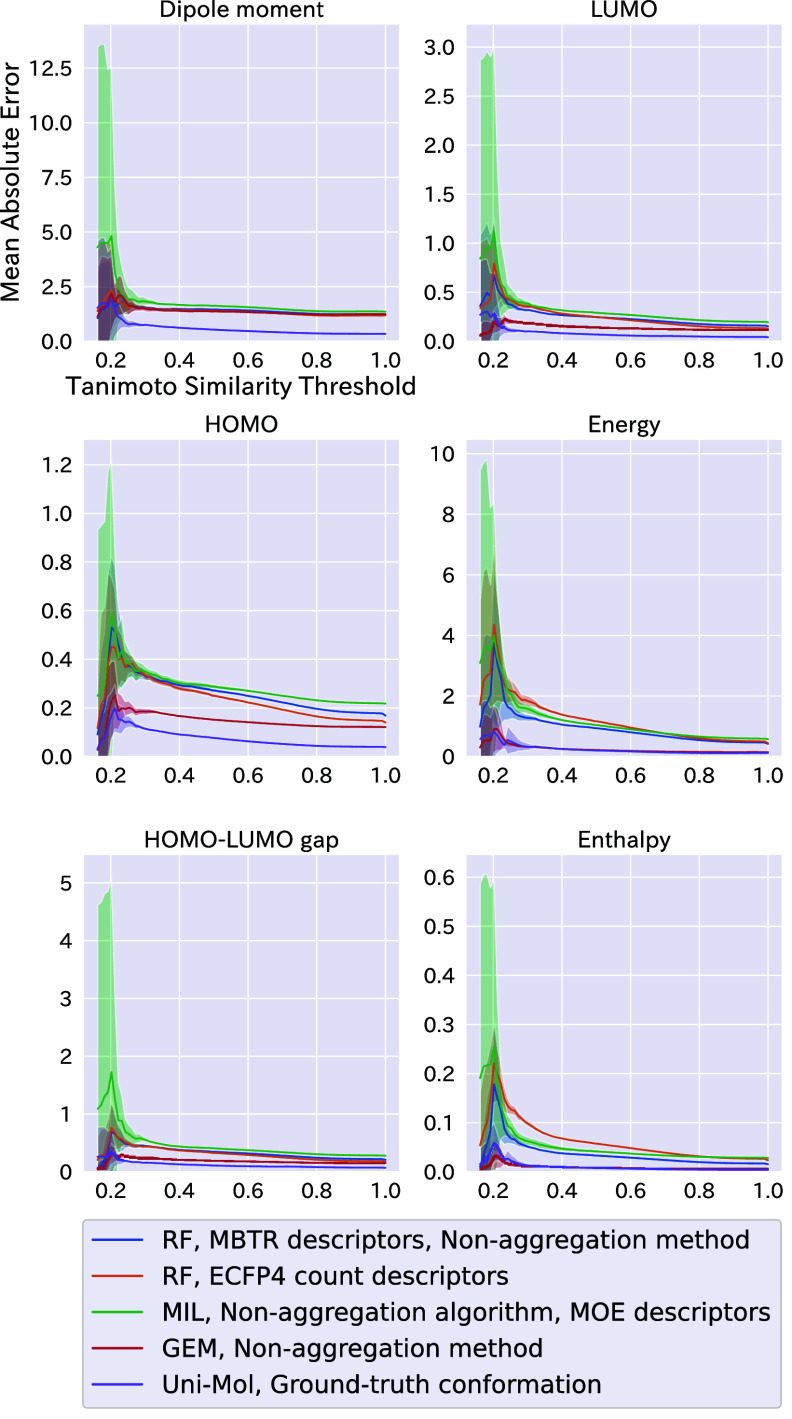
Transition of prediction accuracy against similarity to the training data set. For the six QC properties, mean absolute error (MAE) values for test data sets were shown. A test data set consisted of only compounds with a Tanimoto similarity value less than a threshold indicated on the *x*-axis. The Tanimoto similarity represents the highest Tanimoto similarity value to the training compounds. For each method and property, the average MAE values are represented as a line and the standard deviation as a shaded area.

The maximum Tanimoto similarity to the training data set (nearest neighbor compound) using ECFP4 was calculated to understand the prediction accuracy for test compounds regarding the similarity to the training data set. MAE values for the test data sets where only compounds with similarity values less than the threshold are shown as lines in Figure 4. The distribution of the maximum Tanimoto similarity values between test compounds and the training and examples of specific pairs are shown in Figure S2. As shown in Figure 4, when the threshold value was 1.0, the MAE was calculated using all test compounds, matching the prediction accuracy values in Tables 1–3. As the threshold decreased, the MAE values increased, reflecting reduced prediction accuracy for structurally dissimilar compounds.

For all properties, the Uni-Mol models trained and tested on the ground-truth conformation showed the smallest error for even low Tanimoto similarity compounds, i.e., high extrapolation ability. Overall, the ECFP4 count shows the highest negative steep among the shown methods: RF with the MBTR descriptors using nonaggregation methods, MIL nonaggregation algorithm using all conformers, GEM using all conformers, and Uni-Mol using the ground-truth conformations. This indicated that the 2D representation was more vulnerable to inaccuracy for the extrapolation region than the other approaches. This might be because the compound selection from the test data set was based on ECFP similarity in Figure 4.

### 3.3 Application with the MP Data Set

#### 3.3.1 Aggregation Methods for 3D Descriptors

Table 4 reports the prediction accuracy of RF models for the MP data set using the MOE, Pmapper, 3D-MoRSE, and MBTR descriptors. Prediction errors measured by *R*^2^ and its variation among split data sets (25 data sets) were greater than those for the PQC data set. Overall, MOE descriptors exhibited the highest accuracy, followed by MBTR, 3D-MoRSE, and Pmapper. The average *R*^2^ values and the following statistical hypothesis testing indicated that the best aggregation method could not be determined (Table 4) except for the Pmapper descriptor. For Pmapper, the mean aggregation contributed to higher prediction accuracy than the other aggregation methods (Figure S26), although the prediction accuracy using this descriptor was much lower than that of the MOE. Evaluating the prediction accuracy in terms of the MAE reached the same conclusion as above. Table S174 reports the MAE values corresponding to those in Table 4. The average MAE indicated that aggregating the MOE descriptors by Boltzmann weight, mean, global minimum, and nonaggregation was more effective than the random conformation. However, no clear trend was observed among the aggregation methods when different descriptors were used (Table S174), although the mean aggregation was relatively superior to the other methods. The *p*-value by ANOVA to Table S174 was 0.0630, which did not support a performance difference among the aggregation methods at a significance level of 0.05. The inconsistent performances of the aggregation methods among representations may be attributed to the small size of the data set or unknown ground truth conformation. The ANOVA was performed for the five aggregation methods, including the nonaggregation method and the four 3D descriptors (Table 4). Each cell contained 25 evaluation metric values for calculating the statistics. The *p*-value for the difference in aggregation methods was 0.3154, which did not support a performance difference among aggregation methods at a significance level of 0.05.

**Table 4:** Average prediction Accuracy (*R*^2^) of Random Forest (RF) Models for 25 Test Sets of the MP Data Sets Property (Melting Point) Prediction^a^

		MOE	Pmapper	3D-MoRSE	MBTR
aggregation method	Boltzmann weight	0.759 (0.078)	0.452 (0.139)	0.601 (0.151)	0.584 (0.115)
	mean	0.747 (0.093)	0.480* (0.139)	**0.618 (0.144)**	**0.621 (0.105)**
	global minimum	**0.764 (0.075)**	0.436 (0.157)	0.595 (0.133)	0.565 (0.132)
	random	0.735 (0.088)	0.430 (0.147)	0.559 (0.153)	0.577 (0.096)
nonaggregation		0.716 (0.113)	0.425 (0.156)	0.523 (0.195)	0.619 (0.166)

a The standard deviations are provided in parentheses. All methods used MOE 117 descriptors, Pmapper 575 descriptors, 3D-MoRSE 160 descriptors, or MBTR 950 descriptors. The best accuracy value per descriptor is highlighted in bold, and the second-best is underlined. * represents statistically higher than the next best value at a significance level of 5%.

The whole results for the training sets are presented from Tables S33 to S38, and the whole results for the test sets are presented from Tables S128 to S141. The results of pairwise hypothesis testing for Table 4 are presented from Figures S26 to S29.

#### 3.3.2 MolCLR, GEM, and Uni-Mol Models

In addition to RF, Table 5 shows the prediction accuracy for the MLP, MolCLR, GEM, and Uni-Mol models, where the MLP models employed various descriptors, while the other NN models employed two types of conformations. Clearly, the 3D information contributed to prediction performance improvement for these models. MOE nonaggregation and MOE mean were better than MolCLR and the ECFP4 count in combination with MLP or RF. Although the 2D GNN model of MolCLR also showed comparable performance to that of ECFP4, the MolCLR accuracy was lower than GEM, irrespective of input conformer types.

**Table 5:** Comparison of Average Prediction Accuracy (*R*^2^) for 25 Test Sets of the MP Data Sets Property (Melting Point) Prediction among ML Models^a^

MLP	MOE mean	0.631 (0.158)
	MOE nonaggregation	0.724 (0.102)
	Pmapper mean	–0.074 (0.415)
	Pmapper nonaggregation	0.440 (0.200)
	3D-MoRSE mean	0.712 (0.117)
	3D-MoRSE nonaggregation	0.691 (0.122)
	ECFP4 count	0.372 (0.257)
RF	MOE Boltzmann weight	0.759 (0.078)
	MOE global minimum	0.764 (0.075)
	ECFP4 count	0.638 (0.100)
GEM	global min.	0.732 (0.120)
	nonaggregation	0.734 (0.132)
Uni-Mol	global min.	**0.780 (0.078)**
	nonaggregation	0.755 (0.089)
MolCLR		0.632 (0.149)

a The standard deviations are provided in parentheses. Random Forest (RF) model using MOE descriptors and ECFP4 count, Muti-layer perceptron (MLP) using MOE, Pmapper, 3D-MoRSE descriptors, and ECFP4 count. GEM and Uni-Mol models were built using the global minimum conformers or with nonaggregation approaches. The MolCLR is also reported as a comparison. The best accuracy value per target is highlighted in bold, and the second-best is underlined. * represents statistically higher than the next best at a significance level of 5%

Regarding the aggregation methods for these NNs, there was no clear advantage of the nonaggregation method over the aggregation methods in *R*^2^ for MLP, GEM, and Uni-Mol (Table 5 and Figure S30). The large variation in prediction accuracy across all methods illustrated the difficulty of the methods’ comparison. The results of hypothesis testing for Table 5 are presented in Figure S30. However, when evaluating MAE values between the aggregation methods, the nonaggregation method consistently outperformed the aggregation methods for MLP, GEM, Uni-Mol, and Uni-Mol and GEM nonaggregation performed the best (Table S175). This discrepancy might be due to the limited data size and *R*^2^ being relatively sensitive to outliers, indicating that the superiority of aggregation methods for NNs could not be determined from the MP data set analysis.

To summarize prediction accuracies measured by average *R*^2^ for the MP data set, Uni-Mol using the global minimum conformation gave the best prediction accuracy for the MP prediction, and the second-best was the RF with MOE descriptors from the global minimum conformation. However, the statistical testing showed no superiority of the Uni-Mol model to the RF model. The plots comparing predicted values with actual values for these best-performed models are provided in Figure S5.

### 3.4 Application with APTC-1 and APTC-2 Data Sets

#### 3.4.1 Aggregation Methods for 3D Descriptors

Table 6 reports prediction accuracy for the APTCs data sets using MOE, Pmapper, 3D-MoRSE, and MBTR descriptors in combination with RF. Prediction accuracy for the five times 5 CV trials in APTC-1 varied, as shown in Table 6. Overall, Pmapper descriptors performed the best, and the MOE descriptors performed the second best among the four descriptor sets. The nonaggregated approach, in combination with Pmapper descriptors, showed the highest accuracy, followed by the mean aggregation for APTCs data sets. For APTC-1, this combination reached 0.508 in *R*^2^ of ΔΔ*G*^‡^, and for APTC-2, 0.721. The results of the comparison of aggregation methods were consistent with those obtained from the PQC data set. The nonaggregation method yielded the best performance, followed by the mean method. Although the nonaggregation method positively worked as data augmentation for the Pmapper descriptors, it did not work for the MOE descriptors for the APTC-1 data set.

**Table 6:** Averaged Prediction Accuracy (*R*^2^) of Random Forest (RF) Models for 25 Test Sets for the APTC-1&40 Test Points for the APTC-2 Data Set Property (ΔΔ*G*^‡^) Prediction^a^

		APTC-1				APTC-2			
		MOE	Pmapper	3D-MoRSE	MBTR	MOE	Pmapper	3D-MoRSE	MBTR
aggregation method	Boltzmann weight	0.271 (0.247)	0.355 (0.249)	0.077 (0.313)	0.226 (0.210)	**0.430**	0.364	0.305	0.502
	mean	0.302 (0.274)	0.440 (0.157)	0.265* (0.229)	**0.287 (0.243)**	0.314	0.612	**0.476**	0.529
	global minimum	0.203 (0.262)	0.329 (0.239)	0.086 (0.264)	0.212 (0.223)	0.389	0.319	0.234	0.495
	random	**0.346 (0.200)**	0.405 (0.170)	0.153* (0.286)	0.140 (0.254)	0.292	0.377	0.370	**0.544**
nonaggregation		0.094 (0.335)	0.508* (0.230)	–0.365 (0.442)	0.250 (0.344)	0.417	**0.721**	–0.310	0.533

a For APTC-1, the standard deviations are provided in parentheses. All methods used MOE 117 descriptors, Pmapper 1202/1556 (APTC-1/APTC-2) descriptors, 3D-MoRSE 160 descriptors, or MBTR 2310 descriptors. The best accuracy value per descriptor is highlighted in bold, and the second-best value is underlined. * represents statistically higher than the next best value at a significance level of 5% (only for APTC-1).

Unlike the PQC data set, Pmapper outperformed the other 3D descriptors. Pmapper represents triplets of vertices consisting of any atoms and the centers of 5- and 6-membered aromatic rings. This suggested that the appropriate choice of descriptors varies depending on the predicted property and that the rest of the descriptors may be inadequate for representing the objective variable.

Furthermore, ANOVA was performed to examine whether there were differences among the aggregation methods: the five aggregation methods including the nonaggregation method. In the APTC-1 data set, each cell, consisting of a descriptor and an aggregation method, contained 25 evaluation values. Similarly, in the APTC-2 data set, each combination had 40 points, for a total of 160 data points. The *p*-value for the APTC-1 data set was 0.0001725, showing a performance difference in aggregation methods in the APTC-1 data set. Table S176 reports MAE values corresponding to Table 6, and almost the same conclusions were derived as in *R*^2^. The *p*-value by ANOVA for the APTC-1 data set in Table S176 was 0.0213, and for APTC-2 it was 0.955, showing a performance difference in aggregation methods for the APTC-1 data set, while no significant difference was observed for the APTC-2 data set.

The whole prediction accuracy for the training sets of the APTCs data sets is reported from Tables S39 to S50, and the whole results for the test sets from Tables S142 to S173. The hypothesis testing results for the APTC-1 test sets in Table 6 are also provided from Figures S31 to S34.

#### 3.4.2 2D Descriptors Comparable to the 3D Pmapper Descriptor and Neural-Network Model Accuracy

2D molecular descriptors focusing on chemical graph topologies were used for the performance comparison to understand the reason for the high prediction accuracy of the Pmapper descriptors. Furthermore, the three neural networks were introduced to compare the prediction accuracy. Table 7 summarizes the results of prediction using MIL, MolCLR, GEM, and Uni-Mol with various descriptors, including ECFP4 bit, ECFP4 count, and 2D PFP, in addition to RF. Among the 2D descriptors in combination with RF, the 2D PFP descriptor, which can be regarded as a topological version of the Pmapper descriptor, performed the best. For APTC-2, the 2D descriptor performed as well as the 3D-MoRSE descriptors, indicating that the conformation was not as important as the topological pharmacophore for this data set.

**Table 7:** Comparison of Averaged Prediction Accuracy (*R*^2^) for 25 Test Sets for the APTC-1&40 Test Points for the APTC-2 Data Sets Property (ΔΔ*G*^‡^) Prediction among ML Models^a^

		APTC-1	APTC-2
MLP	MOE mean	0.099 (0.364)	0.065
	MOE nonaggregation	0.234 (0.350)	0.763
	Pmapper mean	0.576 (0.144)	0.249
	Pmapper nonaggregation	0.723* (0.144)	0.765
	3D-MoRSE mean	0.322 (0.309)	0.669
	3D-MoRSE nonaggregation	0.197 (0.252)	**0.765**
	ECFP4 bit	0.426 (0.294)	0.383
	ECFP4 count	0.208 (0.496)	0.309
	2D PFP	0.304 (0.261)	0.319
RF	Pmapper mean	0.440 (0.157)	0.612
	Pmapper nonaggregation	0.508 (0.230)	0.721
	ECFP4 bit	0.429 (0.310)	0.556
	ECFP4 count	0.433 (0.307)	0.473
	2D PFP	0.543 (0.207)	0.754
GEM	global min.	0.124 (0.286)	0.202
	nonaggregation	0.540 (0.268)	0.362
Uni-Mol	global min.	0.243 (0.211)	0.237
	nonaggregation	**0.760 (0.144)**	0.625
MolCLR		0.278 (0.262)	–0.027

a Random Forest (RF) model using MOE descriptors, Muti-layer perceptron (MLP) using MOE, Pmapper, 3D-MoRSE, MBTR descriptors. Both models also used 3 different 2D descriptors: ECFP4 as bit vectors, ECFP4 as count-up vectors, and 2D PEP. GEM and Uni-Mol models were built using the global minimum conformers or with nonaggregation approaches. The MolCLR is also reported as a comparison. The best accuracy value per target is highlighted in bold, and the second-best value is underlined. * represents statistically higher than the next best value at the significance level of 5% (only for APTC-1).

For APTC-1, where the Pmapper descriptor in combination with MLP outperformed 2D PEP, Uni-Mol with nonaggregation achieved the highest prediction accuracy. The Uni-Mol and GEM using a single conformer showed poor prediction accuracy for both data sets. This demonstrates that Uni-Mol could efficiently learn from the information on multiple conformations for the small-sized data set, even when the relevant conformation for the property is unknown. However, for APTC-2, Uni-Mol exhibited slightly lower prediction accuracy and greater variability than MLP with 3D-MoRSE nonaggregation descriptors and MLP with Pmapper nonaggregation descriptors, as shown in Figure S7, likely due to the smaller data size of APTC-2, which might hinder sufficient fine-tuning.

As a modeling method, the superiority of MLP to RF was not consistent among different molecular representations and data sets. For APTC-1, the MLP model using the Pmapper descriptors performed better. However, for ECFP4 and 2D PFP, RF performed better than MLP. The results of hypothesis testing for the APTC-1 test data set in Table 7 are reported in Figure S35. In addition to the evaluation in terms of *R*^2^, Table S177 reports the MAE values corresponding to Table 7, leading to the same conclusions as derived using *R*^2^.

To summarize prediction accuracies in *R*^2^ for the APTCs data sets, Uni-Mol using the nonaggregation method showed the best prediction accuracy for the APTC-1 data set, and MLP with Pmapper descriptors using the nonaggregation method was the second-best. For the APTC-2 data set, MLP with 3D-MoRSE descriptors using the nonaggregation method was the best, and MLP with the Pmapper descriptors using the nonaggregation method was the second best. The plots comparing predicted values with actual values for the best performance model are provided in Figure S6 for APTC-1, and Figure S7 for the APTC-2 data set.

The complete outcomes of prediction accuracy for all data sets and methods are reported in Figures S36–S70. For each of the PQC, MP, and APTCs data sets and accuracy metrics, further categorized into training and test, prediction accuracy distributions for all methods are reported. The order of the methods in these figures is provided in Table S178. Each distribution formed a violin plot for the PQC, MP, APTC1, and APTC2 data sets, consisting of 15, 25, 25, and 40 data points, corresponding to a total number of data split times.

## 4 Conclusions

To comprehend the impact of the conformers of a compound on data-driven property prediction models, a large-scale compound data set where a target property highly depends on the conformation along with its corresponding conformation is necessary. For this purpose, we compiled a QM property data set (the PQC data set) from the PubChemQC database and conducted extensive validations on the utilization of multiple conformers. From the analysis using the PQC data set, employing individual 3D molecular descriptors of the multiple conformers of a compound (nonaggregation method) showed the highest prediction accuracy among molecular aggregation methods and comparable performance to the 2D ECFP4 counting descriptor. When predicting QC properties from descriptors, using the ground-truth conformation overall did not produce higher prediction accuracy than the nonaggregation method, possibly due to the underrepresentation of conformational information by the 3D descriptors with one exception: the MBTR descriptor using the ground-truth conformation for the dipole moment prediction. For this conformation-sensitive target property, using the ground-truth conformation contributed to the performance improvement, although the difference in prediction accuracy between MBTR and the ECFP count was marginal. Uni-Mol models: neural networks utilizing atomic coordinates and elements as input outperformed other methods regardless of conformer types. Additionally, the Uni-Mol model, when trained on the ground-truth conformation, exhibited significantly higher prediction accuracy compared with the models trained on incorrect conformations. For one of the enantioselectivity chemical reaction data sets, where important conformation was unknown, 3D-MoRSE, Pmapper, and 2D topological pharmacophore descriptors showed comparable high prediction accuracy, meaning less importance of geographical information. For the other data set, a Uni-Mol model with nonaggregation showed the best performance, possibly due to compensating the small-sized training data set, while using single conformation significantly decreased prediction accuracy. Finally, for the melting point data set, the use of 3D information contributed to a higher prediction accuracy than that of 2D descriptors. For this data set, the mean aggregation showed a relatively better performance than other approaches, although no significant difference was observed among the aggregation methods due to a large variation in prediction accuracy among different data sets.

## Data Availability

The PQC, the MP, the APTC-1, and the APTC-2 data sets with descriptors are provided in the ZENODO repository: https://zenodo.org/records/14575682. The codes of data curation, model construction (RF, Elastic Net, PLS, SVM, MIL, MolCLR, GEM, Uni-Mol), and visualization described in this work are available at https://github.com/YuHamakawa/Conformation-Importance-ML-Models.

## Supporting Information Available

Description of MOE 3D descriptors (Table S1); specification of vertices in Pmapper descriptors (Table S2); description of 3D-MoRSE, MBTR and 2D PFP descriptors (Tables S3–S5); distributions of the PQC, MP, and APTCs data sets and the diversity of the generated conformers (Figures S1–S2); similarity distribution of compounds between the training and the test data of the PQC data set in Seed 42-Fold 0 (Figure S3); hyperparameters of property prediction models (Table S6); prediction against experimental value plots for the PQC, MP, APTC-1, and APTC-2 data set (Figures S4–S7); averaged prediction accuracy (*R*^2^) of ML models for 15 training sets of the PQC data set for six properties using 3D and 2D representations including NN models (Tables S7–S32); averaged prediction accuracy (*R*^2^) of ML models for 25 training sets of the MP data set for MP prediction using 3D and 2D representations including NN models (Tables S33–S38); averaged prediction accuracy (*R*^2^) of ML models for 25 training sets of the APTC-1 data set property (ΔΔ*G*^‡^) prediction using 3D and 2D representations (Tables S39–S44); and for 40 training sets of the APTC-2 data sets (Tables S45–S50); averaged prediction accuracy (*R*^2^, MAE, RMSE) of ML models for 15 test sets of the PQC data set for six properties using 3D and 2D representations including NN models (Tables S51–S127); averaged prediction accuracy (*R*^2^, MAE, RMSE) of ML models for 25 test sets of the MP data set property using 3D and 2D representations including NN models (Tables S128–S141); averaged prediction accuracy (*R*^2^, MAE, RMSE) of ML models for 25 test sets of the APTC-1 data set using 3D and 2D representations including NN models (Tables S142–S157); for the APTC-2 data set (Tables S158–S173); averaged prediction accuracy in MAE corresponding to Tables 4–7 (Tables S174–S177); heatmaps of *p*-values for Table 1 (Figures S8–S13); for Table 2 (Figure S14–S19); for Table 3 (Figures S20–S25); for Table 4 (Figures S26–S29); for Table 5 (Figure S30); for Table 6 (Figures S31–S34); for Table 7 (Figure S35); order of models in Figure S36–S70 (Table S178); violin plots of training prediction accuracy (*R*^2^) for the PQC data set (Figures S36–S41); for the MP data set (Figure S42); for the APTC-1 data set (Figure S43); for the APTC-2 data set (Figure S44); violin plots of test prediction accuracy (*R*^2^, MAE, RMSE) for the PQC data set (Figures S45–S62); for the MP data set (Figures S63–S65); for the APTC-1 data set (Figure S66–S68); and for the APTC-2 data (only MAE and RMSE) (Figures S69 and S70) (PDF)
