# Supplementary material for: Understanding Conformation Importance in Data-Driven Property Prediction Models
Source: J Chem Inf Model. 2025 Mar 18;65(7):3388–404. doi: 10.1021/acs.jcim.5c00018 (PMC12004525; doi:10.1021/acs.jcim.5c00018)
Supplement: Supplementary file 1 — ci5c00018_si_001.pdf [file ci5c00018_si_001.pdf]

Supporting Information for

Understanding Conformation Importance in Data-driven Property Prediction Models

Yu Hamakawa[1] and Tomoyuki Miyao[1,2]*

[1]Graduate School of Science and Technology, Nara Institute of Science and Technology, 8916-5 Takayama-cho, Ikoma, Nara, 630-0192, Japan.

[2]Data Science Center, Nara Institute of Science and Technology, 8916-5 Takayama-cho, Ikoma, Nara, 630-0192, Japan.

*Corresponding author:

E-mail: miyao@dsc.naist.jp

List of Figures

Figure S1. Distributions of the PQC data set and the diversity of the generated conformers. 26

Figure S2. Distributions of the MP and APTCs data sets. 27

Figure S3. Similarity distribution of compounds between the training and the test data of the PQC data set in Seed 42-Fold 0. 28

Figure S4. Prediction against experimental value plots for the PQC data set. Uni-Mol with ground-truth conformations is used for predicting dipole moment, HOMO, HOMO-LUMO gap, and LUMO prediction, while GEM with non-aggregation conformations is used for energy and enthalpy prediction. 32

Figure S5. Prediction against experimental value plots for the MP data set, using (a) Uni-Mol with global minimum conformations, (b) RF with MOE global minimum descriptors, and (c) Uni-Mol with non-aggregation conformations. 33

Figure S6. Prediction against experimental value plots for the APTC-1 data set, using (a) Uni-Mol with non-aggregation conformations and (b) MLP with Pmapper non-aggregation descriptors. 34

Figure S7. Prediction against experimental value plots for the APTC-2 data set, using (a) MLP with 3D-MoRSE non-aggregation descriptors, (b) MLP with Pmapper non-aggregation descriptors, and (c) Uni-Mol with non-aggregation conformations. 35

Figure S8. Heatmap of p-values corresponding to Table 1, showing the significance of dipole moment prediction accuracy (R^2) for 15 test sets of the PQC data set. 207

Figure S9. Heatmap of p-values corresponding to Table 1, showing the significance of HOMO prediction accuracy (R^2) for 15 test sets of the PQC data set. 208

Figure S10. Heatmap of p-values corresponding to Table 1, showing the significance of HOMO-LUMO gap prediction accuracy (R^2) for 15 test sets of the PQC data set. 209

Figure S11. Heatmap of p-values corresponding to Table 1, showing the significance of LUMO prediction accuracy (R^2) for 15 test sets of the PQC data set. 210

Figure S12. Heatmap of p-values corresponding to Table 1, showing the significance of energy prediction accuracy (R^2) for 15 test sets of the PQC data set. 211

Figure S13. Heatmap of p-values corresponding to Table 1, showing the significance of enthalpy prediction accuracy (R^2) for 15 test sets of the PQC data set. 212

Figure S14. Heatmap of p-values corresponding to Table 2, showing the significance of dipole moment prediction accuracy (R^2) for 15 test sets of the PQC data set. 213

Figure S15. Heatmap of p-values corresponding to Table 2, showing the significance of HOMO prediction accuracy (R^2) for 15 test sets of the PQC data set. 214

Figure S16. Heatmap of p-values corresponding to Table 2, showing the significance of HOMO-LUMO gap prediction accuracy (R^2) for 15 test sets of the PQC data set...................................... 215

Figure S17. Heatmap of p-values corresponding to Table 2, showing the significance of LUMO prediction accuracy (R^2) for 15 test sets of the PQC data set. 216

Figure S18. Heatmap of p-values corresponding to Table 2, showing the significance of energy prediction accuracy (R^2) for 15 test sets of the PQC data set. 217

Figure S19. Heatmap of p-values corresponding to Table 2, showing the significance of enthalpy prediction accuracy (R^2) for 15 test sets of the PQC data set. 218

Figure S20. Heatmap of p-values corresponding to Table 3, showing the significance of dipole moment prediction accuracy (R^2) for 15 test sets of the PQC data set. 219

Figure S21. Heatmap of p-values corresponding to Table 3, showing the significance of HOMO prediction accuracy (R^2) for 15 test sets of the PQC data set. 220

Figure S22. Heatmap of p-values corresponding to Table 3, showing the significance of HOMO-LUMO gap prediction accuracy (R^2) for 15 test sets of the PQC data set...................................... 221

Figure S23. Heatmap of p-values corresponding to Table 3, showing the significance of LUMO prediction accuracy (R^2) for 15 test sets of the PQC data set. 222

Figure S24. Heatmap of p-values corresponding to Table 3, showing the significance of energy prediction accuracy (R^2) for 15 test sets of the PQC data set. 223

Figure S25. Heatmap of p-values corresponding to Table 3, showing the significance of enthalpy prediction accuracy (R^2) for 15 test sets of the PQC data set. 224

Figure S26. Heatmap of p-values corresponding to Table 4, showing the significance of property (melting point) prediction accuracy (R^2) using MOE descriptors for 25 test sets of the MP data set. 225

Figure S27. Heatmap of p-values corresponding to Table 4, showing the significance of property (melting point) prediction accuracy (R^2) using Pmapper descriptors for 25 test sets of the MP data set. 226

Figure S28. Heatmap of p-values corresponding to Table 4, showing the significance of property (melting point) prediction accuracy (R^2) using 3D-MoRSE descriptors for 25 test sets of the MP data set. 227

Figure S29. Heatmap of p-values corresponding to Table 4, showing the significance of property (melting point) prediction accuracy (R^2) using MBTR descriptors for 25 test sets of the MP data set. 228

Figure S30. Heatmap of p-values corresponding to Table 5, showing the significance of property (melting point) prediction accuracy (R^2) for 25 test sets of the MP data set. 229

Figure S31. Heatmap of p-values corresponding to Table 6, showing the significance of property ($\Delta\Delta G^{\ddagger}$) prediction accuracy ($R^2$) using MOE descriptors for 25 test sets of the APTC-1 data set.. 230

Figure S32. Heatmap of p-values corresponding to Table 6, showing the significance of property ($\Delta\Delta G^{\ddagger}$) prediction accuracy ($R^2$) using Pmapper descriptors for 25 test sets of the APTC-1 data set. 231

Figure S33. Heatmap of p-values corresponding to Table 6, showing the significance of property ($\Delta\Delta G^{\ddagger}$) prediction accuracy ($R^2$) using 3D-MoRSE descriptors for 25 test sets of the APTC-1 data set. 232

Figure S34. Heatmap of p-values corresponding to Table 6, showing the significance of property ($\Delta\Delta G^{\ddagger}$) prediction accuracy ($R^2$) using MBTR descriptors for 25 test sets of the APTC-1 data set.233

Figure S35. Heatmap of p-values corresponding to Table 7, showing the significance of property ($\Delta\Delta G^{\ddagger}$) prediction accuracy ($R^2$) for 25 test sets of the APTC-1 data set. 234

Figure S36. Violin plot of prediction accuracy (R^2) for 15 training sets of the PQC data set dipole moment prediction. 251

Figure S37. Violin plot of prediction accuracy (R^2) for 15 training sets of the PQC data set HOMO prediction. 252

Figure S38. Violin plot of prediction accuracy (R^2) for 15 training sets of the PQC data set HOMO-LUMO gap prediction. 253

Figure S39. Violin plot of prediction accuracy (R^2) for 15 training sets of the PQC data set LUMO prediction. 254

Figure S40. Violin plot of prediction accuracy (R^2) for 15 training sets of the PQC data set energy prediction. 255

Figure S41. Violin plot of prediction accuracy (R^2) for 15 training sets of the PQC data set enthalpy prediction. 256

Figure S42. Violin plot of prediction accuracy (R^2) for 25 training sets of the MP data set property (melting point) prediction. 257

Figure S43. Violin plot of prediction accuracy (R^2) for 25 training sets of the APTC-1 data set property ($\Delta\Delta G^{\ddagger}$) prediction. 258

Figure S44. Violin plot of prediction accuracy (R^2) for 40 training sets of the APTC-2 data set property ($\Delta\Delta G^{\ddagger}$) prediction. 259

Figure S45. Violin plot of prediction accuracy (R^2) for 15 test sets of the PQC data set dipole moment prediction. 260

Figure S46. Violin plot of prediction accuracy (MAE) for 15 test sets of the PQC data set dipole moment prediction. 261

Figure S47. Violin plot of prediction accuracy (RMSE) for 15 test sets of the PQC data set dipole moment prediction. 262

Figure S48. Violin plot of prediction accuracy (R^2) for 15 test sets of the PQC data set HOMO prediction. 263

Figure S49. Violin plot of prediction accuracy (MAE) for 15 test sets of the PQC data set HOMO prediction. 264

Figure S50. Violin plot of prediction accuracy (RMSE) for 15 test sets of the PQC data set HOMO prediction. 265

Figure S51. Violin plot of prediction accuracy (R^2) for 15 test sets of the PQC data set HOMO-LUMO gap prediction. 266

Figure S52. Violin plot of prediction accuracy (MAE) for 15 test sets of the PQC data set HOMO-LUMO gap prediction. 267

Figure S53. Violin plot of prediction accuracy (RMSE) for 15 test sets of the PQC data set HOMO-LUMO gap prediction. 268

Figure S54. Violin plot of prediction accuracy (R^2) for 15 test sets of the PQC data set LUMO prediction. 269

Figure S55. Violin plot of prediction accuracy (MAE) for 15 test sets of the PQC data set LUMO

prediction. 270

Figure S56. Violin plot of prediction accuracy (RMSE) for 15 test sets of the PQC data set LUMO prediction. 271

Figure S57. Violin plot of prediction accuracy (R^2) for 15 test sets of the PQC data set energy prediction. 272

Figure S58. Violin plot of prediction accuracy (MAE) for 15 test sets of the PQC data set energy prediction. 273

Figure S59. Violin plot of prediction accuracy (RMSE) for 15 test sets of the PQC data set energy prediction. 274

Figure S60. Violin plot of prediction accuracy (R^2) for 15 test sets of the PQC data set enthalpy prediction. 275

Figure S61. Violin plot of prediction accuracy (MAE) for 15 test sets of the PQC data set enthalpy prediction. 276

Figure S62. Violin plot of prediction accuracy (RMSE) for 15 test sets of the PQC data set enthalpy prediction. 277

Figure S63. Violin plot of prediction accuracy (R^2) for 25 test sets of the MP data set property (melting point) prediction. 278

Figure S64. Violin plot of prediction accuracy (MAE) for 25 test sets of the MP data set property (melting point) prediction. 279

Figure S65. Violin plot of prediction accuracy (RMSE) for 25 test sets of the MP data set property (melting point) prediction. 280

Figure S66. Violin plot of prediction accuracy (R^2) for 25 test sets of the APTC-1 data set property ($\Delta\Delta G^{\ddagger}$) prediction. 281

Figure S67. Violin plot of prediction accuracy (MAE) for 25 test sets of the APTC-1 data set property ($\Delta\Delta G^{\ddagger}$) prediction. 282

Figure S68. Violin plot of prediction accuracy (RMSE) for 25 test sets of the APTC-1 data set property ($\Delta\Delta G^{\ddagger}$) prediction. 283

Figure S69. Violin plot of prediction accuracy (MAE) for 40 test points of the APTC-2 data set property ($\Delta\Delta G^{\ddagger}$) prediction. 284

Figure S70. Violin plot of prediction accuracy (RMSE) for 40 test points of the APTC-2 data set property ($\Delta\Delta G^{\ddagger}$) prediction. 285

List of Tables

Table S1. Description of MOE 3D descriptors. 21

Table S2. Specification of vertices in Pmapper descriptors... 23

Table S3. Description of 3D-MoRSE descriptors... 24

Table S4. Parameters of MBTR descriptors. 24

Table S5. Custom features and bins of 2D PFP descriptors. 25

Table S6. Hyperparameters and search space of Random Forest (RF) models, Elastic Net models, Partial Least Squares (PLS) models, Support Vector Machine (SVM) models, Muti Instance Learning (MIL) models, MolCLR models, GEM models, and Uni-Mol models.............................. 29

Table S7. Averaged prediction accuracy (R^2) of RF and MIL models for 15 training sets of the PQC data set dipole moment prediction using MOE descriptors.. 36

Table S8. Averaged prediction accuracy (R^2) of RF and MIL models for 15 training sets of the PQC data set dipole moment prediction using Pmapper descriptors. 37

Table S9. Averaged prediction accuracy (R^2) of RF and MIL models for 15 training sets of the PQC data set dipole moment prediction using 3D-MoRSE descriptors... 38

Table S10. Averaged prediction accuracy (R^2) of RF and MIL models for 15 training sets of the PQC data set dipole moment prediction using MBTR descriptors. 39

Table S11. Averaged prediction accuracy (R^2) of RF and MIL models for 15 training sets of the PQC data set HOMO prediction using MOE descriptors.. 40

Table S12. Averaged prediction accuracy (R^2) of RF and MIL models for 15 training sets of the PQC data set HOMO prediction using Pmapper descriptors. 41

Table S13. Averaged prediction accuracy (R^2) of RF and MIL models for 15 training sets of the PQC data set HOMO prediction using 3D-MoRSE descriptors. 42

Table S14. Averaged prediction accuracy (R^2) of RF and MIL models for 15 training sets of the PQC data set HOMO prediction using MBTR descriptors. 43

Table S15. Averaged prediction accuracy (R^2) of RF and MIL models for 15 training sets of the PQC data set HOMO-LUMO gap prediction using MOE descriptors.. 44

Table S16. Averaged prediction accuracy (R^2) of RF and MIL models for 15 training sets of the PQC data set HOMO-LUMO gap prediction using Pmapper descriptors. 45

Table S17. Averaged prediction accuracy (R^2) of RF and MIL models for 15 training sets of the PQC data set HOMO-LUMO gap prediction using 3D-MoRSE descriptors... 46

Table S18. Averaged prediction accuracy (R^2) of RF and MIL models for 15 training sets of the PQC data set HOMO-LUMO gap prediction using MBTR descriptors. 47

Table S19. Averaged prediction accuracy (R^2) of RF and MIL models for 15 training sets of the PQC data set LUMO prediction using MOE descriptors. 48

Table S20. Averaged prediction accuracy (R^2) of RF and MIL models for 15 training sets of the PQC data set LUMO prediction using Pmapper descriptors... 49

Table S21. Averaged prediction accuracy (R^2) of RF and MIL models for 15 training sets of the PQC data set LUMO prediction using 3D-MoRSE descriptors... 50

Table S22. Averaged prediction accuracy (R^2) of RF and MIL models for 15 training sets of the PQC data set LUMO prediction using MBTR descriptors.. 51

Table S23. Averaged prediction accuracy (R^2) of RF and MIL models for 15 training sets of the PQC data set energy prediction using MOE descriptors... 52

Table S24. Averaged prediction accuracy (R^2) of RF and MIL models for 15 training sets of the PQC data set energy prediction using Pmapper descriptors.. 53

Table S25. Averaged prediction accuracy (R^2) of RF and MIL models for 15 training sets of the PQC data set energy prediction using 3D-MoRSE descriptors.. 54

Table S26. Averaged prediction accuracy (R^2) of RF and MIL models for 15 training sets of the PQC data set energy prediction using MBTR descriptors... 55

Table S27. Averaged prediction accuracy (R^2) of RF and MIL models for 15 training sets of the PQC data set enthalpy prediction using MOE descriptors. 56

Table S28. Averaged prediction accuracy (R^2) of RF and MIL models for 15 training sets of the PQC data set enthalpy prediction using Pmapper descriptors... 57

Table S29. Averaged prediction accuracy (R^2) of RF and MIL models for 15 training sets of the PQC data set enthalpy prediction using 3D-MoRSE descriptors... 58

Table S30. Averaged prediction accuracy (R^2) of RF and MIL models for 15 training sets of the PQC

data set enthalpy prediction using MBTR descriptors. 59

Table S31. Averaged prediction accuracy (R^2) of RF and MIL models for 15 training sets of the PQC data set six property predictions using ECFP4 count. 60

Table S32. Averaged prediction accuracy (R^2) of MolCLR, GEM, and Uni-Mol models for 15 training sets of the PQC data set six property predictions. 61

Table S33. Averaged prediction accuracy (R^2) of RF and MIL models for 25 training sets of the MP data set property (melting point) prediction using MOE descriptors. 62

Table S34. Averaged prediction accuracy (R^2) of RF and MIL models for 25 training sets of the MP data set property (melting point) prediction using Pmapper descriptors. 63

Table S35. Averaged prediction accuracy (R^2) of RF and MIL models for 25 training sets of the MP data set property (melting point) prediction using 3D-MoRSE descriptors. 64

Table S36. Averaged prediction accuracy (R^2) of RF and MIL models for 25 training sets of the MP data set property (melting point) prediction using MBTR descriptors. 65

Table S37. Averaged prediction accuracy (R^2) of RF and MIL models for 25 training sets of the MP data set property (melting point) prediction using ECFP4 count. 66

Table S38. Averaged prediction accuracy (R^2) of MolCLR, GEM, and Uni-Mol models for 25 training sets of the MP data set property (melting point) prediction. 67

Table S39. Averaged prediction accuracy (R^2) of RF, Elastic Net, PLS, SVM, and MIL models for 25 training sets of the APTC-1 data set property ($\Delta\Delta G^{\ddagger}$) prediction using MOE descriptors. 68

Table S40. Averaged prediction accuracy (R^2) of RF, Elastic Net, PLS, SVM, and MIL models for 25 training sets of the APTC-1 data set property ($\Delta\Delta G^{\ddagger}$) prediction using Pmapper descriptors. 69

Table S41. Averaged prediction accuracy (R^2) of RF, Elastic Net, PLS, SVM, and MIL models for 25 training sets of the APTC-1 data set property ($\Delta\Delta G^{\ddagger}$) prediction using 3D-MoRSE descriptors. 70

Table S42. Averaged prediction accuracy (R^2) of RF, Elastic Net, PLS, SVM, and MIL models for 25 training sets of the APTC-1 data set property ($\Delta\Delta G^{\ddagger}$) prediction using MBTR descriptors. 71

Table S43. Averaged prediction accuracy (R^2) of RF, Elastic Net, PLS, SVM, and MIL models for 25 training sets of the APTC-1 data set property ($\Delta\Delta G^{\ddagger}$) prediction using ECFP4 bit, ECFP4 count, and 2D PFP. 72

Table S44. Averaged prediction accuracy (R^2) of MolCLR, GEM, and Uni-Mol models for 25

training sets of the APTC-1 data set property ($\Delta\Delta G^{\ddagger}$) prediction. 73

Table S45. Averaged prediction accuracy (R^2) of RF, Elastic Net, PLS, SVM, and MIL models for 40 training sets of the APTC-2 data set property ($\Delta\Delta G^{\ddagger}$) prediction using MOE descriptors. 74

Table S46. Averaged prediction accuracy (R^2) of RF, Elastic Net, PLS, SVM, and MIL models for 40 training sets of the APTC-2 data set property ($\Delta\Delta G^{\ddagger}$) prediction using Pmapper descriptors...... 75

Table S47. Averaged prediction accuracy (R^2) of RF, Elastic Net, PLS, SVM, and MIL models for 40 training sets of the APTC-2 data set property ($\Delta\Delta G^{\ddagger}$) prediction using 3D-MoRSE descriptors. 76

Table S48. Averaged prediction accuracy (R^2) of RF, Elastic Net, PLS, SVM, and MIL models for 40 training sets of the APTC-2 data set property ($\Delta\Delta G^{\ddagger}$) prediction using MBTR descriptors......... 77

Table S49. Averaged prediction accuracy (R^2) of RF, Elastic Net, PLS, SVM, and MIL models for 40 training sets of the APTC-2 data set property ($\Delta\Delta G^{\ddagger}$) prediction using ECFP4 bit, ECFP4 count, and 2D PFP. 78

Table S50. Averaged prediction accuracy (R^2) of MolCLR, GEM, and Uni-Mol models for 40 training sets of the APTC-2 data set property ($\Delta\Delta G^{\ddagger}$) prediction. 79

Table S51. Averaged prediction accuracy (R^2) of RF and MIL models for 15 test sets of the PQC data set dipole moment prediction using MOE descriptors. 80

Table S52. Averaged prediction accuracy (MAE) of RF and MIL models for 15 test sets of the PQC data set dipole moment prediction using MOE descriptors. 81

Table S53. Averaged prediction accuracy (RMSE) of RF and MIL models for 15 test sets of the PQC data set dipole moment prediction using MOE descriptors. 82

Table S54. Averaged prediction accuracy (R^2) of RF and MIL models for 15 test sets of the PQC data set dipole moment prediction using Pmapper descriptors. 83

Table S55. Averaged prediction accuracy (MAE) of RF and MIL models for 15 test sets of the PQC data set dipole moment prediction using Pmapper descriptors. 84

Table S56. Averaged prediction accuracy (RMSE) of RF and MIL models for 15 test sets of the PQC data set dipole moment prediction using Pmapper descriptors. 85

Table S57. Averaged prediction accuracy (R^2) of RF and MIL models for 15 test sets of the PQC data set dipole moment prediction using 3D-MoRSE descriptors... 86

Table S58. Averaged prediction accuracy (MAE) of RF and MIL models for 15 test sets of the PQC

data set dipole moment prediction using 3D-MoRSE descriptors.. 87

Table S59. Averaged prediction accuracy (RMSE) of RF and MIL models for 15 test sets of the PQC data set dipole moment prediction using 3D-MoRSE descriptors.. 88

Table S60. Averaged prediction accuracy (R^2) of RF and MIL models for 15 test sets of the PQC data set dipole moment prediction using MBTR descriptors. 89

Table S61. Averaged prediction accuracy (MAE) of RF and MIL models for 15 test sets of the PQC data set dipole moment prediction using MBTR descriptors. 90

Table S62. Averaged prediction accuracy (RMSE) of RF and MIL models for 15 test sets of the PQC data set dipole moment prediction using MBTR descriptors. 91

Table S63. Averaged prediction accuracy (R^2) of RF and MIL models for 15 test sets of the PQC data set HOMO prediction using MOE descriptors.. 92

Table S64. Averaged prediction accuracy (MAE) of RF and MIL models for 15 test sets of the PQC data set HOMO prediction using MOE descriptors.. 93

Table S65. Averaged prediction accuracy (RMSE) of RF and MIL models for 15 test sets of the PQC data set HOMO prediction using MOE descriptors.. 94

Table S66. Averaged prediction accuracy (R^2) of RF and MIL models for 15 test sets of the PQC data set HOMO prediction using Pmapper descriptors. 95

Table S67. Averaged prediction accuracy (MAE) of RF and MIL models for 15 test sets of the PQC data set HOMO prediction using Pmapper descriptors. 96

Table S68. Averaged prediction accuracy (RMSE) of RF and MIL models for 15 test sets of the PQC data set HOMO prediction using Pmapper descriptors. 97

Table S69. Averaged prediction accuracy (R^2) of RF and MIL models for 15 test sets of the PQC data set HOMO prediction using 3D-MoRSE descriptors. 98

Table S70. Averaged prediction accuracy (MAE) of RF and MIL models for 15 test sets of the PQC data set HOMO prediction using 3D-MoRSE descriptors. 99

Table S71. Averaged prediction accuracy (RMSE) of RF and MIL models for 15 test sets of the PQC data set HOMO prediction using 3D-MoRSE descriptors. 100

Table S72. Averaged prediction accuracy (R^2) of RF and MIL models for 15 test sets of the PQC data set HOMO prediction using MBTR descriptors. 101

Table S73. Averaged prediction accuracy (MAE) of RF and MIL models for 15 test sets of the PQC data set HOMO prediction using MBTR descriptors. 102

Table S74. Averaged prediction accuracy (RMSE) of RF and MIL models for 15 test sets of the PQC data set HOMO prediction using MBTR descriptors. 103

Table S75. Averaged prediction accuracy (R^2) of RF and MIL models for 15 test sets of the PQC data set HOMO-LUMO gap prediction using MOE descriptors... 104

Table S76. Averaged prediction accuracy (MAE) of RF and MIL models for 15 test sets of the PQC data set HOMO-LUMO gap prediction using MOE descriptors... 105

Table S77. Averaged prediction accuracy (RMSE) of RF and MIL models for 15 test sets of the PQC data set HOMO-LUMO gap prediction using MOE descriptors... 106

Table S78. Averaged prediction accuracy (R^2) of RF and MIL models for 15 test sets of the PQC data set HOMO-LUMO gap prediction using Pmapper descriptors. 107

Table S79. Averaged prediction accuracy (MAE) of RF and MIL models for 15 test sets of the PQC data set HOMO-LUMO gap prediction using Pmapper descriptors. 108

Table S80. Averaged prediction accuracy (RMSE) of RF and MIL models for 15 test sets of the PQC data set HOMO-LUMO gap prediction using Pmapper descriptors. 109

Table S81. Averaged prediction accuracy (R^2) of RF and MIL models for 15 test sets of the PQC data set HOMO-LUMO gap prediction using 3D-MoRSE descriptors..110

Table S82. Averaged prediction accuracy (MAE) of RF and MIL models for 15 test sets of the PQC data set HOMO-LUMO gap prediction using 3D-MoRSE descriptors..111

Table S83. Averaged prediction accuracy (RMSE) of RF and MIL models for 15 test sets of the PQC data set HOMO-LUMO gap prediction using 3D-MoRSE descriptors..112

Table S84. Averaged prediction accuracy (R^2) of RF and MIL models for 15 test sets of the PQC data set HOMO-LUMO gap prediction using MBTR descriptors. 113

Table S85. Averaged prediction accuracy (MAE) of RF and MIL models for 15 test sets of the PQC data set HOMO-LUMO gap prediction using MBTR descriptors. 114

Table S86. Averaged prediction accuracy (RMSE) of RF and MIL models for 15 test sets of the PQC data set HOMO-LUMO gap prediction using MBTR descriptors. 115

Table S87. Averaged prediction accuracy (R^2) of RF and MIL models for 15 test sets of the PQC

data set LUMO prediction using MOE descriptors. 116

Table S88. Averaged prediction accuracy (MAE) of RF and MIL models for 15 test sets of the PQC data set LUMO prediction using MOE descriptors. 117

Table S89. Averaged prediction accuracy (RMSE) of RF and MIL models for 15 test sets of the PQC data set LUMO prediction using MOE descriptors. 118

Table S90. Averaged prediction accuracy (R^2) of RF and MIL models for 15 test sets of the PQC data set LUMO prediction using Pmapper descriptors. 119

Table S91. Averaged prediction accuracy (MAE) of RF and MIL models for 15 test sets of the PQC data set LUMO prediction using Pmapper descriptors. 120

Table S92. Averaged prediction accuracy (RMSE) of RF and MIL models for 15 test sets of the PQC data set LUMO prediction using Pmapper descriptors. 121

Table S93. Averaged prediction accuracy (R^2) of RF and MIL models for 15 test sets of the PQC data set LUMO prediction using 3D-MoRSE descriptors. 122

Table S94. Averaged prediction accuracy (MAE) of RF and MIL models for 15 test sets of the PQC data set LUMO prediction using 3D-MoRSE descriptors. 123

Table S95. Averaged prediction accuracy (RMSE) of RF and MIL models for 15 test sets of the PQC data set LUMO prediction using 3D-MoRSE descriptors. 124

Table S96. Averaged prediction accuracy (R^2) of RF and MIL models for 15 test sets of the PQC data set LUMO prediction using MBTR descriptors. 125

Table S97. Averaged prediction accuracy (MAE) of RF and MIL models for 15 test sets of the PQC data set LUMO prediction using MBTR descriptors. 126

Table S98. Averaged prediction accuracy (RMSE) of RF and MIL models for 15 test sets of the PQC data set LUMO prediction using MBTR descriptors. 127

Table S99. Averaged prediction accuracy (R^2) of RF and MIL models for 15 test sets of the PQC data set energy prediction using MOE descriptors. 128

Table S100. Averaged prediction accuracy (MAE) of RF and MIL models for 15 test sets of the PQC data set energy prediction using MOE descriptors. 129

Table S101. Averaged prediction accuracy (RMSE) of RF and MIL models for 15 test sets of the PQC data set energy prediction using MOE descriptors. 130

Table S102. Averaged prediction accuracy (R^2) of RF and MIL models for 15 test sets of the PQC data set energy prediction using Pmapper descriptors. 131

Table S103. Averaged prediction accuracy (MAE) of RF and MIL models for 15 test sets of the PQC data set energy prediction using Pmapper descriptors. 132

Table S104. Averaged prediction accuracy (RMSE) of RF and MIL models for 15 test sets of the PQC data set energy prediction using Pmapper descriptors. 133

Table S105. Averaged prediction accuracy (R^2) of RF and MIL models for 15 test sets of the PQC data set energy prediction using 3D-MoRSE descriptors. 134

Table S106. Averaged prediction accuracy (MAE) of RF and MIL models for 15 test sets of the PQC data set energy prediction using 3D-MoRSE descriptors. 135

Table S107. Averaged prediction accuracy (RMSE) of RF and MIL models for 15 test sets of the PQC data set energy prediction using 3D-MoRSE descriptors. 136

Table S108. Averaged prediction accuracy (R^2) of RF and MIL models for 15 test sets of the PQC data set energy prediction using MBTR descriptors. 137

Table S109. Averaged prediction accuracy (MAE) of RF and MIL models for 15 test sets of the PQC data set energy prediction using MBTR descriptors. 138

Table S110. Averaged prediction accuracy (RMSE) of RF and MIL models for 15 test sets of the PQC data set energy prediction using MBTR descriptors. 139

Table S111. Averaged prediction accuracy (R^2) of RF and MIL models for 15 test sets of the PQC data set enthalpy prediction using MOE descriptors. 140

Table S112. Averaged prediction accuracy (MAE) of RF and MIL models for 15 test sets of the PQC data set enthalpy prediction using MOE descriptors. 141

Table S113. Averaged prediction accuracy (RMSE) of RF and MIL models for 15 test sets of the PQC data set enthalpy prediction using MOE descriptors. 142

Table S114. Averaged prediction accuracy (R^2) of RF and MIL models for 15 test sets of the PQC data set enthalpy prediction using Pmapper descriptors. 143

Table S115. Averaged prediction accuracy (MAE) of RF and MIL models for 15 test sets of the PQC data set enthalpy prediction using Pmapper descriptors. 144

Table S116. Averaged prediction accuracy (RMSE) of RF and MIL models for 15 test sets of the

PQC data set enthalpy prediction using Pmapper descriptors. 145

Table S117. Averaged prediction accuracy (R^2) of RF and MIL models for 15 test sets of the PQC data set enthalpy prediction using 3D-MoRSE descriptors. 146

Table S118. Averaged prediction accuracy (MAE) of RF and MIL models for 15 test sets of the PQC data set enthalpy prediction using 3D-MoRSE descriptors. 147

Table S119. Averaged prediction accuracy (RMSE) of RF and MIL models for 15 test sets of the PQC data set enthalpy prediction using 3D-MoRSE descriptors. 148

Table S120. Averaged prediction accuracy (R^2) of RF and MIL models for 15 test sets of the PQC data set enthalpy prediction using MBTR descriptors. 149

Table S121. Averaged prediction accuracy (MAE) of RF and MIL models for 15 test sets of the PQC data set enthalpy prediction using MBTR descriptors. 150

Table S122. Averaged prediction accuracy (RMSE) of RF and MIL models for 15 test sets of the PQC data set enthalpy prediction using MBTR descriptors. 151

Table S123. Averaged prediction accuracy (R^2) of RF and MIL models for 15 test sets of the PQC data set six property predictions using ECFP4 count. 152

Table S124. Averaged prediction accuracy (MAE) of RF and MIL models for 15 test sets of the PQC data set six property predictions using ECFP4 count. 153

Table S125. Averaged prediction accuracy (RMSE) of RF and MIL models for 15 test sets of the PQC data set six property predictions using ECFP4 count. 154

Table S126. Averaged prediction accuracy (MAE) of MolCLR, GEM, and Uni-Mol models for 15 test sets of the PQC data set six property predictions. 155

Table S127. Averaged prediction accuracy (RMSE) of MolCLR, GEM, and Uni-Mol models for 15 test sets of the PQC data set six property predictions. 156

Table S128. Averaged prediction accuracy (R^2) of RF and MIL models for 25 test sets of the MP data set property (melting point) prediction using MOE descriptors. 157

Table S129. Averaged prediction accuracy (MAE) of RF and MIL models for 25 test sets of the MP data set property (melting point) prediction using MOE descriptors. 158

Table S130. Averaged prediction accuracy (RMSE) of RF and MIL models for 25 test sets of the MP data set property (melting point) prediction using MOE descriptors. 159

Table S131. Averaged prediction accuracy (R^2) of RF and MIL models for 25 test sets of the MP data set property (melting point) prediction using Pmapper descriptors. 160

Table S132. Averaged prediction accuracy (MAE) of RF and MIL models for 25 test sets of the MP data set property (melting point) prediction using Pmapper descriptors. 161

Table S133. Averaged prediction accuracy (RMSE) of RF and MIL models for 25 test sets of the MP data set property (melting point) prediction using Pmapper descriptors. 162

Table S134. Averaged prediction accuracy (R^2) of RF and MIL models for 25 test sets of the MP data set property (melting point) prediction using 3D-MoRSE descriptors. 163

Table S135. Averaged prediction accuracy (MAE) of RF and MIL models for 25 test sets of the MP data set property (melting point) prediction using 3D-MoRSE descriptors. 164

Table S136. Averaged prediction accuracy (RMSE) of RF and MIL models for 25 test sets of the MP data set property (melting point) prediction using 3D-MoRSE descriptors. 165

Table S137. Averaged prediction accuracy (R^2) of RF and MIL models for 25 test sets of the MP data set property (melting point) prediction using MBTR descriptors. 166

Table S138. Averaged prediction accuracy (MAE) of RF and MIL models for 25 test sets of the MP data set property (melting point) prediction using MBTR descriptors. 167

Table S139. Averaged prediction accuracy (RMSE) of RF and MIL models for 25 test sets of the MP data set property (melting point) prediction using MBTR descriptors. 168

Table S140. Averaged prediction accuracy (R^2, MAE, RMSE) of RF and MIL models for 25 test sets of the MP data set property (melting point) prediction using ECFP4 count. 169

Table S141. Averaged prediction accuracy (R^2, MAE, RMSE) of MolCLR, GEM, and Uni-Mol models for 25 test sets of the MP data set property (melting point) prediction............................... 170

Table S142. Averaged prediction accuracy (R^2) of RF, Elastic Net, PLS, SVM, and MIL models for 25 test sets of the APTC-1 data set property ($\Delta\Delta G^{\ddagger}$) prediction using MOE descriptors. 171

Table S143. Averaged prediction accuracy (MAE) of RF, Elastic Net, PLS, SVM, and MIL models for 25 test sets of the APTC-1 data set property ($\Delta\Delta G^{\ddagger}$) prediction using MOE descriptors........... 172

Table S144.Averaged prediction accuracy (RMSE) of RF, Elastic Net, PLS, SVM, and MIL models for 25 test sets of the APTC-1 data set property ($\Delta\Delta G^{\ddagger}$) prediction using MOE descriptors........... 173

Table S145.Averaged prediction accuracy (R^2) of RF, Elastic Net, PLS, SVM, and MIL models for

25 test sets of the APTC-1 data set property ($\Delta\Delta G^{\ddagger}$) prediction using Pmapper descriptors.......... 174

Table S146. Averaged prediction accuracy (MAE) of RF, Elastic Net, PLS, SVM, and MIL models for 25 test sets of the APTC-1 data set property ($\Delta\Delta G^{\ddagger}$) prediction using Pmapper descriptors. 175

Table S147. Averaged prediction accuracy (RMSE) of RF, Elastic Net, PLS, SVM, and MIL models for 25 test sets of the APTC-1 data set property ($\Delta\Delta G^{\ddagger}$) prediction using Pmapper descriptors. 176

Table S148. Averaged prediction accuracy (R^2) of RF, Elastic Net, PLS, SVM, and MIL models for 25 test sets of the APTC-1 data set property ($\Delta\Delta G^{\ddagger}$) prediction using 3D-MoRSE descriptors...... 177

Table S149. Averaged prediction accuracy (MAE) of RF, Elastic Net, PLS, SVM, and MIL models for 25 test sets of the APTC-1 data set property ($\Delta\Delta G^{\ddagger}$) prediction using 3D-MoRSE descriptors. 178

Table S150. Averaged prediction accuracy (RMSE) of RF, Elastic Net, PLS, SVM, and MIL models for 25 test sets of the APTC-1 data set property ($\Delta\Delta G^{\ddagger}$) prediction using 3D-MoRSE descriptors. 179

Table S151. Averaged prediction accuracy (R^2) of RF, Elastic Net, PLS, SVM, and MIL models for 25 test sets of the APTC-1 data set property ($\Delta\Delta G^{\ddagger}$) prediction using MBTR descriptors............. 180

Table S152. Averaged prediction accuracy (MAE) of RF, Elastic Net, PLS, SVM, and MIL models for 25 test sets of the APTC-1 data set property ($\Delta\Delta G^{\ddagger}$) prediction using MBTR descriptors........ 181

Table S153. Averaged prediction accuracy (RMSE) of RF, Elastic Net, PLS, SVM, and MIL models for 25 test sets of the APTC-1 data set property ($\Delta\Delta G^{\ddagger}$) prediction using MBTR descriptors........ 182

Table S154. Averaged prediction accuracy (R^2) of RF, Elastic Net, PLS, SVM, and MIL models for 25 test sets of the APTC-1 data set property ($\Delta\Delta G^{\ddagger}$) prediction using ECFP4 bit, ECFP4 count, and 2D PFP. 183

Table S155. Averaged prediction accuracy (MAE) of RF, Elastic Net, PLS, SVM, and MIL models for 25 test sets of the APTC-1 data set property ($\Delta\Delta G^{\ddagger}$) prediction using ECFP4 bit, ECFP4 count, and 2D PFP. 184

Table S156. Averaged prediction accuracy (RMSE) of RF, Elastic Net, PLS, SVM, and MIL models for 25 test sets of the APTC-1 data set property ($\Delta\Delta G^{\ddagger}$) prediction using ECFP4 bit, ECFP4 count, and 2D PFP. 185

Table S157. Averaged prediction accuracy (R^2, MAE, RMSE) of MolCLR, GEM, and Uni-Mol models for 25 test sets of the APTC-1 data set property ($\Delta\Delta G^{\ddagger}$) prediction. 186

Table S158. Averaged prediction accuracy (R^2) of RF, Elastic Net, PLS, SVM, and MIL models for 40 test points of the APTC-2 data set property ($\Delta\Delta G^{\ddagger}$) prediction using MOE descriptors. 187

Table S159. Averaged prediction accuracy (MAE) of RF, Elastic Net, PLS, SVM, and MIL models for 40 test points of the APTC-2 data set property ($\Delta\Delta G^{‡}$) prediction using MOE descriptors....... 188

Table S160. Averaged prediction accuracy (RMSE) of RF, Elastic Net, PLS, SVM, and MIL models for 40 test points of the APTC-2 data set property ($\Delta\Delta G^{‡}$) prediction using MOE descriptors....... 189

Table S161. Averaged prediction accuracy (R^2) of RF, Elastic Net, PLS, SVM, and MIL models for 40 test points of the APTC-2 data set property ($\Delta\Delta G^{‡}$) prediction using Pmapper descriptors. 190

Table S162. Averaged prediction accuracy (MAE) of RF, Elastic Net, PLS, SVM, and MIL models for 40 test points of the APTC-2 data set property ($\Delta\Delta G^{‡}$) prediction using Pmapper descriptors. 191

Table S163. Averaged prediction accuracy (RMSE) of RF, Elastic Net, PLS, SVM, and MIL models for 40 test points of the APTC-2 data set property ($\Delta\Delta G^{‡}$) prediction using Pmapper descriptors. 192

Table S164. Averaged prediction accuracy (R^2) of RF, Elastic Net, PLS, SVM, and MIL models for 40 test points of the APTC-2 data set property ($\Delta\Delta G^{‡}$) prediction using 3D-MoRSE descriptors. . 193

Table S165. Averaged prediction accuracy (MAE) of RF, Elastic Net, PLS, SVM, and MIL models for 40 test points of the APTC-2 data set property ($\Delta\Delta G^{‡}$) prediction using 3D-MoRSE descriptors. 194

Table S166. Averaged prediction accuracy (RMSE) of RF, Elastic Net, PLS, SVM, and MIL models for 40 test points of the APTC-2 data set property ($\Delta\Delta G^{‡}$) prediction using 3D-MoRSE descriptors. 195

Table S167. Averaged prediction accuracy (R^2) of RF, Elastic Net, PLS, SVM, and MIL models for 40 test points of the APTC-2 data set property ($\Delta\Delta G^{‡}$) prediction using MBTR descriptors.......... 196

Table S168. Averaged prediction accuracy (MAE) of RF, Elastic Net, PLS, SVM, and MIL models for 40 test points of the APTC-2 data set property ($\Delta\Delta G^{‡}$) prediction using MBTR descriptors. ... 197

Table S169. Averaged prediction accuracy (RMSE) of RF, Elastic Net, PLS, SVM, and MIL models for 40 test points of the APTC-2 data set property ($\Delta\Delta G^{‡}$) prediction using MBTR descriptors. ... 198

Table S170. Averaged prediction accuracy (R^2) of RF, Elastic Net, PLS, SVM, and MIL models for 40 test points of the APTC-2 data set property ($\Delta\Delta G^{‡}$) prediction using ECFP4 bit, ECFP4 count, and 2D PFP. 199

Table S171. Averaged prediction accuracy (MAE) of RF, Elastic Net, PLS, SVM, and MIL models for 40 test points of the APTC-2 data set property ($\Delta\Delta G^{‡}$) prediction using ECFP4 bit, ECFP4 count, and 2D PFP. 200

Table S172. Averaged prediction accuracy (RMSE) of RF, Elastic Net, PLS, SVM, and MIL models for 40 test points of the APTC-2 data set property ($\Delta\Delta G^{\ddagger}$) prediction using ECFP4 bit, ECFP4 count, and 2D PFP. 201

Table S173. Averaged prediction accuracy (R^2, MAE, RMSE) of MolCLR, GEM, and Uni-Mol models for 40 test points of the APTC-2 data set property ($\Delta\Delta G^{\ddagger}$) prediction. 202

Table S174. Average prediction accuracy (MAE) of random forest (RF) models for 25 test sets of the MP data sets property (melting point) prediction. 203

Table S175. Comparison of average prediction accuracy (MAE) for 25 test sets of the MP data sets property (melting point) prediction among ML models. 204

Table S176. Averaged prediction accuracy (MAE) of random forest (RF) models for 25 test sets for the APTC-1 & 40 test points for the APTC-2 data sets property ($\Delta\Delta G^{\ddagger}$) prediction. 205

Table S177. Comparison of averaged prediction accuracy (MAE) for 25 test sets for the APTC-1 & 40 test points for the APTC-2 data sets property ($\Delta\Delta G^{\ddagger}$) prediction among ML models. 206

Table S178. Order of models in Figure S36 to S70. 235

Table S1. Description of MOE 3D descriptors. In the analysis using the MP, APTC-1, and APTC-2 data sets, the following 117 types of descriptors were used, while in the analysis using the PQC data set, 116 types of descriptors were used after excluding 'dipole'. All 3D descriptors are internal coordinate dependent.

**Potential Energy Descriptors**	
**Name**	**Description**
E	Value of the potential energy.
E_ang	Angle bend potential energy.
E_ele	Electrostatic component of the potential energy.
E_nb	Value of the potential energy with all bonded terms disabled.
E_oop	Out-of-plane potential energy.
E_sol	Solvation energy.
E_stb	Bond stretch-bend cross-term potential energy.
E_str	Bond stretch potential energy.
E_strain	Local strain energy: the current energy minus the value of the energy at a near local minimum.
E_tor	Torsion (proper and improper) potential energy.
E_vdw	van der Waals component of the potential energy.
**Conformation Dependent Charge Descriptors**	
**Name**	**Description**
ASA+	Water accessible surface area of all atoms with positive partial charge.
ASA-	Water accessible surface area of all atoms with negative partial charge.
ASA_H	Water accessible surface area of all hydrophobic atoms.
ASA_P	Water accessible surface area of all polar atoms.
DASA	Absolute value of the difference between ASA+ and ASA-.
CASA+	Positive charge weighted surface area.
CASA-	Negative charge weighted surface area.
DCASA	Absolute value of the difference between CASA+ and CASA-.
dipole	Dipole moment calculated from the partial charges of the molecule.
FASA+	Fractional ASA+ calculated as ASA+ / ASA.
FASA-	Fractional ASA- calculated as ASA- / ASA.

FCASA+	Fractional CASA+ calculated as CASA+ / ASA.
FCASA-	Fractional CASA- calculated as CASA- / ASA.
FASA_H	Fractional ASA_H calculated as ASA_H / ASA.
FASA_P	Fractional ASA_P calculated as ASA_P / ASA.
**Surface Area, Volume and Shape Descriptors**	
**Name**	**Description**
ASA	Water accessible surface area calculated using a radius of 1.4 A for the water molecule.
dens	Mass density: molecular weight divided by van der Waals volume as calculated in the vol descriptor.
glob	Globularity, or inverse condition number of the covariance matrix of atomic coordinates.
pmi	Principal moment of inertia.
pmi1	First diagonal element of diagonalized moment of inertia tensor.
pmi2	Second diagonal element of diagonalized moment of inertia tensor.
pmi3	Third diagonal element of diagonalized moment of inertia tensor.
npr1	Normalized PMI ratio pmi1/pmi3.
npr2	Normalized PMI ratio pmi2/pmi3.
rgyr	Radius of gyration.
std_dim1	Standard dimension 1.
std_dim2	Standard dimension 2.
std_dim3	Standard dimension 3.
vol	van der Waals volume calculated using a grid approximation.
VSA	van der Waals surface area.
vsurf_V	Interaction field volume.
vsurf_S	Interaction field surface area.
vsurf_R	Surface rugosity.
vsurf_G	Surface globularity.
vsurf_W*	Hydrophilic volume (8 descriptors).
vsurf_IW*	Hydrophilic integy moment (8 descriptors).
vsurf_CW*	Capacity factor (8 descriptors).
vsurf_EWmin*	Lowest hydrophilic energy (3 descriptors).

vsurf_DW*	Contact distances of vsurf_EWmin (3 descriptors).
vsurf_D*	Hydrophobic volume (8 descriptors).
vsurf_ID*	Hydrophobic integy moment (8 descriptors).
vsurf_EDmin*	Lowest hydrophobic energy (3 descriptors).
vsurf_DD*	Contact distances of vsurf_DDmin (3 descriptors).
vsurf_HL*	Hydrophilic-Lipophilic (2 descriptors).
vsurf_A	Amphiphilic moment.
vsurf_CP	Critical packing parameter.
vsurf_Wp*	Polar volume (8 descriptors).
vsurf_HB*	H-bond donor capacity (8 descriptors).

Table S2. Specification of vertices in Pmapper descriptors. In all analyses using Pmapper descriptors, any atoms except for five- and six-membered ones and the centers of five- or six-membered rings were specified as vertices of 3D pharmacophore triplets. 1,028 triplets, 864 triplets, 575 triplets, 1,202 triplets, and 1,556 triplets are generated to generated conformers in the PQC data set, ground-truth conformers in the PQC data set, MP data set, APTC-1 data set, and APTC-2 data set, respectively.

Data set	SMARTS and feature label
PQC	a1aaaaa1 a<br>a1aaaa1 a<br>[C,N,O] H
MP	a1aaaaa1 a<br>a1aaaa1 a<br>[C,N,O,S] H
APTC-1, APTC-2	a1aaaaa1 a<br>a1aaaa1 a<br>[C,N,O,S,P,F,Cl,Br,I] H

Table S3. Description of 3D-MoRSE descriptors. In all analyses using 3D-MoRSE descriptors, the following 160 descriptors are used.

Name	Description
Mor*	Unweighted 3D-MoRSE (distance from 1 to 32).
Mor*m	3D-MoRSE weighted by mass (distance from 1 to 32).
Mor*v	3D-MoRSE weighted by van der Waals volume (distance from 1 to 32).
Mor*se	3D-MoRSE weighted by sanderson EN (distance from 1 to 32).
Mor*p	3D-MoRSE weighted by polarizability (distance from 1 to 32).

Table S4. Parameters of MBTR descriptors. 540, 950, and 2310 descriptors are generated to the PQC, MP, and APTCs data sets, respectively.

Degree	Parameter	Value
k=1	geometry	"function": "atomic_number"}
	grid	"min": 0, "max": 8, "n": 10, "sigma": 0.1
k=2	geometry	"function": "inverse_distance"
	grid	"min": 0, "max": 4, "n": 10, "sigma": 0.1
	weighting	"function": "exponential","scale": 0.5, "cutoff": 1e-3
k=3	geometry	"function": "cosine"
	grid	"min": -1, "max": 4, "n": 10, "sigma": 0.1
	weighting	"function": "exponential","scale": 0.5, "cutoff": 1e-3
**Parameter**		**Value**
periodic		False
normalization		"l2_each"
species		PQC data set: ['H', 'C', 'N', 'O']<br>MP data set: ['H', 'C', 'N', 'O', 'S']<br>APTCs data set: ['H', 'C', 'N', 'O', 'F', 'Br', 'I']

Table S5. Custom features and bins of 2D PFP descriptors. Only calculated to APTCs data sets and 2048-dimension vectors are generated to the APTCs data sets.

Name	Value
custom_features	DefineFeature AliphaAtom [C,N,O,S,P,F,Cl,Br,I]<br>Family AliphaAtom<br>Weights 1.0<br>EndFeature<br><br>AtomType AromR5 [a;r5,!R1&r4,!R1&r3]<br>DefineFeature Arom5<br>[{AromR5}]1:[{AromR5}]:[{AromR5}]:[{AromR5}]:[{AromR5}]:1<br>Family Aromatic<br>Weights 1.0,1.0,1.0,1.0,1.0<br>EndFeature<br><br>AtomType AromR6 [a;r6,!R1&r5,!R1&r4,!R1&r3]<br>DefineFeature Arom6<br>[{AromR6}]1:[{AromR6}]:[{AromR6}]:[{AromR6}]:[{AromR6}]:[{AromR6}]:1<br>Family Aromatic<br>Weights 1.0,1.0,1.0,1.0,1.0,1.0<br>EndFeature
bins	[(0, 1), (1, 2), (2, 3), (3, 4), (4, 5), (5, 6), (6, 7), (7, 8)]

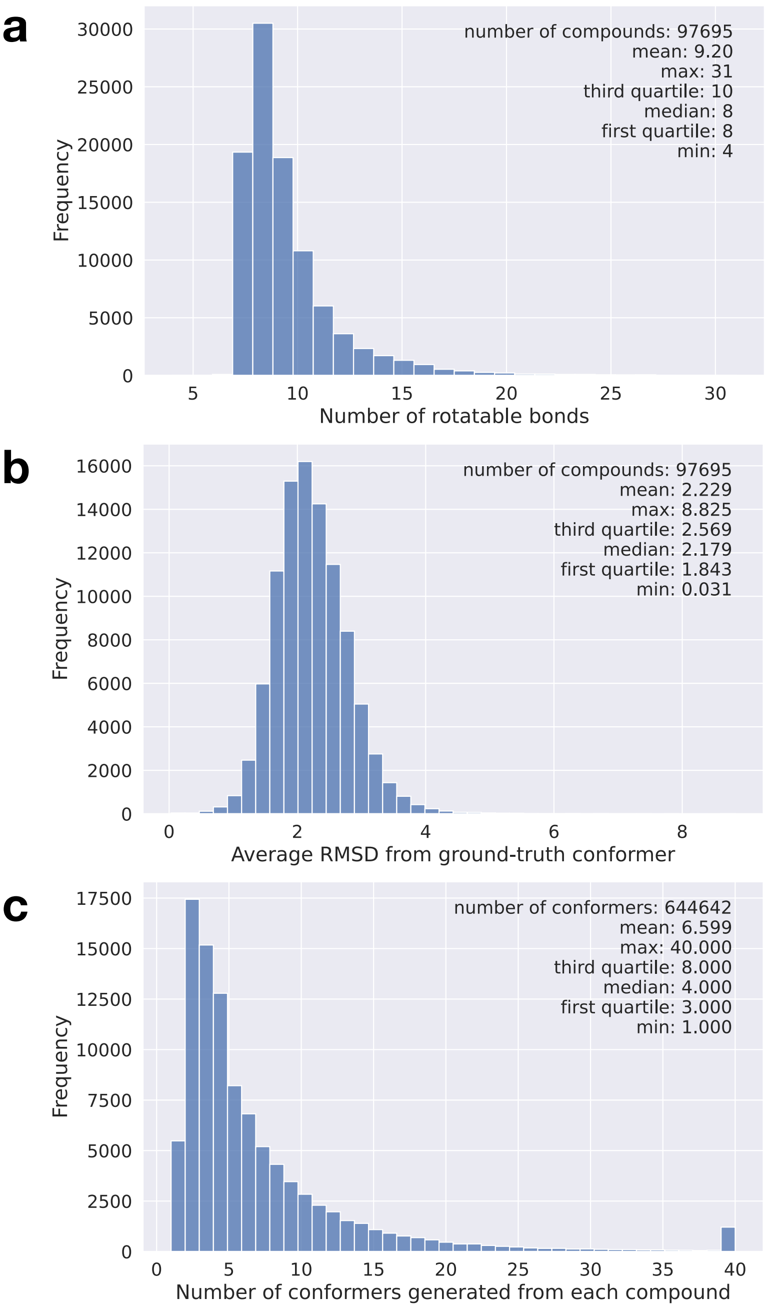

Figure S1. Distributions of the PQC data set and the diversity of the generated conformers. a Histogram of the number of rotatable bonds for the compounds in the data set. **b** Histogram of the average RMSD calculated for each compound between the multiple conformers generated and the correct conformer recorded in the data. Conformers were generated using OMEGA by OpenEye Toolkit, and then optimized using PM6 Hamiltonian in MOPAC. **c** Histogram of the number of conformers generated for each compound. When generating conformers in OMEGA, the RMSD threshold was set to 2 Å and the upper limit of the number of conformers was set to 40.

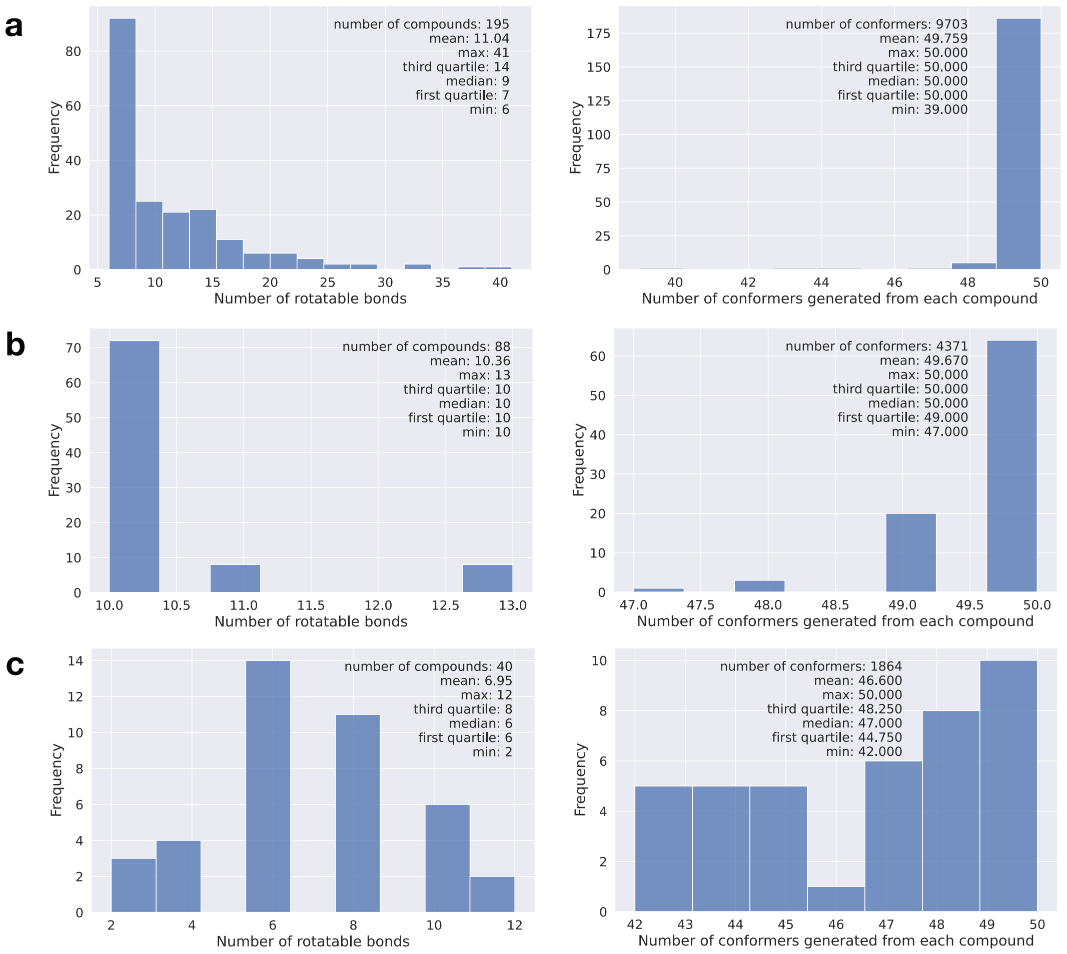

Figure S2. Distributions of the MP and APTCs data sets. a Distributions of the MP data set. **b** Distributions of the APTC-1 data set. **c** Distributions of the APTC-2 data set. (left) Histogram of the number of rotatable bonds for the compounds in the data set. (right) Histogram of the number of conformers generated for each compound.

a

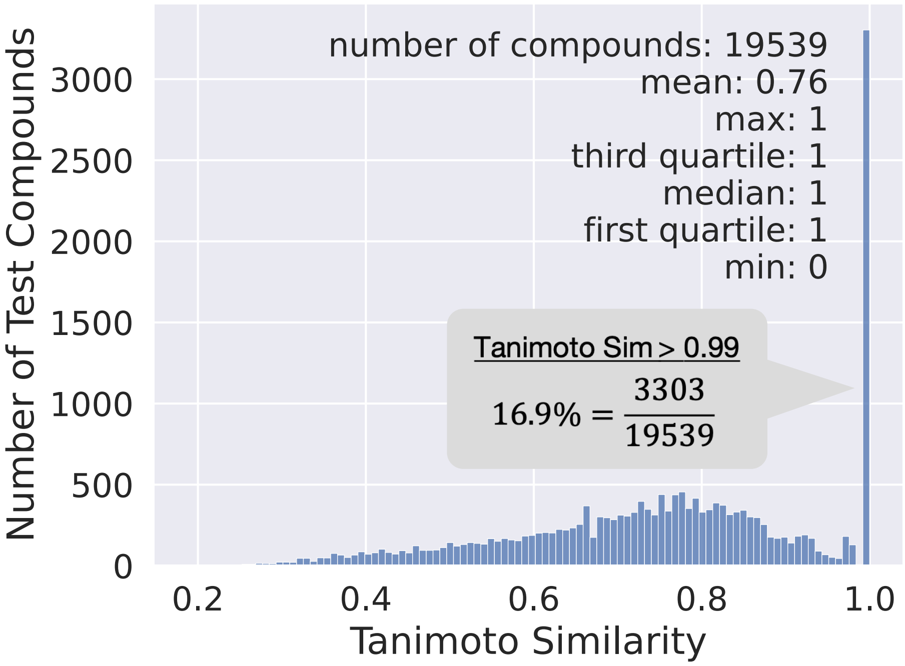

b

	Tanimoto Similarity							
	0.3	0.4	0.5	0.6	0.7	0.8	0.9	1.0
A compound in test data	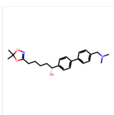	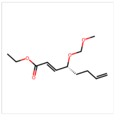	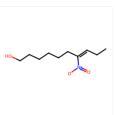	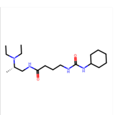	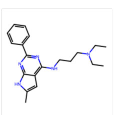	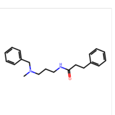	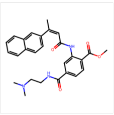	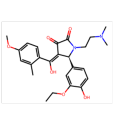
Most similar compound in training data	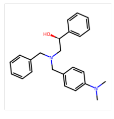	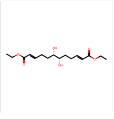	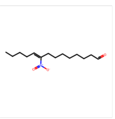	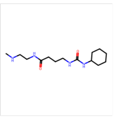	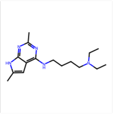	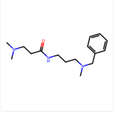	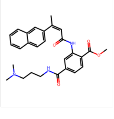	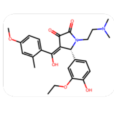

Figure S3. Similarity distribution of compounds between the training and the test data of the PQC data set in Seed 42-Fold 0. a Histogram of Tanimoto Similarity between the compounds in the test data and the compounds in the training data that are structurally most similar. Of the 19,539 compounds in the test data, 3,303 had a Tanimoto Similarity greater than 0.99. Most of these are isomers. **b** An example of a compound pair with a certain Tanimoto Similarity value. A compound in the test data and its most similar compound in the training data are displayed as a pair.

Table S6. Hyperparameters and search space of Random Forest (RF) models, Elastic Net models, Partial Least Squares (PLS) models, Support Vector Machine (SVM) models, Muti Instance Learning (MIL) models, MolCLR models, GEM models, and Uni-Mol models. Only RF hyperparameters in the PQC data set were tuned using Optuna with 30 trials. Hyperparameters of all other models were fixed.

PQC data set		
**Model**	**Hyperparameter**	**Search space (Distribution)**
RF	n_estimators	10 – 500 (Int)
	max_depth	2 – 128 (Int log-scaled)
	max_features	[sqrt, log2, None] (Categorical)
**Model**	**Hyperparameter**	**Value**
MIL	Batch size	512
	Epochs	500
	Learning rate	0.001
	Weight decay	0.0001
	Pooling	mean
MolCLR	Batch size	32
	Epochs	100
	Learning rate for GNN encoder	0.0001
	Learning rate for prediction head	0.0005
	Weight decay	0.000001
GEM	Batch size	32
	Epochs	100
	Learning rate for encoder	0.001
	Learning rate for prediction head	0.001
	Dropout rate	0.2
Uni-Mol	Batch size	128
	Epochs	100
	Learning rate	0.0001

	Warmup ratio	0.03
	Remove hydrogen	True
	Patience	5
**MP, APTC-1, APTC-2 data sets**		
**Model**	**Hyperparameter**	**Value**
RF	n_estimators	500
	max_depth	50
	max_features	None
Elastic Net	alphas	1.0
	l1_ratio	0.5
PLS	n_components	5
SVM	C	1
	epsilon	0.1
	kernel	rbf
MIL	Batch size	128
	Epochs	500
	Learning rate	0.001
	Weight decay	0.0001
	Pooling	mean
MolCLR	Batch size	32
	Epochs	100
	Learning rate for GNN encoder	0.0001
	Learning rate for prediction head	0.0005
	Weight decay	0.000001
GEM	Batch size	32
	Epochs	100
	Learning rate for encoder	0.001
	Learning rate for prediction head	0.001
	Dropout rate	0.2

Uni-Mol	Batch size	8
	Epochs	100
	Learning rate	0.0001
	Warmup ratio	0.03
	Remove hydrogen	True
	Patience	5

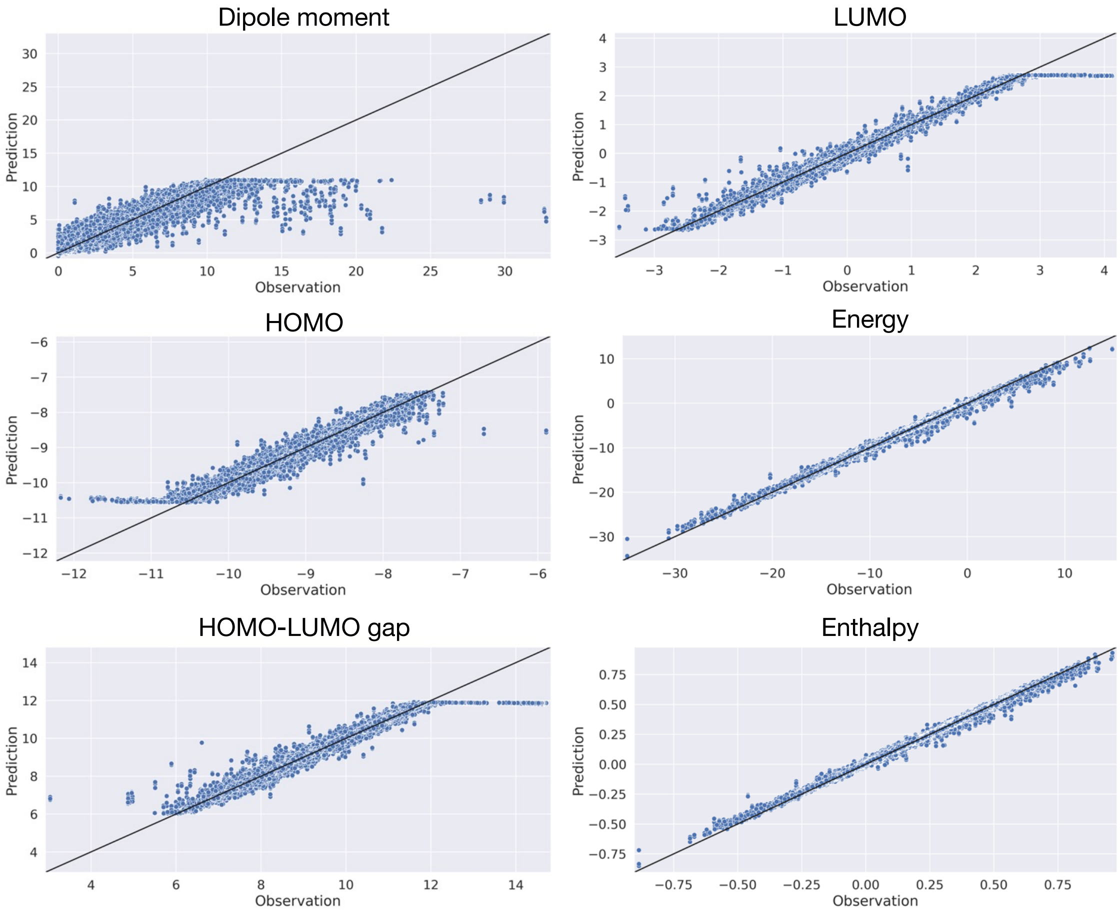

Figure S4. Prediction against experimental value plots for the PQC data set. Uni-Mol with ground-truth conformations is used for predicting dipole moment, HOMO, HOMO-LUMO gap, and LUMO prediction, while GEM with non-aggregation conformations is used for energy and enthalpy prediction.

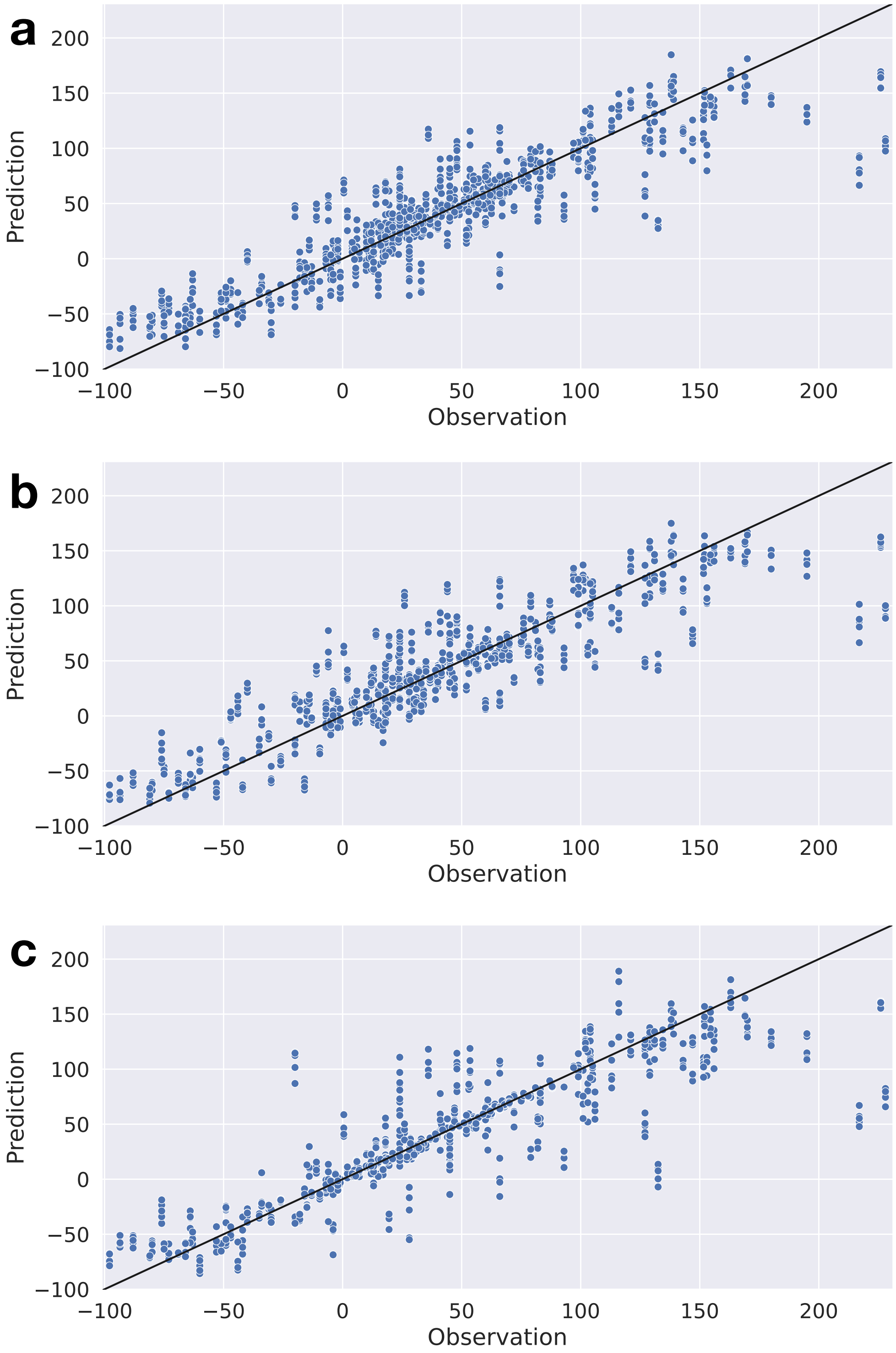

Figure S5. Prediction against experimental value plots for the MP data set, using (a) Uni-Mol with global minimum conformations, (b) RF with MOE global minimum descriptors, and (c) Uni-Mol with non-aggregation conformations.

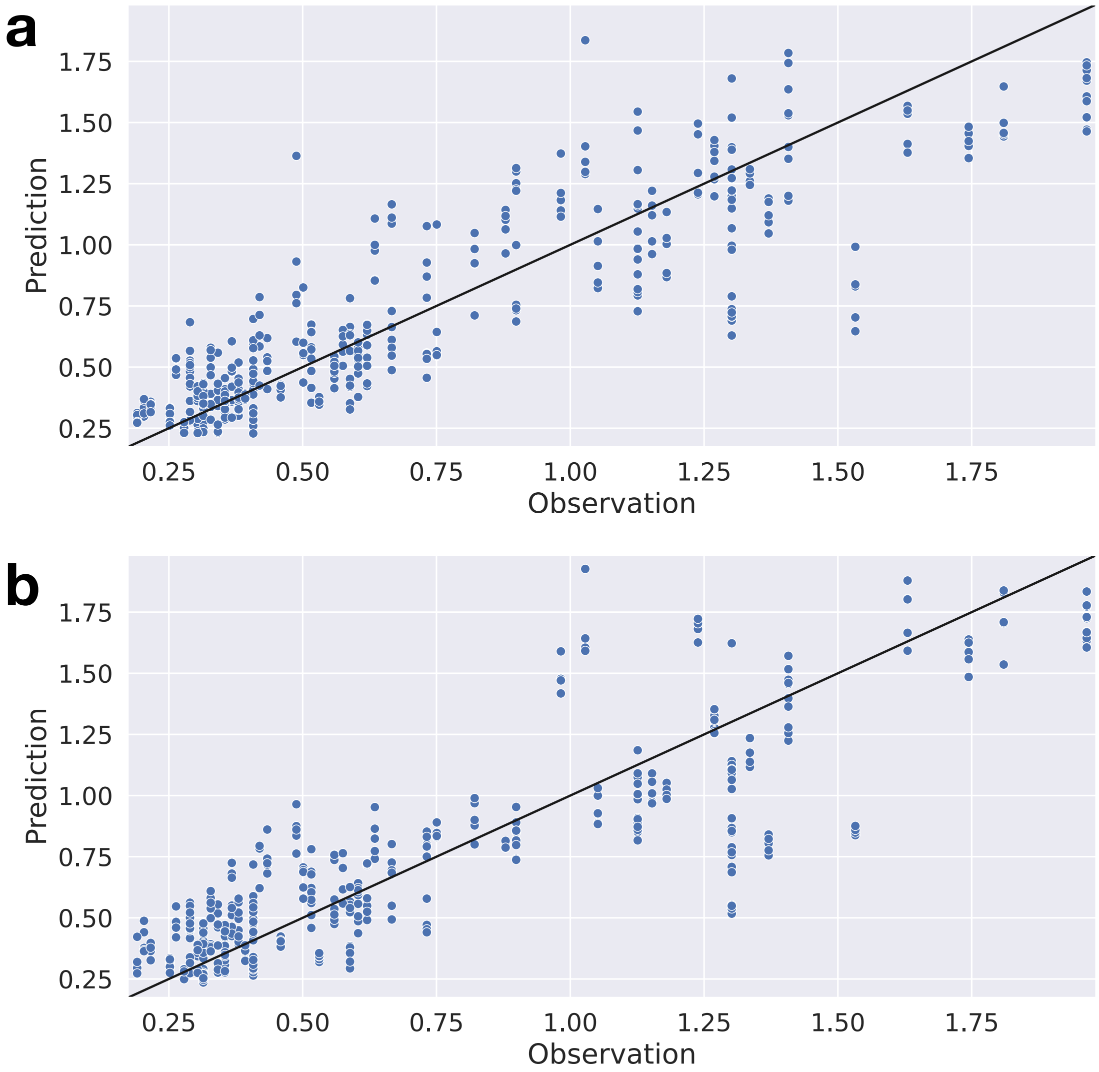

Figure S6. Prediction against experimental value plots for the APTC-1 data set, using (a) Uni-Mol with non-aggregation conformations and (b) MLP with Pmapper non-aggregation descriptors.

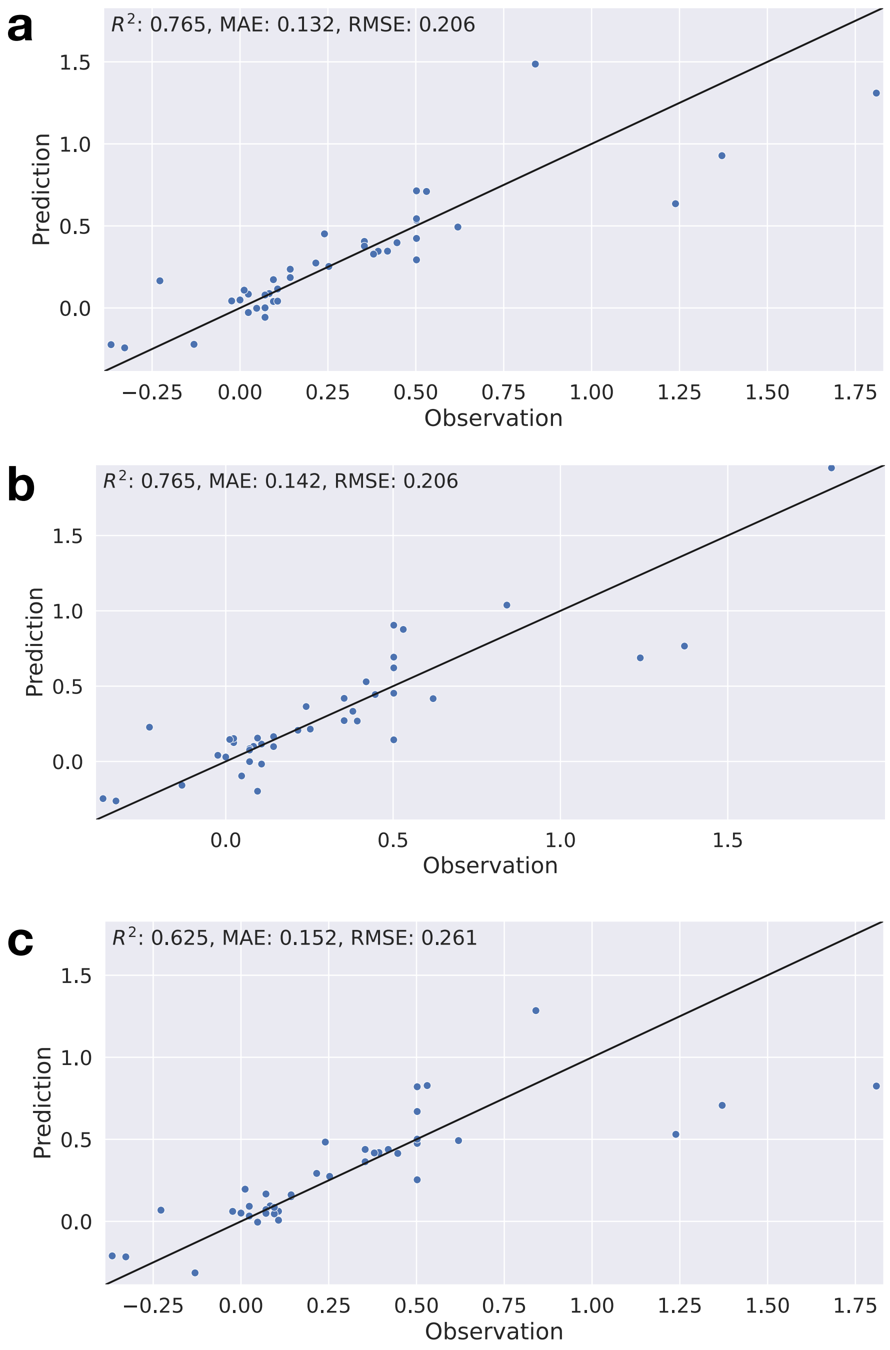

Figure S7. Prediction against experimental value plots for the APTC-2 data set, using (a) MLP with 3D-MoRSE non-aggregation descriptors, (b) MLP with Pmapper non-aggregation descriptors, and (c) Uni-Mol with non-aggregation conformations.

Table S7. Averaged prediction accuracy (R^2) of RF and MIL models for 15 training sets of the PQC data set dipole moment prediction using MOE descriptors. The standard deviations are provided in parentheses.

Data set = PQC (Training sets)<br>Descs = MOE<br>y = Dipole moment (Debye)		RF	MIL				
			Non-aggregation (Instance-Wrapper)	Bag-Wrapper	Instance-Net	Bag-Net	Bag-AttentionNet
		R^2					
Aggregation Method	Boltzmann Weight	0.922 (0.001)	0.591 (0.022)	0.596 (0.019)	0.602 (0.013)	0.598 (0.021)	0.592 (0.008)
	Mean	0.923 (0.003)	0.601 (0.014)	0.597 (0.019)	0.602 (0.012)	0.595 (0.014)	0.595 (0.019)
	Global Minimum	0.920 (0.001)	0.589 (0.014)	0.592 (0.014)	0.593 (0.013)	0.588 (0.017)	0.597 (0.014)
	Random	0.917 (0.007)	0.588 (0.024)	0.593 (0.016)	0.592 (0.012)	0.596 (0.017)	0.601 (0.014)
	RMSD max.	0.919 (0.003)	0.588 (0.017)	0.600 (0.012)	0.589 (0.016)	0.590 (0.018)	0.592 (0.011)
	RMSD min.	0.923 (0.002)	0.611 (0.009)	0.608 (0.014)	0.604 (0.022)	0.611 (0.019)	0.608 (0.011)
Non-Aggregation		0.945 (0.000)	0.658 (0.006)	0.588 (0.015)	0.591 (0.014)	0.589 (0.012)	0.540 (0.007)
Ground-truth		0.931 (0.002)	0.665 (0.022)	0.654 (0.017)	0.657 (0.012)	0.656 (0.015)	0.664 (0.016)

Table S8. Averaged prediction accuracy (R^2) of RF and MIL models for 15 training sets of the PQC data set dipole moment prediction using Pmapper descriptors. The standard deviations are provided in parentheses.

Data set = PQC (Training sets)<br>Descs = Pmapper<br>y = Dipole moment (Debye)		RF	MIL				
			Non-aggregation (Instance-Wrapper)	Bag-Wrapper	Instance-Net	Bag-Net	Bag-AttentionNet
		R^2					
Aggregation Method	Boltzmann Weight	0.905 (0.002)	0.770 (0.006)	0.770 (0.007)	0.770 (0.011)	0.770 (0.004)	0.768 (0.012)
	Mean	0.907 (0.001)	0.782 (0.009)	0.784 (0.005)	0.784 (0.011)	0.786 (0.004)	0.785 (0.005)
	Global Minimum	0.903 (0.002)	0.009 (0.002)	0.009 (0.002)	0.010 (0.001)	0.009 (0.002)	0.009 (0.001)
	Random	0.903 (0.002)	0.007 (0.001)	0.008 (0.001)	0.007 (0.002)	0.008 (0.001)	0.008 (0.001)
	RMSD max.	0.902 (0.003)	0.009 (0.001)	0.009 (0.001)	0.009 (0.001)	0.009 (0.001)	0.009 (0.001)
	RMSD min.	0.902 (0.006)	0.010 (0.001)	0.011 (0.001)	0.010 (0.001)	0.010 (0.001)	0.010 (0.001)
Non-Aggregation		0.923 (0.003)	0.839 (0.005)	0.774 (0.010)	0.765 (0.017)	0.772 (0.011)	0.790 (0.006)
Ground-truth		0.912 (0.000)	0.008 (0.001)	0.008 (0.001)	0.008 (0.001)	0.008 (0.001)	0.007 (0.002)

Table S9. Averaged prediction accuracy (R^2) of RF and MIL models for 15 training sets of the PQC data set dipole moment prediction using 3D-MoRSE descriptors. The standard deviations are provided in parentheses.

Data set = PQC (Training sets)<br>Descs = 3D-MoRSE<br>y = Dipole moment (Debye)		RF	MIL				
			Non-aggregation (Instance-Wrapper)	Bag-Wrapper	Instance-Net	Bag-Net	Bag-AttentionNet
		R^2					
Aggregation Method	Boltzmann Weight	0.908 (0.001)	0.449 (0.015)	0.463 (0.009)	0.449 (0.018)	0.456 (0.013)	0.458 (0.020)
	Mean	0.912 (0.001)	0.457 (0.024)	0.450 (0.025)	0.463 (0.019)	0.450 (0.022)	0.451 (0.024)
	Global Minimum	0.904 (0.005)	0.449 (0.019)	0.440 (0.018)	0.437 (0.023)	0.439 (0.021)	0.451 (0.018)
	Random	0.905 (0.002)	0.440 (0.021)	0.445 (0.017)	0.447 (0.015)	0.442 (0.023)	0.440 (0.016)
	RMSD max.	0.905 (0.001)	0.434 (0.038)	0.454 (0.017)	0.450 (0.019)	0.450 (0.022)	0.435 (0.031)
	RMSD min.	0.906 (0.000)	0.448 (0.016)	0.452 (0.016)	0.455 (0.019)	0.442 (0.016)	0.453 (0.015)
Non-Aggregation		0.921 (0.001)	0.580 (0.010)	0.449 (0.016)	0.453 (0.022)	0.448 (0.022)	0.441 (0.018)
Ground-truth		0.908 (0.003)	0.493 (0.025)	0.494 (0.018)	0.495 (0.019)	0.488 (0.021)	0.486 (0.015)

Table S10. Averaged prediction accuracy (R^2) of RF and MIL models for 15 training sets of the PQC data set dipole moment prediction using MBTR descriptors. The standard deviations are provided in parentheses.

Data set = PQC (Training sets) Descs = MBTR y = Dipole moment (Debye)		RF	MIL				
			Non-aggregation (Instance-Wrapper)	Bag-Wrapper	Instance-Net	Bag-Net	Bag-AttentionNet
		R^2					
Aggregation Method	Boltzmann Weight	0.928 (0.001)	0.645 (0.012)	0.641 (0.013)	0.638 (0.014)	0.645 (0.015)	0.636 (0.035)
	Mean	0.929 (0.000)	0.629 (0.026)	0.628 (0.027)	0.634 (0.011)	0.633 (0.012)	0.634 (0.012)
	Global Minimum	0.927 (0.001)	0.646 (0.022)	0.653 (0.011)	0.646 (0.021)	0.649 (0.017)	0.643 (0.025)
	Random	0.927 (0.001)	0.646 (0.016)	0.650 (0.008)	0.651 (0.012)	0.641 (0.021)	0.635 (0.022)
	RMSD max.	0.928 (0.001)	0.648 (0.018)	0.653 (0.011)	0.642 (0.014)	0.645 (0.060)	0.655 (0.009)
	RMSD min.	0.929 (0.001)	0.658 (0.013)	0.659 (0.018)	0.657 (0.021)	0.660 (0.016)	0.657 (0.016)
Non-Aggregation		0.963 (0.001)	0.755 (0.008)	0.626 (0.023)	0.634 (0.021)	0.638 (0.012)	0.632 (0.010)
Ground-truth		0.937 (0.002)	0.709 (0.017)	0.708 (0.017)	0.710 (0.018)	0.702 (0.025)	0.699 (0.029)

Table S11. Averaged prediction accuracy (R^2) of RF and MIL models for 15 training sets of the PQC data set HOMO prediction using MOE descriptors. The standard deviation is provided in parentheses.

Data set = PQC (Training sets)<br>Descs = MOE<br>y = HOMO (eV)		RF	MIL				
			Non-aggregation (Instance-Wrapper)	Bag-Wrapper	Instance-Net	Bag-Net	Bag-AttentionNet
		R^2					
Aggregation Method	Boltzmann Weight	0.957 (0.015)	0.695 (0.019)	0.694 (0.017)	0.689 (0.024)	0.665 (0.045)	0.687 (0.019)
	Mean	0.965 (0.000)	0.713 (0.023)	0.722 (0.018)	0.714 (0.023)	0.712 (0.018)	0.702 (0.049)
	Global Minimum	0.959 (0.001)	0.684 (0.010)	0.683 (0.013)	0.659 (0.033)	0.661 (0.060)	0.681 (0.015)
	Random	0.960 (0.000)	0.681 (0.018)	0.686 (0.011)	0.672 (0.031)	0.677 (0.029)	0.674 (0.032)
	RMSD max.	0.958 (0.000)	0.649 (0.031)	0.671 (0.011)	0.664 (0.025)	0.665 (0.018)	0.668 (0.025)
	RMSD min.	0.961 (0.000)	0.695 (0.014)	0.681 (0.030)	0.669 (0.042)	0.686 (0.027)	0.697 (0.017)
Non-Aggregation		0.976 (0.004)	0.761 (0.014)	0.684 (0.058)	0.704 (0.047)	0.702 (0.052)	0.708 (0.029)
Ground-truth		0.955 (0.000)	0.640 (0.044)	0.657 (0.029)	0.662 (0.019)	0.635 (0.070)	0.656 (0.024)

Table S12. Averaged prediction accuracy (R^2) of RF and MIL models for 15 training sets of the PQC data set HOMO prediction using Pmapper descriptors. The standard deviations are provided in parentheses.

Data set = PQC (Training sets)<br>Descs = Pmapper<br>y = HOMO (eV)		RF	MIL				
			Non-aggregation (Instance-Wrapper)	Bag-Wrapper	Instance-Net	Bag-Net	Bag-AttentionNet
		R^2					
Aggregation Method	Boltzmann Weight	0.935 (0.001)	0.782 (0.026)	0.784 (0.033)	0.766 (0.059)	0.786 (0.021)	0.785 (0.014)
	Mean	0.940 (0.001)	0.805 (0.026)	0.806 (0.020)	0.804 (0.029)	0.811 (0.012)	0.791 (0.031)
	Global Minimum	0.933 (0.000)	0.006 (0.004)	0.008 (0.003)	0.009 (0.001)	0.007 (0.002)	0.008 (0.002)
	Random	0.932 (0.001)	0.008 (0.003)	0.008 (0.004)	0.008 (0.002)	0.008 (0.003)	0.009 (0.002)
	RMSD max.	0.927 (0.007)	0.009 (0.001)	0.009 (0.001)	0.008 (0.002)	0.008 (0.001)	0.008 (0.003)
	RMSD min.	0.934 (0.001)	0.009 (0.001)	0.009 (0.001)	0.008 (0.002)	0.008 (0.003)	0.008 (0.003)
Non-Aggregation		0.963 (0.013)	0.809 (0.021)	0.785 (0.028)	0.803 (0.019)	0.791 (0.031)	0.761 (0.022)
Ground-truth		0.936 (0.000)	0.006 (0.003)	0.007 (0.001)	0.005 (0.003)	0.007 (0.001)	0.006 (0.003)

Table S13. Averaged prediction accuracy (R^2) of RF and MIL models for 15 training sets of the PQC data set HOMO prediction using 3D-MoRSE descriptors. The standard deviations are provided in parentheses.

Data set = PQC (Training sets)<br>Descs = 3D-MoRSE<br>y = HOMO (eV)		RF	MIL				
			Non-aggregation (Instance-Wrapper)	Bag-Wrapper	Instance-Net	Bag-Net	Bag-AttentionNet
		R^2					
Aggregation Method	Boltzmann Weight	0.952 (0.000)	0.715 (0.027)	0.713 (0.025)	0.713 (0.028)	0.705 (0.043)	0.699 (0.048)
	Mean	0.958 (0.000)	0.724 (0.046)	0.732 (0.024)	0.751 (0.007)	0.734 (0.025)	0.723 (0.037)
	Global Minimum	0.947 (0.001)	0.690 (0.039)	0.701 (0.019)	0.700 (0.025)	0.696 (0.035)	0.709 (0.019)
	Random	0.949 (0.000)	0.711 (0.019)	0.680 (0.063)	0.700 (0.048)	0.708 (0.021)	0.711 (0.020)
	RMSD max.	0.947 (0.000)	0.697 (0.037)	0.703 (0.029)	0.707 (0.012)	0.703 (0.015)	0.698 (0.023)
	RMSD min.	0.950 (0.001)	0.710 (0.017)	0.699 (0.049)	0.705 (0.023)	0.698 (0.026)	0.699 (0.044)
Non-Aggregation		0.959 (0.004)	0.776 (0.027)	0.738 (0.013)	0.733 (0.022)	0.727 (0.039)	0.720 (0.050)
Ground-truth		0.950 (0.000)	0.709 (0.027)	0.723 (0.020)	0.725 (0.009)	0.704 (0.030)	0.712 (0.027)

Table S14. Averaged prediction accuracy (R^2) of RF and MIL models for 15 training sets of the PQC data set HOMO prediction using MBTR descriptors. The standard deviations are provided in parentheses.

Data set = PQC (Training sets)<br>Descs = MBTR<br>y = HOMO (eV)		RF	MIL				
			Non-aggregation (Instance-Wrapper)	Bag-Wrapper	Instance-Net	Bag-Net	Bag-AttentionNet
		R^2					
Aggregation Method	Boltzmann Weight	0.973 (0.000)	0.828 (0.012)	0.825 (0.015)	0.829 (0.018)	0.819 (0.040)	0.835 (0.013)
	Mean	0.974 (0.000)	0.843 (0.011)	0.829 (0.030)	0.831 (0.037)	0.842 (0.009)	0.839 (0.014)
	Global Minimum	0.972 (0.001)	0.824 (0.018)	0.828 (0.014)	0.830 (0.012)	0.830 (0.012)	0.830 (0.015)
	Random	0.972 (0.000)	0.834 (0.012)	0.828 (0.018)	0.822 (0.019)	0.827 (0.015)	0.821 (0.021)
	RMSD max.	0.971 (0.000)	0.820 (0.016)	0.813 (0.028)	0.819 (0.022)	0.827 (0.011)	0.825 (0.015)
	RMSD min.	0.972 (0.000)	0.813 (0.039)	0.839 (0.006)	0.829 (0.019)	0.835 (0.015)	0.828 (0.026)
Non-Aggregation		0.988 (0.001)	0.870 (0.013)	0.835 (0.013)	0.838 (0.017)	0.835 (0.017)	0.835 (0.026)
Ground-truth		0.974 (0.001)	0.842 (0.029)	0.842 (0.044)	0.851 (0.018)	0.832 (0.022)	0.838 (0.032)

Table S15. Averaged prediction accuracy (R^2) of RF and MIL models for 15 training sets of the PQC data set HOMO-LUMO gap prediction using MOE descriptors. The standard deviations are provided in parentheses.

Data set = PQC (Training sets)<br>Descs = MOE<br>y = HOMO-LUMO gap (eV)		RF	MIL				
			Non-aggregation (Instance-Wrapper)	Bag-Wrapper	Instance-Net	Bag-Net	Bag-AttentionNet
		R^2					
Aggregation Method	Boltzmann Weight	0.980 (0.000)	0.858 (0.005)	0.852 (0.011)	0.856 (0.008)	0.853 (0.011)	0.853 (0.014)
	Mean	0.982 (0.000)	0.872 (0.006)	0.869 (0.006)	0.859 (0.019)	0.866 (0.010)	0.866 (0.018)
	Global Minimum	0.979 (0.001)	0.845 (0.010)	0.850 (0.008)	0.847 (0.007)	0.838 (0.023)	0.839 (0.016)
	Random	0.979 (0.000)	0.849 (0.010)	0.846 (0.013)	0.849 (0.012)	0.850 (0.010)	0.850 (0.011)
	RMSD max.	0.979 (0.000)	0.847 (0.009)	0.845 (0.006)	0.837 (0.013)	0.842 (0.019)	0.845 (0.016)
	RMSD min.	0.979 (0.000)	0.845 (0.015)	0.848 (0.016)	0.851 (0.013)	0.847 (0.027)	0.853 (0.009)
Non-Aggregation		0.989 (0.000)	0.908 (0.004)	0.864 (0.010)	0.868 (0.008)	0.867 (0.009)	0.869 (0.015)
Ground-truth		0.982 (0.000)	0.855 (0.012)	0.854 (0.014)	0.858 (0.006)	0.857 (0.007)	0.851 (0.010)

Table S16. Averaged prediction accuracy (R^2) of RF and MIL models for 15 training sets of the PQC data set HOMO-LUMO gap prediction using Pmapper descriptors. The standard deviations are provided in parentheses.

Data set = PQC (Training sets)<br>Descs = Pmapper<br>y = HOMO-LUMO gap (eV)		RF	MIL				
			Non-aggregation (Instance-Wrapper)	Bag-Wrapper	Instance-Net	Bag-Net	Bag-AttentionNet
		R^2					
Aggregation Method	Boltzmann Weight	0.973 (0.001)	0.917 (0.003)	0.914 (0.009)	0.918 (0.003)	0.917 (0.005)	0.914 (0.005)
	Mean	0.975 (0.000)	0.922 (0.010)	0.923 (0.005)	0.922 (0.013)	0.925 (0.008)	0.925 (0.008)
	Global Minimum	0.972 (0.000)	0.008 (0.003)	0.007 (0.005)	0.007 (0.003)	0.008 (0.003)	0.008 (0.003)
	Random	0.970 (0.001)	0.007 (0.006)	0.007 (0.004)	0.005 (0.006)	0.007 (0.004)	0.003 (0.008)
	RMSD max.	0.970 (0.001)	0.006 (0.005)	0.005 (0.005)	0.004 (0.009)	0.004 (0.005)	0.006 (0.004)
	RMSD min.	0.972 (0.000)	0.006 (0.002)	0.005 (0.006)	0.006 (0.003)	0.006 (0.003)	0.007 (0.003)
Non-Aggregation		0.980 (0.002)	0.926 (0.002)	0.916 (0.010)	0.918 (0.012)	0.919 (0.010)	0.902 (0.006)
Ground-truth		0.974 (0.000)	0.001 (0.008)	0.003 (0.006)	0.005 (0.003)	0.004 (0.004)	0.003 (0.006)

Table S17. Averaged prediction accuracy (R^2) of RF and MIL models for 15 training sets of the PQC data set HOMO-LUMO gap prediction using 3D-MoRSE descriptors. The standard deviations are provided in parentheses.

Data set = PQC (Training sets)<br>Descs = 3D-MoRSE<br>y = HOMO-LUMO gap (eV)		RF	MIL				
			Non-aggregation (Instance-Wrapper)	Bag-Wrapper	Instance-Net	Bag-Net	Bag-AttentionNet
		R^2					
Aggregation Method	Boltzmann Weight	0.978 (0.000)	0.863 (0.010)	0.865 (0.013)	0.860 (0.015)	0.864 (0.012)	0.860 (0.011)
	Mean	0.980 (0.001)	0.877 (0.008)	0.877 (0.012)	0.878 (0.015)	0.876 (0.013)	0.873 (0.014)
	Global Minimum	0.976 (0.000)	0.863 (0.004)	0.856 (0.015)	0.859 (0.009)	0.862 (0.008)	0.854 (0.014)
	Random	0.976 (0.000)	0.857 (0.011)	0.857 (0.011)	0.861 (0.006)	0.862 (0.008)	0.853 (0.017)
	RMSD max.	0.975 (0.000)	0.851 (0.019)	0.861 (0.008)	0.849 (0.022)	0.855 (0.016)	0.855 (0.014)
	RMSD min.	0.977 (0.000)	0.858 (0.014)	0.859 (0.008)	0.859 (0.016)	0.861 (0.015)	0.863 (0.010)
Non-Aggregation		0.981 (0.000)	0.909 (0.006)	0.876 (0.006)	0.876 (0.005)	0.876 (0.010)	0.881 (0.006)
Ground-truth		0.976 (0.000)	0.861 (0.010)	0.863 (0.007)	0.858 (0.014)	0.862 (0.013)	0.858 (0.009)

Table S18. Averaged prediction accuracy (R^2) of RF and MIL models for 15 training sets of the PQC data set HOMO-LUMO gap prediction using MBTR descriptors. The standard deviations are provided in parentheses.

Data set = PQC (Training sets)<br>Descs = MBTR<br>y = HOMO-LUMO gap (eV)		RF	MIL				
			Non-aggregation (Instance-Wrapper)	Bag-Wrapper	Instance-Net	Bag-Net	Bag-AttentionNet
		R^2					
Aggregation Method	Boltzmann Weight	0.988 (0.000)	0.922 (0.014)	0.926 (0.008)	0.926 (0.010)	0.925 (0.011)	0.925 (0.012)
	Mean	0.989 (0.000)	0.930 (0.006)	0.927 (0.012)	0.921 (0.022)	0.930 (0.007)	0.931 (0.007)
	Global Minimum	0.988 (0.000)	0.927 (0.007)	0.927 (0.006)	0.923 (0.012)	0.924 (0.011)	0.920 (0.017)
	Random	0.988 (0.000)	0.924 (0.009)	0.927 (0.005)	0.924 (0.008)	0.924 (0.007)	0.923 (0.014)
	RMSD max.	0.988 (0.000)	0.919 (0.015)	0.924 (0.011)	0.922 (0.011)	0.923 (0.007)	0.921 (0.017)
	RMSD min.	0.988 (0.000)	0.923 (0.012)	0.930 (0.006)	0.927 (0.005)	0.924 (0.009)	0.925 (0.008)
Non-Aggregation		0.995 (0.000)	0.949 (0.010)	0.929 (0.011)	0.928 (0.012)	0.929 (0.010)	0.932 (0.007)
Ground-truth		0.989 (0.000)	0.933 (0.009)	0.932 (0.009)	0.927 (0.012)	0.936 (0.002)	0.935 (0.006)

Table S19. Averaged prediction accuracy (R^2) of RF and MIL models for 15 training sets of the PQC data set LUMO prediction using MOE descriptors. The standard deviations are provided in parentheses.

Data set = PQC (Training sets)<br>Descs = MOE<br>y = LUMO (eV)		RF	MIL				
			Non-aggregation (Instance-Wrapper)	Bag-Wrapper	Instance-Net	Bag-Net	Bag-AttentionNet
		R^2					
Aggregation Method	Boltzmann Weight	0.986 (0.000)	0.926 (0.008)	0.925 (0.008)	0.928 (0.007)	0.930 (0.003)	0.925 (0.013)
	Mean	0.987 (0.000)	0.932 (0.008)	0.936 (0.004)	0.934 (0.006)	0.937 (0.003)	0.935 (0.010)
	Global Minimum	0.985 (0.000)	0.923 (0.005)	0.920 (0.014)	0.919 (0.010)	0.922 (0.008)	0.921 (0.005)
	Random	0.985 (0.000)	0.923 (0.006)	0.923 (0.005)	0.921 (0.009)	0.918 (0.011)	0.926 (0.004)
	RMSD max.	0.984 (0.000)	0.924 (0.004)	0.916 (0.008)	0.920 (0.007)	0.918 (0.011)	0.919 (0.009)
	RMSD min.	0.985 (0.000)	0.925 (0.005)	0.928 (0.003)	0.926 (0.007)	0.927 (0.004)	0.925 (0.004)
Non-Aggregation		0.992 (0.002)	0.942 (0.002)	0.935 (0.005)	0.934 (0.005)	0.932 (0.007)	0.924 (0.006)
Ground-truth		0.983 (0.001)	0.909 (0.008)	0.908 (0.009)	0.910 (0.007)	0.911 (0.008)	0.913 (0.005)

Table S20. Averaged prediction accuracy (R^2) of RF and MIL models for 15 training sets of the PQC data set LUMO prediction using Pmapper descriptors. The standard deviations are provided in parentheses.

Data set = PQC (Training sets)<br>Descs = Pmapper<br>y = LUMO (eV)		RF	MIL				
			Non-aggregation (Instance-Wrapper)	Bag-Wrapper	Instance-Net	Bag-Net	Bag-AttentionNet
		R^2					
Aggregation Method	Boltzmann Weight	0.964 (0.001)	0.926 (0.007)	0.928 (0.006)	0.926 (0.005)	0.930 (0.002)	0.927 (0.006)
	Mean	0.967 (0.000)	0.936 (0.008)	0.939 (0.004)	0.937 (0.009)	0.937 (0.008)	0.940 (0.002)
	Global Minimum	0.962 (0.001)	0.006 (0.004)	0.008 (0.002)	0.007 (0.004)	0.007 (0.004)	0.008 (0.003)
	Random	0.962 (0.001)	0.005 (0.005)	0.003 (0.004)	0.006 (0.004)	0.004 (0.004)	0.005 (0.004)
	RMSD max.	0.961 (0.001)	0.005 (0.005)	0.007 (0.004)	0.007 (0.004)	0.005 (0.005)	0.007 (0.004)
	RMSD min.	0.963 (0.000)	0.006 (0.004)	0.007 (0.002)	0.006 (0.003)	0.006 (0.003)	0.007 (0.002)
Non-Aggregation		0.974 (0.001)	0.926 (0.006)	0.933 (0.009)	0.935 (0.008)	0.935 (0.008)	0.909 (0.010)
Ground-truth		0.964 (0.000)	0.005 (0.003)	0.004 (0.003)	0.004 (0.003)	0.004 (0.003)	0.004 (0.004)

Table S21. Averaged prediction accuracy (R^2) of RF and MIL models for 15 training sets of the PQC data set LUMO prediction using 3D-MoRSE descriptors. The standard deviations are provided in parentheses.

Data set = PQC (Training sets)<br>Descs = 3D-MoRSE<br>y = LUMO (eV)		RF	MIL				
			Non-aggregation (Instance-Wrapper)	Bag-Wrapper	Instance-Net	Bag-Net	Bag-AttentionNet
		R^2					
Aggregation Method	Boltzmann Weight	0.979 (0.000)	0.918 (0.020)	0.928 (0.010)	0.919 (0.016)	0.931 (0.009)	0.930 (0.008)
	Mean	0.982 (0.000)	0.937 (0.007)	0.939 (0.007)	0.938 (0.005)	0.935 (0.006)	0.938 (0.007)
	Global Minimum	0.977 (0.000)	0.926 (0.008)	0.921 (0.008)	0.921 (0.011)	0.925 (0.007)	0.918 (0.027)
	Random	0.977 (0.000)	0.918 (0.028)	0.925 (0.006)	0.924 (0.011)	0.921 (0.012)	0.926 (0.009)
	RMSD max.	0.976 (0.001)	0.923 (0.008)	0.924 (0.007)	0.924 (0.007)	0.921 (0.009)	0.924 (0.007)
	RMSD min.	0.977 (0.001)	0.927 (0.007)	0.930 (0.005)	0.925 (0.011)	0.925 (0.011)	0.928 (0.006)
Non-Aggregation		0.984 (0.001)	0.940 (0.013)	0.933 (0.009)	0.934 (0.008)	0.932 (0.008)	0.924 (0.012)
Ground-truth		0.978 (0.001)	0.925 (0.011)	0.926 (0.007)	0.928 (0.011)	0.927 (0.012)	0.928 (0.009)

Table S22. Averaged prediction accuracy (R^2) of RF and MIL models for 15 training sets of the PQC data set LUMO prediction using MBTR descriptors. The standard deviations are provided in parentheses.

Data set = PQC (Training sets)<br>Descs = MBTR<br>y = LUMO (eV)		RF	MIL				
			Non-aggregation (Instance-Wrapper)	Bag-Wrapper	Instance-Net	Bag-Net	Bag-AttentionNet
		R^2					
Aggregation Method	Boltzmann Weight	0.992 (0.000)	0.961 (0.007)	0.965 (0.003)	0.963 (0.005)	0.963 (0.004)	0.962 (0.004)
	Mean	0.992 (0.000)	0.968 (0.002)	0.965 (0.003)	0.961 (0.011	0.964 (0.008)	0.967 (0.002)
	Global Minimum	0.991 (0.000)	0.962 (0.008)	0.963 (0.005)	0.963 (0.005)	0.964 (0.003)	0.963 (0.006)
	Random	0.991 (0.000)	0.963 (0.006)	0.962 (0.009)	0.963 (0.008)	0.961 (0.006)	0.965 (0.004)
	RMSD max.	0.991 (0.000)	0.964 (0.004)	0.963 (0.004)	0.961 (0.007)	0.961 (0.008)	0.961 (0.007)
	RMSD min.	0.992 (0.000)	0.964 (0.007)	0.964 (0.007)	0.966 (0.004)	0.964 (0.005)	0.962 (0.013)
Non-Aggregation		0.997 (0.000)	0.972 (0.004)	0.965 (0.003)	0.962 (0.010)	0.964 (0.005)	0.965 (0.003)
Ground-truth		0.992 (0.000)	0.966 (0.010)	0.970 (0.003)	0.966 (0.006)	0.966 (0.009)	0.967 (0.006)

Table S23. Averaged prediction accuracy (R^2) of RF and MIL models for 15 training sets of the PQC data set energy prediction using MOE descriptors. The standard deviations are provided in parentheses.

Data set = PQC (Training sets)<br>Descs = MOE<br>y = Energy (eV)		RF	MIL				
			Non-aggregation (Instance-Wrapper)	Bag-Wrapper	Instance-Net	Bag-Net	Bag-AttentionNet
		R^2					
Aggregation Method	Boltzmann Weight	0.983 (0.000)	0.940 (0.015)	0.947 (0.008)	0.947 (0.006)	0.947 (0.009)	0.945 (0.009)
	Mean	0.986 (0.000)	0.956 (0.014)	0.955 (0.013)	0.963 (0.004)	0.962 (0.005)	0.956 (0.010)
	Global Minimum	0.983 (0.000)	0.938 (0.012)	0.937 (0.014)	0.941 (0.006)	0.942 (0.004)	0.939 (0.010)
	Random	0.983 (0.000)	0.940 (0.010)	0.940 (0.009)	0.944 (0.008)	0.946 (0.011)	0.946 (0.005)
	RMSD max.	0.982 (0.000)	0.937 (0.012)	0.940 (0.012)	0.938 (0.015)	0.939 (0.011)	0.939 (0.012)
	RMSD min.	0.983 (0.000)	0.943 (0.008)	0.946 (0.005)	0.947 (0.008)	0.946 (0.008)	0.942 (0.011)
Non-Aggregation		0.993 (0.001)	0.975 (0.004)	0.961 (0.006)	0.961 (0.005)	0.959 (0.006)	0.952 (0.011)
Ground-truth		0.979 (0.002)	0.933 (0.021)	0.921 (0.044)	0.936 (0.023)	0.930 (0.027)	0.940 (0.015)

Table S24. Averaged prediction accuracy (R^2) of RF and MIL models for 15 training sets of the PQC data set energy prediction using Pmapper descriptors. The standard deviations are provided in parentheses.

Data set = PQC (Training sets)<br>Descs = Pmapper<br>y = Energy (eV)		RF	MIL				
			Non-aggregation (Instance-Wrapper)	Bag-Wrapper	Instance-Net	Bag-Net	Bag-AttentionNet
		R^2					
Aggregation Method	Boltzmann Weight	0.956 (0.000)	0.911 (0.006)	0.912 (0.005)	0.912 (0.002)	0.913 (0.003)	0.913 (0.003)
	Mean	0.960 (0.000)	0.929 (0.004)	0.928 (0.003)	0.929 (0.003)	0.927 (0.007)	0.929 (0.003)
	Global Minimum	0.954 (0.000)	0.016 (0.001)	0.016 (0.001)	0.016 (0.001)	0.015 (0.001)	0.015 (0.001)
	Random	0.954 (0.000)	0.019 (0.001)	0.019 (0.001)	0.018 (0.001)	0.019 (0.001)	0.019 (0.001)
	RMSD max.	0.953 (0.000)	0.018 (0.002)	0.018 (0.001)	0.018 (0.001)	0.018 (0.002)	0.018 (0.001)
	RMSD min.	0.954 (0.000)	0.020 (0.001)	0.019 (0.001)	0.019 (0.001)	0.020 (0.001)	0.019 (0.002)
Non-Aggregation		0.967 (0.006)	0.950 (0.001)	0.929 (0.004)	0.925 (0.004)	0.925 (0.004)	0.923 (0.008)
Ground-truth		0.955 (0.000)	0.016 (0.001)	0.016 (0.001)	0.016 (0.001)	0.016 (0.001)	0.016 (0.001)

Table S25. Averaged prediction accuracy (R^2) of RF and MIL models for 15 training sets of the PQC data set energy prediction using 3D-MoRSE descriptors. The standard deviations are provided in parentheses.

Data set = PQC (Training sets)<br>Descs = 3D-MoRSE<br>y = Energy (eV)		RF	MIL				
			Non-aggregation (Instance-Wrapper)	Bag-Wrapper	Instance-Net	Bag-Net	Bag-AttentionNet
		R^2					
Aggregation Method	Boltzmann Weight	0.964 (0.000)	0.937 (0.015)	0.941 (0.018)	0.943 (0.011)	0.930 (0.040)	0.942 (0.014)
	Mean	0.972 (0.000)	0.954 (0.011)	0.953 (0.017)	0.957 (0.008)	0.960 (0.011)	0.952 (0.019)
	Global Minimum	0.960 (0.001)	0.922 (0.023)	0.933 (0.022)	0.908 (0.051)	0.925 (0.025)	0.928 (0.021)
	Random	0.961 (0.000)	0.938 (0.018)	0.931 (0.019)	0.930 (0.020)	0.920 (0.042)	0.940 (0.013)
	RMSD max.	0.960 (0.000)	0.931 (0.019)	0.936 (0.011)	0.926 (0.028)	0.931 (0.013)	0.935 (0.017)
	RMSD min.	0.961 (0.000)	0.935 (0.019)	0.932 (0.023)	0.937 (0.017)	0.931 (0.030)	0.920 (0.026)
Non-Aggregation		0.975 (0.000)	0.977 (0.008)	0.946 (0.012)	0.942 (0.031)	0.945 (0.021)	0.948 (0.012)
Ground-truth		0.960 (0.000)	0.923 (0.032)	0.930 (0.023)	0.922 (0.035)	0.940 (0.014)	0.919 (0.039)

Table S26. Averaged prediction accuracy (R^2) of RF and MIL models for 15 training sets of the PQC data set energy prediction using MBTR descriptors. The standard deviations are provided in parentheses.

Data set = PQC (Training sets)<br>Descs = MBTR<br>y = Energy (eV)		RF	MIL				
			Non-aggregation (Instance-Wrapper)	Bag-Wrapper	Instance-Net	Bag-Net	Bag-AttentionNet
		R^2					
Aggregation Method	Boltzmann Weight	0.995 (0.000)	0.992 (0.002)	0.991 (0.003)	0.990 (0.004)	0.991 (0.003)	0.991 (0.003)
	Mean	0.995 (0.001)	0.994 (0.001)	0.992 (0.003)	0.992 (0.002)	0.993 (0.002)	0.993 (0.002)
	Global Minimum	0.994 (0.000)	0.992 (0.001)	0.991 (0.003)	0.991 (0.003)	0.989 (0.005)	0.992 (0.001)
	Random	0.993 (0.005)	0.990 (0.003)	0.990 (0.005)	0.990 (0.002)	0.989 (0.003)	0.990 (0.003)
	RMSD max.	0.994 (0.000)	0.990 (0.002)	0.991 (0.003)	0.990 (0.003)	0.989 (0.004)	0.991 (0.002)
	RMSD min.	0.994 (0.000)	0.990 (0.002)	0.991 (0.002)	0.991 (0.002)	0.991 (0.003)	0.991 (0.002)
Non-Aggregation		0.998 (0.000)	0.996 (0.001)	0.994 (0.001)	0.994 (0.001)	0.993 (0.002)	0.993 (0.001)
Ground-truth		0.994 (0.000)	0.991 (0.003)	0.991 (0.003)	0.990 (0.002)	0.991 (0.002)	0.990 (0.002)

Table S27. Averaged prediction accuracy (R^2) of RF and MIL models for 15 training sets of the PQC data set enthalpy prediction using MOE descriptors. The standard deviations are provided in parentheses.

Data set = PQC (Training sets)<br>Descs = MOE<br>y = Enthalpy (Hartree)		RF	MIL				
			Non-aggregation (Instance-Wrapper)	Bag-Wrapper	Instance-Net	Bag-Net	Bag-AttentionNet
		R^2					
Aggregation Method	Boltzmann Weight	0.988 (0.001)	0.932 (0.012)	0.933 (0.013)	0.930 (0.010)	0.933 (0.009)	0.931 (0.017)
	Mean	0.989 (0.000)	0.949 (0.009)	0.951 (0.006)	0.938 (0.018)	0.944 (0.016)	0.947 (0.008)
	Global Minimum	0.987 (0.000)	0.925 (0.010)	0.924 (0.013)	0.921 (0.013)	0.922 (0.013)	0.919 (0.013)
	Random	0.987 (0.000)	0.923 (0.020)	0.926 (0.011)	0.923 (0.012)	0.924 (0.009)	0.922 (0.020)
	RMSD max.	0.987 (0.000)	0.921 (0.024)	0.914 (0.034)	0.926 (0.016)	0.918 (0.024)	0.916 (0.018)
	RMSD min.	0.987 (0.000)	0.929 (0.010)	0.927 (0.010)	0.930 (0.012)	0.917 (0.026)	0.931 (0.008)
Non-Aggregation		0.995 (0.001)	0.945 (0.010)	0.945 (0.009)	0.937 (0.013)	0.942 (0.011)	0.931 (0.016)
Ground-truth		0.986 (0.000)	0.929 (0.013)	0.929 (0.020)	0.922 (0.022)	0.926 (0.015)	0.928 (0.013)

Table S28. Averaged prediction accuracy (R^2) of RF and MIL models for 15 training sets of the PQC data set enthalpy prediction using Pmapper descriptors. The standard deviations are provided in parentheses.

Data set = PQC (Training sets)<br>Descs = Pmapper<br>y = Enthalpy (Hartree)		RF	MIL				
			Non-aggregation (Instance-Wrapper)	Bag-Wrapper	Instance-Net	Bag-Net	Bag-AttentionNet
		R^2					
Aggregation Method	Boltzmann Weight	0.945 (0.000)	0.859 (0.031)	0.876 (0.010)	0.860 (0.030)	0.862 (0.027)	0.867 (0.018)
	Mean	0.951 (0.000)	0.871 (0.040)	0.881 (0.019)	0.876 (0.031)	0.876 (0.027)	0.882 (0.024)
	Global Minimum	0.941 (0.000)	-0.000 (0.000)	-0.000 (0.000)	-0.000 (0.000)	-0.000 (0.000)	-0.000 (0.000)
	Random	0.942 (0.000)	-0.000 (0.000)	-0.000 (0.000)	-0.000 (0.000)	-0.000 (0.000)	-0.000 (0.000)
	RMSD max.	0.940 (0.001)	-0.000 (0.000)	-0.000 (0.000)	-0.000 (0.000)	-0.000 (0.000)	-0.000 (0.001)
	RMSD min.	0.942 (0.000)	-0.000 (0.000)	-0.000 (0.000)	-0.000 (0.000)	-0.000 (0.000)	-0.000 (0.000)
Non-Aggregation		0.961 (0.001)	0.849 (0.009)	0.865 (0.031)	0.881 (0.016)	0.871 (0.023)	0.842 (0.010)
Ground-truth		0.943 (0.000)	-0.000 (0.000)	-0.000 (0.000)	-0.000 (0.001)	-0.000 (0.000)	-0.000 (0.000)

Table S29. Averaged prediction accuracy (R^2) of RF and MIL models for 15 training sets of the PQC data set enthalpy prediction using 3D-MoRSE descriptors. The standard deviations are provided in parentheses.

Data set = PQC (Training sets)<br>Descs = 3D-MoRSE<br>y = Enthalpy (Hartree)		RF	MIL				
			Non-aggregation (Instance-Wrapper)	Bag-Wrapper	Instance-Net	Bag-Net	Bag-AttentionNet
		R^2					
Aggregation Method	Boltzmann Weight	0.968 (0.000)	0.930 (0.013)	0.912 (0.045)	0.920 (0.031)	0.931 (0.023)	0.925 (0.021)
	Mean	0.975 (0.000)	0.949 (0.013)	0.943 (0.024)	0.939 (0.021)	0.945 (0.012)	0.942 (0.013)
	Global Minimum	0.965 (0.000)	0.913 (0.043)	0.910 (0.029)	0.911 (0.032)	0.919 (0.025)	0.908 (0.035)
	Random	0.965 (0.001)	0.914 (0.031)	0.919 (0.030)	0.927 (0.015)	0.927 (0.014)	0.929 (0.020)
	RMSD max.	0.964 (0.000)	0.921 (0.019)	0.918 (0.019)	0.915 (0.026)	0.915 (0.024)	0.911 (0.042)
	RMSD min.	0.966 (0.000)	0.927 (0.019)	0.921 (0.020)	0.920 (0.027)	0.905 (0.041)	0.924 (0.031)
Non-Aggregation		0.977 (0.001)	0.960 (0.015)	0.938 (0.025)	0.930 (0.028)	0.940 (0.012)	0.935 (0.022)
Ground-truth		0.964 (0.000)	0.917 (0.036)	0.906 (0.047)	0.911 (0.059)	0.915 (0.044)	0.914 (0.034)

Table S30. Averaged prediction accuracy (R^2) of RF and MIL models for 15 training sets of the PQC data set enthalpy prediction using MBTR descriptors. The standard deviations are provided in parentheses.

Data set = PQC (Training sets)<br>Descs = MBTR<br>y = Enthalpy (Hartree)		RF	MIL				
			Non-aggregation (Instance-Wrapper)	Bag-Wrapper	Instance-Net	Bag-Net	Bag-AttentionNet
		R^2					
Aggregation Method	Boltzmann Weight	0.996 (0.000)	0.977 (0.008)	0.979 (0.005)	0.979 (0.005)	0.981 (0.003)	0.977 (0.009)
	Mean	0.996 (0.000)	0.983 (0.003)	0.983 (0.003)	0.984 (0.002)	0.983 (0.004)	0.983 (0.004)
	Global Minimum	0.995 (0.001)	0.979 (0.004)	0.978 (0.006)	0.979 (0.006)	0.979 (0.005)	0.979 (0.004)
	Random	0.995 (0.000)	0.976 (0.014)	0.979 (0.003)	0.978 (0.003)	0.977 (0.008)	0.977 (0.008)
	RMSD max.	0.995 (0.000)	0.981 (0.003)	0.979 (0.004)	0.977 (0.008)	0.979 (0.003)	0.978 (0.006)
	RMSD min.	0.995 (0.000)	0.979 (0.004)	0.978 (0.004)	0.978 (0.007)	0.980 (0.003)	0.978 (0.005)
Non-Aggregation		0.999 (0.000)	0.981 (0.006)	0.984 (0.002)	0.981 (0.008)	0.981 (0.007)	0.981 (0.003)
Ground-truth		0.995 (0.000)	0.976 (0.006)	0.978 (0.004)	0.978 (0.004)	0.978 (0.004)	0.978 (0.004)

Table S31. Averaged prediction accuracy (R^2) of RF and MIL models for 15 training sets of the PQC data set six property predictions using ECFP4 count. The standard deviations are provided in parentheses.

Data set = PQC (Training sets) Descs = ECFP4 count		Dipole moment	HOMO	HOMO-LUMO gap	LUMO	Energy	Enthalpy
		R^2					
MIL	Non-aggregation	0.834 (0.002)	0.964 (0.003)	0.984 (0.001)	0.990 (0.000)	0.999 (0.000)	0.997 (0.000)
	Bag-Wrapper	0.831 (0.004)	0.964 (0.001)	0.984 (0.000)	0.990 (0.001)	0.998 (0.000)	0.997 (0.000)
	Instance-Net	0.830 (0.005)	0.964 (0.001)	0.984 (0.001)	0.990 (0.000)	0.998 (0.000)	0.997 (0.000)
	Bag-Net	0.833 (0.003)	0.964 (0.002)	0.984 (0.001)	0.990 (0.000)	0.998 (0.000)	0.997 (0.000)
	Bag-AttentionNet	0.832 (0.002)	0.964 (0.001)	0.984 (0.001)	0.990 (0.001)	0.998 (0.000)	0.997 (0.000)
RF		0.869 (0.020)	0.979 (0.001)	0.990 (0.000)	0.993 (0.000)	0.995 (0.000)	0.992 (0.000)

Table S32. Averaged prediction accuracy (R^2) of MolCLR, GEM, and Uni-Mol models for 15 training sets of the PQC data set six property predictions. The standard deviations are provided in parentheses.

Data set = PQC (Training sets)		Dipole moment	HOMO	HOMO-LUMO gap	LUMO	Energy	Enthalpy
		R^2					
GEM	Ground-truth	0.510 (0.012)	0.922 (0.002)	0.963 (0.001)	0.972 (0.002)	0.996 (0.001)	0.996 (0.001)
	Global min.	0.482 (0.007)	0.902 (0.004)	0.956 (0.002)	0.967 (0.003)	0.996 (0.001)	0.996 (0.001)
	RMSD max.	0.477 (0.010)	0.900 (0.005)	0.956 (0.002)	0.966 (0.002)	0.996 (0.001)	0.996 (0.001)
	Non-aggregation	0.534 (0.004)	0.922 (0.002)	0.967 (0.001)	0.975 (0.001)	0.997 (0.000)	0.997 (0.001)
Uni-Mol	Ground-truth	0.969 (0.002)	0.994 (0.001)	0.969 (0.001)	0.989 (0.000)	0.987 (0.001)	0.993 (0.000)
	Global min.	0.635 (0.017)	0.966 (0.004)	0.958 (0.002)	0.981 (0.001)	0.987 (0.001)	0.993 (0.000)
	RMSD max.	0.647 (0.012)	0.972 (0.005)	0.960 (0.002)	0.983 (0.001)	0.986 (0.001)	0.993 (0.000)
MolCLR		0.594 (0.008)	0.942 (0.001)	0.971 (0.001)	0.982 (0.000)	0.984 (0.001)	0.983 (0.001)

Table S33. Averaged prediction accuracy (R^2) of RF and MIL models for 25 training sets of the MP data set property (melting point) prediction using MOE descriptors. The standard deviations are provided in parentheses.

Data set = MP (Training sets)<br>Descs = MOE<br>y = Melting Point (degrees Celsius)		RF	MIL				
			Non-aggregation (Instance-Wrapper)	Bag-Wrapper	Instance-Net	Bag-Net	Bag-AttentionNet
		R^2					
Aggregation Method	Boltzmann Weight	0.970 (0.003)	0.872 (0.061)	0.873 (0.048)	0.874 (0.054)	0.881 (0.043)	0.875 (0.051)
	Mean	0.970 (0.003)	0.893 (0.035)	0.898 (0.031)	0.893 (0.035)	0.896 (0.033)	0.900 (0.030)
	Global Minimum	0.969 (0.003)	0.887 (0.041)	0.881 (0.047)	0.886 (0.039)	0.883 (0.050)	0.886 (0.047)
	Random	0.967 (0.003)	0.891 (0.033)	0.889 (0.035)	0.889 (0.034)	0.889 (0.031)	0.889 (0.032)
Non-Aggregation		1.000 (0.000)	0.995 (0.002)	0.886 (0.031)	0.901 (0.037)	0.899 (0.044)	0.874 (0.028)

Table S34. Averaged prediction accuracy (R^2) of RF and MIL models for 25 training sets of the MP data set property (melting point) prediction using Pmapper descriptors. The standard deviations are provided in parentheses.

Data set = MP (Training sets)<br>Descs = Pmapper<br>y = Melting Point (degrees Celsius)		RF	MIL				
			Non-aggregation (Instance-Wrapper)	Bag-Wrapper	Instance-Net	Bag-Net	Bag-AttentionNet
		R^2					
Aggregation Method	Boltzmann Weight	0.930 (0.006)	0.749 (0.098)	0.748 (0.092)	0.740 (0.121)	0.744 (0.114)	0.746 (0.128)
	Mean	0.933 (0.006)	0.731 (0.093)	0.737 (0.086)	0.744 (0.089)	0.734 (0.088)	0.731 (0.091)
	Global Minimum	0.928 (0.006)	0.395 (0.056)	0.396 (0.056)	0.395 (0.055)	0.395 (0.056)	0.396 (0.056)
	Random	0.928 (0.005)	0.412 (0.058)	0.412 (0.060)	0.412 (0.059)	0.412 (0.059)	0.412 (0.059)
Non-Aggregation		0.994 (0.001)	0.992 (0.002)	0.735 (0.099)	0.730 (0.088)	0.733 (0.084)	0.767 (0.047)

Table S35. Averaged prediction accuracy (R^2) of RF and MIL models for 25 training sets of the MP data set property (melting point) prediction using 3D-MoRSE descriptors. The standard deviations are provided in parentheses.

Data set = MP (Training sets)<br>Descs = 3D-MoRSE<br>y = Melting Point (degrees Celsius)		RF	MIL				
			Non-aggregation (Instance-Wrapper)	Bag-Wrapper	Instance-Net	Bag-Net	Bag-AttentionNet
		R^2					
Aggregation Method	Boltzmann Weight	0.948 (0.004)	0.858 (0.024)	0.857 (0.022)	0.855 (0.024)	0.856 (0.020)	0.856 (0.019)
	Mean	0.951 (0.004)	0.873 (0.023)	0.876 (0.025)	0.875 (0.023)	0.877 (0.026)	0.874 (0.024)
	Global Minimum	0.947 (0.004)	0.862 (0.030)	0.859 (0.028)	0.864 (0.024)	0.859 (0.025)	0.861 (0.026)
	Random	0.943 (0.004)	0.854 (0.030)	0.859 (0.029)	0.854 (0.027)	0.856 (0.027)	0.855 (0.024)
Non-Aggregation		0.999 (0.000)	0.990 (0.004)	0.848 (0.025)	0.852 (0.024)	0.852 (0.027)	0.818 (0.031)

Table S36. Averaged prediction accuracy (R^2) of RF and MIL models for 25 training sets of the MP data set property (melting point) prediction using MBTR descriptors. The standard deviations are provided in parentheses.

Data set = MP (Training sets)<br>Descs = MBTR<br>y = Melting Point (degrees Celsius)		RF	MIL				
			Non-aggregation (Instance-Wrapper)	Bag-Wrapper	Instance-Net	Bag-Net	Bag-AttentionNet
		R^2					
Aggregation Method	Boltzmann Weight	0.948 (0.005)	0.861 (0.101)	0.855 (0.118)	0.853 (0.120)	0.857 (0.110)	0.855 (0.111)
	Mean	0.952 (0.005)	0.808 (0.157)	0.799 (0.185)	0.819 (0.162)	0.816 (0.145)	0.805 (0.221)
	Global Minimum	0.946 (0.005)	0.830 (0.114)	0.822 (0.148)	0.817 (0.147)	0.833 (0.115)	0.837 (0.112)
	Random	0.946 (0.004)	0.846 (0.091)	0.845 (0.097)	0.841 (0.110)	0.831 (0.123)	0.840 (0.111)
Non-Aggregation		1.000 (0.000)	0.998 (0.001)	0.811 (0.209)	0.843 (0.106)	0.820 (0.140)	0.822 (0.149)

Table S37. Averaged prediction accuracy (R^2) of RF and MIL models for 25 training sets of the MP data set property (melting point) prediction using ECFP4 count. The standard deviations are provided in parentheses.

Data set = MP (Training sets)<br>Descs = ECFP4 count<br>y = Melting Point (degrees Celsius)		R^2
MIL	Non-aggregation	0.882 (0.055)
	Bag-Wrapper	0.883 (0.053)
	Instance-Net	0.882 (0.057)
	Bag-Net	0.880 (0.055)
	Bag-AttentionNet	0.881 (0.055)
RF		0.950 (0.005)

Table S38. Averaged prediction accuracy (R^2) of MolCLR, GEM, and Uni-Mol models for 25 training sets of the MP data set property (melting point) prediction. The standard deviations are provided in parentheses.

Data set = MP (Training sets)<br>y = Melting Point (degrees Celsius)		R^2
GEM	Global min.	0.924 (0.022)
	Non-aggregation	0.996 (0.001)
Uni-Mol	Global min.	0.912 (0.017)
	Non-aggregation	0.990 (0.017)
MolCLR		0.871 (0.051)

Table S39. Averaged prediction accuracy (R^2) of RF, Elastic Net, PLS, SVM, and MIL models for 25 training sets of the APTC-1 data set property ($\Delta\Delta G^{\ddagger}$) prediction using MOE descriptors. The standard deviations are provided in parentheses.

Data set = APTC-1 (Training sets)<br>Descs = MOE<br>y = $\Delta\Delta G^{\ddagger}$(kcal/mol)		RF	Elastic Net	PLS	SVM	MIL				
						Non-aggregation (Instance-Wrapper)	Bag-Wrapper	Instance-Net	Bag-Net	Bag-AttentionNet
		R^2								
Aggregation Method	Boltzmann Weight	0.911 (0.010)	0.000 (0.000)	0.645 (0.043)	0.833 (0.032)	0.756 (0.083)	0.761 (0.084)	0.757 (0.077)	0.768 (0.073)	0.763 (0.078)
	Mean	0.915 (0.012)	0.000 (0.000)	0.592 (0.035)	0.775 (0.034)	0.815 (0.056)	0.806 (0.061)	0.812 (0.058)	0.818 (0.061)	0.816 (0.056)
	Global Minimum	0.906 (0.010)	0.000 (0.000)	0.633 (0.044)	0.826 (0.034)	0.755 (0.080)	0.755 (0.080)	0.758 (0.075)	0.753 (0.083)	0.761 (0.086)
	Random	0.918 (0.012)	0.000 (0.000)	0.611 (0.038)	0.819 (0.028)	0.737 (0.087)	0.742 (0.087)	0.742 (0.086)	0.748 (0.084)	0.749 (0.082)
Non-Aggregation		0.989 (0.002)	-0.000 (0.000)	0.522 (0.039)	0.913 (0.012)	0.995 (0.003)	0.748 (0.053)	0.734 (0.065)	0.744 (0.046)	0.654 (0.065)

Table S40. Averaged prediction accuracy (R^2) of RF, Elastic Net, PLS, SVM, and MIL models for 25 training sets of the APTC-1 data set property ($\Delta\Delta G^{\ddagger}$) prediction using Pmapper descriptors. The standard deviations are provided in parentheses.

Data set = APTC-1 (Training sets)<br>Descs = Pmapper<br>y = $\Delta\Delta G^{\ddagger}$(kcal/mol)		RF	Elastic Net	PLS	SVM	MIL				
						Non-aggregation (Instance-Wrapper)	Bag-Wrapper	Instance-Net	Bag-Net	Bag-AttentionNet
		R^2								
Aggregation Method	Boltzmann Weight	0.917 (0.010)	0.000 (0.000)	0.950 (0.009)	0.943 (0.006)	0.851 (0.054)	0.853 (0.054)	0.849 (0.055)	0.850 (0.056)	0.853 (0.054)
	Mean	0.923 (0.009)	0.000 (0.000)	0.822 (0.029)	0.908 (0.012)	0.921 (0.040)	0.922 (0.039)	0.923 (0.038)	0.921 (0.038)	0.921 (0.039)
	Global Minimum	0.911 (0.012)	0.000 (0.000)	0.966 (0.006)	0.942 (0.006)	0.821 (0.066)	0.823 (0.069)	0.820 (0.067)	0.824 (0.067)	0.819 (0.068)
	Random	0.920 (0.011)	0.000 (0.000)	0.968 (0.003)	0.946 (0.005)	0.805 (0.065)	0.807 (0.063)	0.803 (0.065)	0.805 (0.058)	0.808 (0.058)
Non-Aggregation		0.993 (0.002)	-0.000 (0.000)	0.728 (0.019)	0.967 (0.002)	0.996 (0.002)	0.938 (0.030)	0.938 (0.026)	0.939 (0.027)	0.904 (0.018)

Table S41. Averaged prediction accuracy (R^2) of RF, Elastic Net, PLS, SVM, and MIL models for 25 training sets of the APTC-1 data set property ($\Delta\Delta G^{\ddagger}$) prediction using 3D-MoRSE descriptors. The standard deviations are provided in parentheses.

Data set = APTC-1 (Training sets) Descs = 3D-MoRSE y = $\Delta\Delta G^{\ddagger}$(kcal/mol)		RF	Elastic Net	PLS	SVM	MIL				
						Non-aggregation (Instance-Wrapper)	Bag-Wrapper	Instance-Net	Bag-Net	Bag-AttentionNet
		R^2								
Aggregation Method	Boltzmann Weight	0.888 (0.010)	0.000 (0.000)	0.762 (0.029)	0.917 (0.015)	0.831 (0.081)	0.833 (0.076)	0.828 (0.088)	0.831 (0.076)	0.832 (0.085)
	Mean	0.905 (0.008)	0.000 (0.000)	0.695 (0.038)	0.804 (0.026)	0.893 (0.040)	0.897 (0.033)	0.889 (0.040)	0.897 (0.036)	0.890 (0.037)
	Global Minimum	0.880 (0.009)	0.000 (0.000)	0.747 (0.029)	0.920 (0.014)	0.764 (0.078)	0.760 (0.082)	0.766 (0.080)	0.765 (0.076)	0.765 (0.074)
	Random	0.891 (0.010)	0.000 (0.000)	0.701 (0.044)	0.928 (0.011)	0.774 (0.074)	0.770 (0.069)	0.761 (0.070)	0.761 (0.074)	0.770 (0.073)
Non-Aggregation		0.977 (0.004)	-0.000 (0.000)	0.518 (0.038)	0.930 (0.010)	0.977 (0.030)	0.778 (0.048)	0.743 (0.050)	0.746 (0.065)	0.587 (0.045)

Table S42. Averaged prediction accuracy (R^2) of RF, Elastic Net, PLS, SVM, and MIL models for 25 training sets of the APTC-1 data set property ($\Delta\Delta G^{\ddagger}$) prediction using MBTR descriptors. The standard deviations are provided in parentheses.

Data set = APTC-1 (Training sets)<br>Descs = MBTR<br>y = $\Delta\Delta G^{\ddagger}$(kcal/mol)		RF	Elastic Net	PLS	SVM	MIL				
						Non-aggregation (Instance-Wrapper)	Bag-Wrapper	Instance-Net	Bag-Net	Bag-AttentionNet
		R^2								
Aggregation Method	Boltzmann Weight	0.901 (0.011)	0.000 (0.000)	0.740 (0.032)	0.777 (0.033)	0.810 (0.091)	0.810 (0.089)	0.810 (0.093)	0.809 (0.094)	0.808 (0.092)
	Mean	0.908 (0.008)	0.000 (0.000)	0.676 (0.031)	0.764 (0.037)	0.842 (0.072)	0.840 (0.071)	0.840 (0.066)	0.841 (0.071)	0.843 (0.069)
	Global Minimum	0.899 (0.009)	0.000 (0.000)	0.712 (0.032)	0.788 (0.035)	0.779 (0.078)	0.777 (0.071)	0.776 (0.080)	0.778 (0.079)	0.776 (0.075)
	Random	0.889 (0.011)	0.000 (0.000)	0.696 (0.029)	0.770 (0.034)	0.761 (0.073)	0.756 (0.080)	0.759 (0.078)	0.762 (0.082)	0.759 (0.081)
Non-Aggregation		0.998 (0.001)	-0.000 (0.000)	0.547 (0.039)	0.868 (0.018)	0.999 (0.001)	0.801 (0.067)	0.800 (0.057)	0.779 (0.092)	0.745 (0.056)

Table S43. Averaged prediction accuracy (R^2) of RF, Elastic Net, PLS, SVM, and MIL models for 25 training sets of the APTC-1 data set property ($\Delta\Delta G^{\ddagger}$) prediction using ECFP4 bit, ECFP4 count, and 2D PFP. The standard deviations are provided in parentheses.

Data set = APTC-1 (Training sets) Descs = 2D y = $\Delta\Delta G^{\ddagger}$(kcal/mol)	RF	Elastic Net	PLS	SVM	MIL				
					Non-aggregation (Instance-Wrapper)	Bag-Wrapper	Instance-Net	Bag-Net	Bag-AttentionNet
	R^2								
ECFP4 bit	0.899 (0.019)	0.000 (0.000)	0.776 (0.023)	0.887 (0.019)	0.871 (0.028)	0.867 (0.028)	0.868 (0.029)	0.871 (0.028)	0.870 (0.030)
ECFP4 count	0.898 (0.019)	0.000 (0.000)	0.782 (0.023)	0.899 (0.016)	0.758 (0.076)	0.759 (0.077)	0.760 (0.076)	0.761 (0.075)	0.758 (0.076)
2D PFP	0.930 (0.014)	0.000 (0.000)	0.754 (0.028)	0.861 (0.016)	0.644 (0.050)	0.643 (0.050)	0.644 (0.049)	0.643 (0.050)	0.643 (0.049)

Table S44. Averaged prediction accuracy (R^2) of MolCLR, GEM, and Uni-Mol models for 25 training sets of the APTC-1 data set property ($\Delta\Delta G^{\ddagger}$) prediction. The standard deviations are provided in parentheses.

Data set = APTC-1 (Training sets)<br>y = $\Delta\Delta G^{\ddagger}$(kcal/mol)		R^2
GEM	Global min.	0.350 (0.093)
	Non-aggregation	0.952 (0.022)
Uni-Mol	Global min.	0.547 (0.135)
	Non-aggregation	1.000 (0.000)
MolCLR		0.390 (0.119)

Table S45. Averaged prediction accuracy (R^2) of RF, Elastic Net, PLS, SVM, and MIL models for 40 training sets of the APTC-2 data set property ($\Delta\Delta G^{\ddagger}$) prediction using MOE descriptors. The standard deviations are provided in parentheses.

Data set = APTC-2 (Training sets) Descs = MOE y = $\Delta\Delta G^{\ddagger}$(kcal/mol)		RF	Elastic Net	PLS	SVM	MIL				
						Non-aggregation (Instance-Wrapper)	Bag-Wrapper	Instance-Net	Bag-Net	Bag-AttentionNet
		R^2								
Aggregation Method	Boltzmann Weight	0.927 (0.007)	-0.000 (0.000)	0.743 (0.012)	0.848 (0.019)	0.626 (0.100)	0.623 (0.100)	0.614 (0.106)	0.616 (0.102)	0.629 (0.103)
	Mean	0.913 (0.006)	-0.000 (0.000)	0.776 (0.017)	0.874 (0.021)	0.881 (0.114)	0.877 (0.122)	0.876 (0.114)	0.874 (0.119)	0.879 (0.116)
	Global Minimum	0.925 (0.006)	-0.000 (0.000)	0.703 (0.015)	0.845 (0.019)	0.621 (0.109)	0.632 (0.105)	0.626 (0.106)	0.633 (0.099)	0.629 (0.092)
	Random	0.911 (0.005)	-0.000 (0.000)	0.775 (0.019)	0.760 (0.025)	0.770 (0.076)	0.767 (0.083)	0.774 (0.076)	0.774 (0.066)	0.768 (0.094)
Non-Aggregation		1.000 (0.000)	-0.001 (0.000)	0.701 (0.017)	0.966 (0.004)	0.998 (0.001)	0.939 (0.057)	0.918 (0.073)	0.919 (0.071)	0.889 (0.059)

Table S46. Averaged prediction accuracy (R^2) of RF, Elastic Net, PLS, SVM, and MIL models for 40 training sets of the APTC-2 data set property ($\Delta\Delta G^{\ddagger}$) prediction using Pmapper descriptors. The standard deviations are provided in parentheses.

Data set = APTC-2 (Training sets)<br>Descs = Pmapper<br>y = $\Delta\Delta G^{\ddagger}$(kcal/mol)		RF	Elastic Net	PLS	SVM	MIL				
						Non-aggregation (Instance-Wrapper)	Bag-Wrapper	Instance-Net	Bag-Net	Bag-AttentionNet
		R^2								
Aggregation Method	Boltzmann Weight	0.928 (0.004)	-0.000 (0.000)	0.977 (0.002)	0.957 (0.004)	0.887 (0.067)	0.885 (0.071)	0.885 (0.081)	0.886 (0.067)	0.888 (0.072)
	Mean	0.954 (0.004)	-0.000 (0.000)	0.915 (0.007)	0.958 (0.006)	0.889 (0.083)	0.891 (0.089)	0.887 (0.102)	0.887 (0.097)	0.887 (0.106)
	Global Minimum	0.919 (0.004)	-0.000 (0.000)	0.979 (0.002)	0.958 (0.004)	0.773 (0.083)	0.777 (0.076)	0.774 (0.077)	0.774 (0.081)	0.780 (0.074)
	Random	0.926 (0.005)	-0.000 (0.000)	0.978 (0.002)	0.956 (0.005)	0.781 (0.085)	0.787 (0.080)	0.779 (0.089)	0.783 (0.087)	0.782 (0.081)
Non-Aggregation		0.997 (0.001)	-0.001 (0.000)	0.891 (0.009)	0.979 (0.002)	0.782 (0.081)	0.916 (0.037)	0.910 (0.043)	0.911 (0.040)	0.918 (0.049)

Table S47. Averaged prediction accuracy (R^2) of RF, Elastic Net, PLS, SVM, and MIL models for 40 training sets of the APTC-2 data set property ($\Delta\Delta G^{\ddagger}$) prediction using 3D-MoRSE descriptors. The standard deviations are provided in parentheses.

Data set = APTC-2 (Training sets)<br>Descs = 3D-MoRSE<br>y = $\Delta\Delta G^{\ddagger}$(kcal/mol)		RF	Elastic Net	PLS	SVM	MIL				
						Non-aggregation (Instance-Wrapper)	Bag-Wrapper	Instance-Net	Bag-Net	Bag-AttentionNet
		R^2								
Aggregation Method	Boltzmann Weight	0.906 (0.005)	0.000 (0.000)	0.848 (0.009)	0.907 (0.007)	0.840 (0.085)	0.852 (0.078)	0.851 (0.077)	0.851 (0.080)	0.847 (0.082)
	Mean	0.929 (0.004)	0.000 (0.000)	0.629 (0.019)	0.833 (0.018)	0.956 (0.034)	0.951 (0.036)	0.956 (0.031)	0.954 (0.036)	0.957 (0.030)
	Global Minimum	0.901 (0.005)	0.000 (0.000)	0.857 (0.008)	0.903 (0.007)	0.903 (0.040)	0.903 (0.037)	0.903 (0.035)	0.904 (0.034)	0.905 (0.034)
	Random	0.912 (0.003)	0.000 (0.000)	0.901 (0.005)	0.925 (0.010)	0.872 (0.041)	0.868 (0.044)	0.873 (0.043)	0.876 (0.040)	0.876 (0.041)
Non-Aggregation		0.998 (0.000)	-0.001 (0.000)	0.607 (0.019)	0.981 (0.002)	0.999 (0.000)	0.937 (0.037)	0.922 (0.042)	0.918 (0.046)	0.878 (0.063)

Table S48. Averaged prediction accuracy (R^2) of RF, Elastic Net, PLS, SVM, and MIL models for 40 training sets of the APTC-2 data set property ($\Delta\Delta G^{\ddagger}$) prediction using MBTR descriptors. The standard deviations are provided in parentheses.

Data set = APTC-2 (Training sets) Descs = MBTR y = $\Delta\Delta G^{\ddagger}$(kcal/mol)		RF	Elastic Net	PLS	SVM	MIL				
						Non-aggregation (Instance-Wrapper)	Bag-Wrapper	Instance-Net	Bag-Net	Bag-AttentionNet
		R^2								
Aggregation Method	Boltzmann Weight	0.931 (0.006)	0.000 (0.000)	0.819 (0.016)	0.834 (0.016)	0.783 (0.121)	0.786 (0.113)	0.789 (0.113)	0.777 (0.122)	0.785 (0.117)
	Mean	0.935 (0.006)	0.000 (0.000)	0.841 (0.014)	0.862 (0.014)	0.953 (0.039)	0.953 (0.040)	0.953 (0.040)	0.954 (0.037)	0.956 (0.036)
	Global Minimum	0.935 (0.004)	0.000 (0.000)	0.805 (0.020)	0.826 (0.019)	0.926 (0.047)	0.927 (0.046)	0.928 (0.043)	0.928 (0.041)	0.926 (0.046)
	Random	0.932 (0.005)	0.000 (0.000)	0.891 (0.012)	0.890 (0.013)	0.964 (0.019)	0.964 (0.021)	0.965 (0.020)	0.964 (0.020)	0.965 (0.019)
Non-Aggregation		1.000 (0.000)	-0.001 (0.000)	0.753 (0.018)	0.962 (0.005)	1.000 (0.000)	0.950 (0.026)	0.942 (0.025)	0.941 (0.025)	0.944 (0.033)

Table S49. Averaged prediction accuracy (R^2) of RF, Elastic Net, PLS, SVM, and MIL models for 40 training sets of the APTC-2 data set property ($\Delta\Delta G^{\ddagger}$) prediction using ECFP4 bit, ECFP4 count, and 2D PFP. The standard deviations are provided in parentheses.

Data set = APTC-2 (Training sets) Descs = 2D y = $\Delta\Delta G^{\ddagger}$(kcal/mol)	RF	Elastic Net	PLS	SVM	MIL				
					Non-aggregation (Instance-Wrapper)	Bag-Wrapper	Instance-Net	Bag-Net	Bag-AttentionNet
	R^2								
ECFP4 bit	0.945 (0.005)	-0.000 (0.000)	0.654 (0.021)	0.939 (0.008)	0.885 (0.045)	0.886 (0.050)	0.884 (0.048)	0.889 (0.038)	0.886 (0.043)
ECFP4 count	0.934 (0.005)	-0.000 (0.000)	0.642 (0.020)	0.939 (0.010)	0.917 (0.064)	0.918 (0.066)	0.919 (0.070)	0.921 (0.061)	0.921 (0.063)
2D PFP	0.970 (0.004)	-0.000 (0.000)	0.741 (0.015)	0.925 (0.013)	0.927 (0.077)	0.926 (0.082)	0.926 (0.086)	0.926 (0.084)	0.924 (0.085)

Table S50. Averaged prediction accuracy (R^2) of MolCLR, GEM, and Uni-Mol models for 40 training sets of the APTC-2 data set property ($\Delta\Delta G^{\ddagger}$) prediction. The standard deviations are provided in parentheses.

Data set = APTC-2 (Training sets)<br>y = $\Delta\Delta G^{\ddagger}$(kcal/mol)		R^2
GEM	Global min.	0.384 (0.054)
	Non-aggregation	0.888 (0.037)
Uni-Mol	Global min.	0.419 (0.081)
	Non-aggregation	0.967 (0.024)
MolCLR		0.325 (0.116)

Table S51. Averaged prediction accuracy (R^2) of RF and MIL models for 15 test sets of the PQC data set dipole moment prediction using MOE descriptors. The standard deviations are provided in parentheses.

Data set = PQC (Test sets)<br>Descs = MOE<br>y = Dipole moment (Debye)		RF	MIL				
			Non-aggregation (Instance-Wrapper)	Bag-Wrapper	Instance-Net	Bag-Net	Bag-AttentionNet
		R^2					
Aggregation Method	Boltzmann Weight	0.431 (0.008)	-2.205 (8.957)	0.058 (0.432)	0.192 (0.110)	0.161 (0.152)	0.152 (0.230)
	Mean	0.452 (0.008)	-0.055 (1.174)	0.166 (0.291)	0.108 (0.471)	0.070 (0.542)	-0.362 (1.925)
	Global Minimum	0.422 (0.008)	-17.457 (50.299)	-117.840 (307.917)	-2.094 (5.737)	-7.868 (26.463)	-63.671 (167.065)
	Random	0.420 (0.008)	0.197 (0.081)	0.204 (0.023)	0.208 (0.047)	0.030 (0.472)	0.115 (0.380)
	RMSD max.	0.418 (0.006)	-0.082 (1.116)	-0.159 (1.397)	0.022 (0.559)	-0.215 (1.489)	0.153 (0.150)
	RMSD min.	0.440 (0.008)	0.258 (0.029)	0.259 (0.022)	0.263 (0.020)	0.244 (0.032)	0.269 (0.029)
Non-Aggregation		0.455 (0.006)	0.388 (0.072)	0.300 (0.090)	-0.053 (1.361)	0.157 (0.475)	0.347 (0.064)
Ground-truth		0.498 (0.008)	0.319 (0.156)	-0.403 (2.691)	0.086 (0.827)	0.186 (0.536)	0.240 (0.411)

Table S52. Averaged prediction accuracy (MAE) of RF and MIL models for 15 test sets of the PQC data set dipole moment prediction using MOE descriptors. The standard deviations are provided in parentheses.

Data set = PQC (Test sets)<br>Descs = MOE<br>y = Dipole moment (Debye)		RF	MIL				
			Non-aggregation (Instance-Wrapper)	Bag-Wrapper	Instance-Net	Bag-Net	Bag-AttentionNet
		MAE					
Aggregation Method	Boltzmann Weight	1.336 (0.008)	1.519 (0.029)	1.522 (0.024)	1.513 (0.021)	1.516 (0.020)	1.514 (0.020)
	Mean	1.308 (0.008)	1.451 (0.018)	1.449 (0.022)	1.446 (0.017)	1.457 (0.031)	1.455 (0.021)
	Global Minimum	1.348 (0.009)	1.559 (0.064)	1.617 (0.166)	1.541 (0.032)	1.551 (0.059)	1.591 (0.139)
	Random	1.349 (0.008)	1.538 (0.022)	1.545 (0.024)	1.533 (0.020)	1.545 (0.015)	1.541 (0.027)
	RMSD max.	1.353 (0.008)	1.546 (0.028)	1.554 (0.022)	1.562 (0.026)	1.561 (0.018)	1.548 (0.018)
	RMSD min.	1.325 (0.008)	1.494 (0.018)	1.499 (0.023)	1.497 (0.016)	1.508 (0.017)	1.492 (0.025)
Non-Aggregation		1.300 (0.008)	1.354 (0.011)	1.420 (0.018)	1.433 (0.022)	1.431 (0.016)	1.388 (0.010)
Ground-truth		1.238 (0.008)	1.367 (0.021)	1.356 (0.024)	1.362 (0.023)	1.356 (0.017)	1.357 (0.019)

Table S53. Averaged prediction accuracy (RMSE) of RF and MIL models for 15 test sets of the PQC data set dipole moment prediction using MOE descriptors. The standard deviations are provided in parentheses.

Data set = PQC (Test sets)<br>Descs = MOE<br>y = Dipole moment (Debye)		RF	MIL				
			Non-aggregation (Instance-Wrapper)	Bag-Wrapper	Instance-Net	Bag-Net	Bag-AttentionNet
		RMSE					
Aggregation Method	Boltzmann Weight	1.756 (0.014)	2.965 (3.078)	2.223 (0.418)	2.088 (0.144)	2.125 (0.180)	2.130 (0.263)
	Mean	1.723 (0.013)	2.236 (0.900)	2.102 (0.342)	2.148 (0.499)	2.183 (0.554)	2.420 (1.301)
	Global Minimum	1.770 (0.014)	5.509 (8.666)	12.591 (22.918)	3.282 (2.538)	4.285 (5.648)	9.047 (17.003)
	Random	1.772 (0.014)	2.084 (0.105)	2.076 (0.032)	2.070 (0.066)	2.248 (0.470)	2.161 (0.382)
	RMSD max.	1.775 (0.013)	2.288 (0.841)	2.330 (0.978)	2.252 (0.511)	2.379 (1.016)	2.136 (0.180)
	RMSD min.	1.742 (0.013)	2.004 (0.046)	2.003 (0.031)	1.998 (0.022)	2.023 (0.046)	1.990 (0.039)
Non-Aggregation		1.718 (0.012)	1.818 (0.105)	1.945 (0.126)	2.198 (0.990)	2.087 (0.495)	1.879 (0.097)
Ground-truth		1.649 (0.014)	1.911 (0.197)	2.326 (1.525)	2.106 (0.739)	2.030 (0.551)	1.986 (0.431)

Table S54. Averaged prediction accuracy (R^2) of RF and MIL models for 15 test sets of the PQC data set dipole moment prediction using Pmapper descriptors. The standard deviations are provided in parentheses.

Data set = PQC (Test sets)<br>Descs = Pmapper<br>y = Dipole moment (Debye)		RF	MIL				
			Non-aggregation (Instance-Wrapper)	Bag-Wrapper	Instance-Net	Bag-Net	Bag-AttentionNet
		R^2					
Aggregation Method	Boltzmann Weight	0.311 (0.006)	-0.083 (0.025)	-0.095 (0.019)	-0.082 (0.017)	-0.094 (0.024)	-0.088 (0.024)
	Mean	0.329 (0.006)	-0.009 (0.017)	-0.022 (0.018)	-0.019 (0.025)	-0.012 (0.020)	-0.016 (0.018)
	Global Minimum	0.302 (0.009)	-0.007 (0.003)	-0.007 (0.004)	-0.007 (0.003)	-0.007 (0.004)	-0.007 (0.003)
	Random	0.300 (0.005)	-0.009 (0.003)	-0.009 (0.003)	-0.009 (0.003)	-0.009 (0.003)	-0.009 (0.002)
	RMSD max.	0.295 (0.007)	-0.009 (0.004)	-0.009 (0.004)	-0.009 (0.004)	-0.010 (0.005)	-0.009 (0.004)
	RMSD min.	0.306 (0.010)	-0.008 (0.004)	-0.008 (0.003)	-0.008 (0.003)	-0.008 (0.003)	-0.008 (0.004)
Non-Aggregation		0.352 (0.007)	0.276 (0.007)	-0.017 (0.025)	-0.068 (0.023)	-0.068 (0.032)	0.027 (0.015)
Ground-truth		0.358 (0.007)	-0.008 (0.004)	-0.008 (0.004)	-0.008 (0.004)	-0.008 (0.004)	-0.009 (0.004)

Table S55. Averaged prediction accuracy (MAE) of RF and MIL models for 15 test sets of the PQC data set dipole moment prediction using Pmapper descriptors. The standard deviations are provided in parentheses.

Data set = PQC (Test sets)<br>Descs = Pmapper<br>y = Dipole moment (Debye)		RF	MIL				
			Non-aggregation (Instance-Wrapper)	Bag-Wrapper	Instance-Net	Bag-Net	Bag-AttentionNet
		MAE					
Aggregation Method	Boltzmann Weight	1.488 (0.010)	1.859 (0.017)	1.869 (0.017)	1.856 (0.016)	1.869 (0.012)	1.865 (0.017)
	Mean	1.467 (0.011)	1.790 (0.013)	1.801 (0.016)	1.799 (0.019)	1.791 (0.013)	1.795 (0.011)
	Global Minimum	1.495 (0.012)	1.816 (0.013)	1.817 (0.017)	1.816 (0.013)	1.814 (0.016)	1.816 (0.014)
	Random	1.499 (0.010)	1.817 (0.012)	1.816 (0.015)	1.817 (0.018)	1.817 (0.013)	1.815 (0.012)
	RMSD max.	1.507 (0.011)	1.814 (0.010)	1.821 (0.010)	1.813 (0.013)	1.821 (0.011)	1.819 (0.009)
	RMSD min.	1.489 (0.015)	1.821 (0.017)	1.817 (0.015)	1.814 (0.011)	1.815 (0.014)	1.816 (0.016)
Non-Aggregation		1.431 (0.012)	1.505 (0.010)	1.787 (0.015)	1.829 (0.019)	1.829 (0.025)	1.756 (0.016)
Ground-truth		1.419 (0.010)	1.815 (0.013)	1.814 (0.012)	1.815 (0.016)	1.816 (0.013)	1.818 (0.016)

Table S56. Averaged prediction accuracy (RMSE) of RF and MIL models for 15 test sets of the PQC data set dipole moment prediction using Pmapper descriptors. The standard deviations are provided in parentheses.

Data set = PQC (Test sets)<br>Descs = Pmapper<br>y = Dipole moment (Debye)		RF	MIL				
			Non-aggregation (Instance-Wrapper)	Bag-Wrapper	Instance-Net	Bag-Net	Bag-AttentionNet
		RMSE					
Aggregation Method	Boltzmann Weight	1.932 (0.014)	2.422 (0.022)	2.436 (0.020)	2.420 (0.019)	2.434 (0.014)	2.428 (0.022)
	Mean	1.906 (0.014)	2.338 (0.017)	2.353 (0.019)	2.349 (0.026)	2.341 (0.016)	2.345 (0.017)
	Global Minimum	1.945 (0.016)	2.336 (0.015)	2.336 (0.016)	2.335 (0.015)	2.336 (0.015)	2.336 (0.015)
	Random	1.948 (0.014)	2.338 (0.016)	2.338 (0.015)	2.338 (0.016)	2.338 (0.015)	2.338 (0.015)
	RMSD max.	1.954 (0.016)	2.338 (0.016)	2.338 (0.016)	2.338 (0.016)	2.339 (0.015)	2.338 (0.015)
	RMSD min.	1.939 (0.017)	2.338 (0.016)	2.336 (0.016)	2.337 (0.016)	2.337 (0.016)	2.337 (0.016)
Non-Aggregation		1.873 (0.016)	1.981 (0.013)	2.347 (0.019)	2.406 (0.023)	2.405 (0.033)	2.295 (0.018)
Ground-truth		1.866 (0.015)	2.337 (0.016)	2.337 (0.016)	2.337 (0.017)	2.337 (0.016)	2.338 (0.016)

Table S57. Averaged prediction accuracy (R^2) of RF and MIL models for 15 test sets of the PQC data set dipole moment prediction using 3D-MoRSE descriptors. The standard deviations are provided in parentheses.

Data set = PQC (Test sets)<br>Descs = 3D-MoRSE<br>y = Dipole moment (Debye)		RF	MIL				
			Non-aggregation (Instance-Wrapper)	Bag-Wrapper	Instance-Net	Bag-Net	Bag-AttentionNet
		R^2					
Aggregation Method	Boltzmann Weight	0.330 (0.005)	0.338 (0.014)	0.339 (0.014)	0.340 (0.020)	0.339 (0.014)	0.337 (0.013)
	Mean	0.359 (0.006)	0.371 (0.020)	0.365 (0.017)	0.375 (0.009)	0.362 (0.031)	0.368 (0.018)
	Global Minimum	0.314 (0.006)	0.324 (0.020)	0.323 (0.015)	0.311 (0.022)	0.322 (0.018)	0.319 (0.014)
	Random	0.314 (0.005)	0.329 (0.016)	0.326 (0.014)	0.325 (0.015)	0.324 (0.011)	0.326 (0.014)
	RMSD max.	0.309 (0.005)	0.316 (0.035)	0.319 (0.012)	0.322 (0.013)	0.311 (0.024)	0.302 (0.018)
	RMSD min.	0.316 (0.006)	0.330 (0.018)	0.328 (0.018)	0.331 (0.012)	0.328 (0.018)	0.335 (0.021)
Non-Aggregation		0.338 (0.004)	0.381 (0.008)	0.375 (0.011)	0.370 (0.011)	0.368 (0.011)	0.363 (0.022)
Ground-truth		0.337 (0.007)	0.342 (0.021)	0.346 (0.013)	0.340 (0.020)	0.350 (0.015)	0.346 (0.014)

Table S58. Averaged prediction accuracy (MAE) of RF and MIL models for 15 test sets of the PQC data set dipole moment prediction using 3D-MoRSE descriptors. The standard deviations are provided in parentheses.

Data set = PQC (Test sets)<br>Descs = 3D-MoRSE<br>y = Dipole moment (Debye)		RF	MIL				
			Non-aggregation (Instance-Wrapper)	Bag-Wrapper	Instance-Net	Bag-Net	Bag-AttentionNet
		MAE					
Aggregation Method	Boltzmann Weight	1.457 (0.008)	1.438 (0.015)	1.440 (0.017)	1.438 (0.025)	1.438 (0.018)	1.437 (0.012)
	Mean	1.423 (0.010)	1.402 (0.019)	1.413 (0.022)	1.402 (0.013)	1.416 (0.033)	1.407 (0.017)
	Global Minimum	1.476 (0.009)	1.453 (0.014)	1.453 (0.014)	1.465 (0.026)	1.457 (0.016)	1.455 (0.017)
	Random	1.477 (0.008)	1.448 (0.018)	1.453 (0.014)	1.452 (0.017)	1.453 (0.018)	1.450 (0.016)
	RMSD max.	1.485 (0.009)	1.464 (0.033)	1.457 (0.011)	1.455 (0.016)	1.468 (0.027)	1.477 (0.027)
	RMSD min.	1.475 (0.010)	1.449 (0.021)	1.452 (0.021)	1.449 (0.021)	1.447 (0.020)	1.443 (0.019)
Non-Aggregation		1.445 (0.009)	1.388 (0.016)	1.403 (0.016)	1.407 (0.016)	1.412 (0.017)	1.418 (0.028)
Ground-truth		1.450 (0.009)	1.428 (0.020)	1.424 (0.016)	1.430 (0.019)	1.422 (0.018)	1.424 (0.019)

Table S59. Averaged prediction accuracy (RMSE) of RF and MIL models for 15 test sets of the PQC data set dipole moment prediction using 3D-MoRSE descriptors. The standard deviations are provided in parentheses.

Data set = PQC (Test sets)<br>Descs = 3D-MoRSE<br>y = Dipole moment (Debye)		RF	MIL				
			Non-aggregation (Instance-Wrapper)	Bag-Wrapper	Instance-Net	Bag-Net	Bag-AttentionNet
		RMSE					
Aggregation Method	Boltzmann Weight	1.905 (0.014)	1.893 (0.017)	1.892 (0.023)	1.890 (0.034)	1.893 (0.021)	1.895 (0.017)
	Mean	1.864 (0.014)	1.846 (0.027)	1.855 (0.027)	1.839 (0.012)	1.859 (0.042)	1.850 (0.026)
	Global Minimum	1.928 (0.014)	1.913 (0.020)	1.915 (0.021)	1.932 (0.032)	1.916 (0.018)	1.920 (0.019)
	Random	1.928 (0.013)	1.906 (0.024)	1.911 (0.018)	1.912 (0.024)	1.914 (0.023)	1.911 (0.024)
	RMSD max.	1.935 (0.013)	1.925 (0.044)	1.920 (0.020)	1.916 (0.019)	1.932 (0.033)	1.944 (0.032)
	RMSD min.	1.925 (0.015)	1.905 (0.027)	1.908 (0.025)	1.904 (0.024)	1.908 (0.033)	1.898 (0.025)
Non-Aggregation		1.894 (0.014)	1.831 (0.020)	1.841 (0.018)	1.848 (0.023)	1.850 (0.014)	1.857 (0.027)
Ground-truth		1.896 (0.014)	1.888 (0.033)	1.882 (0.022)	1.890 (0.028)	1.877 (0.027)	1.882 (0.020)

Table S60. Averaged prediction accuracy (R^2) of RF and MIL models for 15 test sets of the PQC data set dipole moment prediction using MBTR descriptors. The standard deviations are provided in parentheses.

Data set = PQC (Test sets)<br>Descs = MBTR<br>y = Dipole moment (Debye)		RF	MIL				
			Non-aggregation (Instance-Wrapper)	Bag-Wrapper	Instance-Net	Bag-Net	Bag-AttentionNet
		R^2					
Aggregation Method	Boltzmann Weight	0.476 (0.006)	-967.244 (2935.009)	-151.254 (565.702)	-258.291 (946.561)	-1495.829 (4368.085)	-94.527 (247.643)
	Mean	0.487 (0.007)	-248.814 (656.738)	-213.557 (481.621)	-91.580 (266.668)	-172.252 (477.967)	-421.058 (1306.752)
	Global Minimum	0.472 (0.007)	-152.225 (391.068)	-246.227 (899.669)	-153.623 (496.951)	-25.019 (74.482)	-387.771 (1226.846)
	Random	0.472 (0.006)	-3015.082 (11330.714)	-3995.323 (13404.226)	-1447.658 (4927.471)	-297.700 (1108.338)	-62.881 (170.514)
	RMSD max.	0.471 (0.007)	-839.681 (3164.810)	-1189.920 (4374.403)	-972.598 (2758.293)	-475.211 (1787.509)	-1571.587 (5280.424)
	RMSD min.	0.484 (0.006)	-7.005 (16.066)	-7.142 (27.439)	-18.533 (64.264)	-6.837 (20.708)	-2.577 (6.405)
Non-Aggregation		0.489 (0.006)	-5.616 (19.659)	-7.192 (21.085)	-9.192 (25.336)	-40.996 (122.941)	-16.247 (61.331)
Ground-truth		0.543 (0.007)	0.464 (0.028)	0.466 (0.039)	0.453 (0.045)	0.468 (0.028)	0.453 (0.030)

Table S61. Averaged prediction accuracy (MAE) of RF and MIL models for 15 test sets of the PQC data set dipole moment prediction using MBTR descriptors. The standard deviations are provided in parentheses.

Data set = PQC (Test sets)<br>Descs = MBTR<br>y = Dipole moment (Debye)		RF	MIL				
			Non-aggregation (Instance-Wrapper)	Bag-Wrapper	Instance-Net	Bag-Net	Bag-AttentionNet
		MAE					
Aggregation Method	Boltzmann Weight	1.254 (0.009)	1.572 (0.502)	1.448 (0.204)	1.478 (0.260)	1.644 (0.608)	1.456 (0.157)
	Mean	1.241 (0.009)	1.458 (0.244)	1.453 (0.224)	1.399 (0.150)	1.431 (0.213)	1.482 (0.330)
	Global Minimum	1.261 (0.008)	1.492 (0.193)	1.483 (0.268)	1.474 (0.206)	1.436 (0.088)	1.536 (0.311)
	Random	1.262 (0.008)	1.687 (0.906)	1.788 (1.014)	1.628 (0.617)	1.493 (0.281)	1.456 (0.124)
	RMSD max.	1.263 (0.008)	1.565 (0.490)	1.595 (0.568)	1.621 (0.493)	1.536 (0.358)	1.644 (0.641)
	RMSD min.	1.244 (0.008)	1.394 (0.049)	1.395 (0.044)	1.399 (0.078)	1.397 (0.044)	1.386 (0.034)
Non-Aggregation		1.231 (0.009)	1.382 (0.045)	1.370 (0.056)	1.363 (0.048)	1.380 (0.105)	1.353 (0.070)
Ground-truth		1.152 (0.009)	1.246 (0.021)	1.241 (0.017)	1.249 (0.016)	1.251 (0.038)	1.256 (0.032)

Table S62. Averaged prediction accuracy (RMSE) of RF and MIL models for 15 test sets of the PQC data set dipole moment prediction using MBTR descriptors. The standard deviations are provided in parentheses.

Data set = PQC (Test sets)<br>Descs = MBTR<br>y = Dipole moment (Debye)		RF	MIL				
			Non-aggregation (Instance-Wrapper)	Bag-Wrapper	Instance-Net	Bag-Net	Bag-AttentionNet
		RMSE					
Aggregation Method	Boltzmann Weight	1.684 (0.013)	28.140 (69.828)	10.756 (27.903)	14.008 (36.338)	38.140 (85.355)	11.029 (20.773)
	Mean	1.668 (0.013)	16.804 (34.035)	16.438 (31.154)	9.916 (20.990)	13.591 (28.745)	20.314 (44.942)
	Global Minimum	1.691 (0.012)	13.210 (26.820)	13.164 (35.776)	11.879 (27.617)	5.873 (10.772)	19.231 (43.614)
	Random	1.691 (0.011)	41.116 (126.744)	55.874 (141.219)	33.626 (85.007)	14.659 (39.163)	9.204 (16.885)
	RMSD max.	1.692 (0.011)	22.471 (66.658)	27.959 (78.725)	31.361 (68.065)	17.564 (49.385)	36.058 (88.994)
	RMSD min.	1.672 (0.012)	4.328 (5.200)	3.636 (5.761)	5.062 (9.292)	4.077 (5.329)	3.304 (3.042)
Non-Aggregation		1.665 (0.011)	3.680 (4.960)	4.097 (5.493)	4.266 (6.384)	6.923 (14.013)	4.536 (8.948)
Ground-truth		1.573 (0.015)	1.703 (0.043)	1.700 (0.053)	1.720 (0.072)	1.696 (0.044)	1.721 (0.049)

Table S63. Averaged prediction accuracy (R^2) of RF and MIL models for 15 test sets of the PQC data set HOMO prediction using MOE descriptors. The standard deviation is provided in parentheses.

Data set = PQC (Test sets)<br>Descs = MOE<br>y = HOMO (eV)		RF	MIL				
			Non-aggregation (Instance-Wrapper)	Bag-Wrapper	Instance-Net	Bag-Net	Bag-AttentionNet
		R^2					
Aggregation Method	Boltzmann Weight	0.712 (0.003)	0.663 (0.036)	0.665 (0.019)	0.659 (0.028)	0.638 (0.043)	0.657 (0.024)
	Mean	0.745 (0.002)	0.688 (0.022)	0.698 (0.021)	0.689 (0.026)	0.685 (0.017)	0.675 (0.051)
	Global Minimum	0.700 (0.003)	0.312 (1.343)	-8.362 (34.909)	0.570 (0.179)	-8.260 (34.440)	-1.300 (7.051)
	Random	0.706 (0.004)	0.621 (0.135)	0.643 (0.050)	0.618 (0.110)	0.638 (0.055)	0.591 (0.223)
	RMSD max.	0.690 (0.003)	0.624 (0.032)	0.647 (0.014)	0.641 (0.022)	0.639 (0.021)	0.643 (0.023)
	RMSD min.	0.718 (0.003)	0.670 (0.015)	0.657 (0.029)	0.644 (0.042)	0.662 (0.034)	0.674 (0.018)
Non-Aggregation		0.761 (0.003)	0.737 (0.012)	0.663 (0.055)	0.686 (0.045)	0.683 (0.049)	0.686 (0.035)
Ground-truth		0.672 (0.004)	0.564 (0.130)	0.621 (0.030)	0.617 (0.053)	0.589 (0.096)	0.581 (0.160)

Table S64. Averaged prediction accuracy (MAE) of RF and MIL models for 15 test sets of the PQC data set HOMO prediction using MOE descriptors. The standard deviation is provided in parentheses.

Data set = PQC (Test sets) Descs = MOE y = HOMO (eV)		RF	MIL				
			Non-aggregation (Instance-Wrapper)	Bag-Wrapper	Instance-Net	Bag-Net	Bag-AttentionNet
		MAE					
Aggregation Method	Boltzmann Weight	0.222 (0.002)	0.244 (0.008)	0.245 (0.007)	0.247 (0.010)	0.256 (0.017)	0.248 (0.008)
	Mean	0.208 (0.001)	0.236 (0.009)	0.233 (0.008)	0.236 (0.009)	0.237 (0.008)	0.241 (0.022)
	Global Minimum	0.227 (0.001)	0.249 (0.006)	0.253 (0.012)	0.258 (0.014)	0.260 (0.025)	0.252 (0.010)
	Random	0.225 (0.001)	0.249 (0.006)	0.247 (0.004)	0.254 (0.013)	0.251 (0.010)	0.253 (0.013)
	RMSD max.	0.231 (0.001)	0.262 (0.012)	0.254 (0.005)	0.256 (0.009)	0.256 (0.008)	0.254 (0.008)
	RMSD min.	0.220 (0.001)	0.244 (0.006)	0.250 (0.012)	0.254 (0.016)	0.247 (0.011)	0.243 (0.007)
Non-Aggregation		0.201 (0.001)	0.218 (0.005)	0.247 (0.022)	0.238 (0.018)	0.239 (0.019)	0.238 (0.013)
Ground-truth		0.234 (0.001)	0.264 (0.017)	0.258 (0.012)	0.255 (0.006)	0.265 (0.025)	0.257 (0.008)

Table S65. Averaged prediction accuracy (RMSE) of RF and MIL models for 15 test sets of the PQC data set HOMO prediction using MOE descriptors. The standard deviation is provided in parentheses.

Data set = PQC (Test sets)<br>Descs = MOE<br>y = HOMO (eV)		RF	MIL				
			Non-aggregation (Instance-Wrapper)	Bag-Wrapper	Instance-Net	Bag-Net	Bag-AttentionNet
		RMSE					
Aggregation Method	Boltzmann Weight	0.296 (0.003)	0.320 (0.016)	0.320 (0.010)	0.322 (0.013)	0.332 (0.019)	0.323 (0.011)
	Mean	0.279 (0.002)	0.308 (0.010)	0.303 (0.011)	0.308 (0.013)	0.310 (0.009)	0.314 (0.024)
	Global Minimum	0.303 (0.002)	0.388 (0.252)	0.733 (1.575)	0.357 (0.065)	0.737 (1.562)	0.531 (0.670)
	Random	0.300 (0.002)	0.336 (0.048)	0.329 (0.021)	0.339 (0.041)	0.332 (0.022)	0.346 (0.072)
	RMSD max.	0.307 (0.002)	0.338 (0.015)	0.328 (0.006)	0.331 (0.011)	0.332 (0.010)	0.330 (0.010)
	RMSD min.	0.294 (0.002)	0.317 (0.007)	0.323 (0.014)	0.329 (0.019)	0.321 (0.016)	0.315 (0.008)
Non-Aggregation		0.270 (0.002)	0.283 (0.007)	0.320 (0.025)	0.309 (0.021)	0.310 (0.023)	0.309 (0.016)
Ground-truth		0.316 (0.002)	0.362 (0.049)	0.340 (0.013)	0.341 (0.022)	0.352 (0.038)	0.353 (0.055)

Table S66. Averaged prediction accuracy (R^2) of RF and MIL models for 15 test sets of the PQC data set HOMO prediction using Pmapper descriptors. The standard deviations are provided in parentheses.

Data set = PQC (Test sets)<br>Descs = Pmapper<br>y = HOMO (eV)		RF	MIL				
			Non-aggregation (Instance-Wrapper)	Bag-Wrapper	Instance-Net	Bag-Net	Bag-AttentionNet
		R^2					
Aggregation Method	Boltzmann Weight	0.529 (0.006)	0.282 (0.031)	0.280 (0.033)	0.258 (0.061)	0.284 (0.030)	0.280 (0.018)
	Mean	0.561 (0.005)	0.398 (0.028)	0.403 (0.024)	0.400 (0.031)	0.405 (0.024)	0.384 (0.035)
	Global Minimum	0.513 (0.007)	-0.009 (0.004)	-0.007 (0.004)	-0.007 (0.002)	-0.008 (0.004)	-0.008 (0.003)
	Random	0.508 (0.005)	-0.009 (0.004)	-0.008 (0.003)	-0.009 (0.004)	-0.009 (0.003)	-0.008 (0.003)
	RMSD max.	0.487 (0.007)	-0.007 (0.003)	-0.007 (0.003)	-0.009 (0.003)	-0.007 (0.003)	-0.008 (0.004)
	RMSD min.	0.523 (0.006)	-0.008 (0.004)	-0.007 (0.003)	-0.008 (0.004)	-0.008 (0.004)	-0.008 (0.003)
Non-Aggregation		0.676 (0.114)	0.632 (0.018)	0.456 (0.024)	0.456 (0.026)	0.444 (0.035)	0.545 (0.022)
Ground-truth		0.535 (0.006)	-0.006 (0.004)	-0.005 (0.002)	-0.006 (0.003)	-0.004 (0.003)	-0.006 (0.003)

Table S67. Averaged prediction accuracy (MAE) of RF and MIL models for 15 test sets of the PQC data set HOMO prediction using Pmapper descriptors. The standard deviations are provided in parentheses.

Data set = PQC (Test sets)<br>Descs = Pmapper<br>y = HOMO (eV)		RF	MIL				
			Non-aggregation (Instance-Wrapper)	Bag-Wrapper	Instance-Net	Bag-Net	Bag-AttentionNet
		MAE					
Aggregation Method	Boltzmann Weight	0.283 (0.002)	0.356 (0.007)	0.358 (0.010)	0.363 (0.017)	0.357 (0.008)	0.357 (0.006)
	Mean	0.270 (0.002)	0.323 (0.008)	0.320 (0.007)	0.321 (0.010)	0.320 (0.005)	0.327 (0.010)
	Global Minimum	0.287 (0.002)	0.434 (0.004)	0.432 (0.004)	0.433 (0.003)	0.432 (0.004)	0.433 (0.003)
	Random	0.289 (0.001)	0.432 (0.004)	0.433 (0.004)	0.433 (0.004)	0.432 (0.004)	0.433 (0.004)
	RMSD max.	0.296 (0.002)	0.432 (0.003)	0.432 (0.003)	0.432 (0.004)	0.433 (0.003)	0.431 (0.003)
	RMSD min.	0.283 (0.002)	0.433 (0.004)	0.432 (0.003)	0.434 (0.004)	0.433 (0.004)	0.432 (0.003)
Non-Aggregation		0.273 (0.024)	0.247 (0.007)	0.306 (0.008)	0.306 (0.007)	0.311 (0.010)	0.279 (0.008)
Ground-truth		0.276 (0.002)	0.433 (0.003)	0.432 (0.003)	0.433 (0.004)	0.432 (0.003)	0.433 (0.004)

Table S68. Averaged prediction accuracy (RMSE) of RF and MIL models for 15 test sets of the PQC data set HOMO prediction using Pmapper descriptors. The standard deviations are provided in parentheses.

Data set = PQC (Test sets)<br>Descs = Pmapper<br>y = HOMO (eV)		RF	MIL				
			Non-aggregation (Instance-Wrapper)	Bag-Wrapper	Instance-Net	Bag-Net	Bag-AttentionNet
		RMSE					
Aggregation Method	Boltzmann Weight	0.379 (0.003)	0.468 (0.010)	0.468 (0.011)	0.475 (0.017)	0.467 (0.010)	0.469 (0.008)
	Mean	0.366 (0.003)	0.428 (0.009)	0.427 (0.009)	0.428 (0.011)	0.426 (0.008)	0.433 (0.011)
	Global Minimum	0.385 (0.003)	0.555 (0.003)	0.554 (0.003)	0.554 (0.003)	0.554 (0.003)	0.554 (0.003)
	Random	0.387 (0.003)	0.555 (0.003)	0.555 (0.003)	0.555 (0.003)	0.555 (0.003)	0.555 (0.003)
	RMSD max.	0.395 (0.004)	0.554 (0.003)	0.554 (0.003)	0.554 (0.003)	0.554 (0.003)	0.554 (0.003)
	RMSD min.	0.381 (0.003)	0.554 (0.003)	0.554 (0.003)	0.554 (0.003)	0.554 (0.003)	0.554 (0.003)
Non-Aggregation		0.380 (0.044)	0.335 (0.009)	0.407 (0.008)	0.407 (0.009)	0.412 (0.012)	0.372 (0.009)
Ground-truth		0.377 (0.004)	0.554 (0.003)	0.554 (0.003)	0.554 (0.003)	0.553 (0.003)	0.554 (0.003)

Table S69. Averaged prediction accuracy (R^2) of RF and MIL models for 15 test sets of the PQC data set HOMO prediction using 3D-MoRSE descriptors. The standard deviations are provided in parentheses.

Data set = PQC (Test sets)<br>Descs = 3D-MoRSE<br>y = HOMO (eV)		RF	MIL				
			Non-aggregation (Instance-Wrapper)	Bag-Wrapper	Instance-Net	Bag-Net	Bag-AttentionNet
		R^2					
Aggregation Method	Boltzmann Weight	0.649 (0.003)	0.705 (0.027)	0.703 (0.025)	0.703 (0.029)	0.696 (0.042)	0.689 (0.047)
	Mean	0.696 (0.003)	0.716 (0.047)	0.724 (0.024)	0.743 (0.006)	0.727 (0.024)	0.715 (0.038)
	Global Minimum	0.618 (0.003)	0.679 (0.040)	0.690 (0.020)	0.690 (0.024)	0.686 (0.035)	0.698 (0.018)
	Random	0.628 (0.003)	0.700 (0.019)	0.670 (0.062)	0.691 (0.046)	0.699 (0.021)	0.700 (0.019)
	RMSD max.	0.615 (0.004)	0.688 (0.036)	0.693 (0.026)	0.696 (0.013)	0.694 (0.014)	0.688 (0.024)
	RMSD min.	0.635 (0.004)	0.700 (0.017)	0.689 (0.048)	0.695 (0.026)	0.689 (0.024)	0.691 (0.045)
Non-Aggregation		0.679 (0.003)	0.762 (0.025)	0.731 (0.013)	0.726 (0.023)	0.719 (0.040)	0.710 (0.054)
Ground-truth		0.632 (0.003)	0.698 (0.027)	0.711 (0.019)	0.713 (0.009)	0.694 (0.030)	0.700 (0.025)

Table S70. Averaged prediction accuracy (MAE) of RF and MIL models for 15 test sets of the PQC data set HOMO prediction using 3D-MoRSE descriptors. The standard deviations are provided in parentheses.

Data set = PQC (Test sets)<br>Descs = 3D-MoRSE<br>y = HOMO (eV)		RF	MIL				
			Non-aggregation (Instance-Wrapper)	Bag-Wrapper	Instance-Net	Bag-Net	Bag-AttentionNet
		MAE					
Aggregation Method	Boltzmann Weight	0.249 (0.002)	0.234 (0.013)	0.235 (0.012)	0.236 (0.011)	0.237 (0.017)	0.241 (0.020)
	Mean	0.231 (0.002)	0.230 (0.021)	0.226 (0.011)	0.217 (0.003)	0.224 (0.010)	0.229 (0.015)
	Global Minimum	0.260 (0.002)	0.245 (0.019)	0.240 (0.008)	0.240 (0.011)	0.242 (0.015)	0.236 (0.009)
	Random	0.257 (0.002)	0.237 (0.009)	0.249 (0.028)	0.239 (0.020)	0.236 (0.009)	0.236 (0.009)
	RMSD max.	0.262 (0.001)	0.240 (0.015)	0.238 (0.012)	0.237 (0.006)	0.239 (0.007)	0.242 (0.011)
	RMSD min.	0.255 (0.002)	0.236 (0.008)	0.240 (0.021)	0.237 (0.012)	0.241 (0.011)	0.240 (0.017)
Non-Aggregation		0.239 (0.001)	0.209 (0.013)	0.223 (0.007)	0.225 (0.011)	0.227 (0.017)	0.231 (0.023)
Ground-truth		0.255 (0.001)	0.235 (0.010)	0.231 (0.008)	0.230 (0.004)	0.239 (0.013)	0.236 (0.011)

Table S71. Averaged prediction accuracy (RMSE) of RF and MIL models for 15 test sets of the PQC data set HOMO prediction using 3D-MoRSE descriptors. The standard deviations are provided in parentheses.

Data set = PQC (Test sets)<br>Descs = 3D-MoRSE<br>y = HOMO (eV)		RF	MIL				
			Non-aggregation (Instance-Wrapper)	Bag-Wrapper	Instance-Net	Bag-Net	Bag-AttentionNet
		RMSE					
Aggregation Method	Boltzmann Weight	0.327 (0.002)	0.300 (0.013)	0.301 (0.012)	0.301 (0.013)	0.304 (0.020)	0.307 (0.023)
	Mean	0.305 (0.003)	0.293 (0.023)	0.290 (0.012)	0.280 (0.003)	0.289 (0.013)	0.294 (0.018)
	Global Minimum	0.341 (0.003)	0.312 (0.019)	0.307 (0.010)	0.307 (0.012)	0.309 (0.017)	0.303 (0.009)
	Random	0.337 (0.002)	0.302 (0.009)	0.316 (0.030)	0.307 (0.022)	0.303 (0.011)	0.302 (0.010)
	RMSD max.	0.343 (0.002)	0.308 (0.017)	0.306 (0.013)	0.304 (0.007)	0.306 (0.008)	0.308 (0.011)
	RMSD min.	0.334 (0.002)	0.302 (0.008)	0.307 (0.022)	0.305 (0.012)	0.308 (0.012)	0.307 (0.021)
Non-Aggregation		0.313 (0.001)	0.269 (0.014)	0.286 (0.007)	0.289 (0.012)	0.292 (0.020)	0.296 (0.026)
Ground-truth		0.335 (0.002)	0.303 (0.013)	0.297 (0.010)	0.296 (0.004)	0.305 (0.015)	0.302 (0.013)

Table S72. Averaged prediction accuracy (R^2) of RF and MIL models for 15 test sets of the PQC data set HOMO prediction using MBTR descriptors. The standard deviations are provided in parentheses.

Data set = PQC (Test sets)<br>Descs = MBTR<br>y = HOMO (eV)		RF	MIL				
			Non-aggregation (Instance-Wrapper)	Bag-Wrapper	Instance-Net	Bag-Net	Bag-AttentionNet
		R^2					
Aggregation Method	Boltzmann Weight	0.801 (0.002)	-1509.789 (3648.703)	-1248.212 (3317.400)	-1243.084 (3132.129)	-2597.045 (6662.414)	-2194.822 (5246.144)
	Mean	0.814 (0.002)	-1281.972 (3484.445)	-3751.742 (9559.496)	-2244.866 (4733.724)	-2718.771 (6128.406)	-4161.590 (10304.899)
	Global Minimum	0.795 (0.002)	-1188.059 (2465.041)	-1394.246 (2916.974)	-1267.542 (2970.886)	-999.149 (2833.460)	-1116.902 (2410.818)
	Random	0.795 (0.002)	-9762.095 (23211.719)	-2925.715 (7832.810)	-5419.192 (13563.001)	-3440.441 (10744.738)	-4529.094 (14321.403)
	RMSD max.	0.790 (0.002)	-1381.292 (2945.268)	-1510.987 (3135.444)	-1405.415 (2999.537)	-1757.872 (4365.383)	-2106.850 (4798.486)
	RMSD min.	0.798 (0.003)	-10.136 (28.900)	-14.159 (34.241)	-2.824 (11.342)	-24.163 (54.677)	-18.613 (49.881)
Non-Aggregation		0.822 (0.007)	-110.670 (253.526)	-33.265 (73.993)	-20.978 (48.405)	-6.147 (15.690)	-12.609 (35.352)
Ground-truth		0.809 (0.008)	0.824 (0.029)	0.823 (0.042)	0.834 (0.020)	0.815 (0.021)	0.819 (0.034)

Table S73. Averaged prediction accuracy (MAE) of RF and MIL models for 15 test sets of the PQC data set HOMO prediction using MBTR descriptors. The standard deviations are provided in parentheses.

Data set = PQC (Test sets)<br>Descs = MBTR<br>y = HOMO (eV)		RF	MIL				
			Non-aggregation (Instance-Wrapper)	Bag-Wrapper	Instance-Net	Bag-Net	Bag-AttentionNet
		MAE					
Aggregation Method	Boltzmann Weight	0.182 (0.001)	0.251 (0.142)	0.241 (0.137)	0.243 (0.132)	0.275 (0.189)	0.262 (0.180)
	Mean	0.175 (0.001)	0.235 (0.133)	0.288 (0.231)	0.268 (0.184)	0.268 (0.193)	0.289 (0.235)
	Global Minimum	0.186 (0.001)	0.249 (0.130)	0.252 (0.141)	0.247 (0.132)	0.233 (0.120)	0.245 (0.124)
	Random	0.185 (0.001)	0.352 (0.362)	0.272 (0.211)	0.310 (0.273)	0.278 (0.224)	0.295 (0.252)
	RMSD max.	0.188 (0.001)	0.255 (0.136)	0.263 (0.150)	0.257 (0.138)	0.258 (0.154)	0.268 (0.169)
	RMSD min.	0.183 (0.001)	0.198 (0.022)	0.186 (0.015)	0.188 (0.012)	0.191 (0.017)	0.194 (0.023)
Non-Aggregation		0.168 (0.004)	0.185 (0.041)	0.192 (0.022)	0.188 (0.027)	0.186 (0.015)	0.189 (0.017)
Ground-truth		0.175 (0.004)	0.177 (0.015)	0.177 (0.024)	0.172 (0.011)	0.183 (0.014)	0.179 (0.018)

Table S74. Averaged prediction accuracy (RMSE) of RF and MIL models for 15 test sets of the PQC data set HOMO prediction using MBTR descriptors. The standard deviations are provided in parentheses.

Data set = PQC (Test sets)<br>Descs = MBTR<br>y = HOMO (eV)		RF	MIL				
			Non-aggregation (Instance-Wrapper)	Bag-Wrapper	Instance-Net	Bag-Net	Bag-AttentionNet
		RMSE					
Aggregation Method	Boltzmann Weight	0.246 (0.001)	9.415 (20.039)	7.726 (18.644)	8.245 (18.309)	11.882 (26.478)	11.231 (24.183)
	Mean	0.238 (0.001)	8.391 (18.619)	14.630 (31.677)	11.876 (24.226)	12.862 (26.728)	15.499 (33.333)
	Global Minimum	0.250 (0.001)	8.729 (17.576)	9.421 (19.059)	8.785 (18.280)	6.955 (16.595)	8.396 (17.072)
	Random	0.250 (0.001)	23.870 (50.877)	12.089 (28.377)	17.039 (38.321)	12.916 (30.862)	14.668 (35.481)
	RMSD max.	0.253 (0.001)	9.345 (18.995)	9.821 (19.831)	9.427 (19.150)	10.154 (21.616)	11.303 (23.584)
	RMSD min.	0.248 (0.002)	0.968 (1.631)	1.120 (1.905)	0.586 (0.943)	1.414 (2.475)	1.171 (2.229)
Non-Aggregation		0.233 (0.005)	2.723 (5.366)	1.619 (2.908)	1.322 (2.311)	0.829 (1.268)	1.048 (1.815)
Ground-truth		0.241 (0.005)	0.231 (0.018)	0.231 (0.024)	0.225 (0.013)	0.237 (0.014)	0.234 (0.021)

Table S75. Averaged prediction accuracy (R^2) of RF and MIL models for 15 test sets of the PQC data set HOMO-LUMO gap prediction using MOE descriptors. The standard deviations are provided in parentheses.

Data set = PQC (Test sets)<br>Descs = MOE<br>y = HOMO-LUMO gap (eV)		RF	MIL				
			Non-aggregation (Instance-Wrapper)	Bag-Wrapper	Instance-Net	Bag-Net	Bag-AttentionNet
		R^2					
Aggregation Method	Boltzmann Weight	0.856 (0.002)	0.842 (0.007)	0.832 (0.017)	0.839 (0.010)	0.822 (0.049)	0.837 (0.014)
	Mean	0.869 (0.002)	0.813 (0.162)	0.829 (0.065)	0.809 (0.097)	0.797 (0.140)	0.845 (0.029)
	Global Minimum	0.848 (0.005)	-5.603 (24.904)	-46.140 (181.919)	-38.390 (146.164)	0.818 (0.029)	-104.196 (406.736)
	Random	0.848 (0.003)	0.593 (0.707)	0.346 (1.621)	0.562 (0.874)	0.744 (0.242)	-0.374 (3.624)
	RMSD max.	0.843 (0.002)	0.829 (0.009)	0.828 (0.007)	0.820 (0.015)	0.825 (0.020)	0.828 (0.016)
	RMSD min.	0.850 (0.003)	0.828 (0.016)	0.831 (0.016)	0.834 (0.012)	0.831 (0.028)	0.837 (0.011)
Non-Aggregation		0.880 (0.002)	0.872 (0.029)	0.851 (0.009)	0.851 (0.014)	0.851 (0.013)	0.855 (0.014)
Ground-truth		0.865 (0.002)	0.831 (0.028)	0.823 (0.058)	0.820 (0.083)	0.800 (0.153)	0.813 (0.070)

Table S76. Averaged prediction accuracy (MAE) of RF and MIL models for 15 test sets of the PQC data set HOMO-LUMO gap prediction using MOE descriptors. The standard deviations are provided in parentheses.

Data set = PQC (Test sets)<br>Descs = MOE<br>y = HOMO-LUMO gap (eV)		RF	MIL				
			Non-aggregation (Instance-Wrapper)	Bag-Wrapper	Instance-Net	Bag-Net	Bag-AttentionNet
		MAE					
Aggregation Method	Boltzmann Weight	0.300 (0.002)	0.323 (0.007)	0.331 (0.012)	0.327 (0.010)	0.330 (0.012)	0.328 (0.015)
	Mean	0.283 (0.002)	0.307 (0.008)	0.310 (0.007)	0.321 (0.020)	0.316 (0.014)	0.312 (0.024)
	Global Minimum	0.310 (0.006)	0.343 (0.021)	0.348 (0.051)	0.350 (0.050)	0.345 (0.023)	0.365 (0.078)
	Random	0.310 (0.002)	0.336 (0.011)	0.341 (0.018)	0.336 (0.016)	0.334 (0.015)	0.335 (0.011)
	RMSD max.	0.315 (0.002)	0.335 (0.009)	0.338 (0.008)	0.348 (0.018)	0.341 (0.020)	0.338 (0.018)
	RMSD min.	0.307 (0.003)	0.340 (0.018)	0.335 (0.018)	0.331 (0.013)	0.337 (0.032)	0.330 (0.012)
Non-Aggregation		0.272 (0.002)	0.278 (0.005)	0.315 (0.011)	0.309 (0.010)	0.311 (0.012)	0.310 (0.019)
Ground-truth		0.288 (0.002)	0.328 (0.012)	0.327 (0.013)	0.324 (0.006)	0.325 (0.009)	0.332 (0.012)

Table S77. Averaged prediction accuracy (RMSE) of RF and MIL models for 15 test sets of the PQC data set HOMO-LUMO gap prediction using MOE descriptors. The standard deviations are provided in parentheses.

Data set = PQC (Test sets)<br>Descs = MOE<br>y = HOMO-LUMO gap (eV)		RF	MIL				
			Non-aggregation (Instance-Wrapper)	Bag-Wrapper	Instance-Net	Bag-Net	Bag-AttentionNet
		RMSE					
Aggregation Method	Boltzmann Weight	0.406 (0.003)	0.426 (0.010)	0.438 (0.021)	0.430 (0.014)	0.448 (0.052)	0.432 (0.018)
	Mean	0.387 (0.003)	0.443 (0.136)	0.437 (0.071)	0.458 (0.097)	0.466 (0.128)	0.420 (0.037)
	Global Minimum	0.417 (0.007)	1.113 (2.587)	2.295 (7.183)	2.399 (6.454)	0.456 (0.033)	3.238 (10.793)
	Random	0.418 (0.003)	0.576 (0.377)	0.639 (0.599)	0.581 (0.415)	0.508 (0.191)	0.827 (0.969)
	RMSD max.	0.424 (0.003)	0.443 (0.013)	0.444 (0.010)	0.454 (0.020)	0.448 (0.025)	0.443 (0.019)
	RMSD min.	0.415 (0.004)	0.444 (0.020)	0.440 (0.021)	0.436 (0.015)	0.439 (0.032)	0.432 (0.014)
Non-Aggregation		0.372 (0.003)	0.381 (0.039)	0.413 (0.012)	0.413 (0.018)	0.413 (0.018)	0.408 (0.019)
Ground-truth		0.394 (0.003)	0.440 (0.033)	0.447 (0.061)	0.448 (0.081)	0.462 (0.129)	0.458 (0.070)

Table S78. Averaged prediction accuracy (R^2) of RF and MIL models for 15 test sets of the PQC data set HOMO-LUMO gap prediction using Pmapper descriptors. The standard deviations are provided in parentheses.

Data set = PQC (Test sets)<br>Descs = Pmapper<br>y = HOMO-LUMO gap (eV)		RF	MIL				
			Non-aggregation (Instance-Wrapper)	Bag-Wrapper	Instance-Net	Bag-Net	Bag-AttentionNet
		R^2					
Aggregation Method	Boltzmann Weight	0.803 (0.004)	0.701 (0.009)	0.698 (0.011)	0.704 (0.006)	0.704 (0.008)	0.697 (0.009)
	Mean	0.817 (0.004)	0.745 (0.015)	0.745 (0.007)	0.743 (0.020)	0.748 (0.007)	0.750 (0.011)
	Global Minimum	0.794 (0.004)	-0.003 (0.003)	-0.004 (0.005)	-0.004 (0.004)	-0.003 (0.004)	-0.003 (0.002)
	Random	0.785 (0.011)	-0.004 (0.007)	-0.003 (0.005)	-0.006 (0.007)	-0.004 (0.004)	-0.008 (0.009)
	RMSD max.	0.785 (0.004)	-0.003 (0.005)	-0.005 (0.007)	-0.006 (0.009)	-0.007 (0.006)	-0.004 (0.006)
	RMSD min.	0.795 (0.003)	-0.005 (0.004)	-0.005 (0.006)	-0.004 (0.004)	-0.004 (0.003)	-0.004 (0.003)
Non-Aggregation		0.829 (0.010)	0.846 (0.004)	0.769 (0.009)	0.769 (0.009)	0.760 (0.012)	0.810 (0.006)
Ground-truth		0.813 (0.005)	-0.007 (0.008)	-0.006 (0.006)	-0.003 (0.004)	-0.004 (0.006)	-0.006 (0.006)

Table S79. Averaged prediction accuracy (MAE) of RF and MIL models for 15 test sets of the PQC data set HOMO-LUMO gap prediction using Pmapper descriptors. The standard deviations are provided in parentheses.

Data set = PQC (Test sets)<br>Descs = Pmapper<br>y = HOMO-LUMO gap (eV)		RF	MIL				
			Non-aggregation (Instance-Wrapper)	Bag-Wrapper	Instance-Net	Bag-Net	Bag-AttentionNet
		MAE					
Aggregation Method	Boltzmann Weight	0.344 (0.002)	0.439 (0.007)	0.441 (0.009)	0.437 (0.003)	0.436 (0.008)	0.441 (0.007)
	Mean	0.328 (0.002)	0.400 (0.011)	0.401 (0.007)	0.401 (0.015)	0.398 (0.007)	0.397 (0.010)
	Global Minimum	0.351 (0.002)	0.811 (0.011)	0.814 (0.014)	0.813 (0.009)	0.812 (0.010)	0.811 (0.009)
	Random	0.360 (0.009)	0.813 (0.013)	0.813 (0.010)	0.814 (0.015)	0.815 (0.008)	0.815 (0.017)
	RMSD max.	0.361 (0.002)	0.817 (0.012)	0.806 (0.009)	0.818 (0.018)	0.817 (0.014)	0.811 (0.012)
	RMSD min.	0.351 (0.002)	0.811 (0.009)	0.814 (0.012)	0.810 (0.008)	0.812 (0.011)	0.811 (0.006)
Non-Aggregation		0.309 (0.011)	0.300 (0.004)	0.381 (0.008)	0.390 (0.015)	0.390 (0.012)	0.342 (0.008)
Ground-truth		0.328 (0.003)	0.812 (0.017)	0.815 (0.014)	0.813 (0.011)	0.813 (0.011)	0.815 (0.011)

Table S80. Averaged prediction accuracy (RMSE) of RF and MIL models for 15 test sets of the PQC data set HOMO-LUMO gap prediction using Pmapper descriptors. The standard deviations are provided in parentheses.

Data set = PQC (Test sets)<br>Descs = Pmapper<br>y = HOMO-LUMO gap (eV)		RF	MIL				
			Non-aggregation (Instance-Wrapper)	Bag-Wrapper	Instance-Net	Bag-Net	Bag-AttentionNet
		RMSE					
Aggregation Method	Boltzmann Weight	0.475 (0.005)	0.586 (0.008)	0.588 (0.012)	0.583 (0.005)	0.582 (0.008)	0.589 (0.009)
	Mean	0.458 (0.005)	0.541 (0.013)	0.541 (0.008)	0.542 (0.019)	0.538 (0.008)	0.536 (0.011)
	Global Minimum	0.486 (0.004)	1.073 (0.006)	1.073 (0.008)	1.073 (0.007)	1.073 (0.007)	1.072 (0.006)
	Random	0.496 (0.012)	1.073 (0.006)	1.073 (0.006)	1.074 (0.006)	1.073 (0.005)	1.075 (0.006)
	RMSD max.	0.496 (0.004)	1.073 (0.007)	1.074 (0.007)	1.074 (0.007)	1.075 (0.005)	1.073 (0.005)
	RMSD min.	0.485 (0.004)	1.074 (0.005)	1.074 (0.006)	1.073 (0.006)	1.073 (0.006)	1.073 (0.006)
Non-Aggregation		0.442 (0.012)	0.420 (0.006)	0.514 (0.010)	0.526 (0.016)	0.525 (0.013)	0.467 (0.007)
Ground-truth		0.463 (0.006)	1.075 (0.008)	1.074 (0.006)	1.073 (0.006)	1.073 (0.006)	1.074 (0.005)

Table S81. Averaged prediction accuracy (R^2) of RF and MIL models for 15 test sets of the PQC data set HOMO-LUMO gap prediction using 3D-MoRSE descriptors. The standard deviations are provided in parentheses.

Data set = PQC (Test sets)<br>Descs = 3D-MoRSE<br>y = HOMO-LUMO gap (eV)		RF	MIL				
			Non-aggregation (Instance-Wrapper)	Bag-Wrapper	Instance-Net	Bag-Net	Bag-AttentionNet
		R^2					
Aggregation Method	Boltzmann Weight	0.837 (0.002)	0.857 (0.011)	0.860 (0.012)	0.855 (0.016)	0.859 (0.012)	0.855 (0.011)
	Mean	0.858 (0.002)	0.873 (0.008)	0.872 (0.012)	0.874 (0.014)	0.871 (0.013)	0.867 (0.015)
	Global Minimum	0.824 (0.003)	0.857 (0.004)	0.851 (0.014)	0.853 (0.009)	0.856 (0.008)	0.848 (0.015)
	Random	0.824 (0.003)	0.851 (0.012)	0.852 (0.012)	0.855 (0.006)	0.856 (0.007)	0.847 (0.016)
	RMSD max.	0.817 (0.003)	0.845 (0.020)	0.854 (0.008)	0.843 (0.021)	0.849 (0.015)	0.849 (0.014)
	RMSD min.	0.829 (0.002)	0.852 (0.012)	0.854 (0.009)	0.853 (0.016)	0.855 (0.017)	0.857 (0.010)
Non-Aggregation		0.849 (0.002)	0.896 (0.006)	0.872 (0.006)	0.871 (0.005)	0.871 (0.009)	0.875 (0.005)
Ground-truth		0.824 (0.003)	0.855 (0.011)	0.856 (0.008)	0.852 (0.014)	0.855 (0.012)	0.851 (0.009)

Table S82. Averaged prediction accuracy (MAE) of RF and MIL models for 15 test sets of the PQC data set HOMO-LUMO gap prediction using 3D-MoRSE descriptors. The standard deviations are provided in parentheses.

Data set = PQC (Test sets)<br>Descs = 3D-MoRSE<br>y = HOMO-LUMO gap (eV)		RF	MIL				
			Non-aggregation (Instance-Wrapper)	Bag-Wrapper	Instance-Net	Bag-Net	Bag-AttentionNet
		MAE					
Aggregation Method	Boltzmann Weight	0.324 (0.002)	0.312 (0.014)	0.309 (0.017)	0.314 (0.016)	0.310 (0.014)	0.314 (0.014)
	Mean	0.300 (0.002)	0.292 (0.008)	0.293 (0.013)	0.293 (0.020)	0.295 (0.017)	0.300 (0.021)
	Global Minimum	0.338 (0.003)	0.311 (0.004)	0.320 (0.019)	0.316 (0.011)	0.312 (0.009)	0.322 (0.019)
	Random	0.338 (0.003)	0.320 (0.014)	0.321 (0.017)	0.313 (0.007)	0.315 (0.011)	0.324 (0.021)
	RMSD max.	0.344 (0.002)	0.325 (0.024)	0.313 (0.010)	0.329 (0.027)	0.321 (0.020)	0.321 (0.020)
	RMSD min.	0.334 (0.003)	0.318 (0.017)	0.315 (0.011)	0.317 (0.021)	0.315 (0.021)	0.311 (0.011)
Non-Aggregation		0.313 (0.002)	0.263 (0.010)	0.294 (0.008)	0.295 (0.007)	0.294 (0.012)	0.288 (0.006)
Ground-truth		0.337 (0.003)	0.315 (0.014)	0.312 (0.009)	0.319 (0.020)	0.315 (0.017)	0.319 (0.014)

Table S83. Averaged prediction accuracy (RMSE) of RF and MIL models for 15 test sets of the PQC data set HOMO-LUMO gap prediction using 3D-MoRSE descriptors. The standard deviations are provided in parentheses.

Data set = PQC (Test sets)<br>Descs = 3D-MoRSE<br>y = HOMO-LUMO gap (eV)		RF	MIL				
			Non-aggregation (Instance-Wrapper)	Bag-Wrapper	Instance-Net	Bag-Net	Bag-AttentionNet
		RMSE					
Aggregation Method	Boltzmann Weight	0.433 (0.003)	0.404 (0.014)	0.401 (0.017)	0.407 (0.021)	0.403 (0.017)	0.408 (0.016)
	Mean	0.404 (0.003)	0.382 (0.011)	0.384 (0.017)	0.380 (0.021)	0.384 (0.018)	0.390 (0.021)
	Global Minimum	0.450 (0.004)	0.405 (0.005)	0.413 (0.019)	0.411 (0.012)	0.406 (0.012)	0.417 (0.019)
	Random	0.449 (0.004)	0.413 (0.016)	0.412 (0.016)	0.408 (0.009)	0.407 (0.010)	0.418 (0.023)
	RMSD max.	0.458 (0.004)	0.421 (0.025)	0.408 (0.011)	0.424 (0.027)	0.416 (0.021)	0.416 (0.019)
	RMSD min.	0.443 (0.003)	0.412 (0.018)	0.409 (0.011)	0.410 (0.021)	0.408 (0.021)	0.404 (0.014)
Non-Aggregation		0.417 (0.003)	0.345 (0.009)	0.384 (0.009)	0.384 (0.008)	0.384 (0.014)	0.378 (0.008)
Ground-truth		0.449 (0.004)	0.408 (0.014)	0.406 (0.011)	0.412 (0.019)	0.407 (0.016)	0.413 (0.012)

Table S84. Averaged prediction accuracy (R^2) of RF and MIL models for 15 test sets of the PQC data set HOMO-LUMO gap prediction using MBTR descriptors. The standard deviations are provided in parentheses.

Data set = PQC (Test sets)<br>Descs = MBTR<br>y = HOMO-LUMO gap (eV)		RF	MIL				
			Non-aggregation (Instance-Wrapper)	Bag-Wrapper	Instance-Net	Bag-Net	Bag-AttentionNet
			R^2				
Aggregation Method	Boltzmann Weight	0.913 (0.002)	-6518.328 (14134.996)	-4794.570 (12605.364)	-10682.726 (24164.046)	-5820.745 (12894.688)	-8190.800 (18182.475)
	Mean	0.919 (0.002)	-19810.690 (44876.419)	-18607.956 (39023.162)	-18025.366 (39028.013)	-22044.754 (46666.707)	-25916.852 (56752.222)
	Global Minimum	0.910 (0.002)	-4764.439 (10089.333)	-7236.469 (16012.114)	-4917.160 (12623.521)	-7315.113 (16476.828)	-5520.444 (13509.449)
	Random	0.911 (0.002)	-39203.157 (87174.469)	-37297.882 (81694.848)	-27264.599 (61058.391)	-25652.077 (57904.914)	-28554.882 (85591.651)
	RMSD max.	0.909 (0.001)	-7691.414 (17838.963)	-6221.657 (15679.603)	-7706.152 (18543.852)	-7813.439 (17318.174)	-8192.241 (20047.261)
	RMSD min.	0.912 (0.002)	-187.867 (421.177)	-260.238 (623.990)	-336.766 (867.327)	-288.318 (639.481)	-239.524 (599.358)
Non-Aggregation		0.925 (0.002)	-187.517 (407.923)	-153.976 (327.212)	-130.990 (303.589)	-158.758 (397.686)	-217.941 (456.265)
Ground-truth		0.918 (0.002)	0.923 (0.009)	0.922 (0.009)	0.917 (0.012)	0.927 (0.003)	0.925 (0.006)

Table S85. Averaged prediction accuracy (MAE) of RF and MIL models for 15 test sets of the PQC data set HOMO-LUMO gap prediction using MBTR descriptors. The standard deviations are provided in parentheses.

Data set = PQC (Test sets)<br>Descs = MBTR<br>y = HOMO-LUMO gap (eV)		RF	MIL				
			Non-aggregation (Instance-Wrapper)	Bag-Wrapper	Instance-Net	Bag-Net	Bag-AttentionNet
		MAE					
Aggregation Method	Boltzmann Weight	0.228 (0.001)	0.518 (0.569)	0.457 (0.509)	0.583 (0.740)	0.496 (0.543)	0.544 (0.656)
	Mean	0.218 (0.001)	0.705 (1.015)	0.701 (0.969)	0.701 (0.951)	0.738 (1.061)	0.774 (1.148)
	Global Minimum	0.232 (0.001)	0.471 (0.491)	0.522 (0.612)	0.469 (0.516)	0.530 (0.613)	0.488 (0.542)
	Random	0.231 (0.001)	0.909 (1.420)	0.892 (1.385)	0.800 (1.188)	0.781 (1.151)	0.760 (1.238)
	RMSD max.	0.235 (0.001)	0.540 (0.624)	0.498 (0.583)	0.532 (0.644)	0.540 (0.632)	0.536 (0.650)
	RMSD min.	0.228 (0.001)	0.287 (0.099)	0.283 (0.114)	0.294 (0.130)	0.298 (0.117)	0.286 (0.120)
Non-Aggregation		0.208 (0.001)	0.257 (0.109)	0.273 (0.096)	0.269 (0.088)	0.269 (0.099)	0.276 (0.111)
Ground-truth		0.219 (0.001)	0.224 (0.016)	0.226 (0.014)	0.235 (0.023)	0.218 (0.005)	0.221 (0.010)

Table S86. Averaged prediction accuracy (RMSE) of RF and MIL models for 15 test sets of the PQC data set HOMO-LUMO gap prediction using MBTR descriptors. The standard deviations are provided in parentheses.

Data set = PQC (Test sets)<br>Descs = MBTR<br>y = HOMO-LUMO gap (eV)		RF	MIL				
			Non-aggregation (Instance-Wrapper)	Bag-Wrapper	Instance-Net	Bag-Net	Bag-AttentionNet
		RMSE					
Aggregation Method	Boltzmann Weight	0.316 (0.003)	38.789 (80.566)	31.265 (70.286)	48.972 (103.412)	36.462 (76.233)	43.152 (90.488)
	Mean	0.305 (0.003)	66.835 (140.922)	65.803 (136.021)	64.273 (134.077)	71.421 (148.100)	76.880 (161.058)
	Global Minimum	0.321 (0.003)	33.353 (68.856)	40.561 (85.172)	32.162 (70.642)	40.735 (85.544)	34.103 (75.067)
	Random	0.320 (0.003)	94.043 (198.447)	92.239 (193.035)	78.555 (165.402)	75.989 (160.448)	72.750 (173.203)
	RMSD max.	0.323 (0.003)	41.368 (88.004)	36.237 (79.635)	40.718 (88.460)	42.195 (88.440)	41.302 (91.578)
	RMSD min.	0.317 (0.003)	6.775 (13.628)	7.794 (16.147)	8.656 (18.474)	8.335 (16.909)	7.183 (15.640)
Non-Aggregation		0.294 (0.003)	6.776 (13.622)	6.211 (12.303)	5.635 (11.394)	6.007 (12.662)	7.356 (14.641)
Ground-truth		0.307 (0.003)	0.298 (0.018)	0.299 (0.015)	0.307 (0.022)	0.290 (0.006)	0.294 (0.012)

Table S87. Averaged prediction accuracy (R^2) of RF and MIL models for 15 test sets of the PQC data set LUMO prediction using MOE descriptors. The standard deviations are provided in parentheses.

Data set = PQC (Test sets)<br>Descs = MOE<br>y = LUMO (eV)		RF	MIL				
			Non-aggregation (Instance-Wrapper)	Bag-Wrapper	Instance-Net	Bag-Net	Bag-AttentionNet
		R^2					
Aggregation Method	Boltzmann Weight	0.894 (0.001)	0.710 (0.472)	0.673 (0.641)	0.648 (0.723)	0.801 (0.313)	0.855 (0.067)
	Mean	0.904 (0.002)	0.835 (0.246)	0.884 (0.068)	0.857 (0.176)	0.793 (0.444)	0.811 (0.302)
	Global Minimum	0.889 (0.002)	-50.596 (191.336)	-9.584 (30.059)	-18.382 (57.402)	-6.198 (17.496)	-7.406 (19.897)
	Random	0.889 (0.001)	0.634 (0.912)	0.794 (0.285)	0.475 (1.558)	0.679 (0.688)	0.869 (0.047)
	RMSD max.	0.884 (0.002)	0.886 (0.004)	0.876 (0.009)	0.872 (0.039)	0.875 (0.021)	0.864 (0.061)
	RMSD min.	0.891 (0.002)	0.890 (0.005)	0.894 (0.004)	0.891 (0.008)	0.893 (0.005)	0.891 (0.005)
Non-Aggregation		0.909 (0.002)	0.916 (0.010)	0.880 (0.115)	0.878 (0.127)	0.870 (0.150)	0.898 (0.032)
Ground-truth		0.877 (0.004)	0.291 (1.481)	0.454 (1.129)	0.539 (0.825)	0.507 (0.803)	0.514 (0.929)

Table S88. Averaged prediction accuracy (MAE) of RF and MIL models for 15 test sets of the PQC data set LUMO prediction using MOE descriptors. The standard deviations are provided in parentheses.

Data set = PQC (Test sets)<br>Descs = MOE<br>y = LUMO (eV)		RF	MIL				
			Non-aggregation (Instance-Wrapper)	Bag-Wrapper	Instance-Net	Bag-Net	Bag-AttentionNet
		MAE					
Aggregation Method	Boltzmann Weight	0.221 (0.001)	0.226 (0.010)	0.225 (0.010)	0.222 (0.010)	0.220 (0.004)	0.226 (0.015)
	Mean	0.208 (0.001)	0.209 (0.008)	0.205 (0.004)	0.207 (0.007)	0.202 (0.005)	0.206 (0.011)
	Global Minimum	0.227 (0.002)	0.245 (0.045)	0.243 (0.030)	0.245 (0.027)	0.239 (0.016)	0.239 (0.020)
	Random	0.227 (0.001)	0.230 (0.006)	0.232 (0.008)	0.232 (0.014)	0.235 (0.011)	0.226 (0.004)
	RMSD max.	0.232 (0.002)	0.230 (0.004)	0.241 (0.010)	0.235 (0.008)	0.237 (0.013)	0.236 (0.011)
	RMSD min.	0.225 (0.002)	0.226 (0.007)	0.222 (0.006)	0.225 (0.009)	0.224 (0.007)	0.225 (0.004)
Non-Aggregation		0.202 (0.002)	0.194 (0.003)	0.203 (0.007)	0.204 (0.007)	0.206 (0.008)	0.209 (0.005)
Ground-truth		0.235 (0.005)	0.249 (0.013)	0.248 (0.010)	0.249 (0.008)	0.248 (0.007)	0.244 (0.007)

Table S89. Averaged prediction accuracy (RMSE) of RF and MIL models for 15 test sets of the PQC data set LUMO prediction using MOE descriptors. The standard deviations are provided in parentheses.

Data set = PQC (Test sets)<br>Descs = MOE<br>y = LUMO (eV)		RF	MIL				
			Non-aggregation (Instance-Wrapper)	Bag-Wrapper	Instance-Net	Bag-Net	Bag-AttentionNet
		RMSE					
Aggregation Method	Boltzmann Weight	0.298 (0.002)	0.418 (0.270)	0.424 (0.319)	0.436 (0.334)	0.362 (0.195)	0.341 (0.072)
	Mean	0.283 (0.003)	0.334 (0.170)	0.304 (0.072)	0.321 (0.137)	0.342 (0.249)	0.350 (0.199)
	Global Minimum	0.305 (0.003)	2.334 (6.346)	1.432 (2.695)	1.763 (3.741)	1.365 (2.105)	1.389 (2.329)
	Random	0.306 (0.002)	0.422 (0.371)	0.375 (0.184)	0.449 (0.504)	0.421 (0.312)	0.328 (0.051)
	RMSD max.	0.312 (0.002)	0.310 (0.006)	0.322 (0.012)	0.325 (0.043)	0.323 (0.025)	0.333 (0.060)
	RMSD min.	0.303 (0.003)	0.303 (0.007)	0.299 (0.006)	0.302 (0.011)	0.300 (0.007)	0.303 (0.006)
Non-Aggregation		0.276 (0.003)	0.266 (0.015)	0.301 (0.103)	0.302 (0.111)	0.309 (0.124)	0.290 (0.039)
Ground-truth		0.321 (0.006)	0.567 (0.546)	0.517 (0.452)	0.500 (0.382)	0.517 (0.398)	0.496 (0.419)

Table S90. Averaged prediction accuracy (R^2) of RF and MIL models for 15 test sets of the PQC data set LUMO prediction using Pmapper descriptors. The standard deviations are provided in parentheses.

Data set = PQC (Test sets)<br>Descs = Pmapper<br>y = LUMO (eV)		RF	MIL				
			Non-aggregation (Instance-Wrapper)	Bag-Wrapper	Instance-Net	Bag-Net	Bag-AttentionNet
		R^2					
Aggregation Method	Boltzmann Weight	0.739 (0.004)	0.692 (0.008)	0.693 (0.010)	0.694 (0.007)	0.697 (0.005)	0.692 (0.012)
	Mean	0.760 (0.005)	0.746 (0.008)	0.749 (0.004)	0.747 (0.009)	0.746 (0.011)	0.749 (0.006)
	Global Minimum	0.724 (0.004)	-0.000 (0.002)	-0.000 (0.002)	-0.001 (0.002)	-0.000 (0.002)	-0.000 (0.002)
	Random	0.722 (0.004)	-0.000 (0.001)	-0.001 (0.001)	-0.000 (0.001)	-0.000 (0.001)	-0.000 (0.001)
	RMSD max.	0.713 (0.005)	-0.000 (0.001)	-0.000 (0.001)	-0.000 (0.003)	-0.000 (0.001)	0.000 (0.001)
	RMSD min.	0.728 (0.004)	-0.001 (0.001)	-0.001 (0.002)	-0.001 (0.001)	-0.001 (0.001)	-0.001 (0.002)
Non-Aggregation		0.775 (0.003)	0.820 (0.005)	0.759 (0.011)	0.747 (0.012)	0.748 (0.010)	0.775 (0.012)
Ground-truth		0.741 (0.005)	0.000 (0.001)	0.000 (0.001)	0.000 (0.001)	0.000 (0.001)	0.000 (0.001)

Table S91. Averaged prediction accuracy (MAE) of RF and MIL models for 15 test sets of the PQC data set LUMO prediction using Pmapper descriptors. The standard deviations are provided in parentheses.

Data set = PQC (Test sets)<br>Descs = Pmapper<br>y = LUMO (eV)		RF	MIL				
			Non-aggregation (Instance-Wrapper)	Bag-Wrapper	Instance-Net	Bag-Net	Bag-AttentionNet
		MAE					
Aggregation Method	Boltzmann Weight	0.339 (0.002)	0.369 (0.005)	0.370 (0.007)	0.369 (0.005)	0.367 (0.003)	0.369 (0.007)
	Mean	0.321 (0.002)	0.330 (0.006)	0.328 (0.003)	0.330 (0.006)	0.330 (0.007)	0.328 (0.004)
	Global Minimum	0.349 (0.002)	0.659 (0.003)	0.659 (0.003)	0.660 (0.003)	0.660 (0.003)	0.659 (0.003)
	Random	0.350 (0.002)	0.660 (0.003)	0.659 (0.003)	0.660 (0.003)	0.659 (0.003)	0.659 (0.003)
	RMSD max.	0.358 (0.003)	0.659 (0.003)	0.660 (0.003)	0.659 (0.002)	0.659 (0.003)	0.659 (0.003)
	RMSD min.	0.345 (0.002)	0.660 (0.002)	0.660 (0.003)	0.659 (0.003)	0.659 (0.003)	0.660 (0.003)
Non-Aggregation		0.303 (0.002)	0.273 (0.006)	0.321 (0.010)	0.333 (0.009)	0.332 (0.007)	0.309 (0.010)
Ground-truth		0.330 (0.002)	0.659 (0.003)	0.659 (0.003)	0.659 (0.003)	0.659 (0.002)	0.659 (0.002)

Table S92. Averaged prediction accuracy (RMSE) of RF and MIL models for 15 test sets of the PQC data set LUMO prediction using Pmapper descriptors. The standard deviations are provided in parentheses.

Data set = PQC (Test sets)<br>Descs = Pmapper<br>y = LUMO (eV)		RF	MIL				
			Non-aggregation (Instance-Wrapper)	Bag-Wrapper	Instance-Net	Bag-Net	Bag-AttentionNet
		RMSE					
Aggregation Method	Boltzmann Weight	0.469 (0.004)	0.508 (0.007)	0.508 (0.008)	0.507 (0.006)	0.504 (0.003)	0.508 (0.009)
	Mean	0.449 (0.004)	0.462 (0.008)	0.459 (0.004)	0.461 (0.007)	0.462 (0.009)	0.459 (0.005)
	Global Minimum	0.482 (0.004)	0.917 (0.004)	0.917 (0.004)	0.917 (0.004)	0.917 (0.004)	0.917 (0.004)
	Random	0.483 (0.004)	0.917 (0.004)	0.917 (0.004)	0.917 (0.005)	0.917 (0.004)	0.917 (0.004)
	RMSD max.	0.491 (0.004)	0.917 (0.004)	0.917 (0.005)	0.917 (0.004)	0.916 (0.004)	0.917 (0.004)
	RMSD min.	0.478 (0.003)	0.917 (0.004)	0.917 (0.004)	0.917 (0.004)	0.917 (0.004)	0.917 (0.004)
Non-Aggregation		0.435 (0.003)	0.388 (0.006)	0.450 (0.010)	0.461 (0.010)	0.460 (0.009)	0.434 (0.011)
Ground-truth		0.466 (0.003)	0.916 (0.004)	0.917 (0.004)	0.917 (0.004)	0.917 (0.004)	0.917 (0.004)

Table S93. Averaged prediction accuracy (R^2) of RF and MIL models for 15 test sets of the PQC data set LUMO prediction using 3D-MoRSE descriptors. The standard deviations are provided in parentheses.

Data set = PQC (Test sets)<br>Descs = 3D-MoRSE<br>y = LUMO (eV)		RF	MIL				
			Non-aggregation (Instance-Wrapper)	Bag-Wrapper	Instance-Net	Bag-Net	Bag-AttentionNet
		R^2					
Aggregation Method	Boltzmann Weight	0.845 (0.003)	0.904 (0.020)	0.913 (0.010)	0.905 (0.014)	0.916 (0.010)	0.915 (0.008)
	Mean	0.868 (0.003)	0.925 (0.007)	0.926 (0.007)	0.926 (0.005)	0.923 (0.006)	0.926 (0.007)
	Global Minimum	0.830 (0.003)	0.910 (0.006)	0.905 (0.007)	0.905 (0.012)	0.908 (0.006)	0.902 (0.027)
	Random	0.830 (0.004)	0.901 (0.028)	0.908 (0.007)	0.908 (0.011)	0.904 (0.012)	0.909 (0.010)
	RMSD max.	0.823 (0.003)	0.905 (0.008)	0.906 (0.007)	0.906 (0.007)	0.903 (0.010)	0.907 (0.005)
	RMSD min.	0.834 (0.004)	0.911 (0.005)	0.914 (0.004)	0.909 (0.010)	0.909 (0.010)	0.912 (0.006)
Non-Aggregation		0.865 (0.006)	0.926 (0.013)	0.922 (0.009)	0.924 (0.009)	0.921 (0.007)	0.916 (0.013)
Ground-truth		0.837 (0.004)	0.907 (0.011)	0.907 (0.007)	0.909 (0.012)	0.909 (0.011)	0.909 (0.009)

Table S94. Averaged prediction accuracy (MAE) of RF and MIL models for 15 test sets of the PQC data set LUMO prediction using 3D-MoRSE descriptors. The standard deviations are provided in parentheses.

Data set = PQC (Test sets)<br>Descs = 3D-MoRSE<br>y = LUMO (eV)		RF	MIL				
			Non-aggregation (Instance-Wrapper)	Bag-Wrapper	Instance-Net	Bag-Net	Bag-AttentionNet
		MAE					
Aggregation Method	Boltzmann Weight	0.271 (0.003)	0.214 (0.024)	0.203 (0.013)	0.214 (0.019)	0.201 (0.012)	0.203 (0.012)
	Mean	0.247 (0.002)	0.189 (0.009)	0.187 (0.009)	0.187 (0.007)	0.191 (0.009)	0.188 (0.009)
	Global Minimum	0.285 (0.003)	0.207 (0.007)	0.214 (0.009)	0.214 (0.015)	0.209 (0.006)	0.217 (0.029)
	Random	0.286 (0.003)	0.217 (0.028)	0.210 (0.008)	0.211 (0.011)	0.216 (0.017)	0.209 (0.012)
	RMSD max.	0.292 (0.004)	0.214 (0.010)	0.213 (0.007)	0.212 (0.009)	0.215 (0.011)	0.211 (0.007)
	RMSD min.	0.283 (0.003)	0.206 (0.006)	0.203 (0.005)	0.209 (0.012)	0.207 (0.010)	0.206 (0.008)
Non-Aggregation		0.255 (0.006)	0.188 (0.016)	0.193 (0.012)	0.191 (0.012)	0.194 (0.009)	0.202 (0.017)
Ground-truth		0.279 (0.004)	0.210 (0.015)	0.210 (0.010)	0.207 (0.011)	0.209 (0.014)	0.206 (0.010)

Table S95. Averaged prediction accuracy (RMSE) of RF and MIL models for 15 test sets of the PQC data set LUMO prediction using 3D-MoRSE descriptors. The standard deviations are provided in parentheses.

Data set = PQC (Test sets)<br>Descs = 3D-MoRSE<br>y = LUMO (eV)		RF	MIL				
			Non-aggregation (Instance-Wrapper)	Bag-Wrapper	Instance-Net	Bag-Net	Bag-AttentionNet
		RMSE					
Aggregation Method	Boltzmann Weight	0.360 (0.003)	0.282 (0.028)	0.269 (0.014)	0.282 (0.020)	0.265 (0.014)	0.267 (0.012)
	Mean	0.333 (0.004)	0.251 (0.011)	0.248 (0.011)	0.249 (0.008)	0.254 (0.010)	0.250 (0.012)
	Global Minimum	0.378 (0.004)	0.275 (0.010)	0.283 (0.011)	0.282 (0.017)	0.277 (0.010)	0.286 (0.035)
	Random	0.378 (0.004)	0.286 (0.035)	0.278 (0.009)	0.277 (0.015)	0.283 (0.018)	0.276 (0.014)
	RMSD max.	0.386 (0.004)	0.282 (0.011)	0.281 (0.011)	0.280 (0.009)	0.285 (0.014)	0.280 (0.009)
	RMSD min.	0.374 (0.004)	0.274 (0.008)	0.269 (0.006)	0.276 (0.015)	0.275 (0.015)	0.272 (0.008)
Non-Aggregation		0.336 (0.007)	0.249 (0.019)	0.256 (0.014)	0.252 (0.014)	0.257 (0.012)	0.265 (0.018)
Ground-truth		0.370 (0.004)	0.279 (0.017)	0.279 (0.011)	0.276 (0.017)	0.276 (0.016)	0.276 (0.014)

Table S96. Averaged prediction accuracy (R^2) of RF and MIL models for 15 test sets of the PQC data set LUMO prediction using MBTR descriptors. The standard deviations are provided in parentheses.

Data set = PQC (Test sets)<br>Descs = MBTR<br>y = LUMO (eV)		RF	MIL				
			Non-aggregation (Instance-Wrapper)	Bag-Wrapper	Instance-Net	Bag-Net	Bag-AttentionNet
			R^2				
Aggregation Method	Boltzmann Weight	0.940 (0.001)	-485.030 (1056.436)	-407.129 (921.903)	-333.458 (702.938)	-535.109 (1636.348)	-436.041 (918.389)
	Mean	0.943 (0.001)	-592.864 (1266.614)	-809.845 (1933.836)	-783.780 (1765.029)	-1437.803 (3083.398)	-749.571 (1621.385)
	Global Minimum	0.937 (0.001)	-843.334 (1932.679)	-766.983 (1680.942)	-666.820 (1422.471)	-764.102 (1683.291)	-777.714 (1793.739)
	Random	0.938 (0.001)	-1751.865 (3877.904)	-1043.100 (2449.850)	-1272.814 (3337.838)	-1672.167 (4578.499)	-1265.966 (3021.538)
	RMSD max.	0.936 (0.001)	-685.670 (1787.938)	-629.189 (1547.594)	-579.670 (1544.153)	-249.427 (758.123)	-242.906 (636.357)
	RMSD min.	0.939 (0.001)	-11.083 (25.439)	-11.517 (26.308)	-13.072 (30.671)	-11.603 (26.363)	-9.050 (21.859)
Non-Aggregation		0.946 (0.002)	-8.505 (20.377)	-2.594 (7.893)	-1.998 (7.527)	-2.073 (7.991)	-3.755 (10.236)
Ground-truth		0.940 (0.001)	0.956 (0.009)	0.959 (0.004)	0.956 (0.006)	0.956 (0.008)	0.957 (0.005)

Table S97. Averaged prediction accuracy (MAE) of RF and MIL models for 15 test sets of the PQC data set LUMO prediction using MBTR descriptors. The standard deviations are provided in parentheses.

Data set = PQC (Test sets)<br>Descs = MBTR<br>y = LUMO (eV)		RF	MIL				
			Non-aggregation (Instance-Wrapper)	Bag-Wrapper	Instance-Net	Bag-Net	Bag-AttentionNet
		MAE					
Aggregation Method	Boltzmann Weight	0.163 (0.001)	0.216 (0.131)	0.204 (0.124)	0.202 (0.113)	0.209 (0.145)	0.212 (0.127)
	Mean	0.157 (0.001)	0.210 (0.148)	0.225 (0.175)	0.231 (0.169)	0.256 (0.232)	0.220 (0.166)
	Global Minimum	0.166 (0.001)	0.235 (0.183)	0.230 (0.166)	0.226 (0.157)	0.230 (0.169)	0.230 (0.169)
	Random	0.166 (0.001)	0.271 (0.253)	0.243 (0.201)	0.249 (0.221)	0.265 (0.250)	0.247 (0.219)
	RMSD max.	0.168 (0.001)	0.220 (0.162)	0.219 (0.156)	0.219 (0.148)	0.194 (0.098)	0.196 (0.103)
	RMSD min.	0.163 (0.001)	0.159 (0.021)	0.159 (0.022)	0.157 (0.023)	0.159 (0.022)	0.160 (0.032)
Non-Aggregation		0.152 (0.002)	0.147 (0.022)	0.150 (0.014)	0.153 (0.017)	0.150 (0.015)	0.148 (0.017)
Ground-truth		0.159 (0.001)	0.144 (0.018)	0.137 (0.007)	0.143 (0.012)	0.144 (0.016)	0.142 (0.011)

Table S98. Averaged prediction accuracy (RMSE) of RF and MIL models for 15 test sets of the PQC data set LUMO prediction using MBTR descriptors. The standard deviations are provided in parentheses.

Data set = PQC (Test sets)<br>Descs = MBTR<br>y = LUMO (eV)		RF	MIL				
			Non-aggregation (Instance-Wrapper)	Bag-Wrapper	Instance-Net	Bag-Net	Bag-AttentionNet
		RMSE					
Aggregation Method	Boltzmann Weight	0.225 (0.002)	9.089 (18.658)	8.275 (17.131)	7.632 (15.458)	8.532 (20.046)	8.697 (17.659)
	Mean	0.218 (0.002)	10.080 (20.648)	11.456 (24.221)	11.446 (23.792)	15.580 (32.182)	11.270 (23.196)
	Global Minimum	0.229 (0.003)	11.823 (24.698)	11.385 (23.511)	10.697 (21.895)	11.357 (23.489)	11.267 (23.795)
	Random	0.229 (0.002)	17.098 (35.606)	12.975 (27.593)	13.999 (30.695)	15.791 (35.315)	14.306 (30.411)
	RMSD max.	0.232 (0.002)	10.094 (22.581)	9.802 (21.592)	9.235 (20.786)	5.743 (13.791)	5.918 (13.514)
	RMSD min.	0.226 (0.002)	1.576 (2.869)	1.601 (2.921)	1.670 (3.115)	1.607 (2.931)	1.438 (2.612)
Non-Aggregation		0.214 (0.004)	1.397 (2.540)	0.913 (1.527)	0.818 (1.403)	0.756 (1.463)	1.030 (1.771)
Ground-truth		0.224 (0.002)	0.192 (0.018)	0.184 (0.009)	0.191 (0.012)	0.192 (0.017)	0.190 (0.011)

Table S99. Averaged prediction accuracy (R^2) of RF and MIL models for 15 test sets of the PQC data set energy prediction using MOE descriptors. The standard deviations are provided in parentheses.

Data set = PQC (Test sets)<br>Descs = MOE<br>y = Energy (eV)		RF	MIL				
			Non-aggregation (Instance-Wrapper)	Bag-Wrapper	Instance-Net	Bag-Net	Bag-AttentionNet
		R^2					
Aggregation Method	Boltzmann Weight	0.879 (0.003)	0.558 (0.836)	0.368 (1.395)	0.554 (1.072)	0.625 (0.941)	-2.234 (11.441)
	Mean	0.894 (0.003)	0.748 (0.502)	0.747 (0.598)	0.825 (0.375)	0.575 (1.274)	0.687 (0.782)
	Global Minimum	0.872 (0.003)	-56.034 (182.664)	-5.579 (16.463)	-206.342 (781.550)	-55.460 (204.245)	-137.509 (403.002)
	Random	0.873 (0.003)	-0.579 (3.781)	-0.283 (3.107)	0.389 (1.437)	0.533 (0.901)	-0.301 (2.687)
	RMSD max.	0.867 (0.003)	0.910 (0.011)	0.894 (0.036)	0.899 (0.030)	0.906 (0.015)	0.907 (0.014)
	RMSD min.	0.874 (0.003)	0.914 (0.010)	0.911 (0.026)	0.908 (0.049)	0.917 (0.015)	0.916 (0.011)
Non-Aggregation		0.907 (0.007)	0.956 (0.004)	0.914 (0.110)	0.936 (0.033)	0.908 (0.091)	0.933 (0.026)
Ground-truth		0.849 (0.015)	0.312 (1.556)	0.309 (1.786)	0.478 (1.576)	0.665 (0.720)	0.492 (0.841)

Table S100. Averaged prediction accuracy (MAE) of RF and MIL models for 15 test sets of the PQC data set energy prediction using MOE descriptors. The standard deviations are provided in parentheses.

Data set = PQC (Test sets)<br>Descs = MOE<br>y = Energy (eV)		RF	MIL				
			Non-aggregation (Instance-Wrapper)	Bag-Wrapper	Instance-Net	Bag-Net	Bag-AttentionNet
		MAE					
Aggregation Method	Boltzmann Weight	0.961 (0.009)	0.818 (0.055)	0.804 (0.052)	0.801 (0.039)	0.799 (0.061)	0.827 (0.080)
	Mean	0.883 (0.007)	0.703 (0.087)	0.715 (0.099)	0.655 (0.033)	0.664 (0.043)	0.713 (0.074)
	Global Minimum	0.994 (0.008)	0.942 (0.214)	0.900 (0.123)	0.981 (0.404)	0.914 (0.216)	0.987 (0.344)
	Random	0.990 (0.009)	0.868 (0.056)	0.860 (0.064)	0.836 (0.055)	0.823 (0.059)	0.835 (0.047)
	RMSD max.	1.018 (0.010)	0.869 (0.063)	0.857 (0.071)	0.863 (0.086)	0.856 (0.063)	0.861 (0.062)
	RMSD min.	0.982 (0.008)	0.824 (0.046)	0.808 (0.032)	0.800 (0.052)	0.802 (0.041)	0.832 (0.071)
Non-Aggregation		0.814 (0.034)	0.571 (0.032)	0.661 (0.045)	0.655 (0.042)	0.681 (0.043)	0.702 (0.076)
Ground-truth		1.103 (0.058)	0.827 (0.043)	0.855 (0.131)	0.807 (0.061)	0.835 (0.072)	0.798 (0.022)

Table S101. Averaged prediction accuracy (RMSE) of RF and MIL models for 15 test sets of the PQC data set energy prediction using MOE descriptors. The standard deviations are provided in parentheses.

Data set = PQC (Test sets) Descs = MOE y = Energy (eV)		RF	MIL				
			Non-aggregation (Instance-Wrapper)	Bag-Wrapper	Instance-Net	Bag-Net	Bag-AttentionNet
		RMSE					
Aggregation Method	Boltzmann Weight	1.380 (0.018)	2.050 (1.729)	2.146 (2.396)	1.941 (1.878)	1.796 (1.712)	3.355 (6.541)
	Mean	1.292 (0.017)	1.585 (1.254)	1.524 (1.342)	1.334 (1.032)	1.648 (2.074)	1.626 (1.575)
	Global Minimum	1.421 (0.017)	11.569 (28.724)	5.350 (8.967)	18.969 (55.961)	11.139 (28.716)	18.150 (44.791)
	Random	1.417 (0.019)	2.967 (4.156)	2.700 (3.719)	2.128 (2.349)	2.003 (1.895)	2.864 (3.632)
	RMSD max.	1.446 (0.018)	1.187 (0.069)	1.277 (0.198)	1.249 (0.172)	1.215 (0.092)	1.209 (0.091)
	RMSD min.	1.409 (0.016)	1.164 (0.068)	1.177 (0.155)	1.183 (0.249)	1.137 (0.095)	1.151 (0.075)
Non-Aggregation		1.210 (0.041)	0.836 (0.039)	1.070 (0.476)	0.988 (0.206)	1.126 (0.452)	1.016 (0.168)
Ground-truth		1.543 (0.070)	2.190 (2.561)	2.196 (2.560)	1.866 (2.272)	1.781 (1.503)	2.134 (1.922)

Table S102. Averaged prediction accuracy (R^2) of RF and MIL models for 15 test sets of the PQC data set energy prediction using Pmapper descriptors. The standard deviations are provided in parentheses.

Data set = PQC (Test sets)<br>Descs = Pmapper<br>y = Energy (eV)		RF	MIL				
			Non-aggregation (Instance-Wrapper)	Bag-Wrapper	Instance-Net	Bag-Net	Bag-AttentionNet
		R^2					
Aggregation Method	Boltzmann Weight	0.678 (0.005)	0.600 (0.010)	0.603 (0.010)	0.601 (0.007)	0.602 (0.008)	0.603 (0.009)
	Mean	0.706 (0.005)	0.685 (0.009)	0.684 (0.004)	0.685 (0.006)	0.682 (0.009)	0.685 (0.008)
	Global Minimum	0.664 (0.005)	-0.009 (0.007)	-0.009 (0.006)	-0.009 (0.007)	-0.009 (0.007)	-0.010 (0.007)
	Random	0.663 (0.004)	-0.007 (0.005)	-0.007 (0.005)	-0.007 (0.005)	-0.007 (0.005)	-0.007 (0.005)
	RMSD max.	0.654 (0.006)	-0.011 (0.009)	-0.010 (0.008)	-0.010 (0.008)	-0.010 (0.008)	-0.010 (0.008)
	RMSD min.	0.663 (0.004)	-0.012 (0.011)	-0.012 (0.010)	-0.012 (0.010)	-0.012 (0.010)	-0.012 (0.010)
Non-Aggregation		0.719 (0.005)	0.793 (0.005)	0.701 (0.009)	0.670 (0.010)	0.674 (0.008)	0.710 (0.021)
Ground-truth		0.669 (0.005)	-0.005 (0.005)	-0.005 (0.005)	-0.005 (0.006)	-0.005 (0.006)	-0.005 (0.006)

Table S103. Averaged prediction accuracy (MAE) of RF and MIL models for 15 test sets of the PQC data set energy prediction using Pmapper descriptors. The standard deviations are provided in parentheses.

Data set = PQC (Test sets)<br>Descs = Pmapper<br>y = Energy (eV)		RF	MIL				
			Non-aggregation (Instance-Wrapper)	Bag-Wrapper	Instance-Net	Bag-Net	Bag-AttentionNet
		MAE					
Aggregation Method	Boltzmann Weight	1.660 (0.012)	1.864 (0.021)	1.854 (0.020)	1.859 (0.019)	1.857 (0.018)	1.855 (0.020)
	Mean	1.566 (0.015)	1.621 (0.024)	1.623 (0.018)	1.619 (0.021)	1.632 (0.025)	1.620 (0.020)
	Global Minimum	1.699 (0.014)	2.996 (0.017)	2.996 (0.017)	2.998 (0.018)	2.994 (0.019)	2.999 (0.018)
	Random	1.698 (0.012)	2.995 (0.017)	2.995 (0.020)	2.993 (0.013)	2.994 (0.021)	2.995 (0.021)
	RMSD max.	1.730 (0.015)	2.998 (0.014)	2.999 (0.018)	2.998 (0.021)	2.999 (0.020)	2.994 (0.018)
	RMSD min.	1.691 (0.012)	2.995 (0.018)	2.994 (0.018)	2.996 (0.020)	2.993 (0.018)	2.997 (0.018)
Non-Aggregation		1.522 (0.015)	1.272 (0.012)	1.568 (0.025)	1.658 (0.027)	1.648 (0.020)	1.543 (0.061)
Ground-truth		1.675 (0.016)	2.990 (0.019)	2.991 (0.022)	2.993 (0.017)	2.995 (0.020)	2.992 (0.016)

Table S104. Averaged prediction accuracy (RMSE) of RF and MIL models for 15 test sets of the PQC data set energy prediction using Pmapper descriptors. The standard deviations are provided in parentheses.

Data set = PQC (Test sets)<br>Descs = Pmapper<br>y = Energy (eV)		RF	MIL				
			Non-aggregation (Instance-Wrapper)	Bag-Wrapper	Instance-Net	Bag-Net	Bag-AttentionNet
		RMSE					
Aggregation Method	Boltzmann Weight	2.254 (0.023)	2.511 (0.030)	2.502 (0.028)	2.508 (0.029)	2.505 (0.025)	2.503 (0.029)
	Mean	2.152 (0.025)	2.229 (0.035)	2.232 (0.022)	2.228 (0.030)	2.238 (0.034)	2.229 (0.028)
	Global Minimum	2.303 (0.025)	3.990 (0.025)	3.990 (0.025)	3.990 (0.025)	3.990 (0.025)	3.990 (0.024)
	Random	2.306 (0.022)	3.985 (0.023)	3.985 (0.022)	3.986 (0.023)	3.985 (0.023)	3.985 (0.023)
	RMSD max.	2.335 (0.027)	3.992 (0.019)	3.992 (0.019)	3.991 (0.020)	3.991 (0.019)	3.991 (0.019)
	RMSD min.	2.304 (0.020)	3.995 (0.032)	3.995 (0.031)	3.995 (0.030)	3.994 (0.031)	3.994 (0.030)
Non-Aggregation		2.104 (0.025)	1.806 (0.025)	2.172 (0.037)	2.280 (0.034)	2.266 (0.032)	2.138 (0.082)
Ground-truth		2.285 (0.025)	3.981 (0.026)	3.981 (0.026)	3.982 (0.026)	3.982 (0.025)	3.982 (0.025)

Table S105. Averaged prediction accuracy (R^2) of RF and MIL models for 15 test sets of the PQC data set energy prediction using 3D-MoRSE descriptors. The standard deviations are provided in parentheses.

Data set = PQC (Test sets)<br>Descs = 3D-MoRSE<br>y = Energy (eV)		RF	MIL				
			Non-aggregation (Instance-Wrapper)	Bag-Wrapper	Instance-Net	Bag-Net	Bag-AttentionNet
		R^2					
Aggregation Method	Boltzmann Weight	0.739 (0.005)	0.930 (0.015)	0.933 (0.018)	0.935 (0.010)	0.922 (0.039)	0.934 (0.014)
	Mean	0.793 (0.003)	0.948 (0.011)	0.947 (0.018)	0.951 (0.008)	0.946 (0.019)	0.946 (0.019)
	Global Minimum	0.709 (0.008)	0.913 (0.023)	0.924 (0.021)	0.899 (0.051)	0.917 (0.025)	0.920 (0.020)
	Random	0.715 (0.005)	0.929 (0.016)	0.922 (0.020)	0.921 (0.020)	0.912 (0.040)	0.931 (0.012)
	RMSD max.	0.706 (0.004)	0.921 (0.019)	0.927 (0.011)	0.917 (0.029)	0.922 (0.013)	0.926 (0.018)
	RMSD min.	0.717 (0.004)	0.926 (0.019)	0.924 (0.024)	0.929 (0.017)	0.922 (0.030)	0.911 (0.026)
Non-Aggregation		0.789 (0.004)	0.972 (0.008)	0.941 (0.012)	0.937 (0.031)	0.940 (0.021)	0.944 (0.011)
Ground-truth		0.708 (0.005)	0.913 (0.032)	0.921 (0.022)	0.913 (0.034)	0.931 (0.014)	0.909 (0.040)

Table S106. Averaged prediction accuracy (MAE) of RF and MIL models for 15 test sets of the PQC data set energy prediction using 3D-MoRSE descriptors. The standard deviations are provided in parentheses.

Data set = PQC (Test sets)<br>Descs = 3D-MoRSE<br>y = Energy (eV)		RF	MIL				
			Non-aggregation (Instance-Wrapper)	Bag-Wrapper	Instance-Net	Bag-Net	Bag-AttentionNet
		MAE					
Aggregation Method	Boltzmann Weight	1.472 (0.014)	0.783 (0.104)	0.757 (0.121)	0.742 (0.070)	0.812 (0.205)	0.749 (0.098)
	Mean	1.283 (0.010)	0.664 (0.089)	0.666 (0.121)	0.640 (0.060)	0.608 (0.072)	0.669 (0.140)
	Global Minimum	1.575 (0.028)	0.876 (0.147)	0.809 (0.132)	0.939 (0.281)	0.846 (0.137)	0.845 (0.133)
	Random	1.557 (0.014)	0.771 (0.094)	0.824 (0.119)	0.839 (0.137)	0.871 (0.209)	0.768 (0.082)
	RMSD max.	1.588 (0.012)	0.824 (0.111)	0.800 (0.079)	0.856 (0.174)	0.828 (0.091)	0.801 (0.120)
	RMSD min.	1.546 (0.015)	0.799 (0.122)	0.818 (0.158)	0.779 (0.098)	0.827 (0.192)	0.906 (0.171)
Non-Aggregation		1.312 (0.013)	0.479 (0.082)	0.715 (0.098)	0.733 (0.204)	0.721 (0.156)	0.685 (0.069)
Ground-truth		1.574 (0.016)	0.877 (0.190)	0.834 (0.148)	0.888 (0.207)	0.766 (0.090)	0.901 (0.227)

Table S107. Averaged prediction accuracy (RMSE) of RF and MIL models for 15 test sets of the PQC data set energy prediction using 3D-MoRSE descriptors. The standard deviations are provided in parentheses.

Data set = PQC (Test sets)<br>Descs = 3D-MoRSE<br>y = Energy (eV)		RF	MIL				
			Non-aggregation (Instance-Wrapper)	Bag-Wrapper	Instance-Net	Bag-Net	Bag-AttentionNet
		RMSE					
Aggregation Method	Boltzmann Weight	2.027 (0.020)	1.048 (0.111)	1.023 (0.135)	1.010 (0.078)	1.086 (0.245)	1.019 (0.104)
	Mean	1.809 (0.013)	0.901 (0.087)	0.904 (0.133)	0.880 (0.072)	0.844 (0.089)	0.911 (0.152)
	Global Minimum	2.143 (0.030)	1.160 (0.152)	1.084 (0.146)	1.231 (0.294)	1.131 (0.165)	1.119 (0.140)
	Random	2.119 (0.018)	1.049 (0.112)	1.101 (0.132)	1.106 (0.135)	1.157 (0.229)	1.039 (0.090)
	RMSD max.	2.154 (0.016)	1.106 (0.127)	1.071 (0.085)	1.132 (0.185)	1.106 (0.095)	1.071 (0.119)
	RMSD min.	2.112 (0.020)	1.073 (0.130)	1.085 (0.161)	1.055 (0.118)	1.091 (0.195)	1.172 (0.170)
Non-Aggregation		1.825 (0.019)	0.664 (0.091)	0.961 (0.103)	0.975 (0.203)	0.959 (0.156)	0.937 (0.090)
Ground-truth		2.146 (0.021)	1.153 (0.202)	1.107 (0.148)	1.155 (0.209)	1.041 (0.097)	1.174 (0.235)

Table S108. Averaged prediction accuracy (R^2) of RF and MIL models for 15 test sets of the PQC data set energy prediction using MBTR descriptors. The standard deviations are provided in parentheses.

Data set = PQC (Test sets)<br>Descs = MBTR<br>y = Energy (eV)		RF	MIL				
			Non-aggregation (Instance-Wrapper)	Bag-Wrapper	Instance-Net	Bag-Net	Bag-AttentionNet
		R^2					
Aggregation Method	Boltzmann Weight	0.960 (0.001)	-210.308 (453.744)	-170.256 (452.850)	-49.556 (149.447)	-67.645 (201.157)	-169.196 (458.785)
	Mean	0.963 (0.001)	-175.372 (520.130)	-491.836 (1109.648)	-227.535 (555.686)	-189.986 (539.574)	-263.560 (992.043)
	Global Minimum	0.957 (0.001)	-64.591 (211.435)	-28.810 (91.678)	-26.540 (94.534)	-7.780 (21.679)	-29.418 (86.843)
	Random	0.954 (0.003)	-218.458 (781.746)	-35.579 (81.314)	-41.946 (89.907)	-481.210 (1628.524)	-437.267 (1122.137)
	RMSD max.	0.955 (0.001)	-159.966 (555.071)	-32.943 (98.490)	-147.708 (485.257)	-100.284 (219.560)	-54.733 (193.185)
	RMSD min.	0.956 (0.001)	-26.046 (57.971)	-12.015 (31.500)	-19.545 (45.111)	-11.971 (29.156)	-20.553 (53.665)
Non-Aggregation		0.968 (0.001)	-2.715 (8.018)	-52.195 (112.305)	-19.587 (44.107)	-12.269 (35.869)	-12.003 (29.003)
Ground-truth		0.954 (0.002)	0.987 (0.003)	0.987 (0.003)	0.987 (0.003)	0.988 (0.001)	0.987 (0.002)

Table S109. Averaged prediction accuracy (MAE) of RF and MIL models for 15 test sets of the PQC data set energy prediction using MBTR descriptors. The standard deviations are provided in parentheses.

Data set = PQC (Test sets)<br>Descs = MBTR<br>y = Energy (eV)		RF	MIL				
			Non-aggregation (Instance-Wrapper)	Bag-Wrapper	Instance-Net	Bag-Net	Bag-AttentionNet
		MAE					
Aggregation Method	Boltzmann Weight	0.509 (0.006)	0.467 (0.381)	0.430 (0.352)	0.380 (0.239)	0.378 (0.226)	0.444 (0.364)
	Mean	0.481 (0.007)	0.398 (0.377)	0.543 (0.597)	0.457 (0.417)	0.408 (0.367)	0.399 (0.444)
	Global Minimum	0.532 (0.005)	0.367 (0.235)	0.349 (0.152)	0.351 (0.141)	0.354 (0.108)	0.349 (0.152)
	Random	0.547 (0.025)	0.446 (0.404)	0.387 (0.170)	0.388 (0.174)	0.549 (0.602)	0.548 (0.551)
	RMSD max.	0.542 (0.005)	0.433 (0.353)	0.364 (0.189)	0.437 (0.329)	0.449 (0.275)	0.373 (0.197)
	RMSD min.	0.535 (0.005)	0.370 (0.148)	0.343 (0.094)	0.354 (0.127)	0.343 (0.103)	0.350 (0.128)
Non-Aggregation		0.421 (0.007)	0.258 (0.065)	0.351 (0.195)	0.292 (0.118)	0.305 (0.113)	0.302 (0.100)
Ground-truth		0.542 (0.007)	0.299 (0.040)	0.300 (0.042)	0.306 (0.031)	0.302 (0.027)	0.302 (0.030)

Table S110. Averaged prediction accuracy (RMSE) of RF and MIL models for 15 test sets of the PQC data set energy prediction using MBTR descriptors. The standard deviations are provided in parentheses.

Data set = PQC (Test sets) Descs = MBTR y = Energy (eV)		RF	MIL				
			Non-aggregation (Instance-Wrapper)	Bag-Wrapper	Instance-Net	Bag-Net	Bag-AttentionNet
		RMSE					
Aggregation Method	Boltzmann Weight	0.792 (0.016)	25.964 (53.353)	20.423 (49.325)	11.041 (26.867)	12.792 (31.277)	21.801 (48.527)
	Mean	0.764 (0.015)	21.378 (49.809)	38.856 (81.807)	25.751 (56.241)	22.761 (51.637)	20.108 (63.316)
	Global Minimum	0.822 (0.015)	12.393 (30.674)	8.491 (20.657)	7.411 (20.089)	5.413 (10.802)	9.282 (20.516)
	Random	0.850 (0.026)	19.921 (57.122)	10.937 (22.156)	11.972 (23.929)	32.544 (83.648)	34.779 (78.019)
	RMSD max.	0.840 (0.015)	18.329 (48.866)	9.282 (22.043)	18.753 (46.474)	18.051 (36.880)	11.039 (28.450)
	RMSD min.	0.836 (0.013)	9.533 (18.998)	6.518 (13.251)	8.314 (16.563)	6.623 (13.147)	8.236 (17.131)
Non-Aggregation		0.707 (0.016)	3.665 (6.962)	13.206 (26.686)	8.275 (16.562)	6.351 (13.510)	6.587 (13.171)
Ground-truth		0.851 (0.017)	0.445 (0.049)	0.445 (0.044)	0.453 (0.042)	0.442 (0.024)	0.447 (0.039)

Table S111. Averaged prediction accuracy (R^2) of RF and MIL models for 15 test sets of the PQC data set enthalpy prediction using MOE descriptors. The standard deviations are provided in parentheses.

Data set = PQC (Test sets)<br>Descs = MOE<br>y = Enthalpy (Hartree)		RF	MIL				
			Non-aggregation (Instance-Wrapper)	Bag-Wrapper	Instance-Net	Bag-Net	Bag-AttentionNet
		R^2					
Aggregation Method	Boltzmann Weight	0.912 (0.007)	0.912 (0.036)	0.910 (0.055)	0.903 (0.077)	0.909 (0.064)	0.911 (0.057)
	Mean	0.923 (0.002)	0.928 (0.059)	0.919 (0.099)	0.900 (0.129)	0.912 (0.102)	0.903 (0.148)
	Global Minimum	0.908 (0.002)	0.541 (1.141)	0.723 (0.456)	0.667 (0.586)	0.662 (0.742)	0.552 (0.911)
	Random	0.906 (0.002)	0.906 (0.027)	0.911 (0.027)	0.904 (0.034)	0.906 (0.030)	0.898 (0.042)
	RMSD max.	0.904 (0.002)	0.915 (0.024)	0.907 (0.035)	0.921 (0.016)	0.911 (0.024)	0.910 (0.019)
	RMSD min.	0.908 (0.002)	0.924 (0.009)	0.922 (0.009)	0.925 (0.011)	0.912 (0.027)	0.926 (0.008)
Non-Aggregation		0.933 (0.006)	0.936 (0.024)	0.931 (0.040)	0.918 (0.060)	0.929 (0.039)	0.924 (0.024)
Ground-truth		0.901 (0.002)	0.772 (0.340)	0.747 (0.395)	0.701 (0.447)	0.747 (0.362)	0.737 (0.406)

Table S112. Averaged prediction accuracy (MAE) of RF and MIL models for 15 test sets of the PQC data set enthalpy prediction using MOE descriptors. The standard deviations are provided in parentheses.

Data set = PQC (Test sets) Descs = MOE y = Enthalpy (Hartree)		RF	MIL				
			Non-aggregation (Instance-Wrapper)	Bag-Wrapper	Instance-Net	Bag-Net	Bag-AttentionNet
		MAE					
Aggregation Method	Boltzmann Weight	0.033 (0.002)	0.032 (0.003)	0.032 (0.004)	0.033 (0.003)	0.032 (0.003)	0.033 (0.004)
	Mean	0.030 (0.000)	0.028 (0.003)	0.027 (0.002)	0.031 (0.005)	0.029 (0.004)	0.028 (0.002)
	Global Minimum	0.034 (0.000)	0.034 (0.002)	0.035 (0.003)	0.035 (0.003)	0.035 (0.003)	0.036 (0.003)
	Random	0.034 (0.000)	0.034 (0.005)	0.034 (0.003)	0.034 (0.003)	0.034 (0.002)	0.035 (0.005)
	RMSD max.	0.035 (0.000)	0.035 (0.006)	0.037 (0.008)	0.034 (0.003)	0.036 (0.006)	0.036 (0.004)
	RMSD min.	0.034 (0.000)	0.033 (0.003)	0.034 (0.002)	0.033 (0.003)	0.036 (0.007)	0.032 (0.002)
Non-Aggregation		0.028 (0.002)	0.028 (0.002)	0.029 (0.003)	0.031 (0.004)	0.030 (0.004)	0.032 (0.005)
Ground-truth		0.036 (0.000)	0.033 (0.003)	0.033 (0.005)	0.035 (0.006)	0.034 (0.004)	0.033 (0.004)

Table S113. Averaged prediction accuracy (RMSE) of RF and MIL models for 15 test sets of the PQC data set enthalpy prediction using MOE descriptors. The standard deviations are provided in parentheses.

Data set = PQC (Test sets)<br>Descs = MOE<br>y = Enthalpy (Hartree)		RF	MIL				
			Non-aggregation (Instance-Wrapper)	Bag-Wrapper	Instance-Net	Bag-Net	Bag-AttentionNet
		RMSE					
Aggregation Method	Boltzmann Weight	0.048 (0.002)	0.047 (0.009)	0.047 (0.012)	0.048 (0.014)	0.047 (0.013)	0.047 (0.012)
	Mean	0.045 (0.001)	0.042 (0.013)	0.043 (0.018)	0.047 (0.022)	0.045 (0.019)	0.045 (0.024)
	Global Minimum	0.049 (0.001)	0.078 (0.080)	0.070 (0.050)	0.075 (0.057)	0.073 (0.061)	0.081 (0.074)
	Random	0.049 (0.001)	0.049 (0.007)	0.048 (0.007)	0.050 (0.008)	0.049 (0.007)	0.051 (0.010)
	RMSD max.	0.050 (0.001)	0.047 (0.006)	0.049 (0.008)	0.045 (0.004)	0.048 (0.006)	0.048 (0.005)
	RMSD min.	0.049 (0.001)	0.044 (0.003)	0.045 (0.003)	0.044 (0.003)	0.048 (0.007)	0.044 (0.002)
Non-Aggregation		0.042 (0.002)	0.040 (0.007)	0.042 (0.010)	0.045 (0.013)	0.042 (0.010)	0.044 (0.006)
Ground-truth		0.051 (0.001)	0.065 (0.043)	0.067 (0.048)	0.072 (0.053)	0.068 (0.046)	0.068 (0.050)

Table S114. Averaged prediction accuracy (R^2) of RF and MIL models for 15 test sets of the PQC data set enthalpy prediction using Pmapper descriptors. The standard deviations are provided in parentheses.

Data set = PQC (Test sets)<br>Descs = Pmapper<br>y = Enthalpy (Hartree)		RF	MIL				
			Non-aggregation (Instance-Wrapper)	Bag-Wrapper	Instance-Net	Bag-Net	Bag-AttentionNet
		R^2					
Aggregation Method	Boltzmann Weight	0.599 (0.004)	0.620 (0.030)	0.639 (0.012)	0.620 (0.030)	0.624 (0.028)	0.629 (0.017)
	Mean	0.643 (0.005)	0.699 (0.041)	0.709 (0.021)	0.705 (0.031)	0.704 (0.026)	0.709 (0.025)
	Global Minimum	0.572 (0.005)	-0.000 (0.000)	-0.000 (0.000)	-0.000 (0.000)	-0.000 (0.001)	-0.000 (0.001)
	Random	0.574 (0.004)	-0.000 (0.000)	-0.000 (0.000)	-0.000 (0.000)	-0.000 (0.000)	-0.000 (0.000)
	RMSD max.	0.564 (0.004)	-0.000 (0.000)	-0.000 (0.001)	-0.000 (0.000)	-0.000 (0.000)	-0.000 (0.001)
	RMSD min.	0.574 (0.003)	-0.000 (0.000)	-0.000 (0.000)	-0.000 (0.000)	-0.000 (0.000)	-0.000 (0.000)
Non-Aggregation		0.661 (0.004)	0.768 (0.010)	0.728 (0.031)	0.743 (0.014)	0.732 (0.024)	0.741 (0.012)
Ground-truth		0.581 (0.004)	-0.000 (0.000)	-0.000 (0.000)	-0.000 (0.001)	-0.000 (0.000)	-0.000 (0.000)

Table S115. Averaged prediction accuracy (MAE) of RF and MIL models for 15 test sets of the PQC data set enthalpy prediction using Pmapper descriptors. The standard deviations are provided in parentheses.

Data set = PQC (Test sets)<br>Descs = Pmapper<br>y = Enthalpy (Hartree)		RF	MIL				
			Non-aggregation (Instance-Wrapper)	Bag-Wrapper	Instance-Net	Bag-Net	Bag-AttentionNet
		MAE					
Aggregation Method	Boltzmann Weight	0.076 (0.001)	0.075 (0.004)	0.073 (0.001)	0.074 (0.003)	0.074 (0.003)	0.074 (0.002)
	Mean	0.071 (0.001)	0.066 (0.005)	0.065 (0.002)	0.065 (0.004)	0.065 (0.004)	0.064 (0.003)
	Global Minimum	0.079 (0.001)	0.125 (0.001)	0.125 (0.001)	0.125 (0.001)	0.125 (0.001)	0.125 (0.001)
	Random	0.079 (0.001)	0.125 (0.001)	0.125 (0.001)	0.125 (0.001)	0.125 (0.001)	0.125 (0.001)
	RMSD max.	0.080 (0.001)	0.125 (0.001)	0.125 (0.001)	0.125 (0.001)	0.125 (0.001)	0.125 (0.001)
	RMSD min.	0.079 (0.000)	0.125 (0.001)	0.125 (0.001)	0.125 (0.001)	0.125 (0.001)	0.125 (0.001)
Non-Aggregation		0.069 (0.001)	0.057 (0.001)	0.062 (0.004)	0.060 (0.002)	0.062 (0.003)	0.061 (0.002)
Ground-truth		0.078 (0.001)	0.125 (0.001)	0.125 (0.001)	0.125 (0.001)	0.125 (0.001)	0.125 (0.001)

Table S116. Averaged prediction accuracy (RMSE) of RF and MIL models for 15 test sets of the PQC data set enthalpy prediction using Pmapper descriptors. The standard deviations are provided in parentheses.

Data set = PQC (Test sets)<br>Descs = Pmapper<br>y = Enthalpy (Hartree)		RF	MIL				
			Non-aggregation (Instance-Wrapper)	Bag-Wrapper	Instance-Net	Bag-Net	Bag-AttentionNet
		RMSE					
Aggregation Method	Boltzmann Weight	0.102 (0.001)	0.099 (0.004)	0.097 (0.002)	0.099 (0.004)	0.099 (0.004)	0.098 (0.002)
	Mean	0.097 (0.001)	0.088 (0.006)	0.087 (0.003)	0.088 (0.005)	0.088 (0.004)	0.087 (0.004)
	Global Minimum	0.106 (0.001)	0.162 (0.001)	0.162 (0.001)	0.162 (0.001)	0.162 (0.001)	0.162 (0.001)
	Random	0.105 (0.001)	0.162 (0.001)	0.162 (0.001)	0.162 (0.001)	0.162 (0.001)	0.162 (0.001)
	RMSD max.	0.107 (0.001)	0.162 (0.001)	0.162 (0.001)	0.162 (0.001)	0.162 (0.001)	0.162 (0.001)
	RMSD min.	0.105 (0.001)	0.162 (0.001)	0.162 (0.001)	0.162 (0.001)	0.162 (0.001)	0.162 (0.001)
Non-Aggregation		0.094 (0.001)	0.078 (0.002)	0.084 (0.004)	0.082 (0.002)	0.084 (0.004)	0.082 (0.002)
Ground-truth		0.105 (0.001)	0.162 (0.001)	0.162 (0.001)	0.162 (0.001)	0.162 (0.001)	0.162 (0.001)

Table S117. Averaged prediction accuracy (R^2) of RF and MIL models for 15 test sets of the PQC data set enthalpy prediction using 3D-MoRSE descriptors. The standard deviations are provided in parentheses.

Data set = PQC (Test sets)<br>Descs = 3D-MoRSE<br>y = Enthalpy (Hartree)		RF	MIL				
			Non-aggregation (Instance-Wrapper)	Bag-Wrapper	Instance-Net	Bag-Net	Bag-AttentionNet
		R^2					
Aggregation Method	Boltzmann Weight	0.770 (0.004)	0.927 (0.013)	0.909 (0.044)	0.917 (0.032)	0.929 (0.023)	0.922 (0.021)
	Mean	0.816 (0.003)	0.947 (0.013)	0.940 (0.024)	0.938 (0.021)	0.943 (0.012)	0.941 (0.013)
	Global Minimum	0.745 (0.004)	0.911 (0.042)	0.907 (0.029)	0.907 (0.032)	0.916 (0.025)	0.905 (0.035)
	Random	0.746 (0.004)	0.916 (0.035)	0.916 (0.029)	0.924 (0.016)	0.924 (0.015)	0.926 (0.020)
	RMSD max.	0.739 (0.003)	0.918 (0.019)	0.915 (0.019)	0.912 (0.027)	0.912 (0.025)	0.908 (0.042)
	RMSD min.	0.752 (0.003)	0.924 (0.019)	0.918 (0.020)	0.916 (0.028)	0.902 (0.041)	0.921 (0.031)
Non-Aggregation		0.809 (0.006)	0.959 (0.015)	0.936 (0.024)	0.928 (0.028)	0.938 (0.012)	0.934 (0.022)
Ground-truth		0.740 (0.003)	0.914 (0.036)	0.903 (0.047)	0.907 (0.059)	0.912 (0.044)	0.911 (0.033)

Table S118. Averaged prediction accuracy (MAE) of RF and MIL models for 15 test sets of the PQC data set enthalpy prediction using 3D-MoRSE descriptors. The standard deviations are provided in parentheses.

Data set = PQC (Test sets)<br>Descs = 3D-MoRSE<br>y = Enthalpy (Hartree)		RF	MIL				
			Non-aggregation (Instance-Wrapper)	Bag-Wrapper	Instance-Net	Bag-Net	Bag-AttentionNet
		MAE					
Aggregation Method	Boltzmann Weight	0.057 (0.001)	0.033 (0.004)	0.037 (0.011)	0.035 (0.008)	0.032 (0.007)	0.034 (0.006)
	Mean	0.050 (0.000)	0.027 (0.004)	0.030 (0.007)	0.030 (0.006)	0.029 (0.004)	0.030 (0.004)
	Global Minimum	0.061 (0.001)	0.036 (0.009)	0.038 (0.008)	0.038 (0.008)	0.035 (0.006)	0.038 (0.009)
	Random	0.061 (0.001)	0.035 (0.008)	0.036 (0.008)	0.034 (0.004)	0.034 (0.004)	0.033 (0.006)
	RMSD max.	0.062 (0.000)	0.035 (0.005)	0.036 (0.005)	0.037 (0.007)	0.037 (0.007)	0.037 (0.010)
	RMSD min.	0.060 (0.001)	0.034 (0.005)	0.035 (0.005)	0.035 (0.007)	0.039 (0.010)	0.034 (0.008)
Non-Aggregation		0.052 (0.001)	0.024 (0.006)	0.031 (0.007)	0.033 (0.008)	0.030 (0.004)	0.031 (0.006)
Ground-truth		0.061 (0.001)	0.036 (0.009)	0.039 (0.011)	0.037 (0.013)	0.036 (0.011)	0.037 (0.009)

Table S119. Averaged prediction accuracy (RMSE) of RF and MIL models for 15 test sets of the PQC data set enthalpy prediction using 3D-MoRSE descriptors. The standard deviations are provided in parentheses.

Data set = PQC (Test sets)<br>Descs = 3D-MoRSE<br>y = Enthalpy (Hartree)		RF	MIL				
			Non-aggregation (Instance-Wrapper)	Bag-Wrapper	Instance-Net	Bag-Net	Bag-AttentionNet
		RMSE					
Aggregation Method	Boltzmann Weight	0.078 (0.001)	0.043 (0.004)	0.048 (0.011)	0.046 (0.008)	0.043 (0.006)	0.045 (0.006)
	Mean	0.069 (0.001)	0.037 (0.004)	0.039 (0.007)	0.040 (0.006)	0.038 (0.004)	0.039 (0.004)
	Global Minimum	0.082 (0.001)	0.047 (0.010)	0.049 (0.008)	0.048 (0.008)	0.046 (0.007)	0.049 (0.009)
	Random	0.081 (0.001)	0.046 (0.009)	0.046 (0.008)	0.044 (0.004)	0.044 (0.004)	0.044 (0.005)
	RMSD max.	0.082 (0.001)	0.046 (0.005)	0.047 (0.005)	0.047 (0.007)	0.047 (0.006)	0.048 (0.010)
	RMSD min.	0.081 (0.001)	0.044 (0.005)	0.046 (0.005)	0.046 (0.007)	0.050 (0.010)	0.045 (0.008)
Non-Aggregation		0.071 (0.001)	0.032 (0.005)	0.040 (0.007)	0.043 (0.008)	0.040 (0.004)	0.041 (0.006)
Ground-truth		0.082 (0.001)	0.047 (0.009)	0.049 (0.011)	0.048 (0.013)	0.047 (0.010)	0.048 (0.008)

Table S120. Averaged prediction accuracy (R^2) of RF and MIL models for 15 test sets of the PQC data set enthalpy prediction using MBTR descriptors. The standard deviations are provided in parentheses.

Data set = PQC (Test sets) Descs = MBTR y = Enthalpy (Hartree)		RF	MIL				
			Non-aggregation (Instance-Wrapper)	Bag-Wrapper	Instance-Net	Bag-Net	Bag-AttentionNet
		R^2					
Aggregation Method	Boltzmann Weight	0.968 (0.001)	-6.349 (16.752)	-7.357 (18.315)	-9.552 (23.726)	-7.492 (19.472)	-6.602 (17.620)
	Mean	0.970 (0.001)	-119.909 (263.739)	-110.958 (236.435)	-85.757 (195.190)	-138.150 (300.929)	-87.792 (187.280)
	Global Minimum	0.966 (0.004)	-3.585 (9.573)	-6.148 (15.500)	-5.922 (14.849)	-6.008 (17.015)	-3.413 (9.261)
	Random	0.966 (0.001)	-92.593 (198.305)	-112.316 (234.700)	-149.713 (339.548)	-136.493 (337.644)	-167.964 (351.969)
	RMSD max.	0.966 (0.001)	-11.944 (27.763)	-9.670 (23.244)	-8.724 (21.613)	-8.452 (20.397)	-6.707 (16.031)
	RMSD min.	0.966 (0.001)	0.743 (0.536)	0.757 (0.542)	0.725 (0.574)	0.750 (0.526)	0.676 (0.673)
Non-Aggregation		0.976 (0.001)	0.664 (0.679)	0.582 (0.904)	0.675 (0.650)	0.647 (0.697)	0.612 (0.830)
Ground-truth		0.966 (0.001)	0.975 (0.005)	0.977 (0.004)	0.977 (0.003)	0.976 (0.004)	0.977 (0.004)

Table S121. Averaged prediction accuracy (MAE) of RF and MIL models for 15 test sets of the PQC data set enthalpy prediction using MBTR descriptors. The standard deviations are provided in parentheses.

Data set = PQC (Test sets)<br>Descs = MBTR<br>y = Enthalpy (Hartree)		RF	MIL				
			Non-aggregation (Instance-Wrapper)	Bag-Wrapper	Instance-Net	Bag-Net	Bag-AttentionNet
		MAE					
Aggregation Method	Boltzmann Weight	0.019 (0.000)	0.020 (0.006)	0.019 (0.004)	0.019 (0.004)	0.018 (0.004)	0.020 (0.005)
	Mean	0.018 (0.000)	0.021 (0.011)	0.020 (0.011)	0.020 (0.010)	0.021 (0.014)	0.020 (0.010)
	Global Minimum	0.020 (0.001)	0.018 (0.003)	0.019 (0.003)	0.019 (0.004)	0.018 (0.003)	0.018 (0.002)
	Random	0.019 (0.000)	0.023 (0.014)	0.023 (0.012)	0.024 (0.014)	0.024 (0.013)	0.025 (0.016)
	RMSD max.	0.020 (0.000)	0.018 (0.004)	0.019 (0.005)	0.019 (0.005)	0.019 (0.003)	0.020 (0.005)
	RMSD min.	0.019 (0.000)	0.017 (0.002)	0.018 (0.003)	0.018 (0.003)	0.017 (0.002)	0.018 (0.003)
Non-Aggregation		0.015 (0.000)	0.017 (0.003)	0.015 (0.001)	0.017 (0.004)	0.017 (0.004)	0.017 (0.002)
Ground-truth		0.019 (0.000)	0.019 (0.003)	0.018 (0.002)	0.017 (0.002)	0.018 (0.002)	0.018 (0.002)

Table S122. Averaged prediction accuracy (RMSE) of RF and MIL models for 15 test sets of the PQC data set enthalpy prediction using MBTR descriptors. The standard deviations are provided in parentheses.

Data set = PQC (Test sets)<br>Descs = MBTR<br>y = Enthalpy (Hartree)		RF	MIL				
			Non-aggregation (Instance-Wrapper)	Bag-Wrapper	Instance-Net	Bag-Net	Bag-AttentionNet
		RMSE					
Aggregation Method	Boltzmann Weight	0.029 (0.001)	0.211 (0.397)	0.225 (0.424)	0.249 (0.478)	0.224 (0.429)	0.214 (0.404)
	Mean	0.028 (0.001)	0.802 (1.640)	0.780 (1.579)	0.678 (1.390)	0.861 (1.760)	0.696 (1.402)
	Global Minimum	0.030 (0.002)	0.173 (0.310)	0.211 (0.391)	0.208 (0.385)	0.203 (0.389)	0.170 (0.304)
	Random	0.030 (0.000)	0.716 (1.443)	0.790 (1.585)	0.887 (1.834)	0.836 (1.758)	0.959 (1.938)
	RMSD max.	0.030 (0.000)	0.277 (0.531)	0.253 (0.482)	0.240 (0.458)	0.239 (0.451)	0.220 (0.405)
	RMSD min.	0.030 (0.001)	0.053 (0.064)	0.052 (0.062)	0.055 (0.066)	0.053 (0.063)	0.059 (0.073)
Non-Aggregation		0.025 (0.000)	0.059 (0.076)	0.062 (0.087)	0.058 (0.074)	0.060 (0.078)	0.061 (0.083)
Ground-truth		0.030 (0.001)	0.025 (0.003)	0.024 (0.002)	0.024 (0.002)	0.025 (0.002)	0.025 (0.002)

Table S123. Averaged prediction accuracy (R^2) of RF and MIL models for 15 test sets of the PQC data set six property predictions using ECFP4 count. The standard deviations are provided in parentheses.

Data set = PQC (Test sets) Descs = ECFP4 count		Dipole moment	HOMO	HOMO-LUMO gap	LUMO	Energy	Enthalpy
		R^2					
MIL	Non-aggregation	0.326 (0.014)	0.863 (0.006)	0.937 (0.001)	0.962 (0.001)	0.994 (0.000)	0.994 (0.000)
	Bag-Wrapper	0.317 (0.016)	0.864 (0.003)	0.937 (0.002)	0.963 (0.001)	0.994 (0.000)	0.994 (0.000)
	Instance-Net	0.318 (0.014)	0.864 (0.004)	0.937 (0.002)	0.962 (0.001)	0.994 (0.000)	0.994 (0.000)
	Bag-Net	0.323 (0.014)	0.864 (0.004)	0.937 (0.002)	0.962 (0.001)	0.994 (0.000)	0.994 (0.000)
	Bag-AttentionNet	0.317 (0.014)	0.864 (0.003)	0.937 (0.002)	0.962 (0.002)	0.994 (0.000)	0.994 (0.000)
RF		0.516 (0.006)	0.867 (0.005)	0.939 (0.001)	0.956 (0.001)	0.960 (0.002)	0.941 (0.002)

Table S124. Averaged prediction accuracy (MAE) of RF and MIL models for 15 test sets of the PQC data set six property predictions using ECFP4 count. The standard deviations are provided in parentheses.

Data set = PQC (Test sets)<br>Descs = ECFP4 count		Dipole moment	HOMO	HOMO-LUMO gap	LUMO	Energy	Enthalpy
		MAE					
MIL	Non-aggregation	1.410 (0.011)	0.149 (0.003)	0.191 (0.002)	0.128 (0.001)	0.184 (0.005)	0.008 (0.000)
	Bag-Wrapper	1.419 (0.016)	0.148 (0.002)	0.191 (0.002)	0.127 (0.002)	0.185 (0.010)	0.008 (0.000)
	Instance-Net	1.417 (0.012)	0.148 (0.002)	0.191 (0.003)	0.127 (0.001)	0.186 (0.008)	0.008 (0.000)
	Bag-Net	1.412 (0.011)	0.149 (0.002)	0.192 (0.003)	0.128 (0.001)	0.185 (0.006)	0.008 (0.000)
	Bag-AttentionNet	1.417 (0.013)	0.148 (0.001)	0.192 (0.002)	0.128 (0.004)	0.185 (0.006)	0.008 (0.000)
RF		1.189 (0.009)	0.140 (0.004)	0.175 (0.001)	0.128 (0.001)	0.445 (0.007)	0.024 (0.000)

Table S125. Averaged prediction accuracy (RMSE) of RF and MIL models for 15 test sets of the PQC data set six property predictions using ECFP4 count. The standard deviations are provided in parentheses.

Data set = PQC (Test sets)<br>Descs = ECFP4 count		Dipole moment	HOMO	HOMO-LUMO gap	LUMO	Energy	Enthalpy
		RMSE					
MIL	Non-aggregation	1.910 (0.017)	0.204 (0.004)	0.269 (0.003)	0.178 (0.002)	0.311 (0.006)	0.012 (0.000)
	Bag-Wrapper	1.923 (0.022)	0.204 (0.002)	0.269 (0.003)	0.177 (0.002)	0.311 (0.009)	0.012 (0.000)
	Instance-Net	1.922 (0.017)	0.204 (0.003)	0.269 (0.004)	0.178 (0.001)	0.312 (0.006)	0.012 (0.000)
	Bag-Net	1.914 (0.015)	0.204 (0.003)	0.270 (0.003)	0.178 (0.002)	0.312 (0.004)	0.012 (0.000)
	Bag-AttentionNet	1.620 (0.011)	0.201 (0.004)	0.265 (0.003)	0.193 (0.003)	0.793 (0.016)	0.039 (0.001)
RF		1.620 (0.011)	0.201 (0.004)	0.265 (0.003)	0.193 (0.003)	0.793 (0.016)	0.039 (0.001)

Table S126. Averaged prediction accuracy (MAE) of MolCLR, GEM, and Uni-Mol models for 15 test sets of the PQC data set six property predictions. The standard deviations are provided in parentheses.

Data set = PQC (Test sets)		Dipole moment	HOMO	HOMO-LUMO gap	LUMO	Energy	Enthalpy
		MAE					
GEM	Ground-truth	1.243 (0.015)	0.120 (0.001)	0.155 (0.003)	0.119 (0.004)	0.167 (0.019)	0.007 (0.001)
	Global min.	1.281 (0.011)	0.134 (0.002)	0.168 (0.003)	0.129 (0.005)	0.169 (0.025)	0.007 (0.001)
	RMSD max.	1.286 (0.014)	0.135 (0.003)	0.170 (0.004)	0.132 (0.005)	0.169 (0.019)	0.007 (0.001)
	Non-aggregation	1.239 (0.009)	0.121 (0.002)	0.150 (0.003)	0.114 (0.003)	0.146 (0.012)	0.006 (0.001)
Uni-Mol	Ground-truth	0.336 (0.003)	0.039 (0.001)	0.069 (0.001)	0.042 (0.001)	0.118 (0.011)	0.004 (0.000)
	Global min.	1.164 (0.006)	0.099 (0.001)	0.133 (0.002)	0.095 (0.001)	0.133 (0.009)	0.005 (0.000)
	RMSD max.	1.161 (0.007)	0.097 (0.001)	0.131 (0.002)	0.094 (0.001)	0.130 (0.005)	0.004 (0.000)
MolCLR		1.203 (0.010)	0.111 (0.001)	0.144 (0.001)	0.101 (0.001)	0.327 (0.008)	0.015 (0.000)

Table S127. Averaged prediction accuracy (RMSE) of MolCLR, GEM, and Uni-Mol models for 15 test sets of the PQC data set six property predictions. The standard deviations are provided in parentheses.

Data set = PQC (Test sets)		Dipole moment	HOMO	HOMO-LUMO gap	LUMO	Energy	Enthalpy
		RMSE					
GEM	Ground-truth	1.648 (0.021)	0.159 (0.002)	0.212 (0.005)	0.157 (0.005)	0.242 (0.019)	0.010 (0.001)
	Global min.	1.691 (0.017)	0.177 (0.003)	0.228 (0.004)	0.168 (0.006)	0.248 (0.023)	0.010 (0.001)
	RMSD max.	1.699 (0.021)	0.178 (0.004)	0.229 (0.004)	0.171 (0.006)	0.245 (0.023)	0.010 (0.001)
	Non-aggregation	1.645 (0.012)	0.161 (0.003)	0.206 (0.004)	0.151 (0.004)	0.211 (0.014)	0.008 (0.001)
Uni-Mol	Ground-truth	0.602 (0.017)	0.066 (0.003)	0.203 (0.004)	0.107 (0.002)	0.455 (0.038)	0.014 (0.001)
	Global min.	1.571 (0.011)	0.137 (0.002)	0.254 (0.003)	0.152 (0.002)	0.459 (0.037)	0.014 (0.001)
	RMSD max.	1.566 (0.011)	0.135 (0.001)	0.252 (0.004)	0.150 (0.001)	0.467 (0.033)	0.014 (0.001)
MolCLR		1.626 (0.015)	0.152 (0.002)	0.207 (0.002)	0.138 (0.002)	0.584 (0.018)	0.024 (0.001)

Table S128. Averaged prediction accuracy (R^2) of RF and MIL models for 25 test sets of the MP data set property (melting point) prediction using MOE descriptors. The standard deviations are provided in parentheses.

Data set = MP (Test sets)<br>Descs = MOE<br>y = Melting Point (degrees Celsius)		RF	MIL				
			Non-aggregation (Instance-Wrapper)	Bag-Wrapper	Instance-Net	Bag-Net	Bag-AttentionNet
		R^2					
Aggregation Method	Boltzmann Weight	0.759 (0.078)	-242.832 (645.763)	-205.327 (851.506)	-197.022 (470.222)	-154.182 (417.812)	-314.314 (1017.445)
	Mean	0.747 (0.093)	0.631 (0.158)	0.651 (0.161)	0.644 (0.167)	0.628 (0.183)	0.650 (0.143)
	Global Minimum	0.764 (0.075)	0.495 (0.344)	0.517 (0.307)	0.502 (0.442)	0.517 (0.357)	0.494 (0.449)
	Random	0.735 (0.088)	0.557 (0.215)	0.561 (0.196)	0.569 (0.193)	0.559 (0.214)	0.583 (0.179)
Non-Aggregation		0.716 (0.113)	0.724 (0.102)	0.674 (0.118)	0.686 (0.124)	0.705 (0.107)	0.672 (0.111)

Table S129. Averaged prediction accuracy (MAE) of RF and MIL models for 25 test sets of the MP data set property (melting point) prediction using MOE descriptors. The standard deviations are provided in parentheses.

Data set = MP (Test sets)<br>Descs = MOE<br>y = Melting Point (degrees Celsius)		RF	MIL				
			Non-aggregation (Instance-Wrapper)	Bag-Wrapper	Instance-Net	Bag-Net	Bag-AttentionNet
		MAE					
Aggregation Method	Boltzmann Weight	20.695 (2.357)	82.300 (123.572)	63.518 (120.328)	81.722 (124.000)	68.939 (103.783)	82.344 (133.875)
	Mean	20.836 (2.683)	24.402 (3.492)	23.654 (3.575)	23.991 (3.621)	24.162 (3.394)	23.928 (3.364)
	Global Minimum	20.753 (2.268)	27.266 (4.566)	27.015 (4.063)	26.887 (4.876)	26.751 (5.029)	26.871 (4.571)
	Random	21.843 (2.769)	27.055 (3.499)	27.316 (3.222)	26.975 (3.306)	27.177 (3.560)	26.854 (3.477)
Non-Aggregation		21.144 (2.885)	19.931 (3.675)	23.535 (3.384)	22.678 (3.267)	22.114 (3.172)	24.200 (3.026)

Table S130. Averaged prediction accuracy (RMSE) of RF and MIL models for 25 test sets of the MP data set property (melting point) prediction using MOE descriptors. The standard deviations are provided in parentheses.

Data set = MP (Test sets)<br>Descs = MOE<br>y = Melting Point (degrees Celsius)		RF	MIL				
			Non-aggregation (Instance-Wrapper)	Bag-Wrapper	Instance-Net	Bag-Net	Bag-AttentionNet
		RMSE					
Aggregation Method	Boltzmann Weight	30.013 (4.113)	380.119 (755.213)	265.373 (739.467)	375.424 (738.845)	296.440 (634.705)	378.838 (817.156)
	Mean	30.684 (4.879)	36.688 (6.674)	35.480 (6.879)	35.954 (7.510)	36.749 (8.551)	35.880 (6.860)
	Global Minimum	29.732 (3.956)	42.030 (11.031)	41.293 (10.010)	41.065 (12.444)	40.790 (11.794)	41.435 (13.047)
	Random	31.575 (4.902)	40.069 (8.516)	40.079 (7.952)	39.613 (7.702)	40.021 (7.979)	39.074 (6.942)
Non-Aggregation		32.587 (6.522)	32.056 (5.943)	34.830 (5.665)	34.089 (6.225)	33.062 (5.773)	34.954 (5.221)

Table S131. Averaged prediction accuracy (R^2) of RF and MIL models for 25 test sets of the MP data set property (melting point) prediction using Pmapper descriptors. The standard deviations are provided in parentheses.

Data set = MP (Test sets)<br>Descs = Pmapper<br>y = Melting Point (degrees Celsius)		RF	MIL				
			Non-aggregation (Instance-Wrapper)	Bag-Wrapper	Instance-Net	Bag-Net	Bag-AttentionNet
		R^2					
Aggregation Method	Boltzmann Weight	0.452 (0.139)	-0.174 (0.651)	-0.146 (0.665)	-0.213 (0.697)	-0.205 (0.674)	-0.181 (0.638)
	Mean	0.480 (0.139)	-0.074 (0.415)	-0.134 (0.418)	-0.164 (0.504)	-0.029 (0.344)	-0.051 (0.368)
	Global Minimum	0.436 (0.157)	-0.656 (3.019)	-0.633 (2.925)	-0.614 (2.822)	-0.642 (2.883)	-0.633 (2.888)
	Random	0.430 (0.147)	-0.109 (0.447)	-0.098 (0.406)	-0.106 (0.430)	-0.087 (0.380)	-0.084 (0.381)
Non-Aggregation		0.425 (0.156)	0.440 (0.200)	0.033 (0.467)	0.036 (0.305)	-0.017 (0.331)	0.368 (0.234)

Table S132. Averaged prediction accuracy (MAE) of RF and MIL models for 25 test sets of the MP data set property (melting point) prediction using Pmapper descriptors. The standard deviations are provided in parentheses.

Data set = MP (Test sets)<br>Descs = Pmapper<br>y = Melting Point (degrees Celsius)		RF	MIL				
			Non-aggregation (Instance-Wrapper)	Bag-Wrapper	Instance-Net	Bag-Net	Bag-AttentionNet
		MAE					
Aggregation Method	Boltzmann Weight	34.881 (3.715)	46.332 (7.903)	46.092 (8.206)	46.640 (7.586)	46.391 (7.334)	46.477 (7.300)
	Mean	32.959 (3.822)	42.537 (7.804)	43.242 (7.717)	43.709 (7.406)	41.985 (7.332)	42.334 (7.581)
	Global Minimum	35.075 (4.334)	49.140 (13.721)	49.220 (13.698)	49.163 (13.627)	49.291 (13.764)	49.243 (13.756)
	Random	35.093 (4.089)	47.154 (6.849)	47.087 (6.577)	47.181 (6.737)	46.992 (6.565)	47.064 (6.626)
Non-Aggregation		35.148 (3.413)	31.753 (4.936)	40.652 (8.803)	41.124 (7.334)	41.426 (7.719)	35.698 (4.624)

Table S133. Averaged prediction accuracy (RMSE) of RF and MIL models for 25 test sets of the MP data set property (melting point) prediction using Pmapper descriptors. The standard deviations are provided in parentheses.

Data set = MP (Test sets)<br>Descs = Pmapper<br>y = Melting Point (degrees Celsius)		RF	MIL				
			Non-aggregation (Instance-Wrapper)	Bag-Wrapper	Instance-Net	Bag-Net	Bag-AttentionNet
		RMSE					
Aggregation Method	Boltzmann Weight	45.620 (4.721)	64.921 (14.796)	63.983 (14.960)	65.786 (14.307)	65.644 (14.977)	65.342 (14.744)
	Mean	44.386 (4.858)	63.293 (13.784)	65.210 (14.059)	65.590 (14.364)	62.159 (11.534)	62.670 (12.519)
	Global Minimum	46.202 (5.141)	71.250 (41.009)	71.006 (40.281)	70.864 (39.458)	71.139 (40.016)	71.139 (40.016)
	Random	46.462 (4.848)	64.898 (14.846)	64.722 (14.063)	64.875 (14.503)	64.443 (13.571)	64.355 (13.539)
Non-Aggregation		46.495 (3.982)	45.578 (7.158)	59.793 (16.227)	60.259 (10.751)	62.046 (12.323)	48.358 (6.188)

Table S134. Averaged prediction accuracy (R^2) of RF and MIL models for 25 test sets of the MP data set property (melting point) prediction using 3D-MoRSE descriptors. The standard deviations are provided in parentheses.

Data set = MP (Test sets)<br>Descs = 3D-MoRSE<br>y = Melting Point (degrees Celsius)		RF	MIL				
			Non-aggregation (Instance-Wrapper)	Bag-Wrapper	Instance-Net	Bag-Net	Bag-AttentionNet
		R^2					
Aggregation Method	Boltzmann Weight	0.601 (0.151)	0.646 (0.109)	0.654 (0.118)	0.644 (0.122)	0.645 (0.126)	0.643 (0.133)
	Mean	0.618 (0.144)	0.712 (0.117)	0.703 (0.113)	0.711 (0.108)	0.708 (0.103)	0.713 (0.108)
	Global Minimum	0.595 (0.133)	0.608 (0.143)	0.617 (0.138)	0.604 (0.152)	0.626 (0.127)	0.615 (0.139)
	Random	0.559 (0.153)	0.591 (0.165)	0.597 (0.153)	0.594 (0.159)	0.607 (0.160)	0.597 (0.166)
Non-Aggregation		0.523 (0.195)	0.691 (0.122)	0.715 (0.101)	0.723 (0.101)	0.720 (0.107)	0.690 (0.109)

Table S135. Averaged prediction accuracy (MAE) of RF and MIL models for 25 test sets of the MP data set property (melting point) prediction using 3D-MoRSE descriptors. The standard deviations are provided in parentheses.

Data set = MP (Test sets)<br>Descs = 3D-MoRSE<br>y = Melting Point (degrees Celsius)		RF	MIL				
			Non-aggregation (Instance-Wrapper)	Bag-Wrapper	Instance-Net	Bag-Net	Bag-AttentionNet
		MAE					
Aggregation Method	Boltzmann Weight	28.253 (3.792)	25.425 (3.473)	25.220 (3.628)	25.603 (3.520)	25.652 (3.308)	25.512 (3.374)
	Mean	26.955 (3.640)	22.068 (3.409)	22.267 (3.350)	22.245 (3.703)	21.922 (3.375)	21.878 (3.738)
	Global Minimum	29.438 (3.732)	27.134 (3.813)	27.051 (3.942)	27.437 (4.239)	27.115 (3.795)	27.209 (4.002)
	Random	30.808 (3.932)	28.416 (4.127)	28.164 (4.243)	28.345 (4.377)	27.617 (4.295)	28.064 (4.357)
Non-Aggregation		31.953 (3.675)	21.356 (3.484)	22.688 (3.507)	22.032 (3.749)	22.046 (3.554)	24.145 (3.861)

Table S136. Averaged prediction accuracy (RMSE) of RF and MIL models for 25 test sets of the MP data set property (melting point) prediction using 3D-MoRSE descriptors. The standard deviations are provided in parentheses.

Data set = MP (Test sets)<br>Descs = 3D-MoRSE<br>y = Melting Point (degrees Celsius)		RF	MIL				
			Non-aggregation (Instance-Wrapper)	Bag-Wrapper	Instance-Net	Bag-Net	Bag-AttentionNet
		RMSE					
Aggregation Method	Boltzmann Weight	38.618 (5.584)	36.472 (4.359)	35.888 (4.873)	36.415 (4.483)	36.292 (4.617)	36.335 (4.688)
	Mean	37.650 (4.472)	32.606 (5.788)	33.163 (5.938)	32.853 (6.231)	33.021 (5.723)	32.673 (5.918)
	Global Minimum	39.050 (5.520)	38.164 (5.106)	37.816 (5.172)	38.478 (6.606)	37.452 (5.133)	37.908 (5.492)
	Random	40.571 (4.808)	38.650 (5.680)	38.477 (5.561)	38.583 (5.609)	37.894 (5.855)	38.335 (6.038)
Non-Aggregation		41.818 (5.072)	33.771 (5.139)	32.535 (5.395)	32.180 (6.042)	32.263 (5.762)	34.073 (5.299)

Table S137. Averaged prediction accuracy (R^2) of RF and MIL models for 25 test sets of the MP data set property (melting point) prediction using MBTR descriptors. The standard deviations are provided in parentheses.

Data set = MP (Test sets) Descs = MBTR y = Melting Point (degrees Celsius)		RF	MIL				
			Non-aggregation (Instance-Wrapper)	Bag-Wrapper	Instance-Net	Bag-Net	Bag-AttentionNet
			R^2				
Aggregation Method	Boltzmann Weight	0.584 (0.115)	-7165098737083.528 (19317595242150.559)	-11966441932864.590 (30918158493774.270)	-13847010827263.953 (34992374555407.730)	-10317852678437.828 (30645327727806.609)	-12528213401093.645 (32659234767763.434)
	Mean	0.621 (0.105)	-143401521347.409 (388142685616.117)	-272056301772.412 (605016642088.474)	-226633214244.099 (531963773691.735)	-118324970468.071 (454291604594.911)	-185848580345.569 (442584076192.239)
	Global Minimum	0.565 (0.132)	-628148346966.149 (2155873063931.699)	-704447761257.113 (2431237753335.554)	-594145961114.222 (1896965012092.329)	-866654244218.489 (2975254352379.127)	-422296551446.357 (1922604608526.179)
	Random	0.577 (0.096)	-414346400370.205 (1689685009510.833)	-919365769003.414 (3009282586415.008)	-1337165689960.647 (4798636277621.771)	-298645903420.383 (1052757278278.989)	-106363014699.195 (418246135155.466)
Non-Aggregation		0.619 (0.166)	-291021128.892 (1006180228.711)	-139065188.832 (328815256.637)	-100516000.118 (284106413.246)	-178362035.908 (449310267.213)	-112751402.103 (371690244.214)

Table S138. Averaged prediction accuracy (MAE) of RF and MIL models for 25 test sets of the MP data set property (melting point) prediction using MBTR descriptors. The standard deviations are provided in parentheses.

Data set = MP (Test sets)<br>Descs = MBTR<br>y = Melting Point (degrees Celsius)		RF	MIL				
			Non-aggregation (Instance-Wrapper)	Bag-Wrapper	Instance-Net	Bag-Net	Bag-AttentionNet
		MAE					
Aggregation Method	Boltzmann Weight	27.045 (3.482)	11599209.995 (28302425.918)	15215226.358 (35797841.079)	17152074.557 (37446182.789)	13157284.205 (33068848.599)	16361685.289 (35329530.382)
	Mean	25.527 (3.671)	1667215.406 (3859611.713)	2502843.855 (5262170.235)	2232282.021 (4724657.449)	1251479.489 (3779788.514)	1999621.572 (4306051.826)
	Global Minimum	28.432 (3.484)	2638920.221 (8099524.993)	2898060.525 (8752442.704)	2683856.929 (7917973.491)	3071023.223 (9538278.506)	1897965.704 (7020061.425)
	Random	28.423 (3.721)	2012478.791 (6508874.308)	3277225.726 (9836318.842)	3736624.246 (11809257.061)	1844853.194 (5709929.660)	1063596.256 (3466362.765)
Non-Aggregation		23.389 (3.877)	67316.086 (178356.762)	54814.310 (121193.367)	43559.918 (106075.324)	60999.532 (138006.747)	43627.834 (104455.731)

Table S139. Averaged prediction accuracy (RMSE) of RF and MIL models for 25 test sets of the MP data set property (melting point) prediction using MBTR descriptors. The standard deviations are provided in parentheses.

Data set = MP (Test sets)<br>Descs = MBTR<br>y = Melting Point (degrees Celsius)		RF	MIL				
			Non-aggregation (Instance-Wrapper)	Bag-Wrapper	Instance-Net	Bag-Net	Bag-AttentionNet
			RMSE				
Aggregation Method	Boltzmann Weight	39.656 (4.766)	72387842.585 (176608815.205)	94977786.390 (223483503.756)	107090386.778 (233809848.613)	82143393.480 (206478664.841)	102107680.871 (220513034.155)
	Mean	37.905 (5.022)	10411139.759 (24102113.762)	15629605.076 (32861479.113)	13939947.508 (29504460.109)	7814824.161 (23603932.130)	12486915.321 (26889789.033)
	Global Minimum	40.488 (4.981)	16479107.443 (50580896.746)	18097488.858 (54658065.362)	16759429.334 (49446407.234)	19177729.300 (59566037.044)	11852173.791 (43839744.411)
	Random	40.215 (4.688)	12567300.347 (40647435.342)	20465625.377 (61427330.055)	23334577.427 (73748438.067)	11520475.258 (35658132.389)	6641511.480 (21646718.506)
Non-Aggregation		37.401 (6.812)	420285.920 (1113817.804)	342185.426 (756802.708)	271906.203 (662401.963)	380815.459 (861810.605)	272331.534 (652301.681)

Table S140. Averaged prediction accuracy (R^2, MAE, RMSE) of RF and MIL models for 25 test sets of the MP data set property (melting point) prediction using ECFP4 count. The standard deviations are provided in parentheses.

Data set = MP (Test sets)<br>Descs = ECFP4 count<br>y = Melting Point (degrees Celsius)		R^2	MAE	RMSE
MIL	Non-aggregation	28.609 (5.226)	48.405 (9.187)	0.372 (0.257)
	Bag-Wrapper	28.272 (4.872)	47.837 (8.829)	0.390 (0.228)
	Instance-Net	28.151 (5.200)	48.227 (8.790)	0.378 (0.241)
	Bag-Net	28.523 (5.220)	48.433 (8.801)	0.372 (0.246)
	Bag-AttentionNet	28.437 (5.297)	48.276 (9.225)	0.381 (0.228)
RF		0.638 (0.100)	23.838 (3.332)	37.234 (6.445)

Table S141. Averaged prediction accuracy (R^2, MAE, RMSE) of MolCLR, GEM, and Uni-Mol models for 25 test sets of the MP data set property (melting point) prediction. The standard deviations are provided in parentheses.

Data set = MP (Test sets)<br>y = Melting Point (degrees Celsius)		R^2	MAE	RMSE
GEM	Global min.	0.732 (0.120)	19.414 (3.466)	31.298 (6.412)
	Non-aggregation	0.734 (0.132)	17.331 (3.529)	31.311 (7.503)
Uni-Mol	Global min.	0.780 (0.078)	19.623 (2.396)	28.659 (4.260)
	Non-aggregation	0.755 (0.089)	16.786 (2.477)	30.229 (5.797)
MolCLR		0.632 (0.149)	23.942 (4.032)	37.220 (7.510)

Table S142. Averaged prediction accuracy (R^2) of RF, Elastic Net, PLS, SVM, and MIL models for 25 test sets of the APTC-1 data set property ($\Delta\Delta G^{\ddagger}$) prediction using MOE descriptors. The standard deviations are provided in parentheses.

Data set = APTC-1 (Test sets)<br>Descs = MOE<br>y = $\Delta\Delta G^{\ddagger}$(kcal/mol)		RF	Elastic Net	PLS	SVM	MIL				
						Non-aggregation (Instance-Wrapper)	Bag-Wrapper	Instance-Net	Bag-Net	Bag-AttentionNet
		R^2								
Aggregation Method	Boltzmann Weight	0.271 (0.247)	-0.092 (0.175)	0.192 (0.341)	0.160 (0.266)	-0.036 (0.610)	-0.013 (0.574)	-0.039 (0.604)	-0.008 (0.608)	-0.027 (0.612)
	Mean	0.302 (0.274)	-0.092 (0.175)	0.266 (0.315)	0.220 (0.288)	0.099 (0.364)	0.116 (0.378)	0.102 (0.386)	0.119 (0.375)	0.109 (0.365)
	Global Minimum	0.203 (0.262)	-0.092 (0.175)	0.113 (0.345)	0.109 (0.302)	-0.120 (0.530)	-0.142 (0.602)	-0.125 (0.582)	-0.157 (0.613)	-0.141 (0.590)
	Random	0.346 (0.200)	-0.092 (0.175)	0.127 (0.310)	0.032 (0.285)	-0.310 (0.479)	-0.286 (0.469)	-0.252 (0.482)	-0.255 (0.476)	-0.263 (0.450)
Non-Aggregation		0.094 (0.335)	-0.092 (0.175)	0.333 (0.245)	0.249 (0.260)	0.234 (0.350)	0.211 (0.293)	0.257 (0.290)	0.203 (0.412)	0.245 (0.328)

Table S143. Averaged prediction accuracy (MAE) of RF, Elastic Net, PLS, SVM, and MIL models for 25 test sets of the APTC-1 data set property ($\Delta\Delta G^{\ddagger}$) prediction using MOE descriptors. The standard deviations are provided in parentheses.

Data set = APTC-1 (Test sets)<br>Descs = MOE<br>y = $\Delta\Delta G^{\ddagger}$(kcal/mol)		RF	Elastic Net	PLS	SVM	MIL				
						Non-aggregation (Instance-Wrapper)	Bag-Wrapper	Instance-Net	Bag-Net	Bag-AttentionNet
		MAE								
Aggregation Method	Boltzmann Weight	0.294 (0.059)	0.394 (0.050)	0.319 (0.069)	0.321 (0.057)	0.331 (0.075)	0.326 (0.071)	0.332 (0.073)	0.327 (0.079)	0.327 (0.080)
	Mean	0.283 (0.054)	0.394 (0.050)	0.296 (0.062)	0.297 (0.061)	0.307 (0.062)	0.298 (0.061)	0.307 (0.062)	0.299 (0.064)	0.302 (0.058)
	Global Minimum	0.304 (0.053)	0.394 (0.050)	0.337 (0.064)	0.327 (0.060)	0.349 (0.062)	0.355 (0.068)	0.354 (0.063)	0.356 (0.068)	0.352 (0.069)
	Random	0.276 (0.045)	0.394 (0.050)	0.325 (0.053)	0.352 (0.054)	0.383 (0.061)	0.379 (0.063)	0.379 (0.066)	0.377 (0.068)	0.376 (0.062)
Non-Aggregation		0.306 (0.064)	0.394 (0.050)	0.283 (0.054)	0.285 (0.054)	0.279 (0.065)	0.295 (0.065)	0.288 (0.060)	0.295 (0.066)	0.286 (0.075)

Table S144.Averaged prediction accuracy (RMSE) of RF, Elastic Net, PLS, SVM, and MIL models for 25 test sets of the APTC-1 data set property ($\Delta\Delta G^{\ddagger}$) prediction using MOE descriptors. The standard deviations are provided in parentheses.

Data set = APTC-1 (Test sets)<br>Descs = MOE<br>y = $\Delta\Delta G^{\ddagger}$(kcal/mol)		RF	Elastic Net	PLS	SVM	MIL				
						Non-aggregation (Instance-Wrapper)	Bag-Wrapper	Instance-Net	Bag-Net	Bag-AttentionNet
		RMSE								
Aggregation Method	Boltzmann Weight	0.371 (0.067)	0.461 (0.062)	0.384 (0.068)	0.397 (0.065)	0.423 (0.093)	0.420 (0.088)	0.423 (0.089)	0.416 (0.093)	0.419 (0.096)
	Mean	0.360 (0.064)	0.461 (0.062)	0.368 (0.069)	0.381 (0.071)	0.406 (0.075)	0.401 (0.076)	0.404 (0.077)	0.400 (0.078)	0.403 (0.069)
	Global Minimum	0.388 (0.062)	0.461 (0.062)	0.405 (0.066)	0.408 (0.068)	0.447 (0.074)	0.450 (0.080)	0.447 (0.077)	0.452 (0.082)	0.449 (0.077)
	Random	0.354 (0.065)	0.461 (0.062)	0.402 (0.063)	0.428 (0.060)	0.493 (0.076)	0.488 (0.076)	0.481 (0.081)	0.483 (0.083)	0.485 (0.078)
Non-Aggregation		0.413 (0.083)	0.461 (0.062)	0.352 (0.060)	0.375 (0.067)	0.373 (0.079)	0.383 (0.075)	0.371 (0.076)	0.380 (0.079)	0.372 (0.088)

Table S145.Averaged prediction accuracy (R^2) of RF, Elastic Net, PLS, SVM, and MIL models for 25 test sets of the APTC-1 data set property ($\Delta\Delta G^{‡}$) prediction using Pmapper descriptors. The standard deviations are provided in parentheses.

Data set = APTC-1 (Test sets)<br>Descs = Pmapper<br>y = $\Delta\Delta G^{‡}$(kcal/mol)		RF	Elastic Net	PLS	SVM	MIL				
						Non-aggregation (Instance-Wrapper)	Bag-Wrapper	Instance-Net	Bag-Net	Bag-AttentionNet
		R^2								
Aggregation Method	Boltzmann Weight	0.355 (0.249)	-0.092 (0.175)	0.296 (0.389)	0.197 (0.188)	0.204 (0.381)	0.213 (0.380)	0.216 (0.375)	0.200 (0.380)	0.215 (0.367)
	Mean	0.440 (0.157)	-0.092 (0.175)	0.478 (0.183)	0.440 (0.148)	0.576 (0.144)	0.571 (0.163)	0.571 (0.160)	0.573 (0.152)	0.564 (0.163)
	Global Minimum	0.329 (0.239)	-0.092 (0.175)	0.339 (0.279)	0.132 (0.172)	0.180 (0.273)	0.197 (0.240)	0.173 (0.230)	0.171 (0.260)	0.175 (0.244)
	Random	0.405 (0.170)	-0.092 (0.175)	0.176 (0.228)	0.134 (0.189)	0.172 (0.333)	0.198 (0.319)	0.175 (0.348)	0.173 (0.315)	0.175 (0.348)
Non-Aggregation		0.508 (0.230)	-0.092 (0.175)	0.517 (0.177)	0.602 (0.132)	0.723 (0.144)	0.642 (0.168)	0.657 (0.159)	0.661 (0.162)	0.662 (0.149)

Table S146. Averaged prediction accuracy (MAE) of RF, Elastic Net, PLS, SVM, and MIL models for 25 test sets of the APTC-1 data set property ($\Delta\Delta G^{\ddagger}$) prediction using Pmapper descriptors. The standard deviations are provided in parentheses.

Data set = APTC-1 (Test sets) Descs = Pmapper y = $\Delta\Delta G^{\ddagger}$(kcal/mol)		RF	Elastic Net	PLS	SVM	MIL				
						Non-aggregation (Instance-Wrapper)	Bag-Wrapper	Instance-Net	Bag-Net	Bag-AttentionNet
		MAE								
Aggregation Method	Boltzmann Weight	0.288 (0.046)	0.394 (0.050)	0.291 (0.066)	0.327 (0.049)	0.292 (0.074)	0.291 (0.074)	0.290 (0.077)	0.292 (0.075)	0.290 (0.077)
	Mean	0.267 (0.052)	0.394 (0.050)	0.258 (0.044)	0.265 (0.046)	0.216 (0.051)	0.216 (0.055)	0.217 (0.052)	0.216 (0.052)	0.217 (0.055)
	Global Minimum	0.291 (0.048)	0.394 (0.050)	0.284 (0.055)	0.345 (0.048)	0.329 (0.067)	0.327 (0.067)	0.332 (0.065)	0.331 (0.065)	0.332 (0.067)
	Random	0.285 (0.048)	0.394 (0.050)	0.335 (0.053)	0.352 (0.050)	0.329 (0.058)	0.323 (0.062)	0.326 (0.063)	0.327 (0.059)	0.327 (0.059)
Non-Aggregation		0.225 (0.050)	0.394 (0.050)	0.247 (0.055)	0.225 (0.040)	0.163 (0.036)	0.187 (0.046)	0.187 (0.043)	0.185 (0.043)	0.182 (0.037)

Table S147. Averaged prediction accuracy (RMSE) of RF, Elastic Net, PLS, SVM, and MIL models for 25 test sets of the APTC-1 data set property ($\Delta\Delta G^{\ddagger}$) prediction using Pmapper descriptors. The standard deviations are provided in parentheses.

Data set = APTC-1 (Test sets) Descs = Pmapper y = $\Delta\Delta G^{\ddagger}$(kcal/mol)		RF	Elastic Net	PLS	SVM	MIL				
						Non-aggregation (Instance-Wrapper)	Bag-Wrapper	Instance-Net	Bag-Net	Bag-AttentionNet
		RMSE								
Aggregation Method	Boltzmann Weight	0.348 (0.053)	0.461 (0.062)	0.358 (0.071)	0.393 (0.056)	0.381 (0.089)	0.380 (0.089)	0.380 (0.092)	0.383 (0.089)	0.379 (0.091)
	Mean	0.327 (0.061)	0.461 (0.062)	0.312 (0.051)	0.329 (0.062)	0.284 (0.065)	0.284 (0.069)	0.285 (0.067)	0.284 (0.068)	0.287 (0.071)
	Global Minimum	0.356 (0.058)	0.461 (0.062)	0.352 (0.060)	0.410 (0.057)	0.397 (0.082)	0.394 (0.081)	0.400 (0.080)	0.399 (0.080)	0.399 (0.083)
	Random	0.337 (0.057)	0.461 (0.062)	0.396 (0.053)	0.409 (0.055)	0.393 (0.066)	0.387 (0.068)	0.391 (0.070)	0.393 (0.066)	0.391 (0.066)
Non-Aggregation		0.299 (0.065)	0.461 (0.062)	0.301 (0.060)	0.274 (0.053)	0.223 (0.054)	0.255 (0.062)	0.250 (0.062)	0.249 (0.063)	0.249 (0.053)

Table S148. Averaged prediction accuracy (R^2) of RF, Elastic Net, PLS, SVM, and MIL models for 25 test sets of the APTC-1 data set property ($\Delta\Delta G^‡$) prediction using 3D-MoRSE descriptors. The standard deviations are provided in parentheses.

Data set = APTC-1 (Test sets) Descs = 3D-MoRSE y = $\Delta\Delta G^‡$(kcal/mol)		RF	Elastic Net	PLS	SVM	MIL				
						Non-aggregation (Instance-Wrapper)	Bag-Wrapper	Instance-Net	Bag-Net	Bag-AttentionNet
		R^2								
Aggregation Method	Boltzmann Weight	0.077 (0.313)	-0.092 (0.175)	0.116 (0.370)	0.213 (0.257)	0.048 (0.458)	0.037 (0.470)	0.040 (0.463)	0.031 (0.459)	0.031 (0.482)
	Mean	0.265 (0.229)	-0.092 (0.175)	0.472 (0.213)	0.355 (0.184)	0.322 (0.309)	0.320 (0.305)	0.345 (0.262)	0.349 (0.253)	0.340 (0.286)
	Global Minimum	0.086 (0.264)	-0.092 (0.175)	-0.020 (0.366)	0.149 (0.257)	-0.130 (0.420)	-0.144 (0.466)	-0.172 (0.466)	-0.142 (0.476)	-0.147 (0.440)
	Random	0.153 (0.286)	-0.092 (0.175)	-0.648 (0.661)	0.089 (0.231)	-0.394 (0.554)	-0.428 (0.546)	-0.436 (0.547)	-0.417 (0.546)	-0.330 (0.485)
Non-Aggregation		-0.365 (0.442)	-0.092 (0.175)	0.343 (0.196)	0.293 (0.193)	0.197 (0.252)	0.403 (0.252)	0.414 (0.220)	0.380 (0.377)	0.269 (0.307)

Table S149. Averaged prediction accuracy (MAE) of RF, Elastic Net, PLS, SVM, and MIL models for 25 test sets of the APTC-1 data set property ($\Delta\Delta G^{\ddagger}$) prediction using 3D-MoRSE descriptors. The standard deviations are provided in parentheses.

Data set = APTC-1 (Test sets) Descs = 3D-MoRSE y = $\Delta\Delta G^{\ddagger}$(kcal/mol)		RF	Elastic Net	PLS	SVM	MIL				
						Non-aggregation (Instance-Wrapper)	Bag-Wrapper	Instance-Net	Bag-Net	Bag-AttentionNet
		MAE								
Aggregation Method	Boltzmann Weight	0.344 (0.049)	0.394 (0.050)	0.338 (0.058)	0.319 (0.046)	0.335 (0.056)	0.333 (0.060)	0.332 (0.058)	0.336 (0.056)	0.332 (0.056)
	Mean	0.302 (0.050)	0.394 (0.050)	0.245 (0.055)	0.274 (0.050)	0.259 (0.059)	0.266 (0.064)	0.261 (0.058)	0.259 (0.059)	0.261 (0.058)
	Global Minimum	0.343 (0.049)	0.394 (0.050)	0.363 (0.054)	0.329 (0.054)	0.357 (0.079)	0.356 (0.080)	0.363 (0.083)	0.355 (0.080)	0.358 (0.080)
	Random	0.328 (0.045)	0.394 (0.050)	0.435 (0.072)	0.349 (0.054)	0.395 (0.089)	0.398 (0.081)	0.400 (0.088)	0.397 (0.087)	0.385 (0.086)
Non-Aggregation		0.379 (0.057)	0.394 (0.050)	0.292 (0.051)	0.287 (0.056)	0.293 (0.055)	0.250 (0.065)	0.252 (0.058)	0.256 (0.074)	0.287 (0.056)

Table S150. Averaged prediction accuracy (RMSE) of RF, Elastic Net, PLS, SVM, and MIL models for 25 test sets of the APTC-1 data set property ($\Delta\Delta G^{\ddagger}$) prediction using 3D-MoRSE descriptors. The standard deviations are provided in parentheses.

Data set = APTC-1 (Test sets)<br>Descs = 3D-MoRSE<br>y = $\Delta\Delta G^{\ddagger}$(kcal/mol)		RF	Elastic Net	PLS	SVM	MIL				
						Non-aggregation (Instance-Wrapper)	Bag-Wrapper	Instance-Net	Bag-Net	Bag-AttentionNet
		RMSE								
Aggregation Method	Boltzmann Weight	0.416 (0.055)	0.461 (0.062)	0.403 (0.064)	0.385 (0.052)	0.418 (0.069)	0.419 (0.071)	0.418 (0.068)	0.422 (0.068)	0.420 (0.066)
	Mean	0.374 (0.064)	0.461 (0.062)	0.314 (0.065)	0.352 (0.066)	0.352 (0.073)	0.353 (0.083)	0.348 (0.077)	0.348 (0.072)	0.347 (0.074)
	Global Minimum	0.416 (0.057)	0.461 (0.062)	0.436 (0.061)	0.403 (0.060)	0.460 (0.086)	0.460 (0.085)	0.467 (0.088)	0.459 (0.087)	0.463 (0.088)
	Random	0.398 (0.053)	0.461 (0.062)	0.551 (0.086)	0.418 (0.058)	0.508 (0.097)	0.515 (0.089)	0.516 (0.093)	0.512 (0.092)	0.498 (0.092)
Non-Aggregation		0.505 (0.072)	0.461 (0.062)	0.353 (0.060)	0.369 (0.067)	0.390 (0.070)	0.334 (0.087)	0.332 (0.082)	0.334 (0.088)	0.367 (0.066)

Table S151. Averaged prediction accuracy (R^2) of RF, Elastic Net, PLS, SVM, and MIL models for 25 test sets of the APTC-1 data set property ($\Delta\Delta G^{\ddagger}$) prediction using MBTR descriptors. The standard deviations are provided in parentheses.

Data set = APTC-1 (Test sets) Descs = MBTR y = $\Delta\Delta G^{\ddagger}$(kcal/mol)		RF	Elastic Net	PLS	SVM	MIL				
						Non-aggregation (Instance-Wrapper)	Bag-Wrapper	Instance-Net	Bag-Net	Bag-AttentionNet
		R^2								
Aggregation Method	Boltzmann Weight	0.226 (0.210)	-0.092 (0.175)	-5.450e+17 (1.571e+18)	0.067 (0.266)	-2.23e+13 (4.87e+13)	-2.366e+13 (5.597e+13)	-1.817e+13 (4.727e+13)	-1.473e+13 (3.492e+13)	-1.507e+13 (3.318e+13)
	Mean	0.287 (0.243)	-0.092 (0.175)	-1.280e+10 (3.024e+10)	0.009 (0.358)	-2.622e+10 (7.932e+10)	-2.614e+10 (6.101e+10)	-2.229e+10 (6.555e+10)	-3.364e+10 (9.700e+10)	-3.136e+10 (1.090e+11)
	Global Minimum	0.212 (0.223)	-0.092 (0.175)	-2.358e+9 (1.178e+10)	0.098 (0.309)	-1.77e+10 (8.85e+10)	-7.058e+9 (3.52e+10)	-1.027e+10 (5.127e+10)	-1.761e+10 (8.801e+10)	-2.890e+10 (1.445e+11)
	Random	0.140 (0.254)	-0.092 (0.175)	-1.444e+11 (6.705e+11)	-0.090 (0.322)	-1.893e+11 (4.490e+11)	-2.873e+11 (7.217e+11)	-1.715e+11 (3.717e+11)	-2.331e+11 (5.901e+11)	-1.760e+11 (3.631e+11)
Non-Aggregation		0.250 (0.344)	-0.092 (0.175)	-12990125.518 (33036107.604)	0.112 (0.356)	-5539164.488 (16278526.060)	-8779228.228 (18919270.690)	-21240670.538 (66081539.063)	-11874066.843 (25370865.389)	-4253196.857 (12249576.099)

Table S152. Averaged prediction accuracy (MAE) of RF, Elastic Net, PLS, SVM, and MIL models for 25 test sets of the APTC-1 data set property ($\Delta\Delta G^{\ddagger}$) prediction using MBTR descriptors. The standard deviations are provided in parentheses.

Data set = APTC-1 (Test sets) Descs = MBTR y = $\Delta\Delta G^{\ddagger}$(kcal/mol)		RF	Elastic Net	PLS	SVM	MIL				
						Non-aggregation (Instance-Wrapper)	Bag-Wrapper	Instance-Net	Bag-Net	Bag-AttentionNet
		MAE								
Aggregation Method	Boltzmann Weight	0.316 (0.043)	0.394 (0.050)	24204437.257 (66154265.051)	0.345 (0.049)	195235.462 (399293.377)	198978.272 (432479.250)	167605.550 (360550.870)	156161.898 (334604.074)	161767.844 (339280.264)
	Mean	0.303 (0.047)	0.394 (0.050)	5329.602 (11592.277)	0.339 (0.061)	7230.721 (14775.969)	8103.000 (15584.253)	6861.075 (13548.923)	8215.232 (16894.625)	7366.088 (15650.158)
	Global Minimum	0.319 (0.047)	0.394 (0.050)	1090.647 (4970.069)	0.328 (0.062)	2881.966 (13583.965)	1901.511 (8585.155)	2246.661 (10328.958)	2912.527 (13545.763)	3588.128 (17329.364)
	Random	0.334 (0.043)	0.394 (0.050)	14767.713 (45306.204)	0.367 (0.042)	24857.598 (47655.879)	29202.944 (55961.705)	24071.672 (43383.365)	27173.506 (50954.507)	24434.667 (42862.954)
Non-Aggregation		0.285 (0.059)	0.394 (0.050)	160.706 (358.362)	0.318 (0.062)	102.437 (217.992)	155.461 (295.410)	199.047 (418.240)	179.019 (338.783)	73.456 (173.338)

Table S153. Averaged prediction accuracy (RMSE) of RF, Elastic Net, PLS, SVM, and MIL models for 25 test sets of the APTC-1 data set property ($\Delta\Delta G^{\ddagger}$) prediction using MBTR descriptors. The standard deviations are provided in parentheses.

Data set = APTC-1 (Test sets) Descs = MBTR y = $\Delta\Delta G^{\ddagger}$(kcal/mol)		RF	Elastic Net	PLS	SVM	MIL				
						Non-aggregation (Instance-Wrapper)	Bag-Wrapper	Instance-Net	Bag-Net	Bag-AttentionNet
		RMSE								
Aggregation Method	Boltzmann Weight	0.383 (0.057)	0.461 (0.062)	100626818.195 (275147645.927)	0.419 (0.051)	814250.493 (1665643.349)	830792.942 (1806885.648)	699742.533 (1506971.158)	650657.376 (1391940.108)	674488.203 (1413490.526)
	Mean	0.365 (0.053)	0.461 (0.062)	22195.123 (48472.599)	0.429 (0.066)	29945.515 (62125.130)	33464.714 (65351.117)	28403.801 (56935.751)	34099.917 (71076.808)	30646.161 (65938.187)
	Global Minimum	0.387 (0.061)	0.461 (0.062)	4319.230 (19905.395)	0.410 (0.064)	11470.783 (54627.977)	7514.122 (34425.920)	8915.851 (41547.204)	11561.493 (54451.390)	14389.768 (69844.004)
	Random	0.403 (0.050)	0.461 (0.062)	60119.838 (181759.460)	0.452 (0.047)	101468.204 (192223.307)	119675.919 (226014.843)	98286.935 (174820.802)	111030.239 (204741.052)	100036.933 (175331.883)
Non-Aggregation		0.371 (0.073)	0.461 (0.062)	668.313 (1497.114)	0.403 (0.068)	425.335 (923.831)	641.013 (1238.540)	828.865 (1771.038)	739.623 (1420.993)	304.713 (722.055)

Table S154. Averaged prediction accuracy (R^2) of RF, Elastic Net, PLS, SVM, and MIL models for 25 test sets of the APTC-1 data set property ($\Delta\Delta G^{\ddagger}$) prediction using ECFP4 bit, ECFP4 count, and 2D PFP. The standard deviations are provided in parentheses.

Data set = APTC-1 (Test sets) Descs = 2D y = $\Delta\Delta G^{\ddagger}$(kcal/mol)	RF	Elastic Net	PLS	SVM	MIL				
					Non-aggregation (Instance-Wrapper)	Bag-Wrapper	Instance-Net	Bag-Net	Bag-AttentionNet
	R^2								
ECFP4 bit	0.429 (0.310)	-0.092 (0.175)	0.607 (0.186)	0.459 (0.184)	0.426 (0.294)	0.426 (0.275)	0.423 (0.291)	0.425 (0.293)	0.433 (0.279)
ECFP4 count	0.433 (0.307)	-0.092 (0.175)	0.638 (0.185)	0.478 (0.179)	0.208 (0.496)	0.202 (0.506)	0.210 (0.520)	0.213 (0.498)	0.206 (0.502)
2D PFP	0.543 (0.207)	-0.092 (0.175)	0.632 (0.166)	0.630 (0.132)	0.304 (0.261)	0.297 (0.268)	0.302 (0.261)	0.300 (0.265)	0.302 (0.265)

Table S155. Averaged prediction accuracy (MAE) of RF, Elastic Net, PLS, SVM, and MIL models for 25 test sets of the APTC-1 data set property ($\Delta\Delta G^{\ddagger}$) prediction using ECFP4 bit, ECFP4 count, and 2D PFP. The standard deviations are provided in parentheses.

Data set = APTC-1 (Test sets) Descs = 2D y = $\Delta\Delta G^{\ddagger}$(kcal/mol)	RF	Elastic Net	PLS	SVM	MIL				
					Non-aggregation (Instance-Wrapper)	Bag-Wrapper	Instance-Net	Bag-Net	Bag-AttentionNet
	MAE								
ECFP4 bit	0.244 (0.050)	0.394 (0.050)	0.215 (0.046)	0.260 (0.041)	0.239 (0.056)	0.240 (0.056)	0.241 (0.057)	0.240 (0.056)	0.239 (0.055)
ECFP4 count	0.239 (0.050)	0.394 (0.050)	0.207 (0.045)	0.249 (0.041)	0.281 (0.064)	0.283 (0.066)	0.280 (0.067)	0.279 (0.067)	0.282 (0.066)
2D PFP	0.219 (0.045)	0.394 (0.050)	0.213 (0.046)	0.210 (0.040)	0.287 (0.050)	0.289 (0.049)	0.287 (0.048)	0.288 (0.048)	0.288 (0.048)

Table S156. Averaged prediction accuracy (RMSE) of RF, Elastic Net, PLS, SVM, and MIL models for 25 test sets of the APTC-1 data set property ($\Delta\Delta G^{\ddagger}$) prediction using ECFP4 bit, ECFP4 count, and 2D PFP. The standard deviations are provided in parentheses.

Data set = APTC-1 (Test sets) Descs = 2D y = $\Delta\Delta G^{\ddagger}$(kcal/mol)	RF	Elastic Net	PLS	SVM	MIL				
					Non-aggregation (Instance-Wrapper)	Bag-Wrapper	Instance-Net	Bag-Net	Bag-AttentionNet
	RMSE								
ECFP4 bit	0.321 (0.066)	0.461 (0.062)	0.268 (0.049)	0.318 (0.053)	0.321 (0.072)	0.323 (0.074)	0.323 (0.074)	0.322 (0.074)	0.320 (0.073)
ECFP4 count	0.320 (0.069)	0.461 (0.062)	0.257 (0.051)	0.313 (0.050)	0.372 (0.072)	0.373 (0.075)	0.371 (0.077)	0.371 (0.074)	0.372 (0.075)
2D PFP	0.289 (0.058)	0.461 (0.062)	0.259 (0.050)	0.264 (0.052)	0.359 (0.050)	0.361 (0.051)	0.360 (0.050)	0.360 (0.051)	0.360 (0.050)

Table S157. Averaged prediction accuracy (R^2, MAE, RMSE) of MolCLR, GEM, and Uni-Mol models for 25 test sets of the APTC-1 data set property ($\Delta\Delta G^{\ddagger}$) prediction. The standard deviations are provided in parentheses.

Data set = APTC-1 (Test sets) y = $\Delta\Delta G^{\ddagger}$(kcal/mol)		R^2	MAE	RMSE
GEM	Global min.	0.124 (0.286)	0.326 (0.057)	0.406 (0.057)
	Non-aggregation	0.540 (0.268)	0.206 (0.049)	0.286 (0.068)
Uni-Mol	Global min.	0.243 (0.211)	0.311 (0.065)	0.382 (0.076)
	Non-aggregation	0.760 (0.144)	0.152 (0.040)	0.205 (0.056)
MolCLR		0.278 (0.262)	0.298 (0.061)	0.367 (0.075)

Table S158. Averaged prediction accuracy (R^2) of RF, Elastic Net, PLS, SVM, and MIL models for 40 test points of the APTC-2 data set property ($\Delta\Delta G^{\ddagger}$) prediction using MOE descriptors.

Data set = APTC-2 (Test points) Descs = MOE y = $\Delta\Delta G^{\ddagger}$(kcal/mol)		RF	Elastic Net	PLS	SVM	MIL				
						Non-aggregation (Instance-Wrapper)	Bag-Wrapper	Instance-Net	Bag-Net	Bag-AttentionNet
		R^2								
Aggregation Method	Boltzmann Weight	0.430	-0.052	-0.138	0.277	-0.381	-0.411	-0.349	-0.445	-0.494
	Mean	0.314	-0.052	0.177	0.498	0.065	0.075	0.127	0.100	0.039
	Global Minimum	0.389	-0.052	-0.451	0.285	-0.434	-0.494	-0.332	-0.420	-0.434
	Random	0.292	-0.052	-0.279	0.138	-1.163	-0.269	-0.391	-0.357	-0.429
Non-Aggregation		0.417	-0.051	0.415	0.656	0.763	0.433	0.332	0.327	0.178

Table S159. Averaged prediction accuracy (MAE) of RF, Elastic Net, PLS, SVM, and MIL models for 40 test points of the APTC-2 data set property ($\Delta\Delta G^{\ddagger}$) prediction using MOE descriptors.

Data set = APTC-2 (Test points)<br>Descs = MOE<br>y = $\Delta\Delta G^{\ddagger}$(kcal/mol)		RF	Elastic Net	PLS	SVM	MIL				
						Non-aggregation (Instance-Wrapper)	Bag-Wrapper	Instance-Net	Bag-Net	Bag-AttentionNet
		MAE								
Aggregation Method	Boltzmann Weight	0.210	0.316	0.348	0.230	0.312	0.319	0.314	0.311	0.328
	Mean	0.228	0.316	0.259	0.188	0.215	0.205	0.202	0.199	0.214
	Global Minimum	0.213	0.316	0.351	0.228	0.323	0.327	0.294	0.320	0.331
	Random	0.239	0.316	0.340	0.239	0.345	0.283	0.296	0.288	0.306
Non-Aggregation		0.199	0.316	0.206	0.174	0.147	0.210	0.220	0.219	0.232

Table S160. Averaged prediction accuracy (RMSE) of RF, Elastic Net, PLS, SVM, and MIL models for 40 test points of the APTC-2 data set property ($\Delta\Delta G^{\ddagger}$) prediction using MOE descriptors.

Data set = APTC-2 (Test points)<br>Descs = MOE<br>y = $\Delta\Delta G^{\ddagger}$(kcal/mol)		RF	Elastic Net	PLS	SVM	MIL				
						Non-aggregation (Instance-Wrapper)	Bag-Wrapper	Instance-Net	Bag-Net	Bag-AttentionNet
		RMSE								
Aggregation Method	Boltzmann Weight	0.210	0.316	0.348	0.230	0.312	0.319	0.314	0.311	0.328
	Mean	0.228	0.316	0.259	0.188	0.215	0.205	0.202	0.199	0.214
	Global Minimum	0.213	0.316	0.351	0.228	0.323	0.327	0.294	0.320	0.331
	Random	0.239	0.316	0.340	0.239	0.345	0.283	0.296	0.288	0.306
Non-Aggregation		0.199	0.314	0.206	0.174	0.147	0.210	0.220	0.219	0.232

Table S161. Averaged prediction accuracy (R^2) of RF, Elastic Net, PLS, SVM, and MIL models for 40 test points of the APTC-2 data set property ($\Delta\Delta G^{\ddagger}$) prediction using Pmapper descriptors.

Data set = APTC-2 (Test points) Descs = Pmapper y = $\Delta\Delta G^{\ddagger}$(kcal/mol)		RF	Elastic Net	PLS	SVM	MIL				
						Non-aggregation (Instance-Wrapper)	Bag-Wrapper	Instance-Net	Bag-Net	Bag-AttentionNet
		R^2								
Aggregation Method	Boltzmann Weight	0.364	-0.052	0.469	0.253	0.364	0.356	0.260	0.409	0.357
	Mean	0.612	-0.052	0.650	0.605	0.249	0.216	0.187	0.218	0.168
	Global Minimum	0.319	-0.052	0.437	0.273	0.257	0.334	0.282	0.348	0.249
	Random	0.377	-0.052	0.380	0.265	0.354	0.310	0.354	0.241	0.322
Non-Aggregation		0.721	-0.051	0.615	0.661	0.765	0.591	0.596	0.610	0.576

Table S162. Averaged prediction accuracy (MAE) of RF, Elastic Net, PLS, SVM, and MIL models for 40 test points of the APTC-2 data set property ($\Delta\Delta G^{‡}$) prediction using Pmapper descriptors.

Data set = APTC-2 (Test points) Descs = Pmapper y = $\Delta\Delta G^{‡}$(kcal/mol)		RF	Elastic Net	PLS	SVM	MIL				
						Non-aggregation (Instance-Wrapper)	Bag-Wrapper	Instance-Net	Bag-Net	Bag-AttentionNet
		MAE								
Aggregation Method	Boltzmann Weight	0.197	0.316	0.199	0.229	0.180	0.184	0.197	0.178	0.183
	Mean	0.163	0.316	0.189	0.167	0.213	0.213	0.214	0.212	0.214
	Global Minimum	0.215	0.316	0.205	0.222	0.222	0.214	0.223	0.209	0.218
	Random	0.210	0.316	0.219	0.227	0.206	0.213	0.212	0.217	0.207
Non-Aggregation		0.142	0.316	0.188	0.158	0.142	0.192	0.187	0.179	0.179

Table S163. Averaged prediction accuracy (RMSE) of RF, Elastic Net, PLS, SVM, and MIL models for 40 test points of the APTC-2 data set property ($\Delta\Delta G^{\ddagger}$) prediction using Pmapper descriptors.

Data set = APTC-2 (Test points) Descs = Pmapper y = $\Delta\Delta G^{\ddagger}$(kcal/mol)		RF	Elastic Net	PLS	SVM	MIL				
						Non-aggregation (Instance-Wrapper)	Bag-Wrapper	Instance-Net	Bag-Net	Bag-AttentionNet
		RMSE								
Aggregation Method	Boltzmann Weight	0.197	0.316	0.199	0.229	0.180	0.184	0.197	0.178	0.183
	Mean	0.163	0.316	0.189	0.167	0.213	0.213	0.214	0.212	0.214
	Global Minimum	0.215	0.316	0.205	0.222	0.222	0.214	0.223	0.209	0.218
	Random	0.210	0.316	0.219	0.227	0.206	0.213	0.212	0.217	0.207
Non-Aggregation		0.142	0.314	0.188	0.158	0.142	0.192	0.187	0.179	0.179

Table S164. Averaged prediction accuracy (R^2) of RF, Elastic Net, PLS, SVM, and MIL models for 40 test points of the APTC-2 data set property ($\Delta\Delta G^{\ddagger}$) prediction using 3D-MoRSE descriptors.

Data set = APTC-2 (Test points)<br>Descs = 3D-MoRSE<br>y = $\Delta\Delta G^{\ddagger}$(kcal/mol)		RF	Elastic Net	PLS	SVM	MIL				
						Non-aggregation (Instance-Wrapper)	Bag-Wrapper	Instance-Net	Bag-Net	Bag-AttentionNet
		R^2								
Aggregation Method	Boltzmann Weight	0.305	-0.052	-0.204	0.283	0.330	0.326	0.239	0.282	0.310
	Mean	0.476	-0.052	0.262	0.447	0.669	0.685	0.667	0.662	0.698
	Global Minimum	0.234	-0.052	-0.297	0.271	0.337	0.289	0.292	0.324	0.324
	Random	0.370	-0.052	0.258	0.339	0.476	0.439	0.465	0.487	0.423
Non-Aggregation		-0.310	-0.051	0.311	0.563	0.765	0.701	0.63	0.649	0.455

Table S165. Averaged prediction accuracy (MAE) of RF, Elastic Net, PLS, SVM, and MIL models for 40 test points of the APTC-2 data set property ($\Delta\Delta G^{\ddagger}$) prediction using 3D-MoRSE descriptors.

Data set = APTC-2 (Test points) Descs = 3D-MoRSE y = $\Delta\Delta G^{\ddagger}$(kcal/mol)		RF	Elastic Net	PLS	SVM	MIL				
						Non-aggregation (Instance-Wrapper)	Bag-Wrapper	Instance-Net	Bag-Net	Bag-AttentionNet
		MAE								
Aggregation Method	Boltzmann Weight	0.232	0.316	0.361	0.223	0.216	0.214	0.225	0.226	0.222
	Mean	0.196	0.316	0.241	0.185	0.147	0.150	0.152	0.154	0.147
	Global Minimum	0.249	0.316	0.371	0.226	0.245	0.245	0.243	0.234	0.243
	Random	0.214	0.316	0.274	0.207	0.214	0.221	0.215	0.209	0.220
Non-Aggregation		0.295	0.316	0.215	0.176	0.132	0.147	0.165	0.167	0.192

Table S166. Averaged prediction accuracy (RMSE) of RF, Elastic Net, PLS, SVM, and MIL models for 40 test points of the APTC-2 data set property ($\Delta\Delta G^{\ddagger}$) prediction using 3D-MoRSE descriptors.

Data set = APTC-2 (Test points)<br>Descs = 3D-MoRSE<br>y = $\Delta\Delta G^{\ddagger}$(kcal/mol)		RF	Elastic Net	PLS	SVM	MIL				
						Non-aggregation (Instance-Wrapper)	Bag-Wrapper	Instance-Net	Bag-Net	Bag-AttentionNet
		RMSE								
Aggregation Method	Boltzmann Weight	0.232	0.316	0.361	0.223	0.216	0.214	0.225	0.226	0.222
	Mean	0.196	0.316	0.241	0.185	0.147	0.150	0.152	0.154	0.147
	Global Minimum	0.249	0.316	0.371	0.226	0.245	0.245	0.243	0.234	0.243
	Random	0.214	0.316	0.274	0.207	0.214	0.221	0.215	0.209	0.220
Non-Aggregation		0.295	0.314	0.215	0.176	0.132	0.147	0.165	0.167	0.192

Table S167. Averaged prediction accuracy (R^2) of RF, Elastic Net, PLS, SVM, and MIL models for 40 test points of the APTC-2 data set property ($\Delta\Delta G^{\ddagger}$) prediction using MBTR descriptors.

Data set = APTC-2 (Test points)<br>Descs = MBTR<br>y = $\Delta\Delta G^{\ddagger}$(kcal/mol)		RF	Elastic Net	PLS	SVM	MIL				
						Non-aggregation (Instance-Wrapper)	Bag-Wrapper	Instance-Net	Bag-Net	Bag-AttentionNet
		R^2								
Aggregation Method	Boltzmann Weight	0.502	-0.052	-1.41e+31	0.023	-8.50e+10	-7.51e+10	-9.99e+9	-8.73e+9	-1.56e+10
	Mean	0.529	-0.052	-4.67e+10	0.488	-1.72e+9	-9.22e+9	-5.19e+9	-6.69e+9	-6.21e+8
	Global Minimum	0.495	-0.052	-566858.929	0.021	-738255.650	-246167.038	-267511.605	-98695.977	-260023.220
	Random	0.544	-0.052	-112.596	0.063	-310.953	-296.204	-624.981	-455.618	-149.490
Non-Aggregation		0.533	-0.051	-49687308.215	0.694	-1679342.168	-49768.159	-2.91e+6	-530898.819	-54.718

Table S168. Averaged prediction accuracy (MAE) of RF, Elastic Net, PLS, SVM, and MIL models for 40 test points of the APTC-2 data set property ($\Delta\Delta G^{\ddagger}$) prediction using MBTR descriptors.

Data set = APTC-2 (Test points)<br>Descs = MBTR<br>y = $\Delta\Delta G^{\ddagger}$(kcal/mol)		RF	Elastic Net	PLS	SVM	MIL				
						Non-aggregation (Instance-Wrapper)	Bag-Wrapper	Instance-Net	Bag-Net	Bag-AttentionNet
		MAE								
Aggregation Method	Boltzmann Weight	0.196	0.316	2.52e+14	0.260	24008.704	25540.935	9375.611	8913.600	11181.217
	Mean	0.197	0.316	14526.792	0.207	2787.697	6458.445	4845.627	5501.794	1675.985
	Global Minimum	0.194	0.316	60.258	0.263	84.711	44.656	51.128	33.928	51.229
	Random	0.182	0.316	1.233	0.259	1.408	1.642	2.283	1.820	1.058
Non-Aggregation		0.169	0.316	474.277	0.167	87.308	15.159	114.968	49.159	0.659

Table S169. Averaged prediction accuracy (RMSE) of RF, Elastic Net, PLS, SVM, and MIL models for 40 test points of the APTC-2 data set property ($\Delta\Delta G^{\ddagger}$) prediction using MBTR descriptors.

Data set = APTC-2 (Test points)<br>Descs = MBTR<br>y = $\Delta\Delta G^{\ddagger}$(kcal/mol)		RF	Elastic Net	PLS	SVM	MIL				
						Non-aggregation (Instance-Wrapper)	Bag-Wrapper	Instance-Net	Bag-Net	Bag-AttentionNet
		RMSE								
Aggregation Method	Boltzmann Weight	0.196	0.316	2.523e+14	0.260	24008.704	25540.935	9375.611	8913.600	11181.217
	Mean	0.197	0.316	14526.792	0.207	2787.697	6458.445	4845.627	5501.794	1675.985
	Global Minimum	0.194	0.316	60.258	0.263	84.711	44.656	51.128	33.928	51.229
	Random	0.182	0.316	1.233	0.259	1.408	1.642	2.283	1.820	1.058
Non-Aggregation		0.169	0.314	474.277	0.167	87.308	15.159	114.968	49.159	0.659

Table S170. Averaged prediction accuracy (R^2) of RF, Elastic Net, PLS, SVM, and MIL models for 40 test points of the APTC-2 data set property ($\Delta\Delta G^{\ddagger}$) prediction using ECFP4 bit, ECFP4 count, and 2D PFP.

Data set = APTC-2 (Test points) Descs = 2D y = $\Delta\Delta G^{\ddagger}$(kcal/mol)	RF	Elastic Net	PLS	SVM	MIL				
					Non-aggregation (Instance-Wrapper)	Bag-Wrapper	Instance-Net	Bag-Net	Bag-AttentionNet
	R^2								
ECFP4 bit	0.556	-0.052	0.157	0.507	0.383	0.334	0.348	0.351	0.405
ECFP4 count	0.473	-0.052	0.206	0.459	0.309	0.293	0.262	0.300	0.271
2D PFP	0.754	-0.052	0.492	0.577	0.319	0.291	0.319	0.317	0.325

Table S171. Averaged prediction accuracy (MAE) of RF, Elastic Net, PLS, SVM, and MIL models for 40 test points of the APTC-2 data set property ($\Delta\Delta G^{\ddagger}$) prediction using ECFP4 bit, ECFP4 count, and 2D PFP.

Data set = APTC-2 (Test points) Descs = 2D y = $\Delta\Delta G^{\ddagger}$(kcal/mol)	RF	Elastic Net	PLS	SVM	MIL				
					Non-aggregation (Instance-Wrapper)	Bag-Wrapper	Instance-Net	Bag-Net	Bag-AttentionNet
	MAE								
ECFP4 bit	0.171	0.316	0.259	0.186	0.181	0.183	0.182	0.189	0.178
ECFP4 count	0.198	0.316	0.256	0.196	0.193	0.201	0.200	0.206	0.202
2D PFP	0.134	0.316	0.213	0.182	0.184	0.189	0.179	0.187	0.181

Table S172. Averaged prediction accuracy (RMSE) of RF, Elastic Net, PLS, SVM, and MIL models for 40 test points of the APTC-2 data set property ($\Delta\Delta G^{\ddagger}$) prediction using ECFP4 bit, ECFP4 count, and 2D PFP.

Data set = APTC-2 (Test points) Descs = 2D y = $\Delta\Delta G^{\ddagger}$(kcal/mol)	RF	Elastic Net	PLS	SVM	MIL				
					Non-aggregation (Instance-Wrapper)	Bag-Wrapper	Instance-Net	Bag-Net	Bag-AttentionNet
	RMSE								
ECFP4 bit	0.171	0.316	0.259	0.186	0.181	0.183	0.182	0.189	0.178
ECFP4 count	0.198	0.316	0.256	0.196	0.193	0.201	0.200	0.206	0.202
2D PFP	0.134	0.316	0.213	0.182	0.184	0.189	0.179	0.187	0.181

Table S173. Averaged prediction accuracy (R^2, MAE, RMSE) of MolCLR, GEM, and Uni-Mol models for 40 test points of the APTC-2 data set property ($\Delta\Delta G^{\ddagger}$) prediction.

Data set = APTC-2 (Test points)<br>y = $\Delta\Delta G^{\ddagger}$(kcal/mol)		R^2	MAE	RMSE
GEM	Global min.	0.202	0.373	0.373
	Non-aggregation	0.362	0.309	0.309
Uni-Mol	Global min.	0.237	0.226	0.226
	Non-aggregation	0.625	0.152	0.152
MolCLR		-0.027	0.285	0.285

Table S174. Average prediction accuracy (MAE) of random forest (RF) models for 25 test sets of the MP data sets property (melting point) prediction. The standard deviations are provided in parentheses. All methods used MOE 117 descriptors, Pmapper 575 descriptors, 3D-MoRSE 160 descriptors, or MBTR 950 descriptors. The best accuracy value per descriptor is highlighted in **bold,** and the second-best is underlined. * represents statistically higher than the next best at a significance level of 5%.

		MOE	Pmapper	3D-MoRSE	MBTR
Aggregation Method	Boltzmann weight	**20.695 (2.357)**	34.881 (3.715)	28.253* (3.792)	27.045 (3.482)
	Mean	20.836 (2.683)	**32.959* (3.822)**	**26.955* (3.640)**	25.527* (3.671)
	Global Minimum	20.753 (2.268)	35.075 (4.334)	29.438 (3.732)	28.432 (3.484)
	Random	21.843 (2.769)	35.093 (4.089)	30.808 (3.932)	28.423 (3.721)
Non-aggregation		21.144 (2.885)	35.148 (3.413)	31.953 (3.675)	**23.389* (3.877)**

Table S175. Comparison of average prediction accuracy (MAE) for 25 test sets of the MP data sets property (melting point) prediction among ML models. The standard deviations are provided in parentheses. Random Forest (RF) model using MOE descriptors and ECFP4 count, Muti-layer perceptron (MLP) using MOE, Pmapper, 3D-MoRSE descriptors and ECFP4 count. GEM and Uni-Mol models were built using the global minimum conformers or with non-aggregation approaches. The MolCLR is also reported as a comparison. The best accuracy value per target is highlighted in **bold,** and the second-best is underlined. * represents statistically higher than the next best at a significance level of 5%.

MLP	MOE mean	24.402 (3.492)
	MOE non-aggregation	19.931 (3.675)
	Pmapper mean	42.537 (7.804)
	Pmapper non-aggregation	31.753 (4.936)
	3D-MoRSE mean	22.068 (3.409)
	3D-MoRSE non-aggregation	21.356 (3.484)
	ECFP4 count	28.609 (5.226)
RF	MOE Boltzmann weight	20.695 (2.357)
	MOE global minimum	20.753 (2.268)
	ECFP4 count	23.838 (3.332)
GEM	Global min.	19.414 (3.466)
	Non-aggregation	17.331 (3.529)*
Uni-Mol	Global min.	19.623 (2.396)
	Non-aggregation	**16.786 (2.477)**
MolCLR		23.942 (4.032)

Table S176. Averaged prediction accuracy (MAE) of random forest (RF) models for 25 test sets for the APTC-1 & 40 test points for the APTC-2 data sets property ($\Delta\Delta G^{\ddagger}$) prediction. For APTC-1, the standard deviations are provided in parentheses. All methods used MOE 117 descriptors, Pmapper 1202/1556 (APTC-1/APTC-2) descriptors, 3D-MoRSE 160 descriptors, or MBTR 2310 descriptors. The best accuracy value per descriptor is highlighted in **bold**, and the second-best is underlined. * represents statistically higher than the next best at a significance level of 5%.

		APTC-1				APTC-2			
		MOE	Pmapper	3D-MoRSE	MBTR	MOE	Pmapper	3D-MoRSE	MBTR
Aggregation Method	Boltzmann weight	0.294 (0.059)	0.288 (0.046)	0.344 (0.049)	0.316 (0.043)	<u>0.210</u>	0.197	0.232	0.196
	Mean	<u>0.283 (0.054)</u>	<u>0.267 (0.052)</u>	**0.302* (0.050)**	<u>0.303 (0.047)</u>	0.228	<u>0.163</u>	**0.196**	0.197
	Global Minimum	0.304 (0.053)	0.291 (0.048)	0.343 (0.049)	0.319 (0.047)	0.213	0.215	0.249	0.194
	Random	**0.276 (0.045)**	0.285 (0.048)	<u>0.328* (0.045)</u>	0.334 (0.043)	0.239	0.210	<u>0.214</u>	<u>0.182</u>
Non-aggregation		0.306 (0.064)	**0.225* (0.050)**	0.379 (0.057)	**0.285* (0.059)**	**0.199**	**0.142**	0.295	**0.169**

Table S177. Comparison of averaged prediction accuracy (MAE) for 25 test sets for the APTC-1 & 40 test points for the APTC-2 data sets property ($\Delta\Delta G^{\ddagger}$) prediction among ML models. Random Forest (RF) model using MOE descriptors, Muti-layer perceptron (MLP) using MOE, Pmapper, 3D-MoRSE, MBTR descriptors. Both models also used 3 different 2D descriptors: ECFP4 as bit vectors, ECFP4 as count-up vectors, and 2D PEP. GEM and Uni-Mol models were built using the global minimum conformers or with non-aggregation approaches. The MolCLR is also reported as a comparison. The best accuracy value per target is highlighted in bold, and the second-best is underlined. * represents statistically higher than the next best at the significance level of 5%.

		APTC -1	APTC-2
MLP	MOE mean	0.307 (0.062)	0.215
	MOE non-aggregation	0.279 (0.065)	0.147
	Pmapper mean	0.216 (0.051)	0.213
	Pmapper non-aggregation	<u>0.163 (0.036)*</u>	0.142
	3D-MoRSE mean	0.259 (0.059)	0.147
	3D-MoRSE non-aggregation	0.293 (0.055)	**0.132**
	ECFP4 bit	0.239 (0.056)	0.181
	ECFP4 count	0.281 (0.064)	0.193
	2D PFP	0.287 (0.050)	0.184
RF	Pmapper mean	0.267 (0.052)	0.163
	Pmapper non-aggregation	0.225 (0.050)	0.142
	ECFP4 bit	0.244 (0.050)	0.171
	ECFP4 count	0.239 (0.050)	0.198
	2D PFP	0.219 (0.045)	<u>0.134</u>
GEM	Global min.	0.326 (0.057)	0.373
	Non-aggregation	0.206 (0.049)	0.309
Uni-Mol	Global min.	0.311 (0.065)	0.226
	Non-aggregation	**0.152 (0.040)**	0.152
MolCLR		0.298 (0.061)	0.285

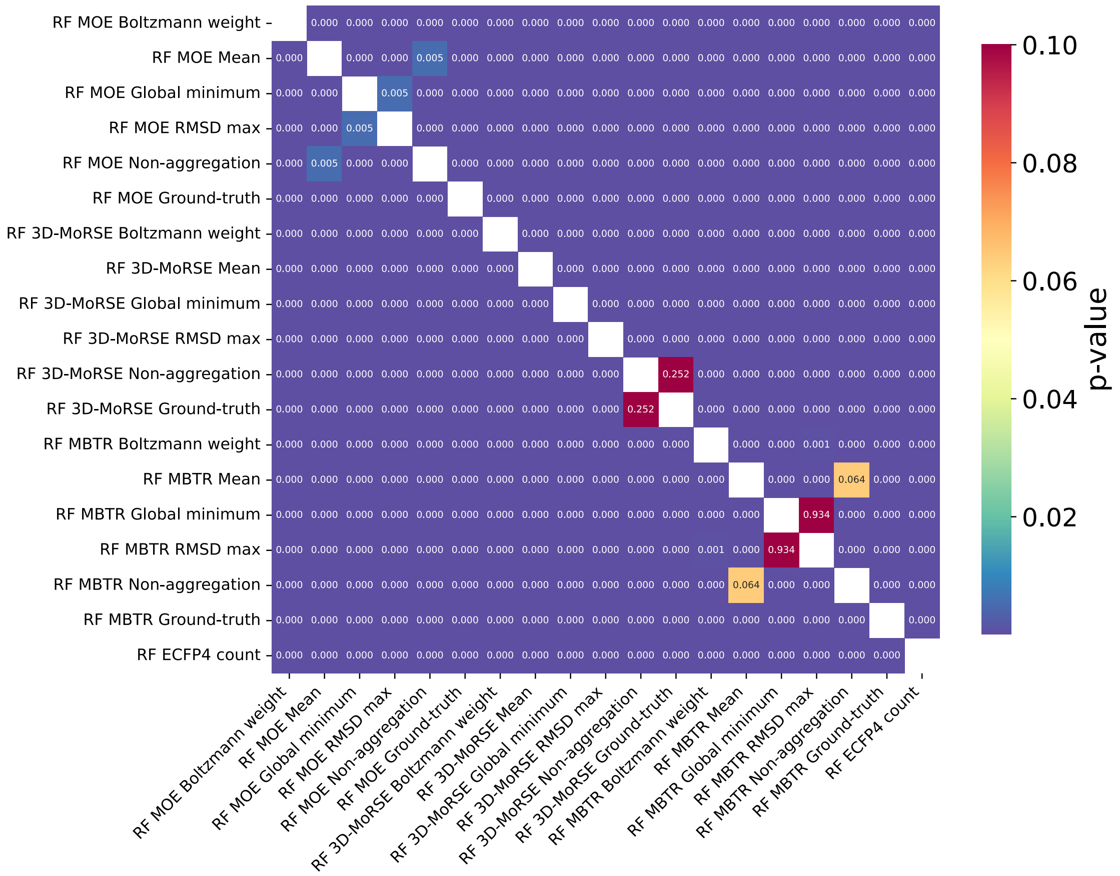

Figure S8. Heatmap of p-values corresponding to Table 1, showing the significance of dipole moment prediction accuracy (R^2) for 15 test sets of the PQC data set.

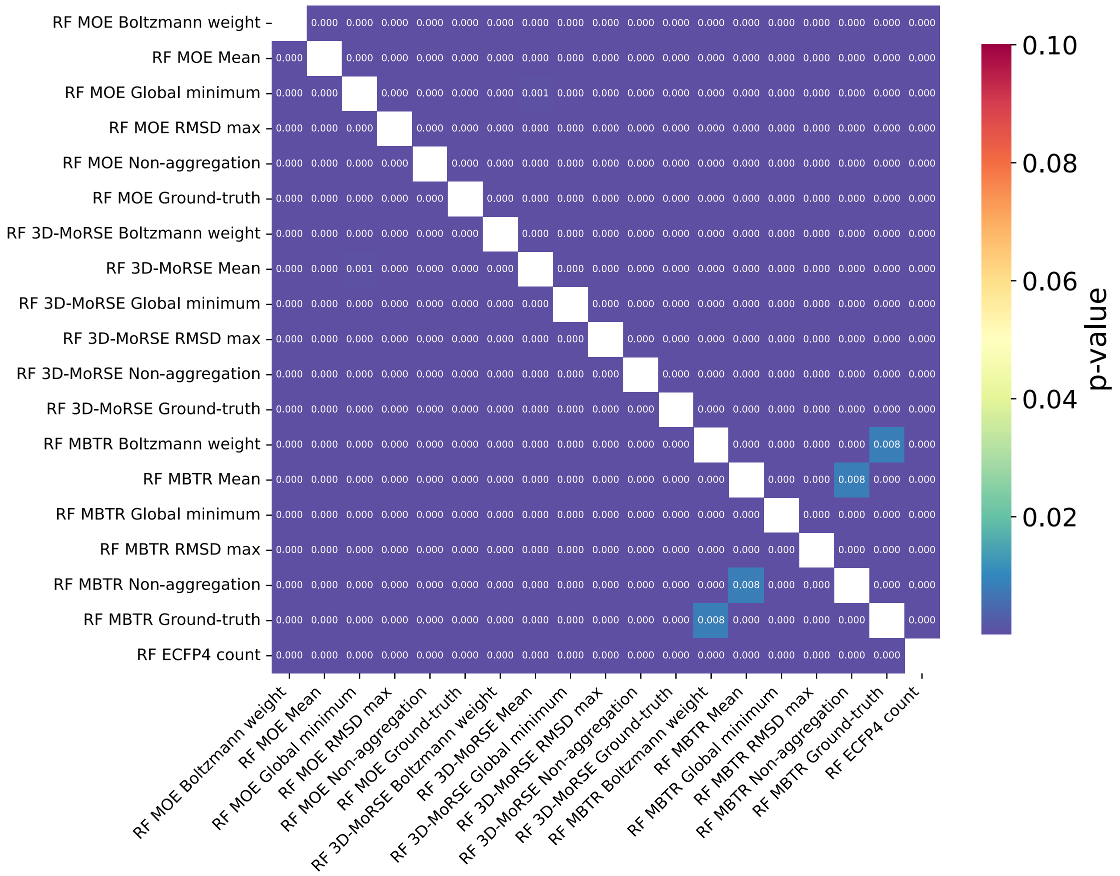

Figure S9. Heatmap of p-values corresponding to Table 1, showing the significance of HOMO prediction accuracy (R^2) for 15 test sets of the PQC data set.

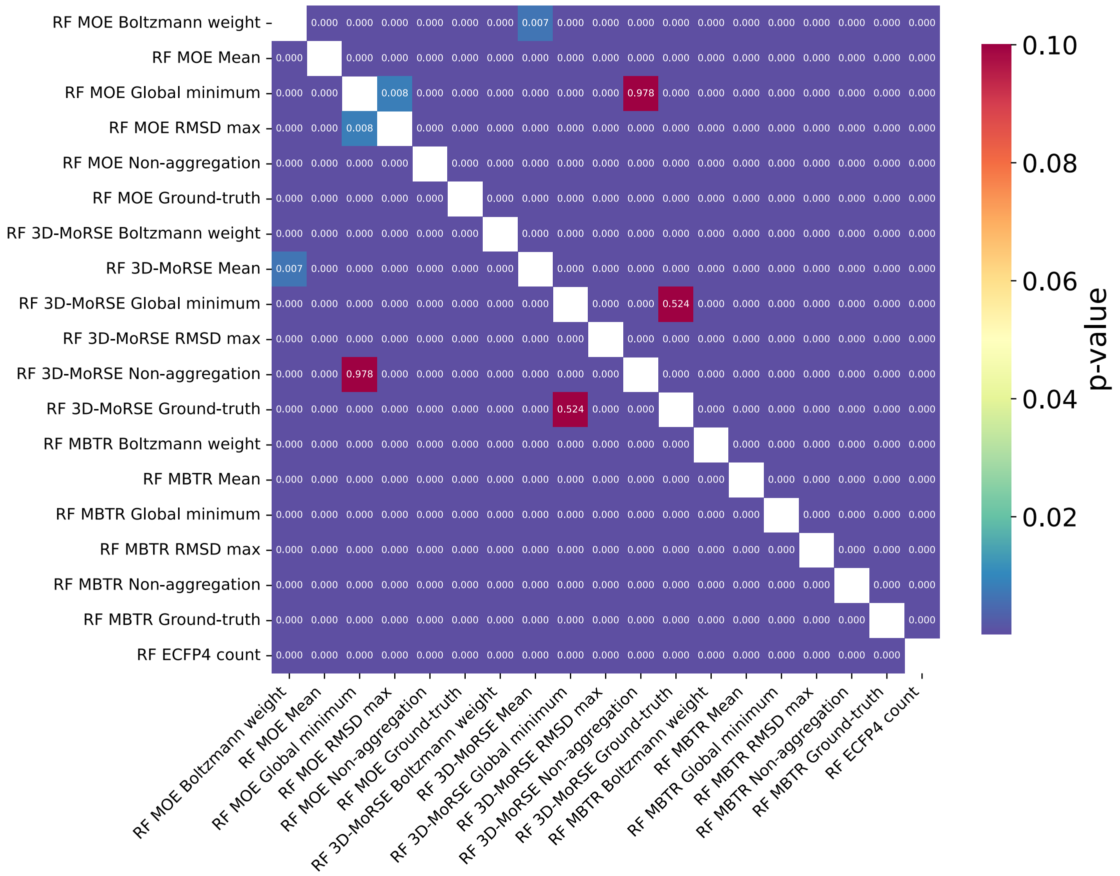

Figure S10. Heatmap of p-values corresponding to Table 1, showing the significance of HOMO-LUMO gap prediction accuracy (R^2) for 15 test sets of the PQC data set.

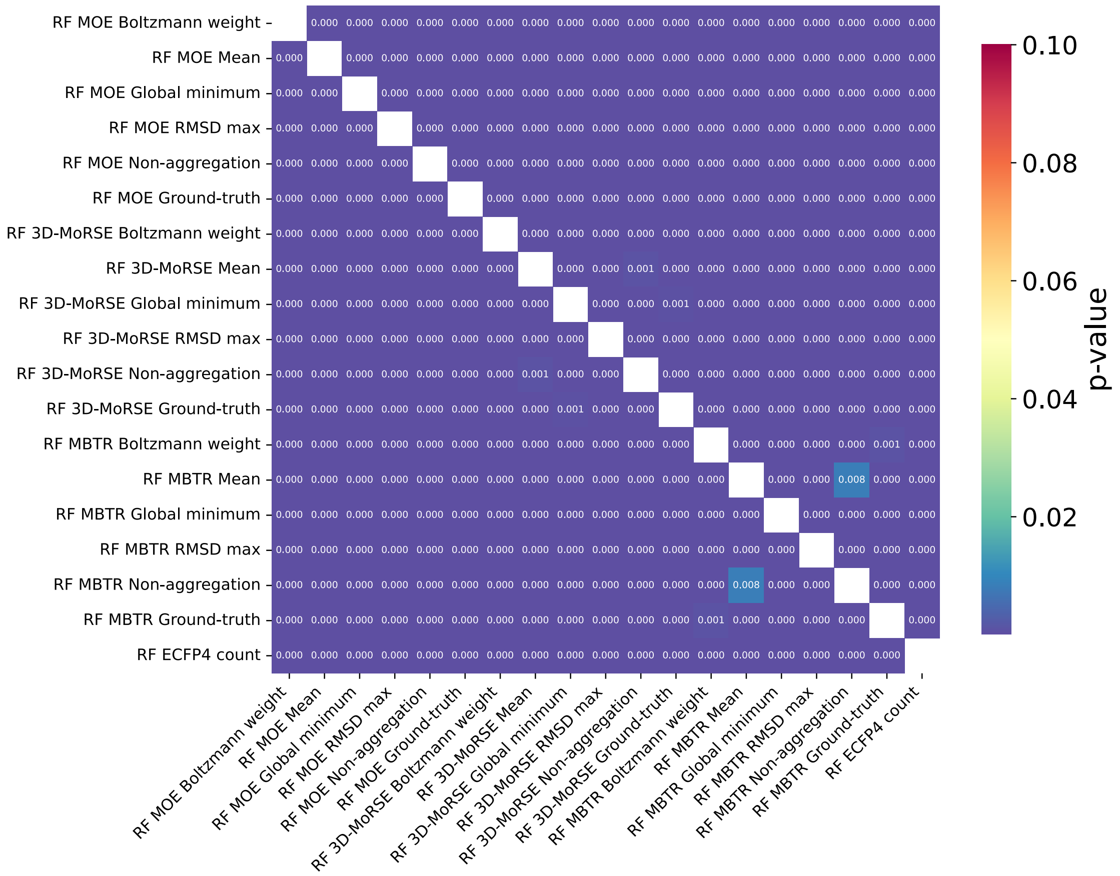

Figure S11. Heatmap of p-values corresponding to Table 1, showing the significance of LUMO prediction accuracy (R^2) for 15 test sets of the PQC data set.

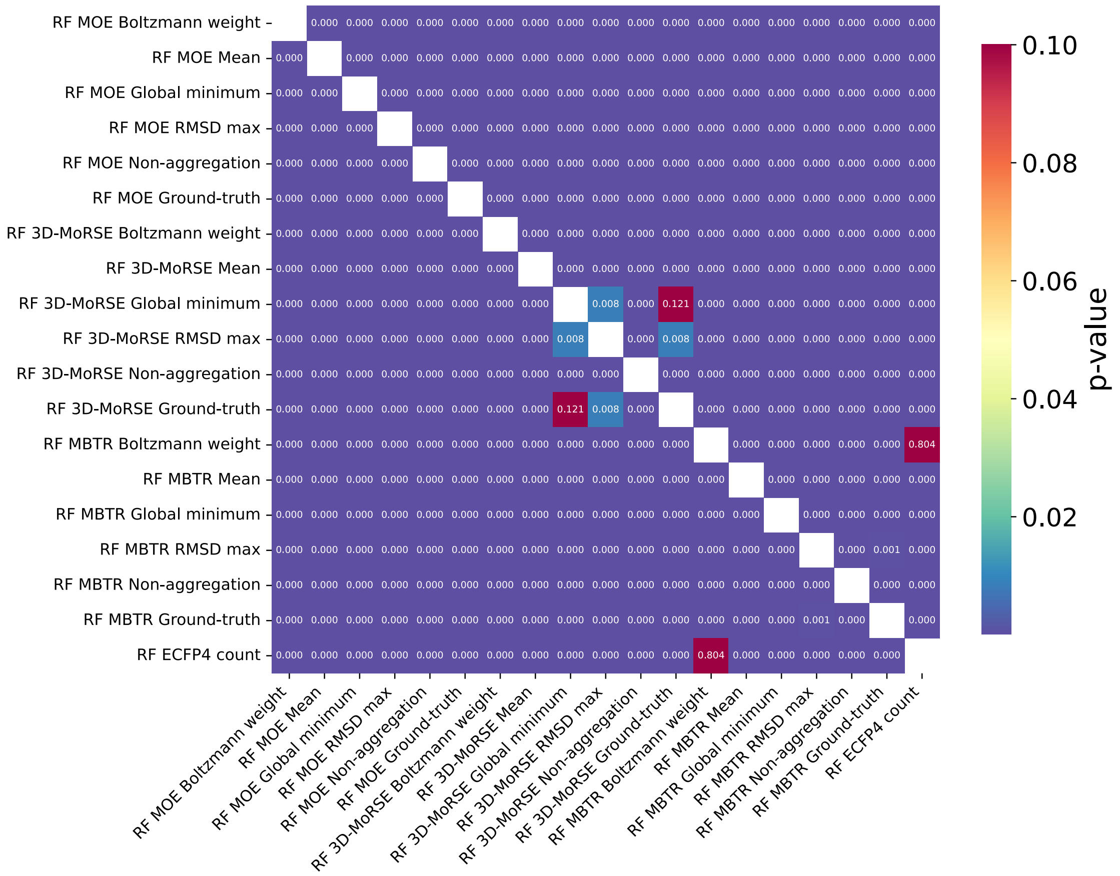

Figure S12. Heatmap of p-values corresponding to Table 1, showing the significance of energy prediction accuracy (R^2) for 15 test sets of the PQC data set.

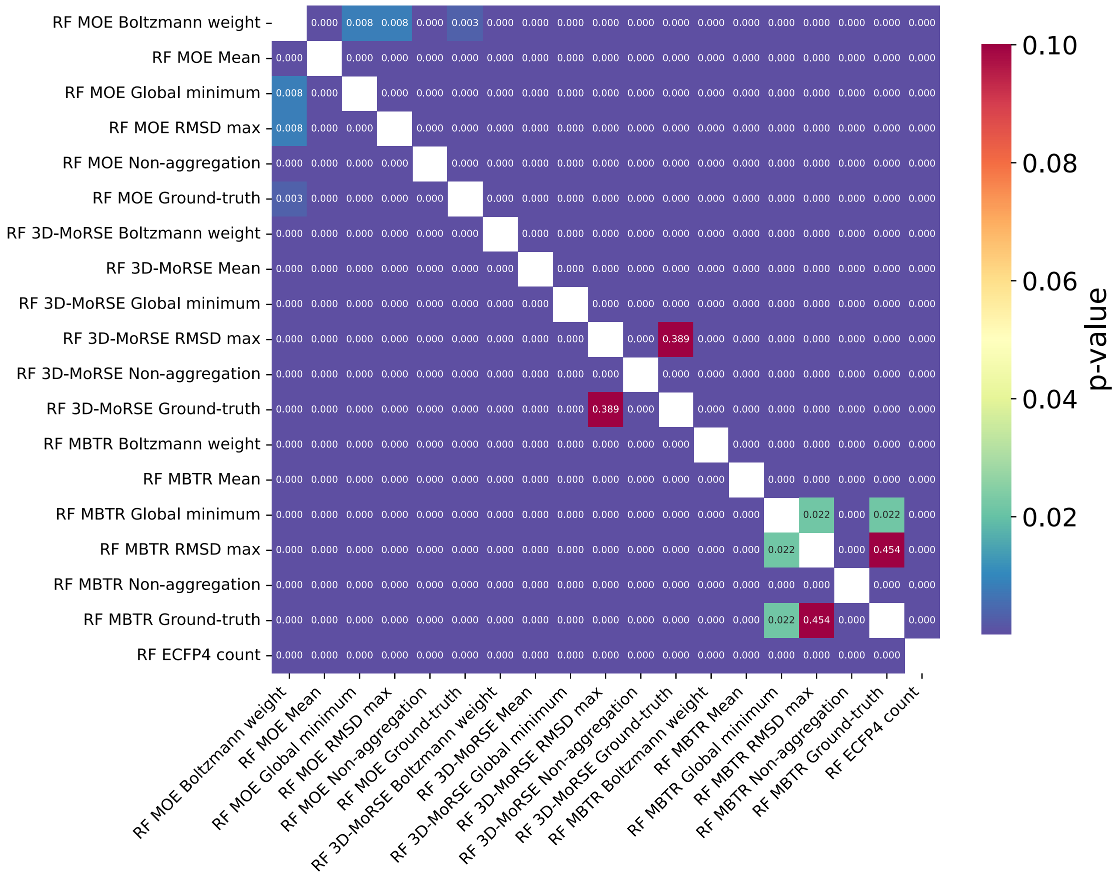

Figure S13. Heatmap of p-values corresponding to Table 1, showing the significance of enthalpy prediction accuracy (R^2) for 15 test sets of the PQC data set.

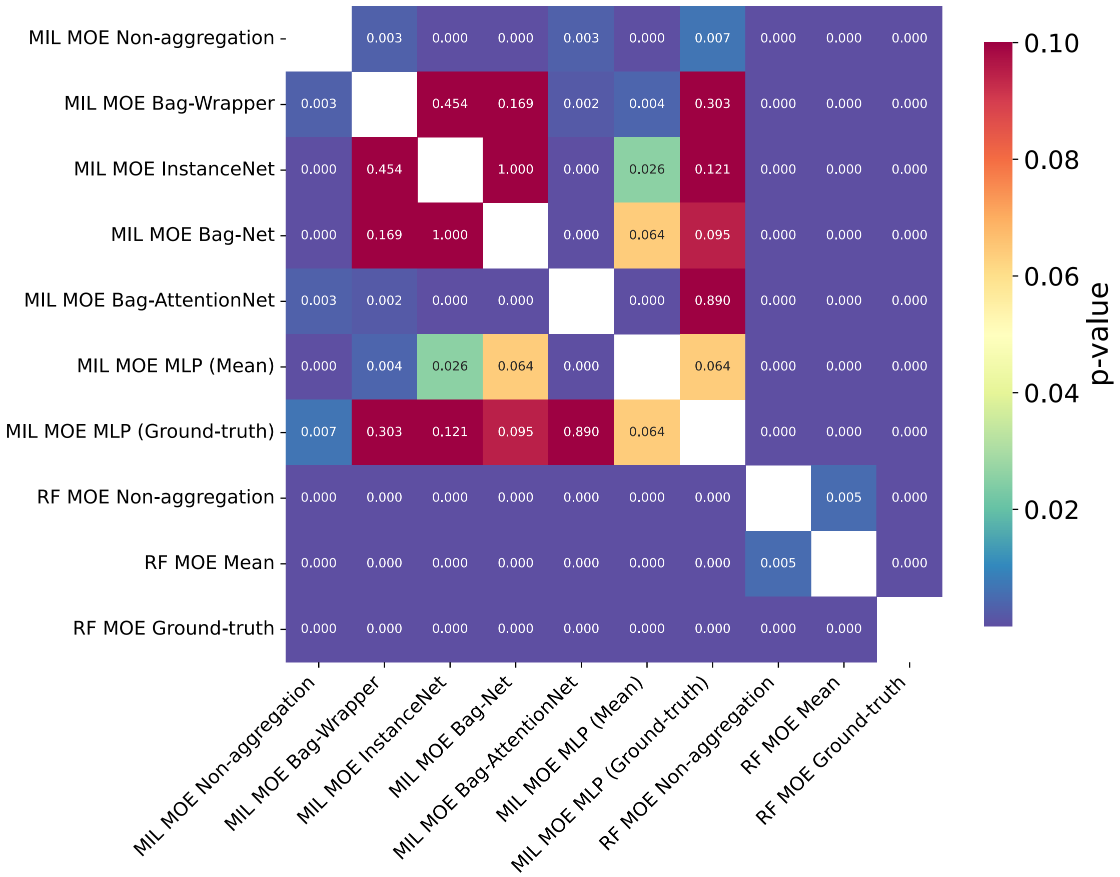

Figure S14. Heatmap of p-values corresponding to Table 2, showing the significance of dipole moment prediction accuracy (R^2) for 15 test sets of the PQC data set.

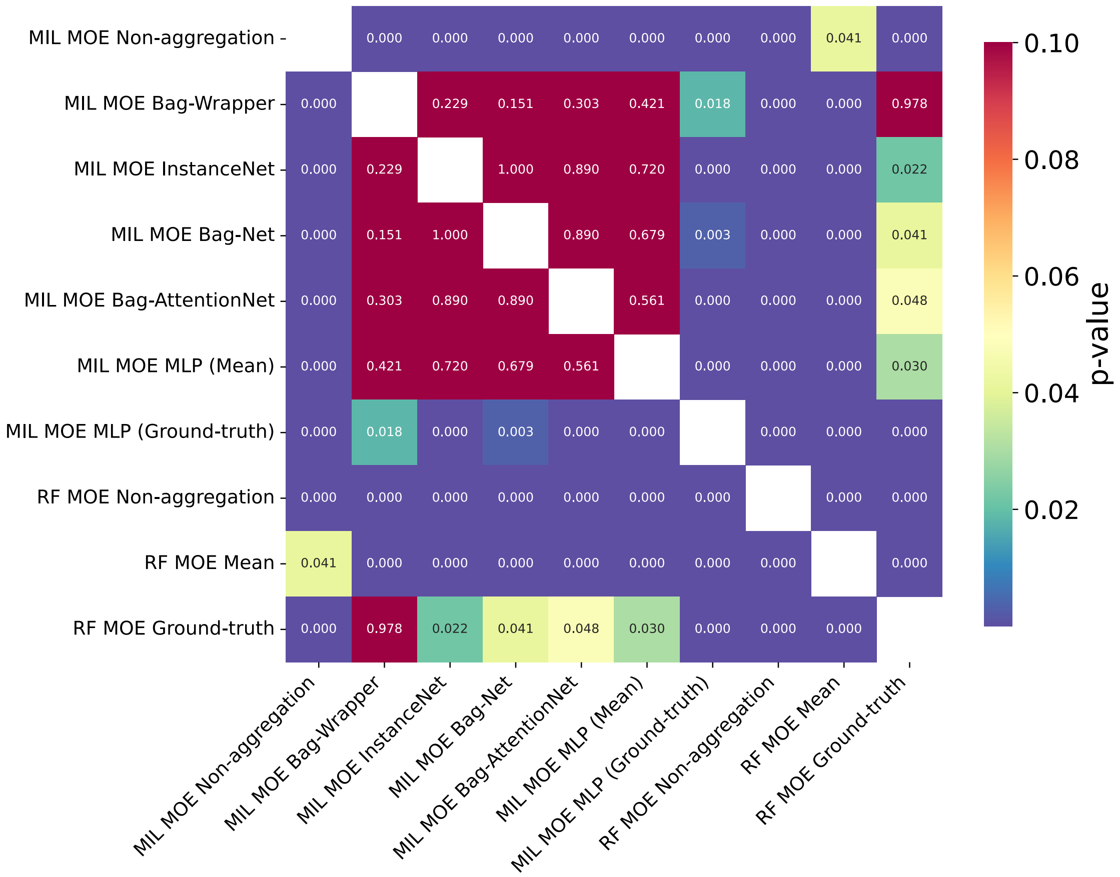

Figure S15. Heatmap of p-values corresponding to Table 2, showing the significance of HOMO prediction accuracy (R^2) for 15 test sets of the PQC data set.

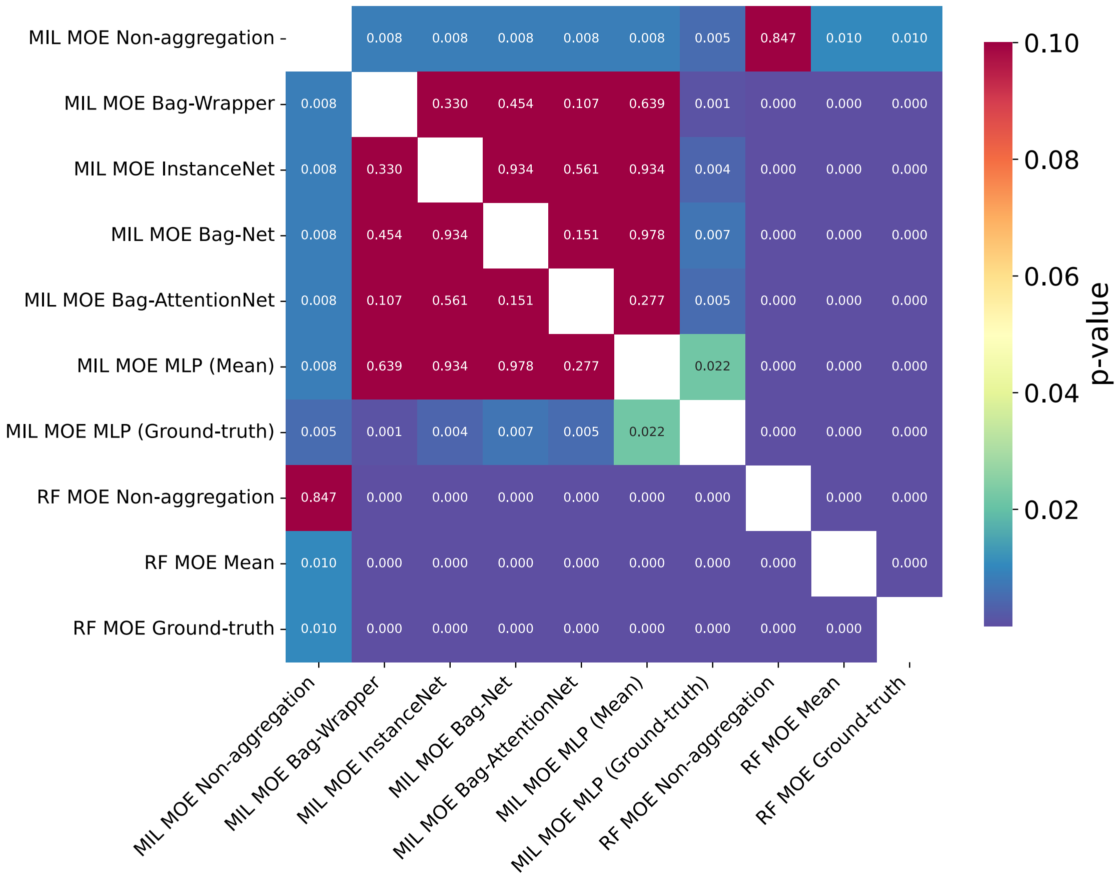

Figure S16. Heatmap of p-values corresponding to Table 2, showing the significance of HOMO-LUMO gap prediction accuracy (R^2) for 15 test sets of the PQC data set.

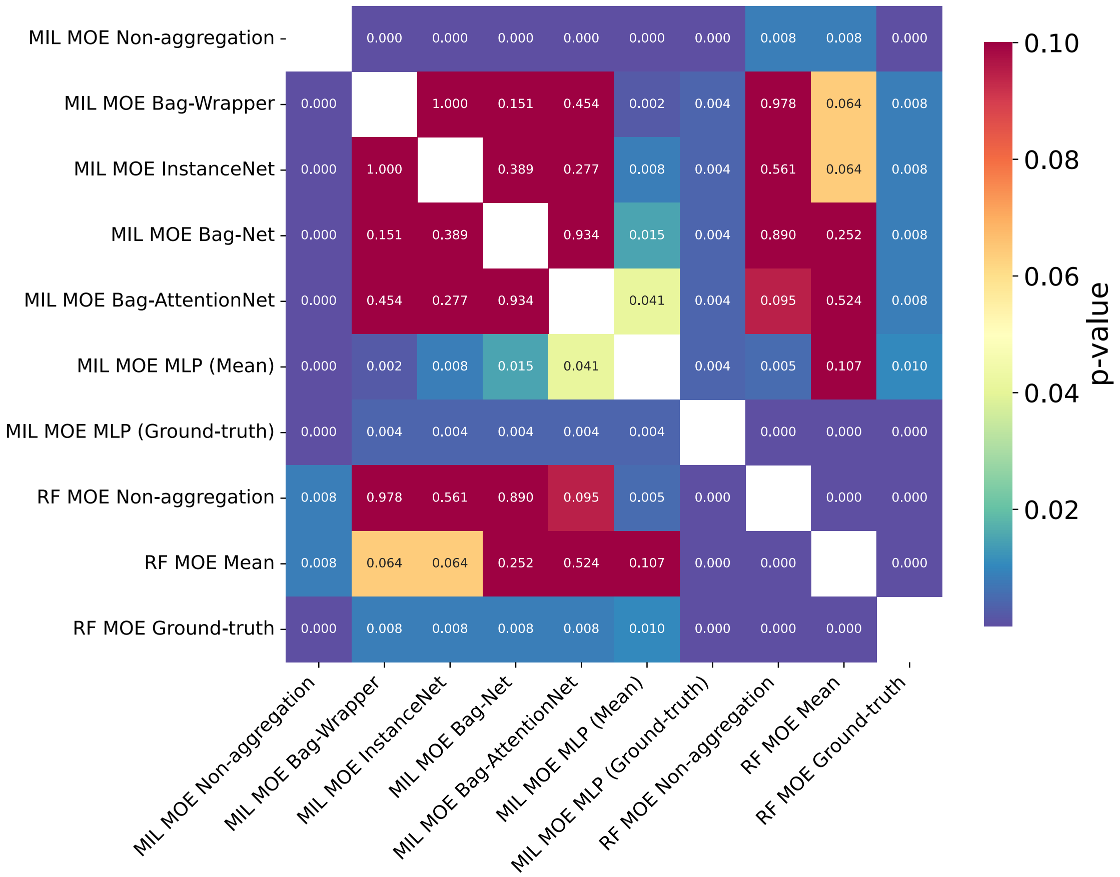

Figure S17. Heatmap of p-values corresponding to Table 2, showing the significance of LUMO prediction accuracy (R^2) for 15 test sets of the PQC data set.

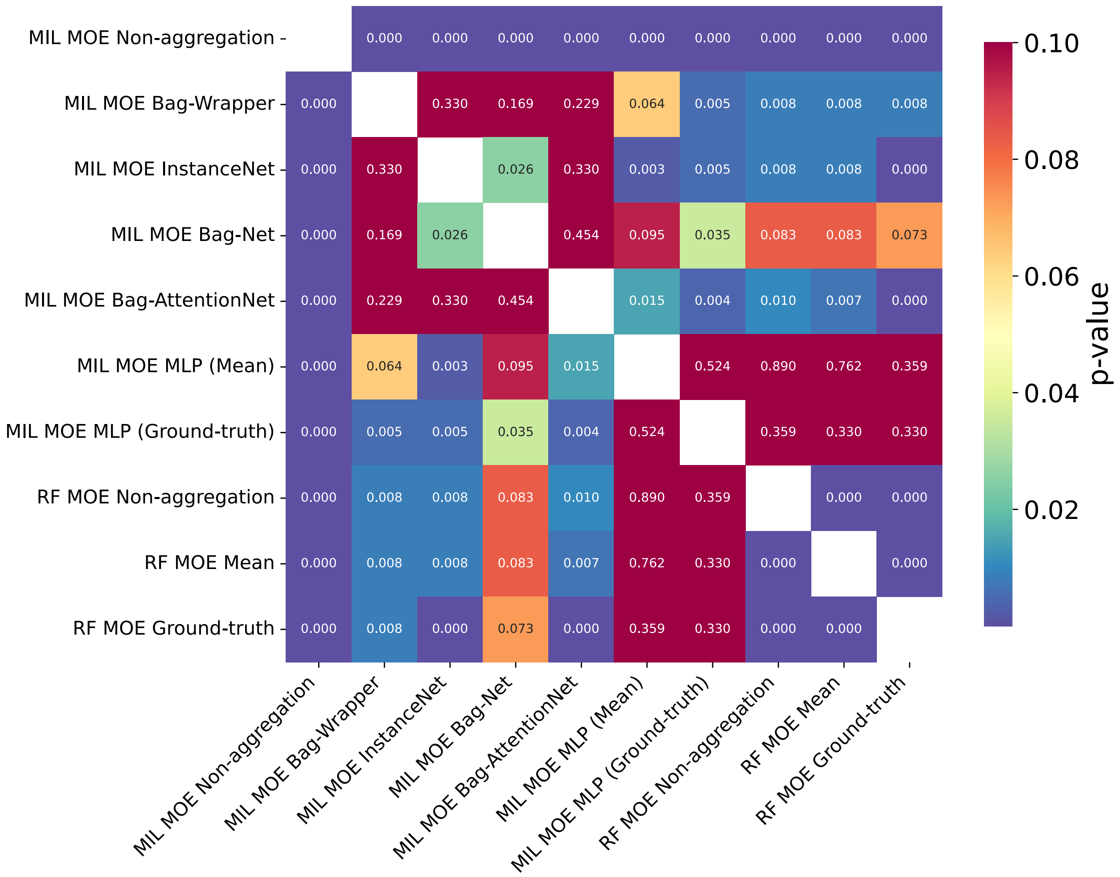

Figure S18. Heatmap of p-values corresponding to Table 2, showing the significance of energy prediction accuracy (R^2) for 15 test sets of the PQC data set.

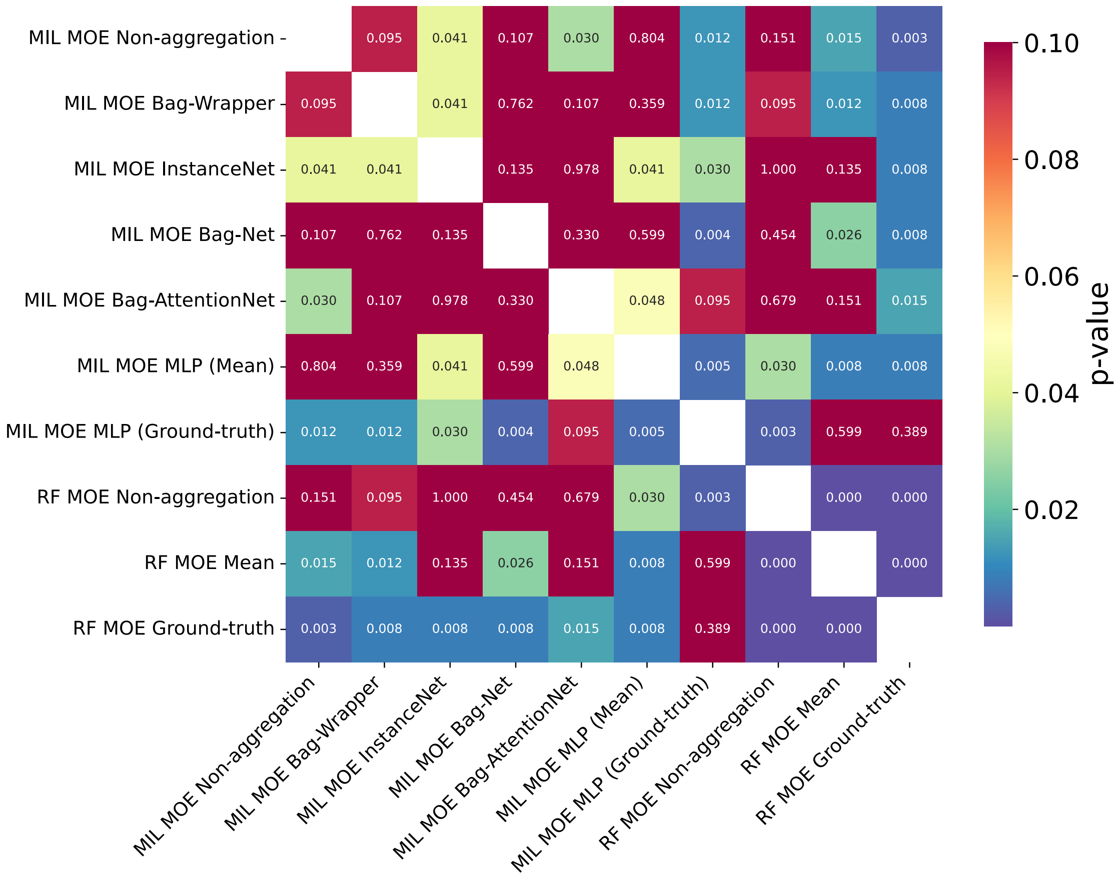

Figure S19. Heatmap of p-values corresponding to Table 2, showing the significance of enthalpy prediction accuracy (R^2) for 15 test sets of the PQC data set.

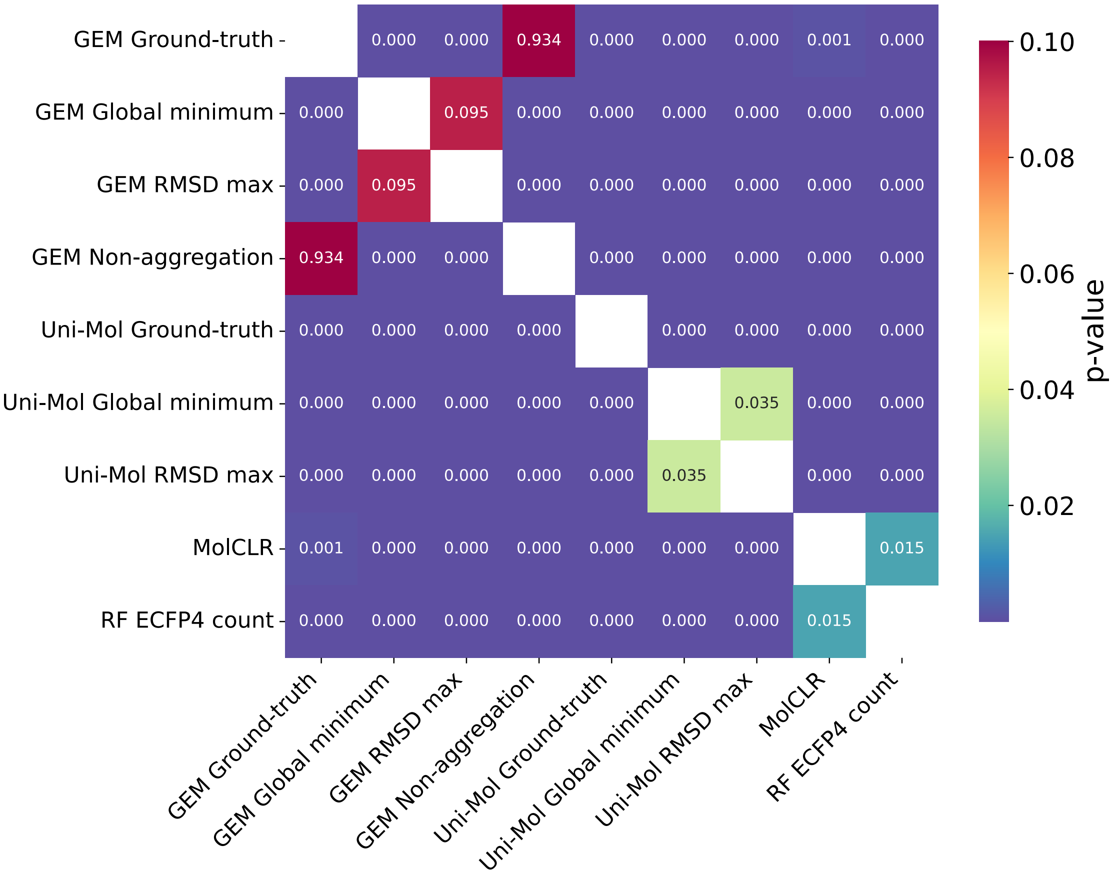

Figure S20. Heatmap of p-values corresponding to Table 3, showing the significance of dipole moment prediction accuracy (R^2) for 15 test sets of the PQC data set.

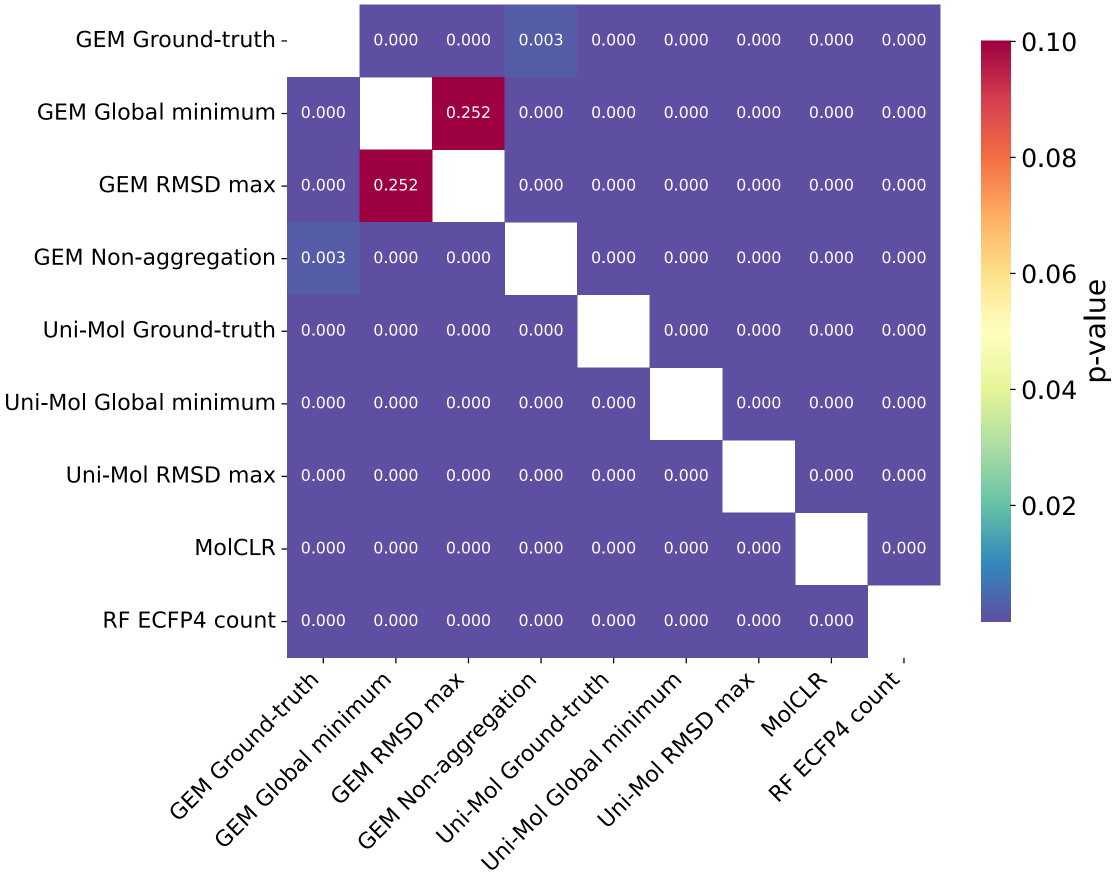

Figure S21. Heatmap of p-values corresponding to Table 3, showing the significance of HOMO prediction accuracy (R^2) for 15 test sets of the PQC data set.

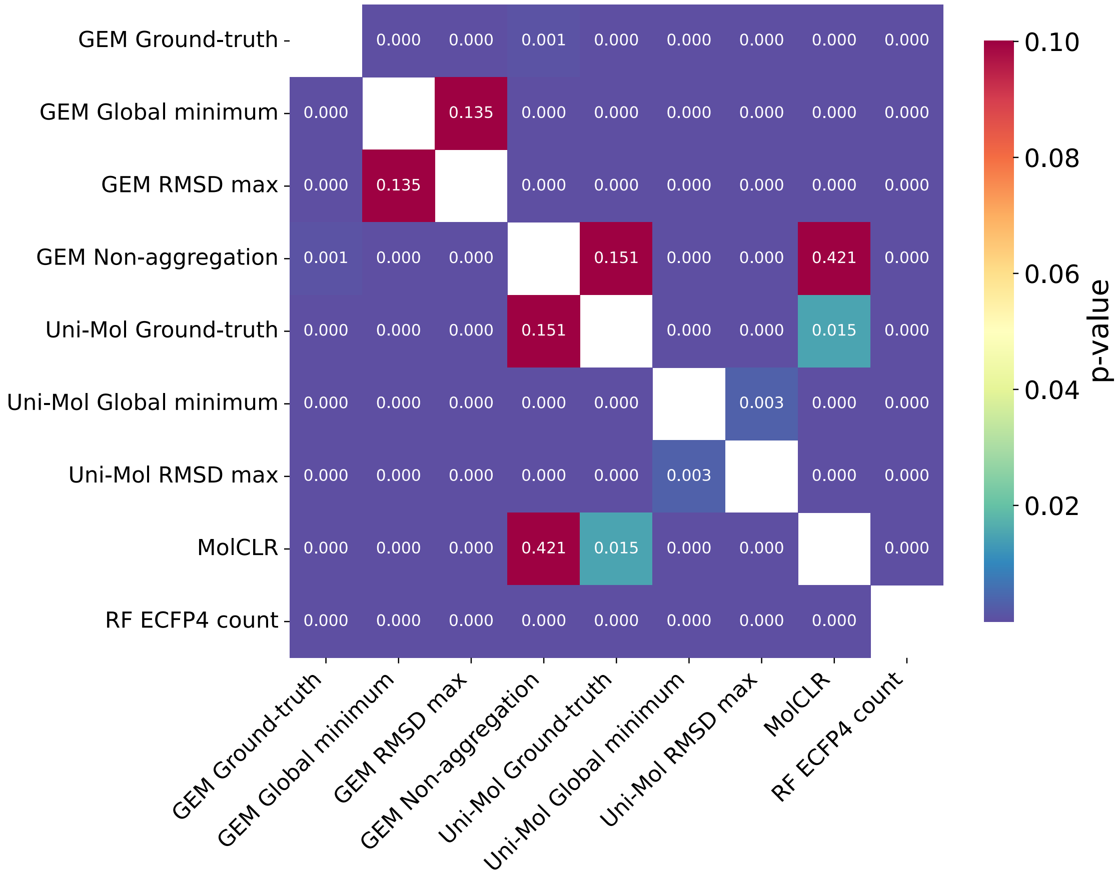

Figure S22. Heatmap of p-values corresponding to Table 3, showing the significance of HOMO-LUMO gap prediction accuracy (R^2) for 15 test sets of the PQC data set.

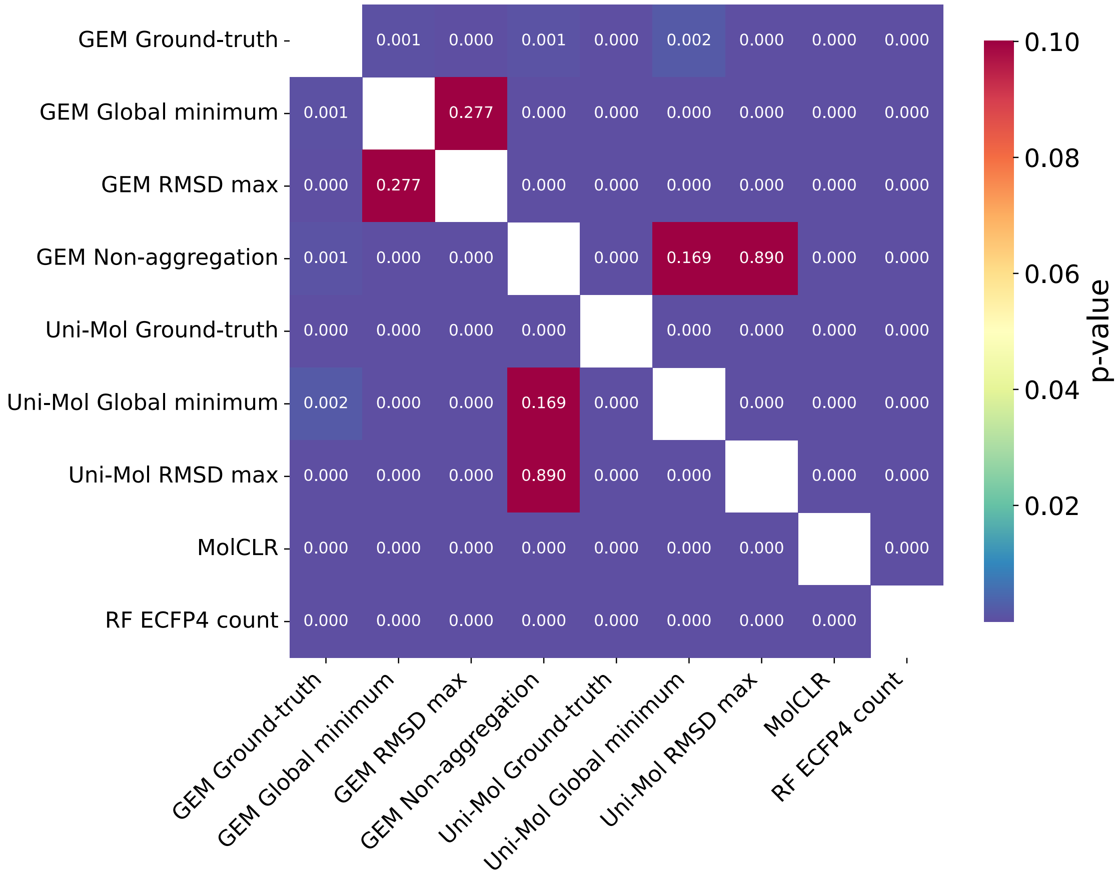

Figure S23. Heatmap of p-values corresponding to Table 3, showing the significance of LUMO prediction accuracy (R^2) for 15 test sets of the PQC data set.

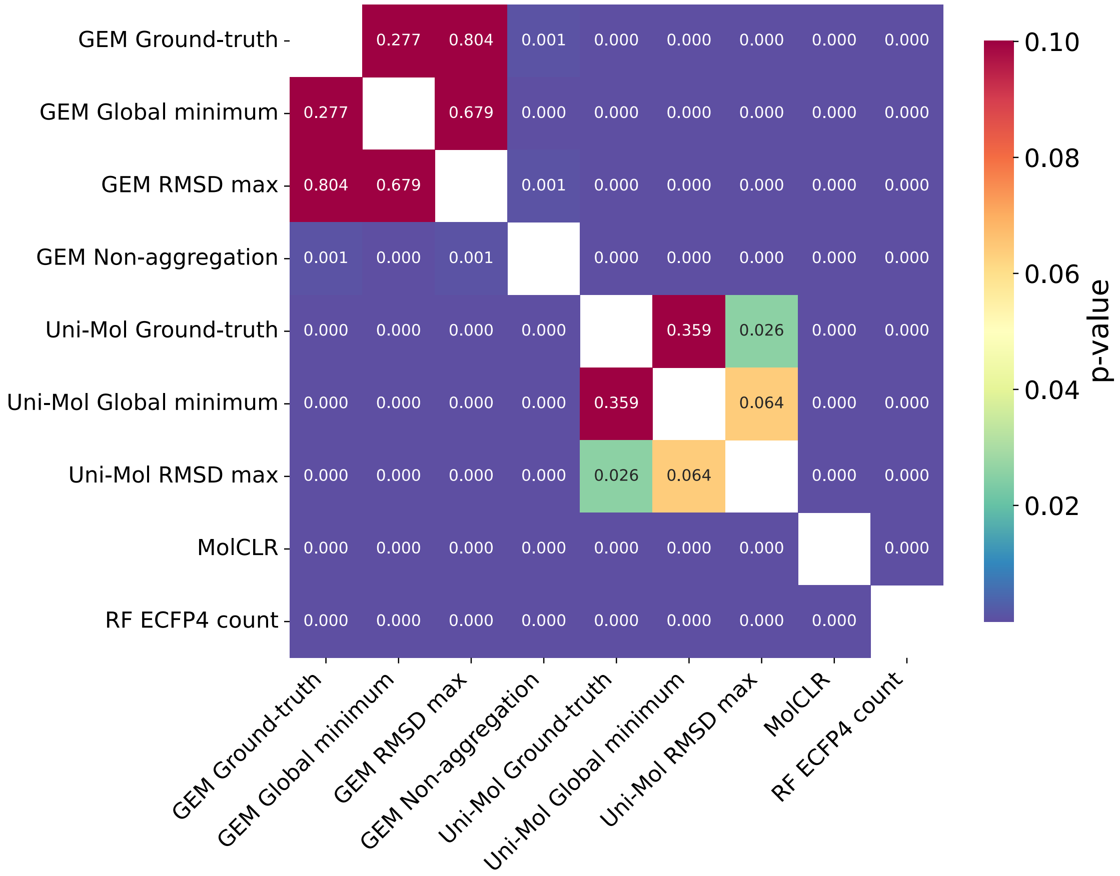

Figure S24. Heatmap of p-values corresponding to Table 3, showing the significance of energy prediction accuracy (R^2) for 15 test sets of the PQC data set.

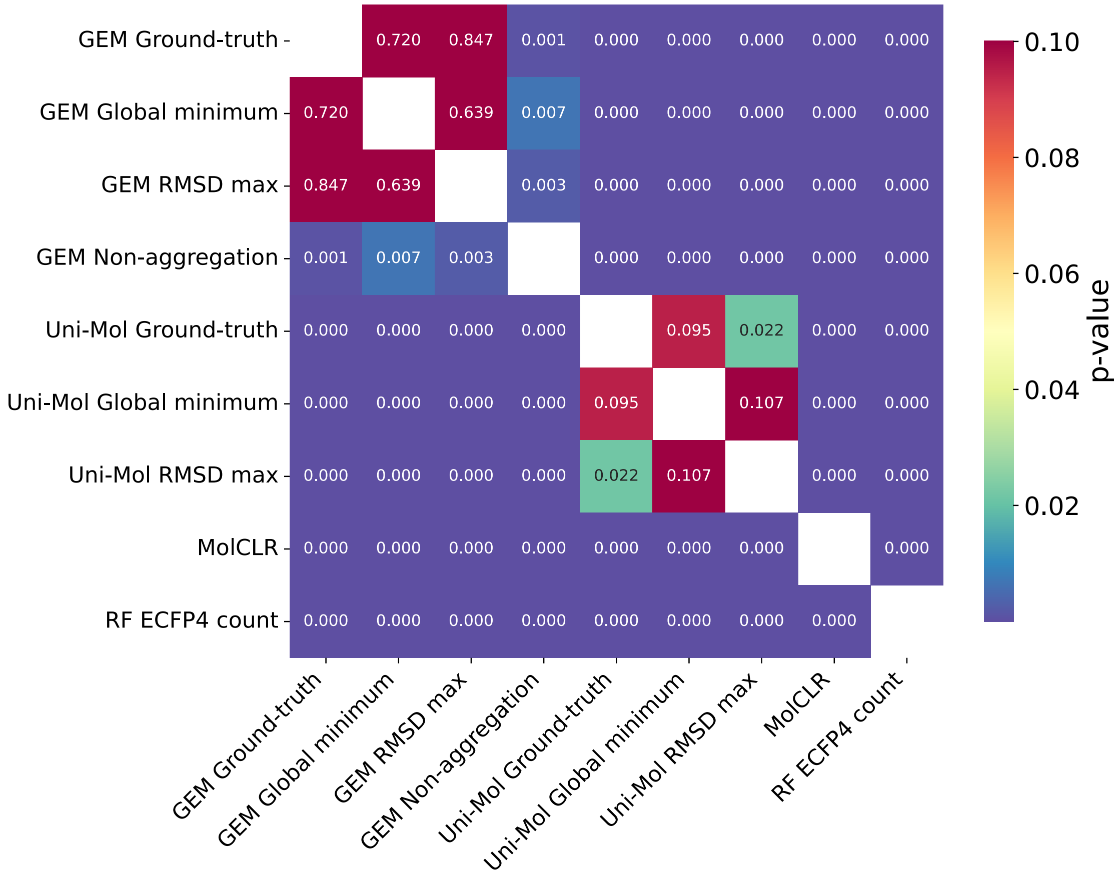

Figure S25. Heatmap of p-values corresponding to Table 3, showing the significance of enthalpy prediction accuracy (R^2) for 15 test sets of the PQC data set.

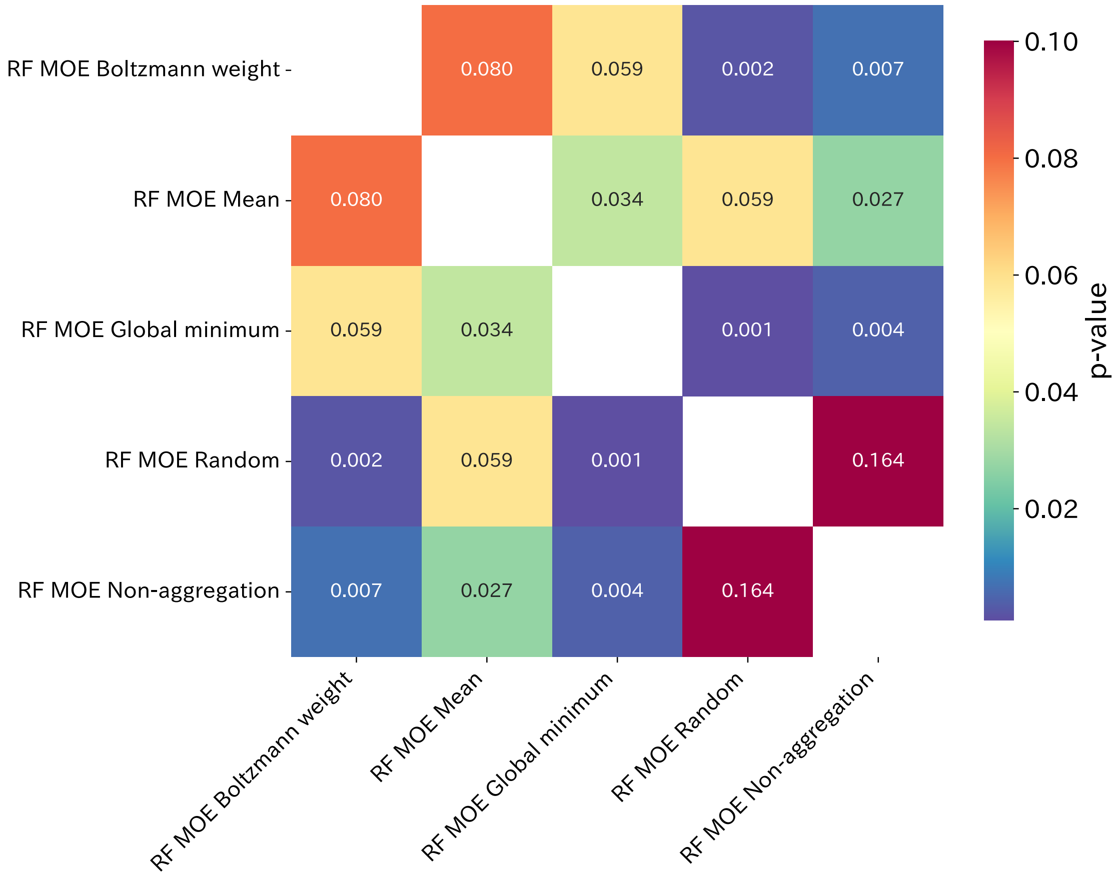

Figure S26. Heatmap of p-values corresponding to Table 4, showing the significance of property (melting point) prediction accuracy (R^2) using MOE descriptors for 25 test sets of the MP data set.

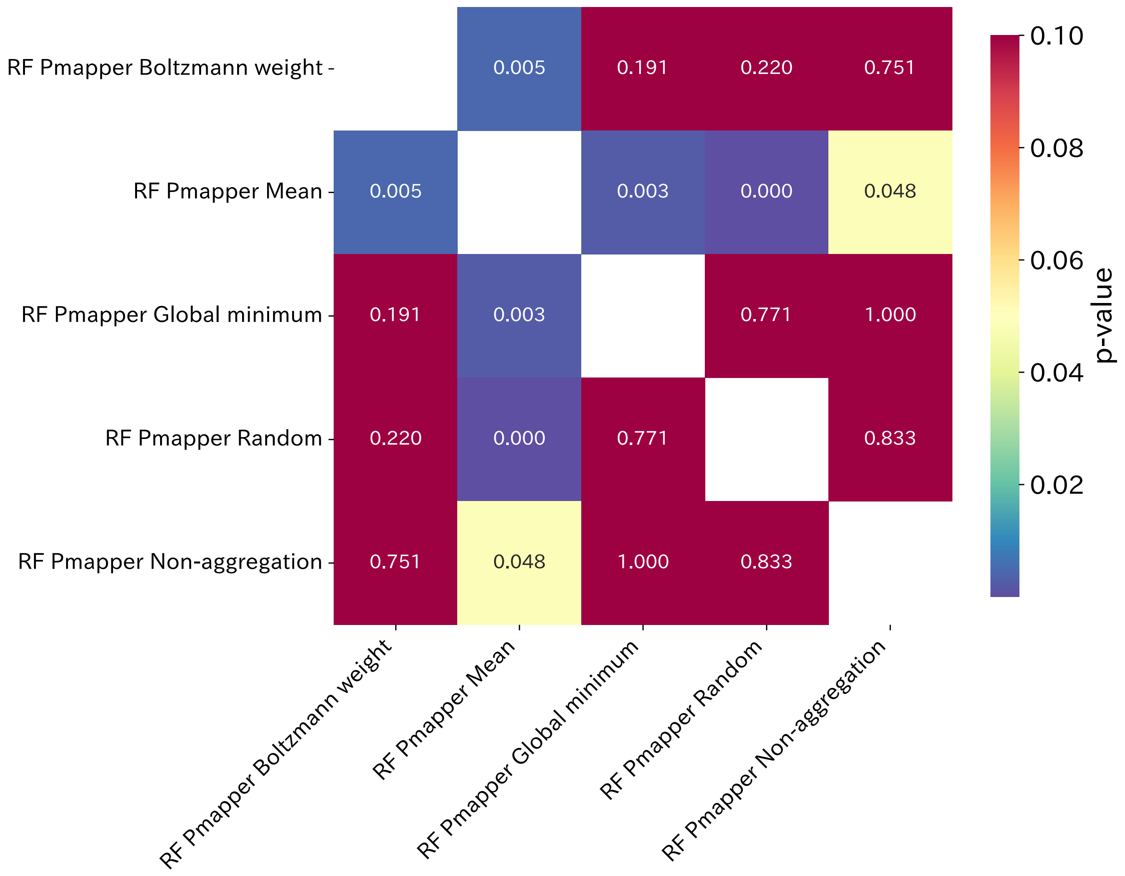

Figure S27. Heatmap of p-values corresponding to Table 4, showing the significance of property (melting point) prediction accuracy (R^2) using Pmapper descriptors for 25 test sets of the MP data set.

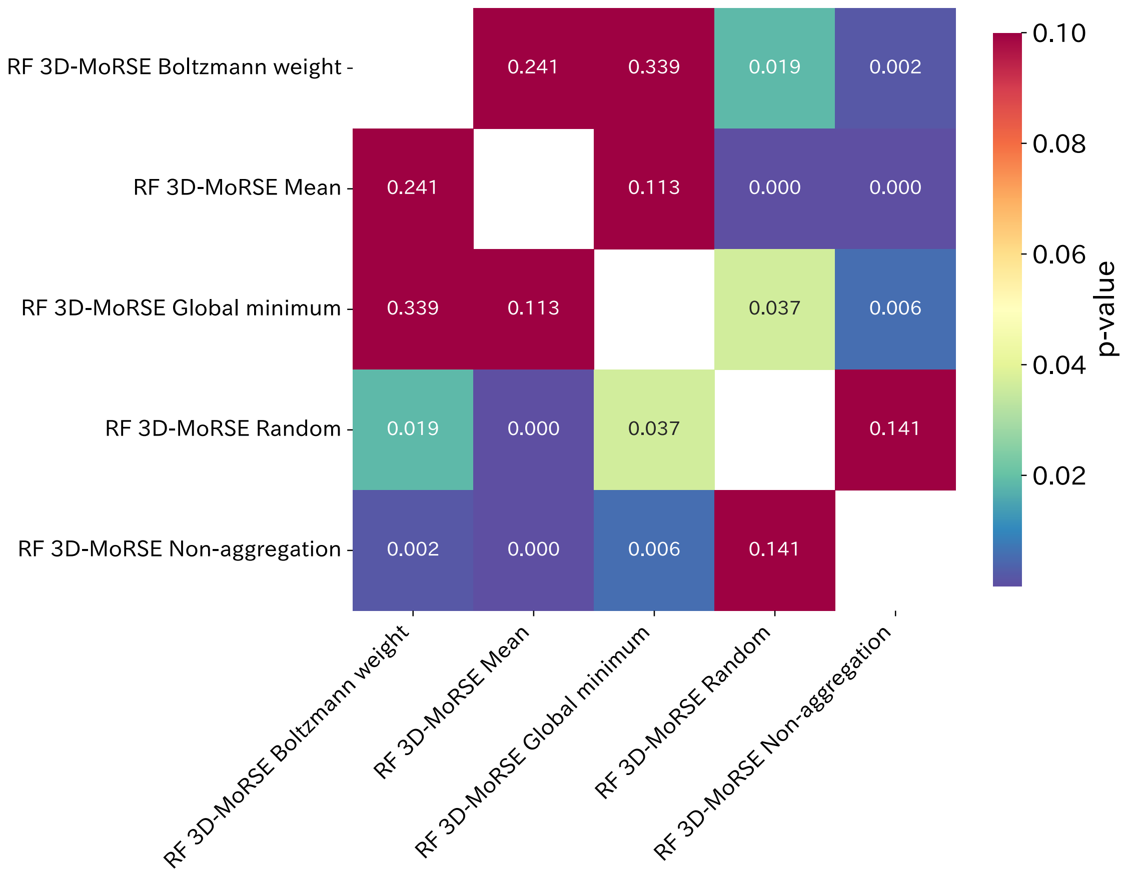

Figure S28. Heatmap of p-values corresponding to Table 4, showing the significance of property (melting point) prediction accuracy (R^2) using 3D-MoRSE descriptors for 25 test sets of the MP data set.

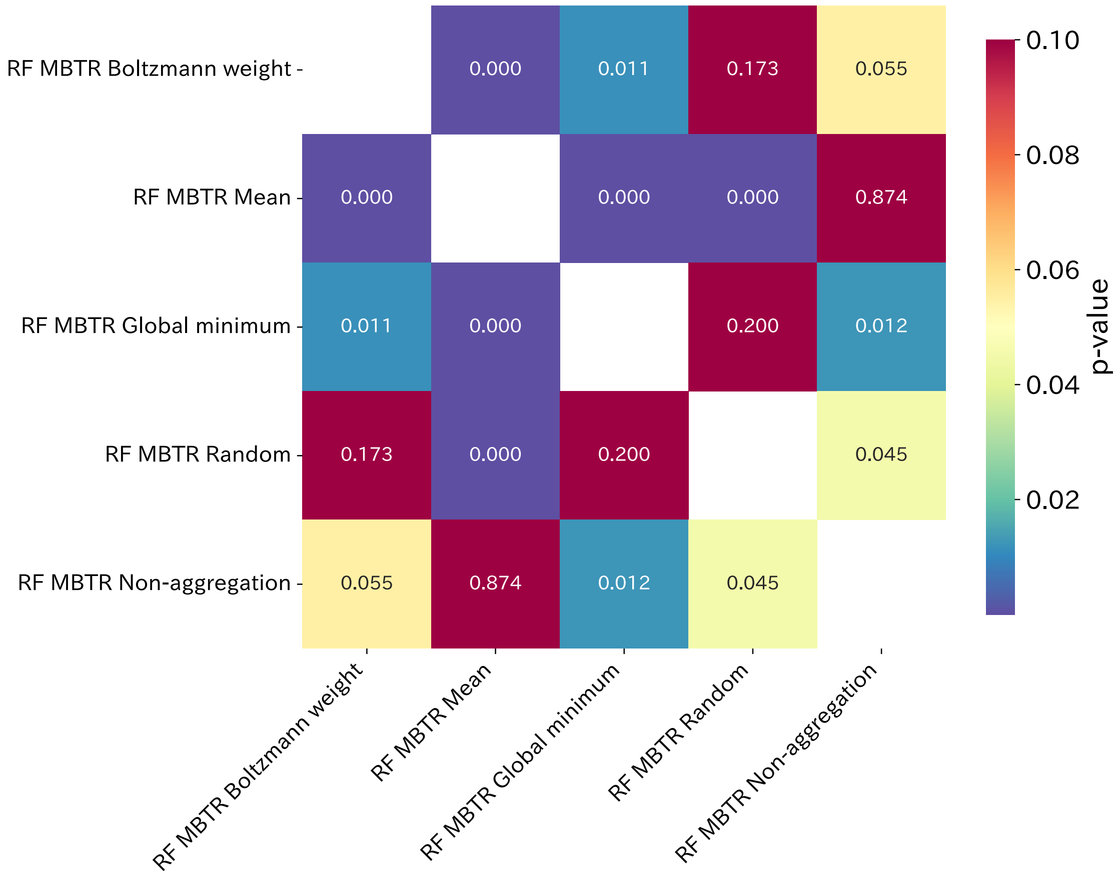

Figure S29. Heatmap of p-values corresponding to Table 4, showing the significance of property (melting point) prediction accuracy (R^2) using MBTR descriptors for 25 test sets of the MP data set.

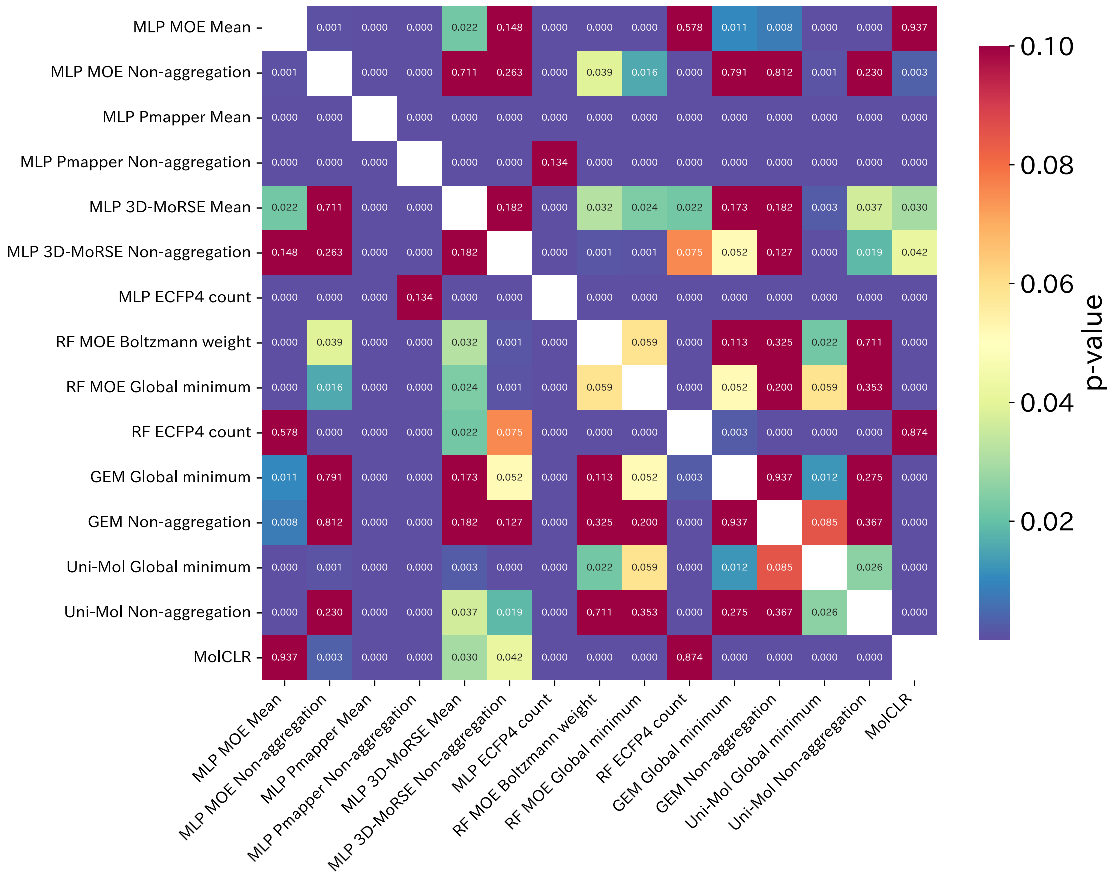

Figure S30. Heatmap of p-values corresponding to Table 5, showing the significance of property (melting point) prediction accuracy (R^2) for 25 test sets of the MP data set.

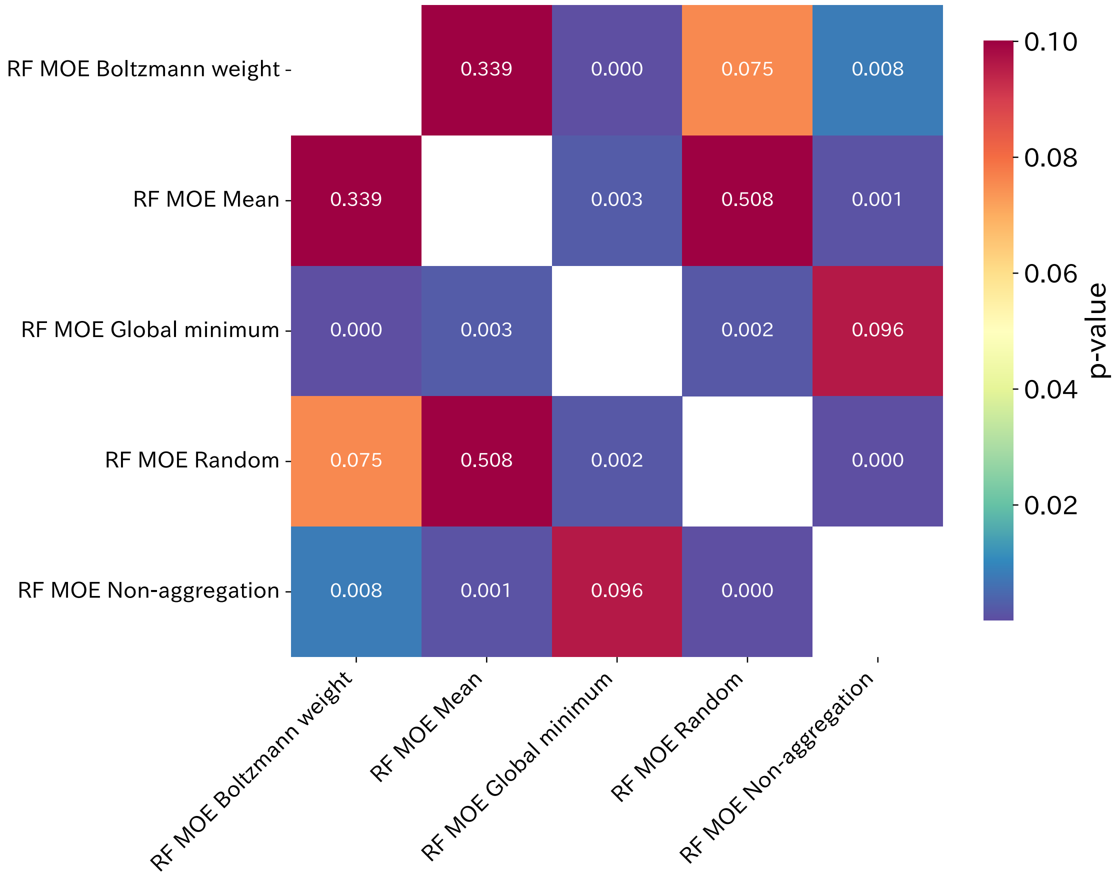

Figure S31. Heatmap of p-values corresponding to Table 6, showing the significance of property ($\Delta\Delta G^{\ddagger}$) prediction accuracy ($R^2$) using MOE descriptors for 25 test sets of the APTC-1 data set.

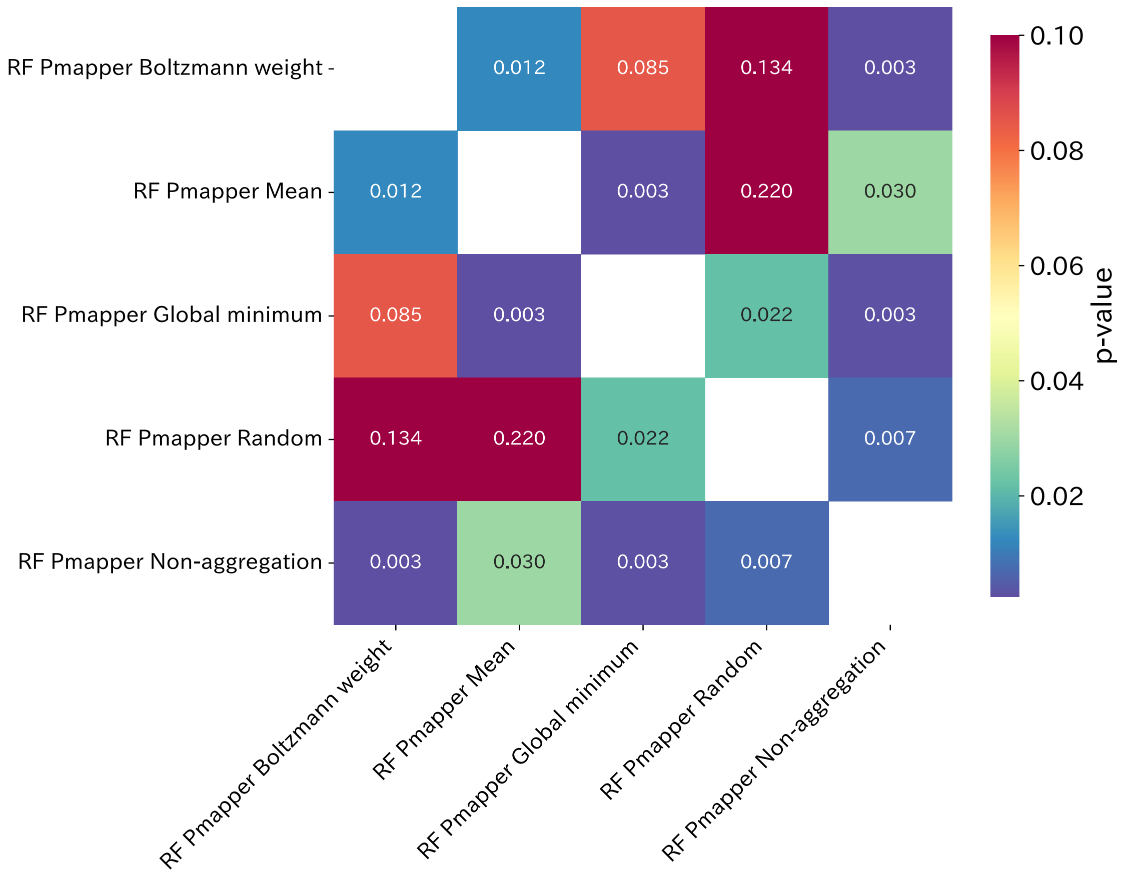

Figure S32. Heatmap of p-values corresponding to Table 6, showing the significance of property ($\Delta\Delta G^{\ddagger}$) prediction accuracy ($R^2$) using Pmapper descriptors for 25 test sets of the APTC-1 data set.

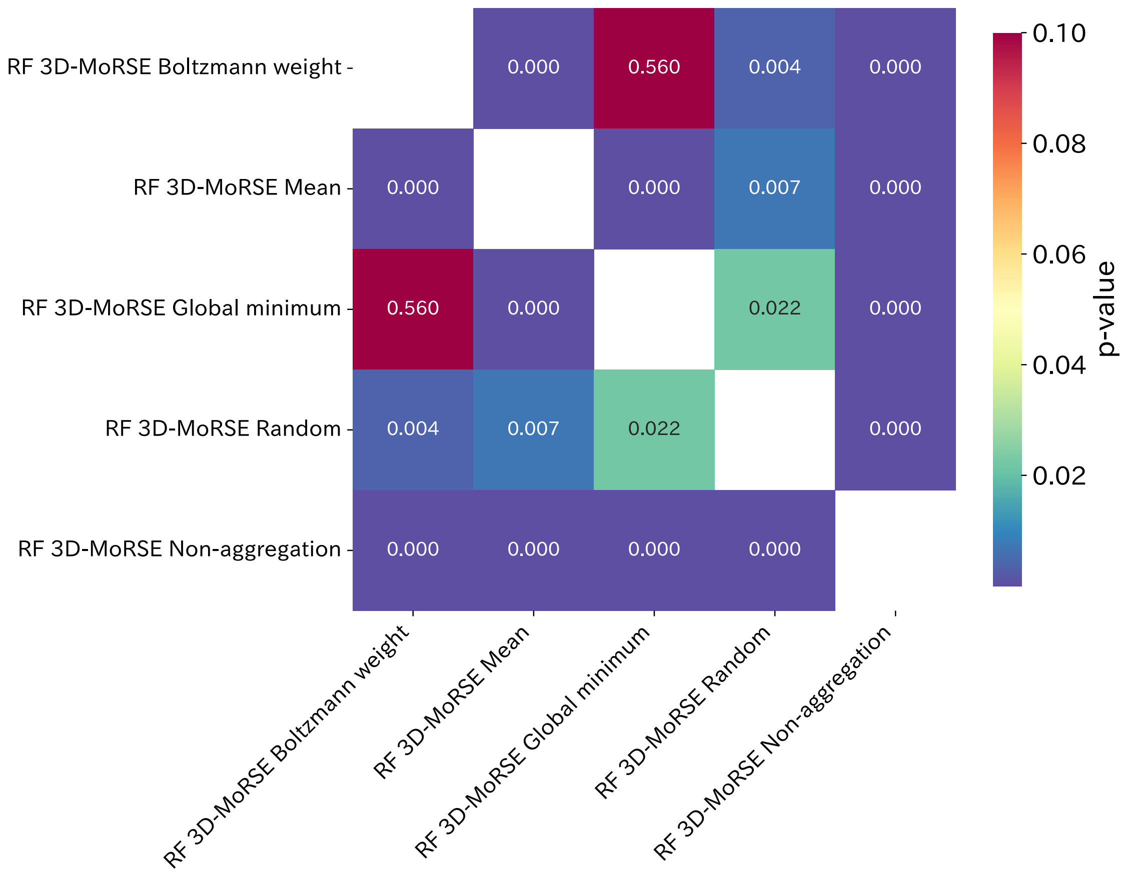

Figure S33. Heatmap of p-values corresponding to Table 6, showing the significance of property ($\Delta\Delta G^{\ddagger}$) prediction accuracy ($R^2$) using 3D-MoRSE descriptors for 25 test sets of the APTC-1 data set.

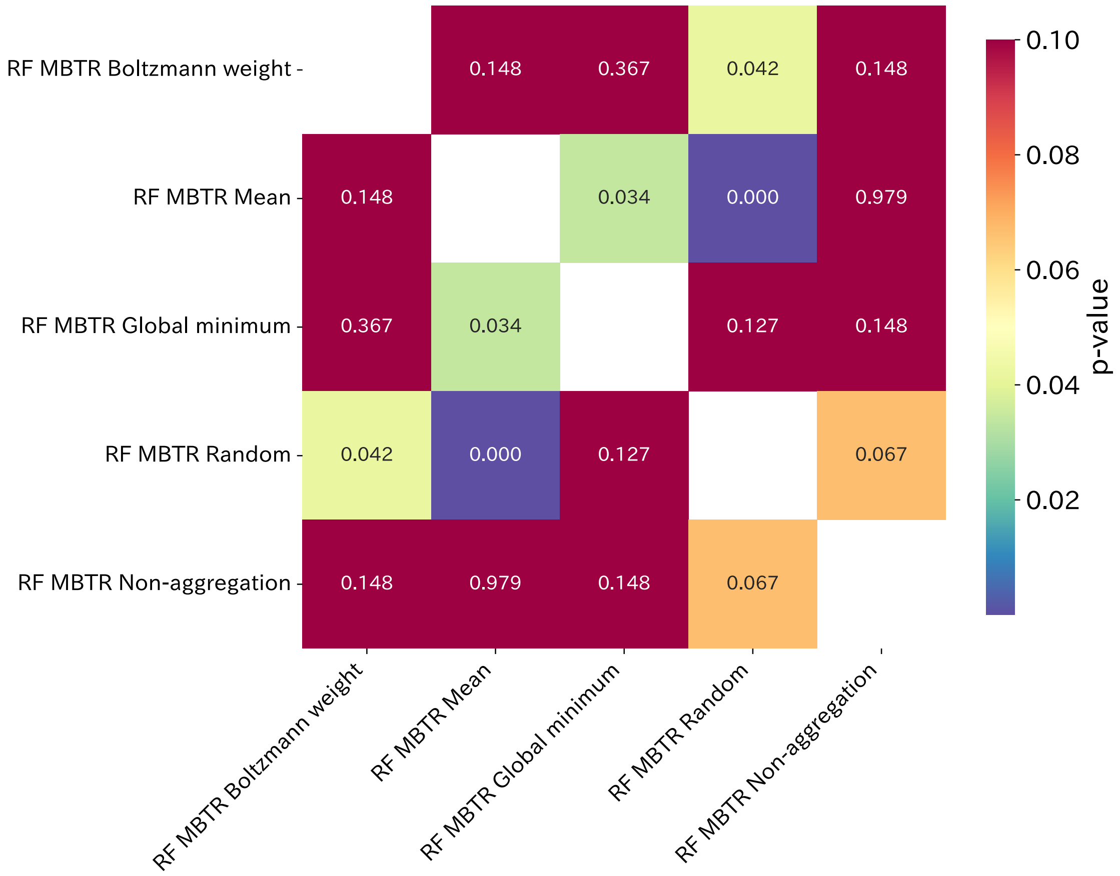

Figure S34. Heatmap of p-values corresponding to Table 6, showing the significance of property ($\Delta\Delta G^{\ddagger}$) prediction accuracy ($R^2$) using MBTR descriptors for 25 test sets of the APTC-1 data set.

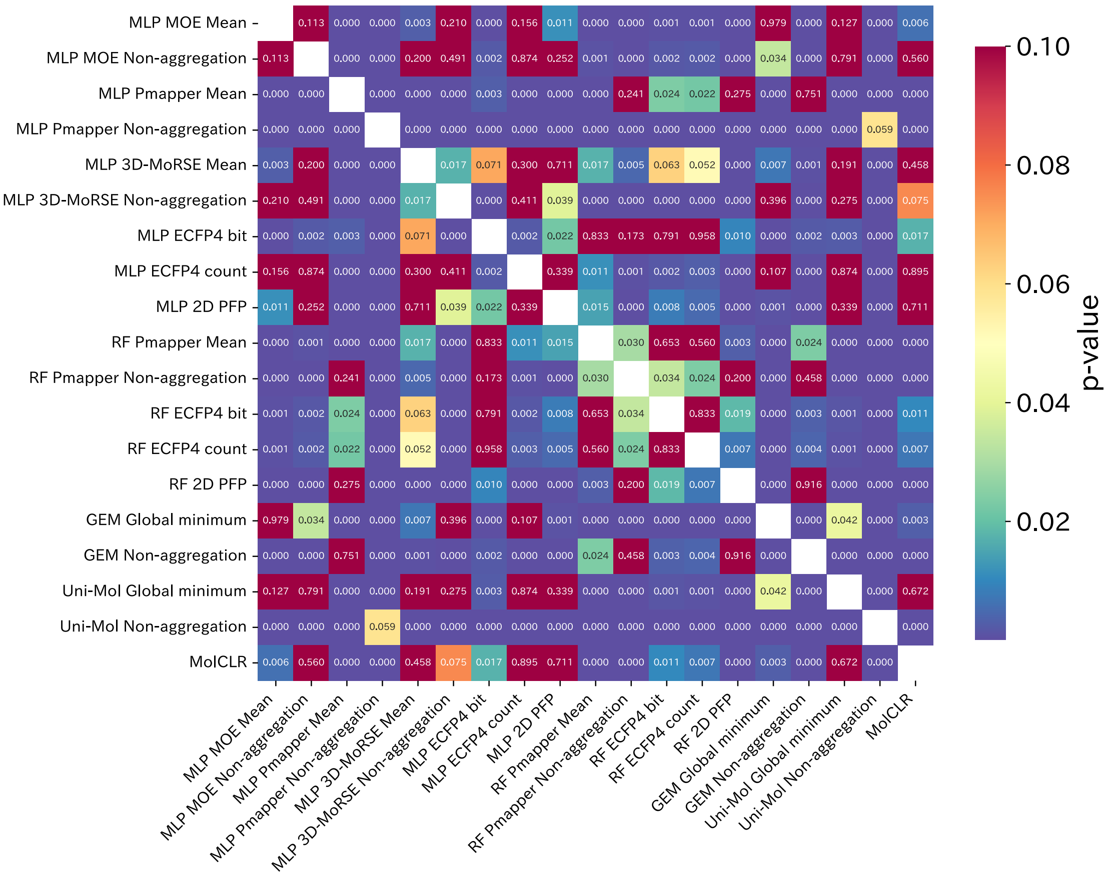

Figure S35. Heatmap of p-values corresponding to Table 7, showing the significance of property ($\Delta\Delta G^{\ddagger}$) prediction accuracy ($R^2$) for 25 test sets of the APTC-1 data set.

Table S178. Order of models in Figure S36 to S70. Left to right in violin plots.

PQC data set			
**Index**	**Model**	**Descriptor**	**Aggregation method or Selected conformation**
1	MIL, Bag-AttentionNet	ECFP4 count	N/A
2	MIL, Bag-Net	ECFP4 count	N/A
3	MIL, Bag-Wrapper	ECFP4 count	N/A
4	MIL, Instance-Net	ECFP4 count	N/A
5	MIL, Non-aggregation (Instance-Wrapper)	ECFP4 count	N/A
6	MIL, Bag-AttentionNet	MBTR	Boltzmann weight
7	MIL, Bag-Net	MBTR	Boltzmann weight
8	MIL, Bag-Wrapper	MBTR	Boltzmann weight
9	MIL, Instance-Net	MBTR	Boltzmann weight
10	MIL, Non-aggregation (Instance-Wrapper)	MBTR	Boltzmann weight
11	MIL, Bag-AttentionNet	MBTR	Ground-truth
12	MIL, Bag-Net	MBTR	Ground-truth
13	MIL, Bag-Wrapper	MBTR	Ground-truth
14	MIL, Instance-Net	MBTR	Ground-truth
15	MIL, Non-aggregation (Instance-Wrapper)	MBTR	Ground-truth
16	MIL, Bag-AttentionNet	MBTR	Global minimum
17	MIL, Bag-Net	MBTR	Global minimum
18	MIL, Bag-Wrapper	MBTR	Global minimum
19	MIL, Instance-Net	MBTR	Global minimum
20	MIL, Non-aggregation (Instance-Wrapper)	MBTR	Global minimum
21	MIL, Bag-AttentionNet	MBTR	Mean
22	MIL, Bag-Net	MBTR	Mean
23	MIL, Bag-Wrapper	MBTR	Mean
24	MIL, Instance-Net	MBTR	Mean
25	MIL, Non-aggregation (Instance-Wrapper)	MBTR	Mean
26	MIL, Bag-AttentionNet	MBTR	Non-aggregation
27	MIL, Bag-Net	MBTR	Non-aggregation
28	MIL, Bag-Wrapper	MBTR	Non-aggregation
29	MIL, Instance-Net	MBTR	Non-aggregation
30	MIL, Non-aggregation (Instance-Wrapper)	MBTR	Non-aggregation
31	MIL, Bag-AttentionNet	MBTR	Random

32	MIL, Bag-Net	MBTR	Random
33	MIL, Bag-Wrapper	MBTR	Random
34	MIL, Instance-Net	MBTR	Random
35	MIL, Non-aggregation (Instance-Wrapper)	MBTR	Random
36	MIL, Bag-AttentionNet	MBTR	RMSD max
37	MIL, Bag-Net	MBTR	RMSD max
38	MIL, Bag-Wrapper	MBTR	RMSD max
39	MIL, Instance-Net	MBTR	RMSD max
40	MIL, Non-aggregation (Instance-Wrapper)	MBTR	RMSD max
41	MIL, Bag-AttentionNet	MBTR	RMSD min
42	MIL, Bag-Net	MBTR	RMSD min
43	MIL, Bag-Wrapper	MBTR	RMSD min
44	MIL, Instance-Net	MBTR	RMSD min
45	MIL, Non-aggregation (Instance-Wrapper)	MBTR	RMSD min
46	MIL, Bag-AttentionNet	MOE	Boltzmann weight
47	MIL, Bag-Net	MOE	Boltzmann weight
48	MIL, Bag-Wrapper	MOE	Boltzmann weight
49	MIL, Instance-Net	MOE	Boltzmann weight
50	MIL, Non-aggregation (Instance-Wrapper)	MOE	Boltzmann weight
51	MIL, Bag-AttentionNet	MOE	Ground-truth
52	MIL, Bag-Net	MOE	Ground-truth
53	MIL, Bag-Wrapper	MOE	Ground-truth
54	MIL, Instance-Net	MOE	Ground-truth
55	MIL, Non-aggregation (Instance-Wrapper)	MOE	Ground-truth
56	MIL, Bag-AttentionNet	MOE	Global minimum
57	MIL, Bag-Net	MOE	Global minimum
58	MIL, Bag-Wrapper	MOE	Global minimum
59	MIL, Instance-Net	MOE	Global minimum
60	MIL, Non-aggregation (Instance-Wrapper)	MOE	Global minimum
61	MIL, Bag-AttentionNet	MOE	Mean
62	MIL, Bag-Net	MOE	Mean
63	MIL, Bag-Wrapper	MOE	Mean
64	MIL, Instance-Net	MOE	Mean
65	MIL, Non-aggregation (Instance-Wrapper)	MOE	Mean
66	MIL, Bag-AttentionNet	MOE	Non-aggregation

67	MIL, Bag-Net	MOE	Non-aggregation
68	MIL, Bag-Wrapper	MOE	Non-aggregation
69	MIL, Instance-Net	MOE	Non-aggregation
70	MIL, Non-aggregation (Instance-Wrapper)	MOE	Non-aggregation
71	MIL, Bag-AttentionNet	MOE	Random
72	MIL, Bag-Net	MOE	Random
73	MIL, Bag-Wrapper	MOE	Random
74	MIL, Instance-Net	MOE	Random
75	MIL, Non-aggregation (Instance-Wrapper)	MOE	Random
76	MIL, Bag-AttentionNet	MOE	RMSD max
77	MIL, Bag-Net	MOE	RMSD max
78	MIL, Bag-Wrapper	MOE	RMSD max
79	MIL, Instance-Net	MOE	RMSD max
80	MIL, Non-aggregation (Instance-Wrapper)	MOE	RMSD max
81	MIL, Bag-AttentionNet	MOE	RMSD min
82	MIL, Bag-Net	MOE	RMSD min
83	MIL, Bag-Wrapper	MOE	RMSD min
84	MIL, Instance-Net	MOE	RMSD min
85	MIL, Non-aggregation (Instance-Wrapper)	MOE	RMSD min
86	MIL, Bag-AttentionNet	3D-MoRSE	Boltzmann weight
87	MIL, Bag-Net	3D-MoRSE	Boltzmann weight
88	MIL, Bag-Wrapper	3D-MoRSE	Boltzmann weight
89	MIL, Instance-Net	3D-MoRSE	Boltzmann weight
90	MIL, Non-aggregation (Instance-Wrapper)	3D-MoRSE	Boltzmann weight
91	MIL, Bag-AttentionNet	3D-MoRSE	Ground-truth
92	MIL, Bag-Net	3D-MoRSE	Ground-truth
93	MIL, Bag-Wrapper	3D-MoRSE	Ground-truth
94	MIL, Instance-Net	3D-MoRSE	Ground-truth
95	MIL, Non-aggregation (Instance-Wrapper)	3D-MoRSE	Ground-truth
96	MIL, Bag-AttentionNet	3D-MoRSE	Global minimum
97	MIL, Bag-Net	3D-MoRSE	Global minimum
98	MIL, Bag-Wrapper	3D-MoRSE	Global minimum
99	MIL, Instance-Net	3D-MoRSE	Global minimum
100	MIL, Non-aggregation (Instance-Wrapper)	3D-MoRSE	Global minimum
101	MIL, Bag-AttentionNet	3D-MoRSE	Mean

102	MIL, Bag-Net	3D-MoRSE	Mean
103	MIL, Bag-Wrapper	3D-MoRSE	Mean
104	MIL, Instance-Net	3D-MoRSE	Mean
105	MIL, Non-aggregation (Instance-Wrapper)	3D-MoRSE	Mean
106	MIL, Bag-AttentionNet	3D-MoRSE	Non-aggregation
107	MIL, Bag-Net	3D-MoRSE	Non-aggregation
108	MIL, Bag-Wrapper	3D-MoRSE	Non-aggregation
109	MIL, Instance-Net	3D-MoRSE	Non-aggregation
110	MIL, Non-aggregation (Instance-Wrapper)	3D-MoRSE	Non-aggregation
111	MIL, Bag-AttentionNet	3D-MoRSE	Random
112	MIL, Bag-Net	3D-MoRSE	Random
113	MIL, Bag-Wrapper	3D-MoRSE	Random
114	MIL, Instance-Net	3D-MoRSE	Random
115	MIL, Non-aggregation (Instance-Wrapper)	3D-MoRSE	Random
116	MIL, Bag-AttentionNet	3D-MoRSE	RMSD max
117	MIL, Bag-Net	3D-MoRSE	RMSD max
118	MIL, Bag-Wrapper	3D-MoRSE	RMSD max
119	MIL, Instance-Net	3D-MoRSE	RMSD max
120	MIL, Non-aggregation (Instance-Wrapper)	3D-MoRSE	RMSD max
121	MIL, Bag-AttentionNet	3D-MoRSE	RMSD min
122	MIL, Bag-Net	3D-MoRSE	RMSD min
123	MIL, Bag-Wrapper	3D-MoRSE	RMSD min
124	MIL, Instance-Net	3D-MoRSE	RMSD min
125	MIL, Non-aggregation (Instance-Wrapper)	3D-MoRSE	RMSD min
126	MIL, Bag-AttentionNet	Pmapper	Boltzmann weight
127	MIL, Bag-Net	Pmapper	Boltzmann weight
128	MIL, Bag-Wrapper	Pmapper	Boltzmann weight
129	MIL, Instance-Net	Pmapper	Boltzmann weight
130	MIL, Non-aggregation (Instance-Wrapper)	Pmapper	Boltzmann weight
131	MIL, Bag-AttentionNet	Pmapper	Ground-truth
132	MIL, Bag-Net	Pmapper	Ground-truth
133	MIL, Bag-Wrapper	Pmapper	Ground-truth
134	MIL, Instance-Net	Pmapper	Ground-truth
135	MIL, Non-aggregation (Instance-Wrapper)	Pmapper	Ground-truth
136	MIL, Bag-AttentionNet	Pmapper	Global minimum

137	MIL, Bag-Net	Pmapper	Global minimum
138	MIL, Bag-Wrapper	Pmapper	Global minimum
139	MIL, Instance-Net	Pmapper	Global minimum
140	MIL, Non-aggregation (Instance-Wrapper)	Pmapper	Global minimum
141	MIL, Bag-AttentionNet	Pmapper	Mean
142	MIL, Bag-Net	Pmapper	Mean
143	MIL, Bag-Wrapper	Pmapper	Mean
144	MIL, Instance-Net	Pmapper	Mean
145	MIL, Non-aggregation (Instance-Wrapper)	Pmapper	Mean
146	MIL, Bag-AttentionNet	Pmapper	Non-aggregation
147	MIL, Bag-Net	Pmapper	Non-aggregation
148	MIL, Bag-Wrapper	Pmapper	Non-aggregation
149	MIL, Instance-Net	Pmapper	Non-aggregation
150	MIL, Non-aggregation (Instance-Wrapper)	Pmapper	Non-aggregation
151	MIL, Bag-AttentionNet	Pmapper	Random
152	MIL, Bag-Net	Pmapper	Random
153	MIL, Bag-Wrapper	Pmapper	Random
154	MIL, Instance-Net	Pmapper	Random
155	MIL, Non-aggregation (Instance-Wrapper)	Pmapper	Random
156	MIL, Bag-AttentionNet	Pmapper	RMSD max
157	MIL, Bag-Net	Pmapper	RMSD max
158	MIL, Bag-Wrapper	Pmapper	RMSD max
159	MIL, Instance-Net	Pmapper	RMSD max
160	MIL, Non-aggregation (Instance-Wrapper)	Pmapper	RMSD max
161	MIL, Bag-AttentionNet	Pmapper	RMSD min
162	MIL, Bag-Net	Pmapper	RMSD min
163	MIL, Bag-Wrapper	Pmapper	RMSD min
164	MIL, Instance-Net	Pmapper	RMSD min
165	MIL, Non-aggregation (Instance-Wrapper)	Pmapper	RMSD min
166	RF	ECFP4 count	N/A
167	RF	MBTR	Boltzmann weight
168	RF	MBTR	Ground-truth
169	RF	MBTR	Global minimum
170	RF	MBTR	Mean
171	RF	MBTR	Non-aggregation

172	RF	MBTR	Random
173	RF	MBTR	RMSD max
174	RF	MBTR	RMSD min
175	RF	MOE	Boltzmann weight
176	RF	MOE	Ground-truth
177	RF	MOE	Global minimum
178	RF	MOE	Mean
179	RF	MOE	Non-aggregation
180	RF	MOE	Random
181	RF	MOE	RMSD max
182	RF	MOE	RMSD min
183	RF	3D-MoRSE	Boltzmann weight
184	RF	3D-MoRSE	Ground-truth
185	RF	3D-MoRSE	Global minimum
186	RF	3D-MoRSE	Mean
187	RF	3D-MoRSE	Non-aggregation
188	RF	3D-MoRSE	Random
189	RF	3D-MoRSE	RMSD max
190	RF	3D-MoRSE	RMSD min
191	RF	Pmapper	Boltzmann weight
192	RF	Pmapper	Ground-truth
193	RF	Pmapper	Global minimum
194	RF	Pmapper	Mean
195	RF	Pmapper	Non-aggregation
196	RF	Pmapper	Random
197	RF	Pmapper	RMSD max
198	RF	Pmapper	RMSD min
199	GEM	N/A	Global minimum
200	GEM	N/A	Ground-truth
201	GEM	N/A	Non-aggregation
202	GEM	N/A	RMSD max
203	MolCLR	N/A	N/A
204	Uni-Mol	N/A	Global minimum
205	Uni-Mol	N/A	Ground-truth
206	Uni-Mol	N/A	RMSD max

MP data set			
1	MIL, Bag-AttentionNet	ECFP4 count	N/A
2	MIL, Bag-Net	ECFP4 count	N/A
3	MIL, Bag-Wrapper	ECFP4 count	N/A
4	MIL, Instance-Net	ECFP4 count	N/A
5	MIL, Non-aggregation (Instance-Wrapper)	ECFP4 count	N/A
6	MIL, Bag-AttentionNet	MBTR	Boltzmann weight
7	MIL, Bag-Net	MBTR	Boltzmann weight
8	MIL, Bag-Wrapper	MBTR	Boltzmann weight
9	MIL, Instance-Net	MBTR	Boltzmann weight
10	MIL, Non-aggregation (Instance-Wrapper)	MBTR	Boltzmann weight
11	MIL, Bag-AttentionNet	MBTR	Global minimum
12	MIL, Bag-Net	MBTR	Global minimum
13	MIL, Bag-Wrapper	MBTR	Global minimum
14	MIL, Instance-Net	MBTR	Global minimum
15	MIL, Non-aggregation (Instance-Wrapper)	MBTR	Global minimum
16	MIL, Bag-AttentionNet	MBTR	Mean
17	MIL, Bag-Net	MBTR	Mean
18	MIL, Bag-Wrapper	MBTR	Mean
19	MIL, Instance-Net	MBTR	Mean
20	MIL, Non-aggregation (Instance-Wrapper)	MBTR	Mean
21	MIL, Bag-AttentionNet	MBTR	Non-aggregation
22	MIL, Bag-Net	MBTR	Non-aggregation
23	MIL, Bag-Wrapper	MBTR	Non-aggregation
24	MIL, Instance-Net	MBTR	Non-aggregation
25	MIL, Non-aggregation (Instance-Wrapper)	MBTR	Non-aggregation
26	MIL, Bag-AttentionNet	MBTR	Random
27	MIL, Bag-Net	MBTR	Random
28	MIL, Bag-Wrapper	MBTR	Random
29	MIL, Instance-Net	MBTR	Random
30	MIL, Non-aggregation (Instance-Wrapper)	MBTR	Random
31	MIL, Bag-AttentionNet	MOE	Boltzmann weight
32	MIL, Bag-Net	MOE	Boltzmann weight
33	MIL, Bag-Wrapper	MOE	Boltzmann weight
34	MIL, Instance-Net	MOE	Boltzmann weight

35	MIL, Non-aggregation (Instance-Wrapper)	MOE	Boltzmann weight
36	MIL, Bag-AttentionNet	MOE	Global minimum
37	MIL, Bag-Net	MOE	Global minimum
38	MIL, Bag-Wrapper	MOE	Global minimum
39	MIL, Instance-Net	MOE	Global minimum
40	MIL, Non-aggregation (Instance-Wrapper)	MOE	Global minimum
41	MIL, Bag-AttentionNet	MOE	Mean
42	MIL, Bag-Net	MOE	Mean
43	MIL, Bag-Wrapper	MOE	Mean
44	MIL, Instance-Net	MOE	Mean
45	MIL, Non-aggregation (Instance-Wrapper)	MOE	Mean
46	MIL, Bag-AttentionNet	MOE	Non-aggregation
47	MIL, Bag-Net	MOE	Non-aggregation
48	MIL, Bag-Wrapper	MOE	Non-aggregation
49	MIL, Instance-Net	MOE	Non-aggregation
50	MIL, Non-aggregation (Instance-Wrapper)	MOE	Non-aggregation
51	MIL, Bag-AttentionNet	MOE	Random
52	MIL, Bag-Net	MOE	Random
53	MIL, Bag-Wrapper	MOE	Random
54	MIL, Instance-Net	MOE	Random
55	MIL, Non-aggregation (Instance-Wrapper)	MOE	Random
56	MIL, Bag-AttentionNet	3D-MoRSE	Boltzmann weight
57	MIL, Bag-Net	3D-MoRSE	Boltzmann weight
58	MIL, Bag-Wrapper	3D-MoRSE	Boltzmann weight
59	MIL, Instance-Net	3D-MoRSE	Boltzmann weight
60	MIL, Non-aggregation (Instance-Wrapper)	3D-MoRSE	Boltzmann weight
61	MIL, Bag-AttentionNet	3D-MoRSE	Global minimum
62	MIL, Bag-Net	3D-MoRSE	Global minimum
63	MIL, Bag-Wrapper	3D-MoRSE	Global minimum
64	MIL, Instance-Net	3D-MoRSE	Global minimum
65	MIL, Non-aggregation (Instance-Wrapper)	3D-MoRSE	Global minimum
66	MIL, Bag-AttentionNet	3D-MoRSE	Mean
67	MIL, Bag-Net	3D-MoRSE	Mean
68	MIL, Bag-Wrapper	3D-MoRSE	Mean
69	MIL, Instance-Net	3D-MoRSE	Mean

70	MIL, Non-aggregation (Instance-Wrapper)	3D-MoRSE	Mean
71	MIL, Bag-AttentionNet	3D-MoRSE	Non-aggregation
72	MIL, Bag-Net	3D-MoRSE	Non-aggregation
73	MIL, Bag-Wrapper	3D-MoRSE	Non-aggregation
74	MIL, Instance-Net	3D-MoRSE	Non-aggregation
75	MIL, Non-aggregation (Instance-Wrapper)	3D-MoRSE	Non-aggregation
76	MIL, Bag-AttentionNet	3D-MoRSE	Random
77	MIL, Bag-Net	3D-MoRSE	Random
78	MIL, Bag-Wrapper	3D-MoRSE	Random
79	MIL, Instance-Net	3D-MoRSE	Random
80	MIL, Non-aggregation (Instance-Wrapper)	3D-MoRSE	Random
81	MIL, Bag-AttentionNet	Pmapper	Boltzmann weight
82	MIL, Bag-Net	Pmapper	Boltzmann weight
83	MIL, Bag-Wrapper	Pmapper	Boltzmann weight
84	MIL, Instance-Net	Pmapper	Boltzmann weight
85	MIL, Non-aggregation (Instance-Wrapper)	Pmapper	Boltzmann weight
86	MIL, Bag-AttentionNet	Pmapper	Global minimum
87	MIL, Bag-Net	Pmapper	Global minimum
88	MIL, Bag-Wrapper	Pmapper	Global minimum
89	MIL, Instance-Net	Pmapper	Global minimum
90	MIL, Non-aggregation (Instance-Wrapper)	Pmapper	Global minimum
91	MIL, Bag-AttentionNet	Pmapper	Mean
92	MIL, Bag-Net	Pmapper	Mean
93	MIL, Bag-Wrapper	Pmapper	Mean
94	MIL, Instance-Net	Pmapper	Mean
95	MIL, Non-aggregation (Instance-Wrapper)	Pmapper	Mean
96	MIL, Bag-AttentionNet	Pmapper	Non-aggregation
97	MIL, Bag-Net	Pmapper	Non-aggregation
98	MIL, Bag-Wrapper	Pmapper	Non-aggregation
99	MIL, Instance-Net	Pmapper	Non-aggregation
100	MIL, Non-aggregation (Instance-Wrapper)	Pmapper	Non-aggregation
101	MIL, Bag-AttentionNet	Pmapper	Random
102	MIL, Bag-Net	Pmapper	Random
103	MIL, Bag-Wrapper	Pmapper	Random
104	MIL, Instance-Net	Pmapper	Random

105	MIL, Non-aggregation (Instance-Wrapper)	Pmapper	Random
106	RF	ECFP4 count	N/A
107	RF	MBTR	Boltzmann weight
108	RF	MBTR	Global minimum
109	RF	MBTR	Mean
110	RF	MBTR	Non-aggregation
111	RF	MBTR	Random
112	RF	MOE	Boltzmann weight
113	RF	MOE	Global minimum
114	RF	MOE	Mean
115	RF	MOE	Non-aggregation
116	RF	MOE	Random
117	RF	3D-MoRSE	Boltzmann weight
118	RF	3D-MoRSE	Global minimum
119	RF	3D-MoRSE	Mean
120	RF	3D-MoRSE	Non-aggregation
121	RF	3D-MoRSE	Random
122	RF	Pmapper	Boltzmann weight
123	RF	Pmapper	Global minimum
124	RF	Pmapper	Mean
125	RF	Pmapper	Non-aggregation
126	RF	Pmapper	Random
127	GEM	N/A	Global minimum
128	GEM	N/A	Non-aggregation
129	MolCLR	N/A	N/A
130	Uni-Mol	N/A	Global minimum
131	Uni-Mol	N/A	Non-aggregation
**APTC-1, APTC-2 data sets**			
1	Elastic Net	ECFP4 bit	N/A
2	Elastic Net	ECFP4 count	N/A
3	Elastic Net	2D PFP	N/A
4	Elastic Net	MBTR	Boltzmann weight
5	Elastic Net	MBTR	Global minimum
6	Elastic Net	MBTR	Mean
7	Elastic Net	MBTR	Non-aggregation

8	Elastic Net	MBTR	Random
9	Elastic Net	MOE	Boltzmann weight
10	Elastic Net	MOE	Global minimum
11	Elastic Net	MOE	Mean
12	Elastic Net	MOE	Non-aggregation
13	Elastic Net	MOE	Random
14	Elastic Net	3D-MoRSE	Boltzmann weight
15	Elastic Net	3D-MoRSE	Global minimum
16	Elastic Net	3D-MoRSE	Mean
17	Elastic Net	3D-MoRSE	Non-aggregation
18	Elastic Net	3D-MoRSE	Random
19	Elastic Net	Pmapper	Boltzmann weight
20	Elastic Net	Pmapper	Global minimum
21	Elastic Net	Pmapper	Mean
22	Elastic Net	Pmapper	Non-aggregation
23	Elastic Net	Pmapper	Random
24	MIL, Bag-AttentionNet	ECFP4 bit	N/A
25	MIL, Bag-Net	ECFP4 bit	N/A
26	MIL, Bag-Wrapper	ECFP4 bit	N/A
27	MIL, Instance-Net	ECFP4 bit	N/A
28	MIL, Non-aggregation (Instance-Wrapper)	ECFP4 bit	N/A
29	MIL, Bag-AttentionNet	ECFP4 count	N/A
30	MIL, Bag-Net	ECFP4 count	N/A
31	MIL, Bag-Wrapper	ECFP4 count	N/A
32	MIL, Instance-Net	ECFP4 count	N/A
33	MIL, Non-aggregation (Instance-Wrapper)	ECFP4 count	N/A
34	MIL, Bag-AttentionNet	2D PFP	N/A
35	MIL, Bag-Net	2D PFP	N/A
36	MIL, Bag-Wrapper	2D PFP	N/A
37	MIL, Instance-Net	2D PFP	N/A
38	MIL, Non-aggregation (Instance-Wrapper)	2D PFP	N/A
39	MIL, Bag-AttentionNet	MBTR	Boltzmann weight
40	MIL, Bag-Net	MBTR	Boltzmann weight
41	MIL, Bag-Wrapper	MBTR	Boltzmann weight
42	MIL, Instance-Net	MBTR	Boltzmann weight

43	MIL, Non-aggregation (Instance-Wrapper)	MBTR	Boltzmann weight
44	MIL, Bag-AttentionNet	MBTR	Global minimum
45	MIL, Bag-Net	MBTR	Global minimum
46	MIL, Bag-Wrapper	MBTR	Global minimum
47	MIL, Instance-Net	MBTR	Global minimum
48	MIL, Non-aggregation (Instance-Wrapper)	MBTR	Global minimum
49	MIL, Bag-AttentionNet	MBTR	Mean
50	MIL, Bag-Net	MBTR	Mean
51	MIL, Bag-Wrapper	MBTR	Mean
52	MIL, Instance-Net	MBTR	Mean
53	MIL, Non-aggregation (Instance-Wrapper)	MBTR	Mean
54	MIL, Bag-AttentionNet	MBTR	Non-aggregation
55	MIL, Bag-Net	MBTR	Non-aggregation
56	MIL, Bag-Wrapper	MBTR	Non-aggregation
57	MIL, Instance-Net	MBTR	Non-aggregation
58	MIL, Non-aggregation (Instance-Wrapper)	MBTR	Non-aggregation
59	MIL, Bag-AttentionNet	MBTR	Random
60	MIL, Bag-Net	MBTR	Random
61	MIL, Bag-Wrapper	MBTR	Random
62	MIL, Instance-Net	MBTR	Random
63	MIL, Non-aggregation (Instance-Wrapper)	MBTR	Random
64	MIL, Bag-AttentionNet	MOE	Boltzmann weight
65	MIL, Bag-Net	MOE	Boltzmann weight
66	MIL, Bag-Wrapper	MOE	Boltzmann weight
67	MIL, Instance-Net	MOE	Boltzmann weight
68	MIL, Non-aggregation (Instance-Wrapper)	MOE	Boltzmann weight
69	MIL, Bag-AttentionNet	MOE	Global minimum
70	MIL, Bag-Net	MOE	Global minimum
71	MIL, Bag-Wrapper	MOE	Global minimum
72	MIL, Instance-Net	MOE	Global minimum
73	MIL, Non-aggregation (Instance-Wrapper)	MOE	Global minimum
74	MIL, Bag-AttentionNet	MOE	Mean
75	MIL, Bag-Net	MOE	Mean
76	MIL, Bag-Wrapper	MOE	Mean
77	MIL, Instance-Net	MOE	Mean

78	MIL, Non-aggregation (Instance-Wrapper)	MOE	Mean
79	MIL, Bag-AttentionNet	MOE	Non-aggregation
80	MIL, Bag-Net	MOE	Non-aggregation
81	MIL, Bag-Wrapper	MOE	Non-aggregation
82	MIL, Instance-Net	MOE	Non-aggregation
83	MIL, Non-aggregation (Instance-Wrapper)	MOE	Non-aggregation
84	MIL, Bag-AttentionNet	MOE	Random
85	MIL, Bag-Net	MOE	Random
86	MIL, Bag-Wrapper	MOE	Random
87	MIL, Instance-Net	MOE	Random
88	MIL, Non-aggregation (Instance-Wrapper)	MOE	Random
89	MIL, Bag-AttentionNet	3D-MoRSE	Boltzmann weight
90	MIL, Bag-Net	3D-MoRSE	Boltzmann weight
91	MIL, Bag-Wrapper	3D-MoRSE	Boltzmann weight
92	MIL, Instance-Net	3D-MoRSE	Boltzmann weight
93	MIL, Non-aggregation (Instance-Wrapper)	3D-MoRSE	Boltzmann weight
94	MIL, Bag-AttentionNet	3D-MoRSE	Global minimum
95	MIL, Bag-Net	3D-MoRSE	Global minimum
96	MIL, Bag-Wrapper	3D-MoRSE	Global minimum
97	MIL, Instance-Net	3D-MoRSE	Global minimum
98	MIL, Non-aggregation (Instance-Wrapper)	3D-MoRSE	Global minimum
99	MIL, Bag-AttentionNet	3D-MoRSE	Mean
100	MIL, Bag-Net	3D-MoRSE	Mean
101	MIL, Bag-Wrapper	3D-MoRSE	Mean
102	MIL, Instance-Net	3D-MoRSE	Mean
103	MIL, Non-aggregation (Instance-Wrapper)	3D-MoRSE	Mean
104	MIL, Bag-AttentionNet	3D-MoRSE	Non-aggregation
105	MIL, Bag-Net	3D-MoRSE	Non-aggregation
106	MIL, Bag-Wrapper	3D-MoRSE	Non-aggregation
107	MIL, Instance-Net	3D-MoRSE	Non-aggregation
108	MIL, Non-aggregation (Instance-Wrapper)	3D-MoRSE	Non-aggregation
109	MIL, Bag-AttentionNet	3D-MoRSE	Random
110	MIL, Bag-Net	3D-MoRSE	Random
111	MIL, Bag-Wrapper	3D-MoRSE	Random
112	MIL, Instance-Net	3D-MoRSE	Random

113	MIL, Non-aggregation (Instance-Wrapper)	3D-MoRSE	Random
114	MIL, Bag-AttentionNet	Pmapper	Boltzmann weight
115	MIL, Bag-Net	Pmapper	Boltzmann weight
116	MIL, Bag-Wrapper	Pmapper	Boltzmann weight
117	MIL, Instance-Net	Pmapper	Boltzmann weight
118	MIL, Non-aggregation (Instance-Wrapper)	Pmapper	Boltzmann weight
119	MIL, Bag-AttentionNet	Pmapper	Global minimum
120	MIL, Bag-Net	Pmapper	Global minimum
121	MIL, Bag-Wrapper	Pmapper	Global minimum
122	MIL, Instance-Net	Pmapper	Global minimum
123	MIL, Non-aggregation (Instance-Wrapper)	Pmapper	Global minimum
124	MIL, Bag-AttentionNet	Pmapper	Mean
125	MIL, Bag-Net	Pmapper	Mean
126	MIL, Bag-Wrapper	Pmapper	Mean
127	MIL, Instance-Net	Pmapper	Mean
128	MIL, Non-aggregation (Instance-Wrapper)	Pmapper	Mean
129	MIL, Bag-AttentionNet	Pmapper	Non-aggregation
130	MIL, Bag-Net	Pmapper	Non-aggregation
131	MIL, Bag-Wrapper	Pmapper	Non-aggregation
132	MIL, Instance-Net	Pmapper	Non-aggregation
133	MIL, Non-aggregation (Instance-Wrapper)	Pmapper	Non-aggregation
134	MIL, Bag-AttentionNet	Pmapper	Random
135	MIL, Bag-Net	Pmapper	Random
136	MIL, Bag-Wrapper	Pmapper	Random
137	MIL, Instance-Net	Pmapper	Random
138	MIL, Non-aggregation (Instance-Wrapper)	Pmapper	Random
139	PLS	ECFP4 bit	N/A
140	PLS	ECFP4 count	N/A
141	PLS	2D PFP	N/A
142	PLS	MBTR	Boltzmann weight
143	PLS	MBTR	Global minimum
144	PLS	MBTR	Mean
145	PLS	MBTR	Non-aggregation
146	PLS	MBTR	Random
147	PLS	MOE	Boltzmann weight

148	PLS	MOE	Global minimum
149	PLS	MOE	Mean
150	PLS	MOE	Non-aggregation
151	PLS	MOE	Random
152	PLS	3D-MoRSE	Boltzmann weight
153	PLS	3D-MoRSE	Global minimum
154	PLS	3D-MoRSE	Mean
155	PLS	3D-MoRSE	Non-aggregation
156	PLS	3D-MoRSE	Random
157	PLS	Pmapper	Boltzmann weight
158	PLS	Pmapper	Global minimum
159	PLS	Pmapper	Mean
160	PLS	Pmapper	Non-aggregation
161	PLS	Pmapper	Random
162	RF	ECFP4 bit	N/A
163	RF	ECFP4 count	N/A
164	RF	2D PFP	N/A
165	RF	MBTR	Boltzmann weight
166	RF	MBTR	Global minimum
167	RF	MBTR	Mean
168	RF	MBTR	Non-aggregation
169	RF	MBTR	Random
170	RF	MOE	Boltzmann weight
171	RF	MOE	Global minimum
172	RF	MOE	Mean
173	RF	MOE	Non-aggregation
174	RF	MOE	Random
175	RF	3D-MoRSE	Boltzmann weight
176	RF	3D-MoRSE	Global minimum
177	RF	3D-MoRSE	Mean
178	RF	3D-MoRSE	Non-aggregation
179	RF	3D-MoRSE	Random
180	RF	Pmapper	Boltzmann weight
181	RF	Pmapper	Global minimum
182	RF	Pmapper	Mean

183	RF	Pmapper	Non-aggregation
184	RF	Pmapper	Random
185	SVM	ECFP4 bit	N/A
186	SVM	ECFP4 count	N/A
187	SVM	2D PFP	N/A
188	SVM	MBTR	Boltzmann weight
189	SVM	MBTR	Global minimum
190	SVM	MBTR	Mean
191	SVM	MBTR	Non-aggregation
192	SVM	MBTR	Random
193	SVM	MOE	Boltzmann weight
194	SVM	MOE	Global minimum
195	SVM	MOE	Mean
196	SVM	MOE	Non-aggregation
197	SVM	MOE	Random
198	SVM	3D-MoRSE	Boltzmann weight
199	SVM	3D-MoRSE	Global minimum
200	SVM	3D-MoRSE	Mean
201	SVM	3D-MoRSE	Non-aggregation
202	SVM	3D-MoRSE	Random
203	SVM	Pmapper	Boltzmann weight
204	SVM	Pmapper	Global minimum
205	SVM	Pmapper	Mean
206	SVM	Pmapper	Non-aggregation
207	SVM	Pmapper	Random
208	GEM	N/A	Global minimum
209	GEM	N/A	Non-aggregation
210	MolCLR	N/A	N/A
211	Uni-Mol	N/A	Global minimum
212	Uni-Mol	N/A	Non-aggregation

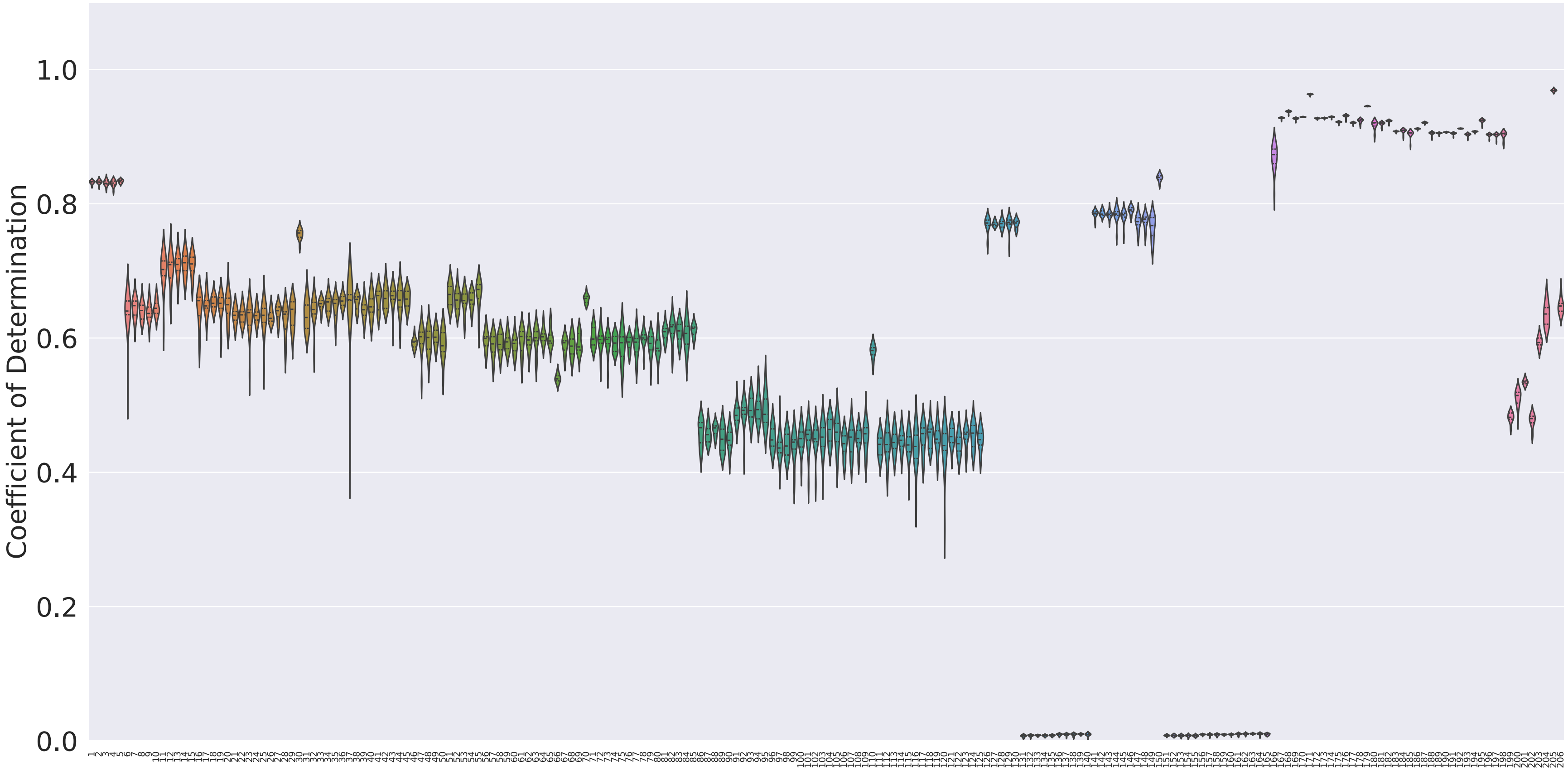

Figure S36. Violin plot of prediction accuracy (R^2) for 15 training sets of the PQC data set dipole moment prediction. The order of models is listed in Table S171.

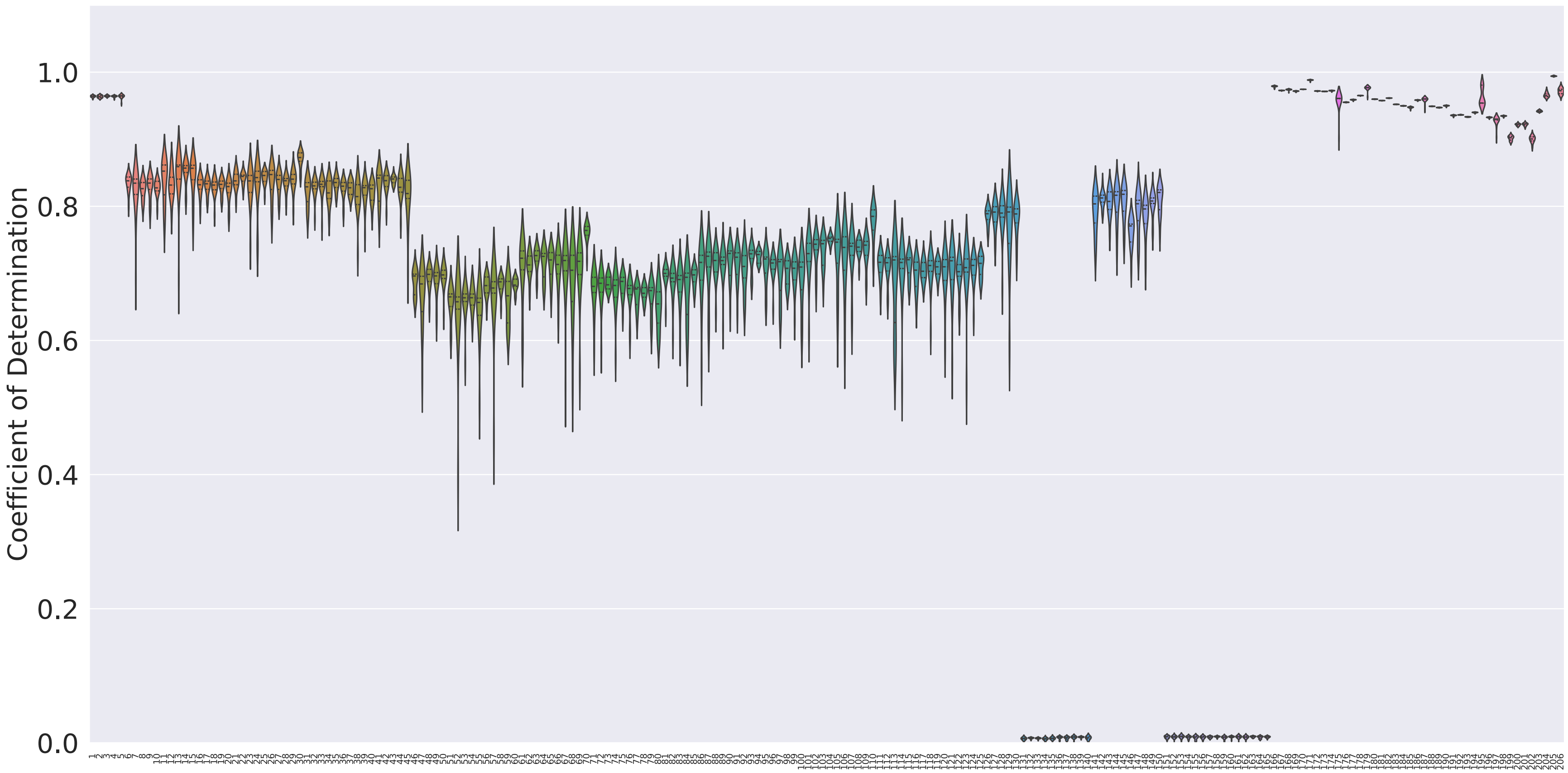

Figure S37. Violin plot of prediction accuracy (R^2) for 15 training sets of the PQC data set HOMO prediction. The order of models is listed in Table S171.

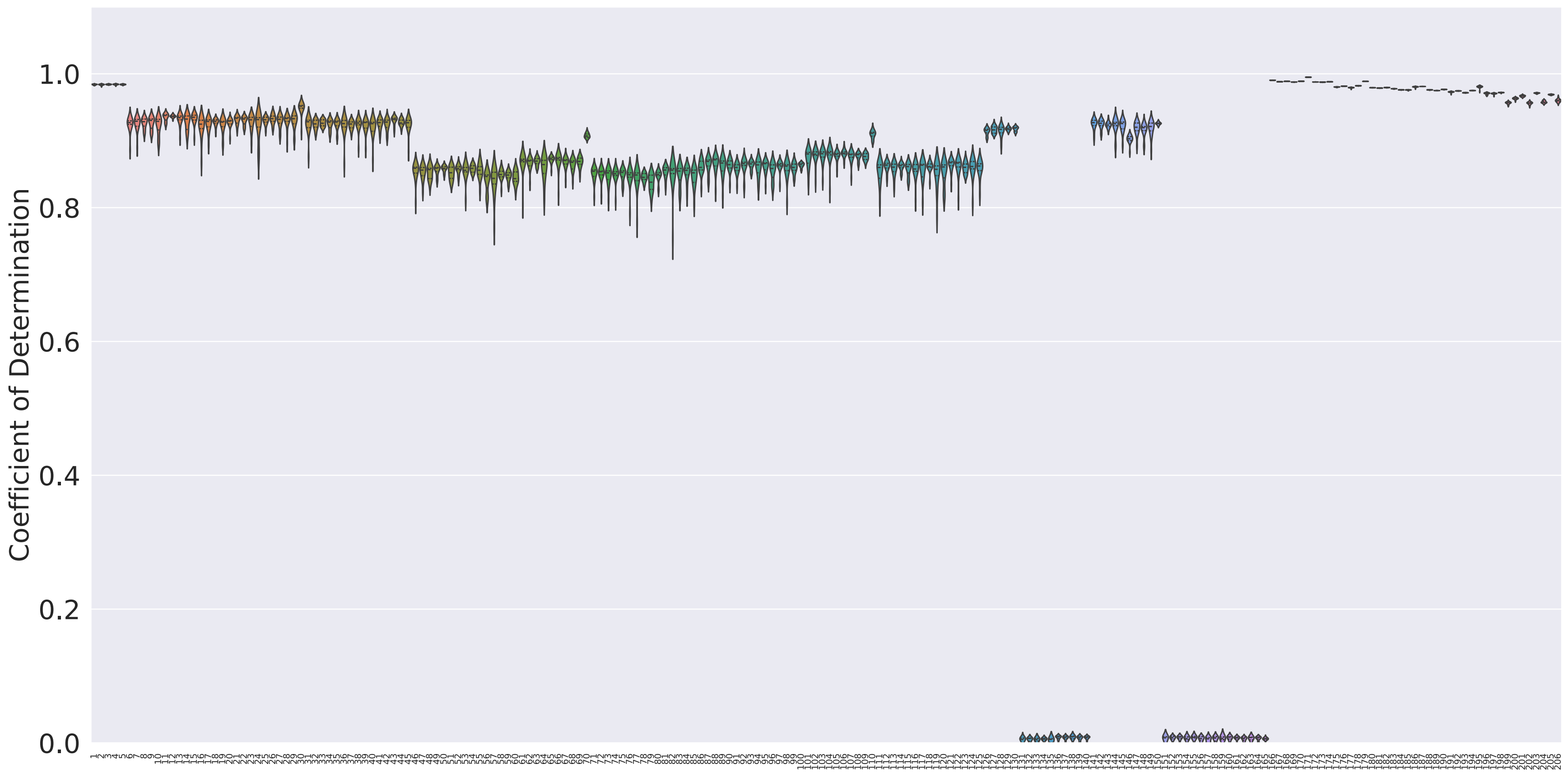

Figure S38. Violin plot of prediction accuracy (R^2) for 15 training sets of the PQC data set HOMO-LUMO gap prediction. The order of models is listed in Table S171.

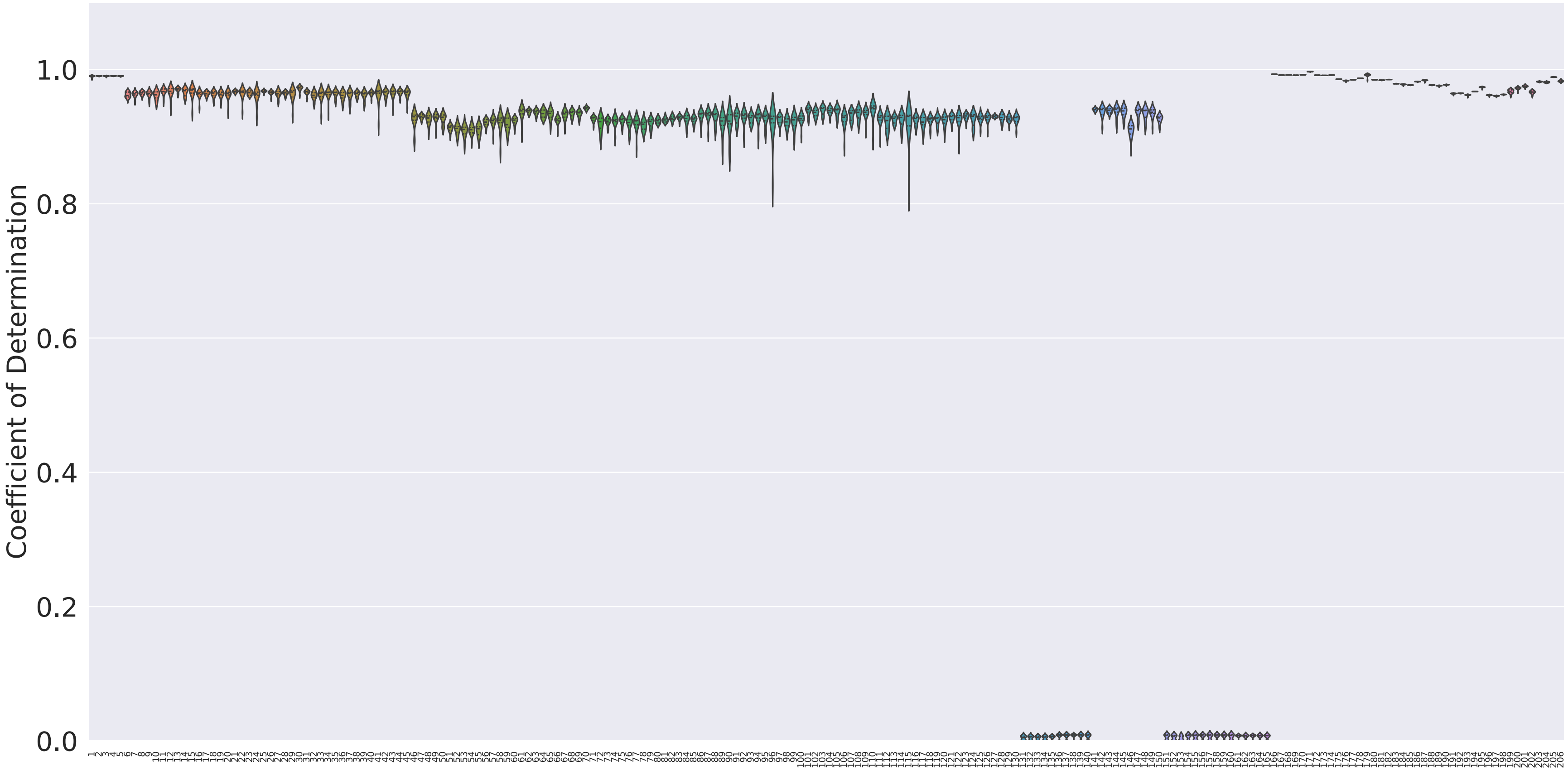

Figure S39. Violin plot of prediction accuracy (R^2) for 15 training sets of the PQC data set LUMO prediction. The order of models is listed in Table S171.

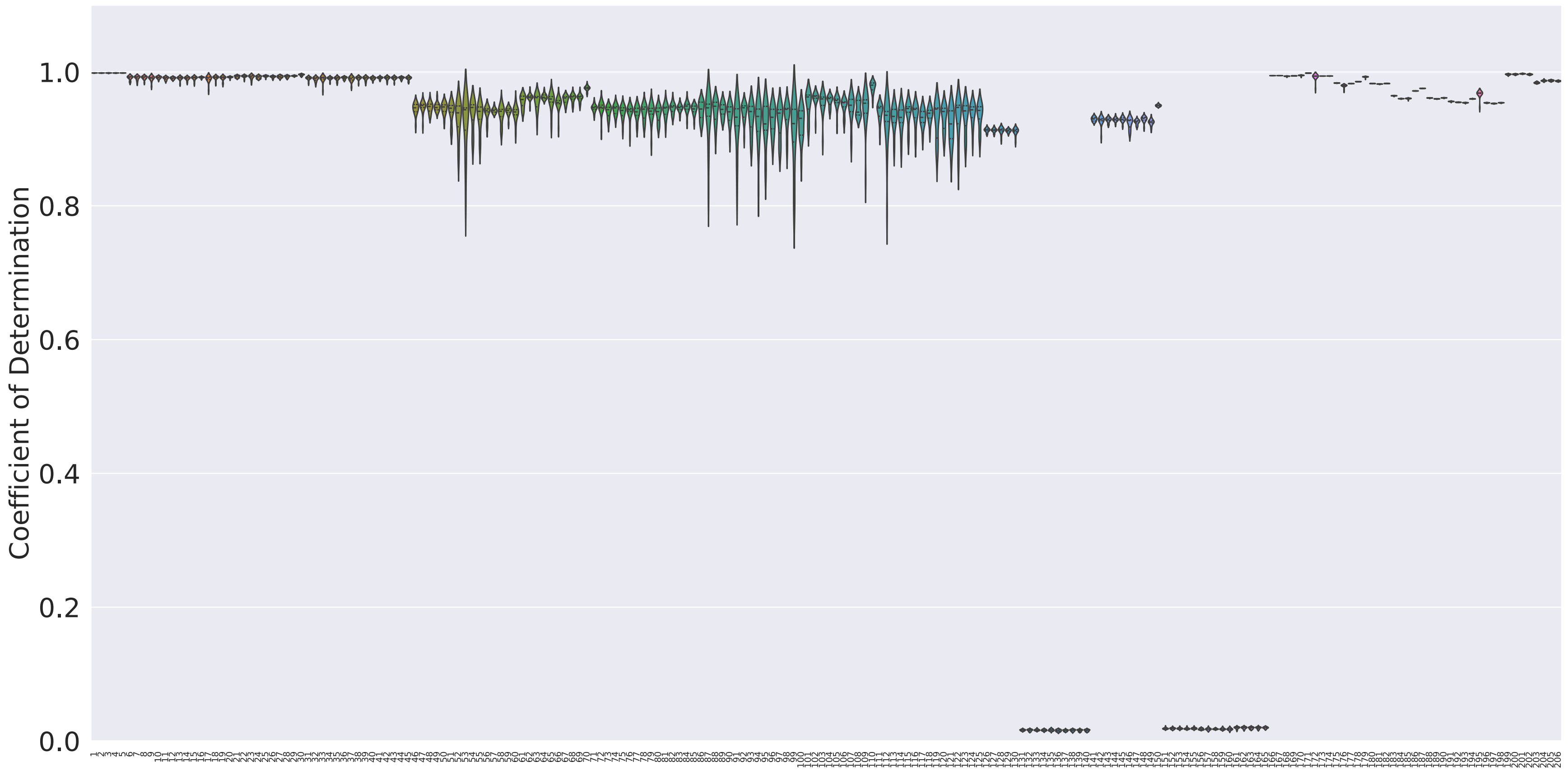

Figure S40. Violin plot of prediction accuracy (R^2) for 15 training sets of the PQC data set energy prediction. The order of models is listed in Table S171.

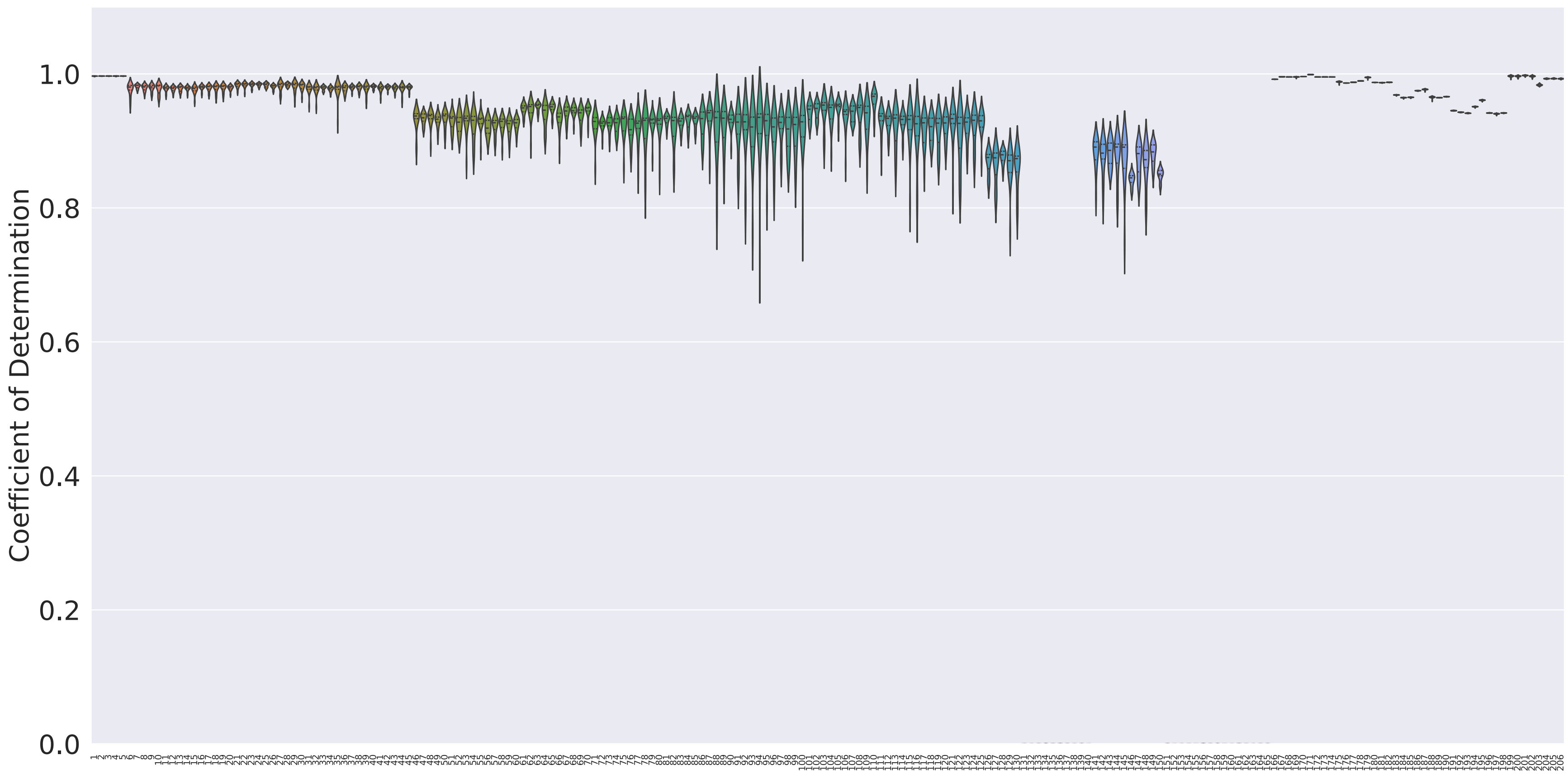

Figure S41. Violin plot of prediction accuracy (R^2) for 15 training sets of the PQC data set enthalpy prediction. The order of models is listed in Table S171.

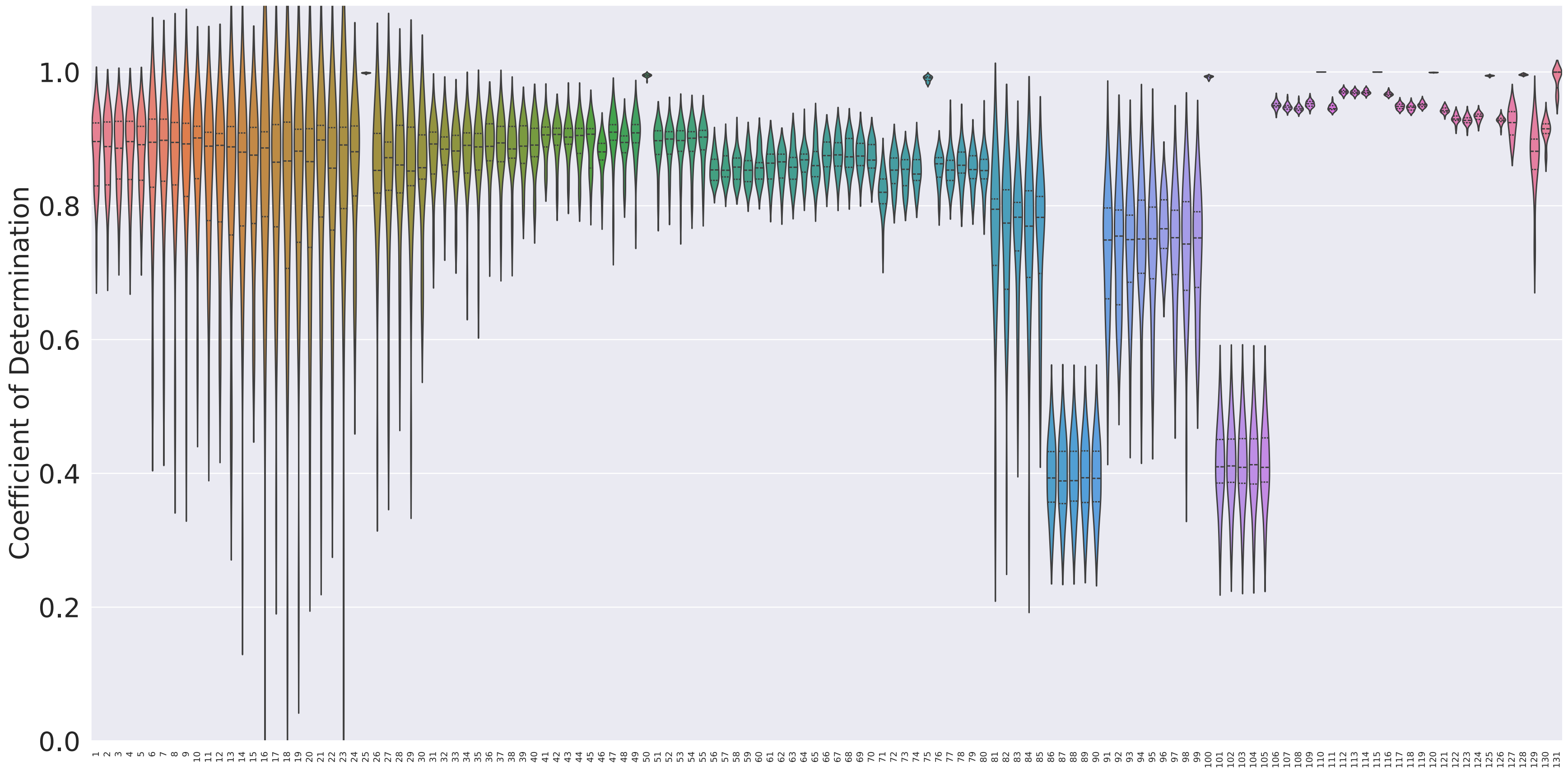

Figure S42. Violin plot of prediction accuracy (R^2) for 25 training sets of the MP data set property (melting point) prediction. The order of models is listed in Table S171.

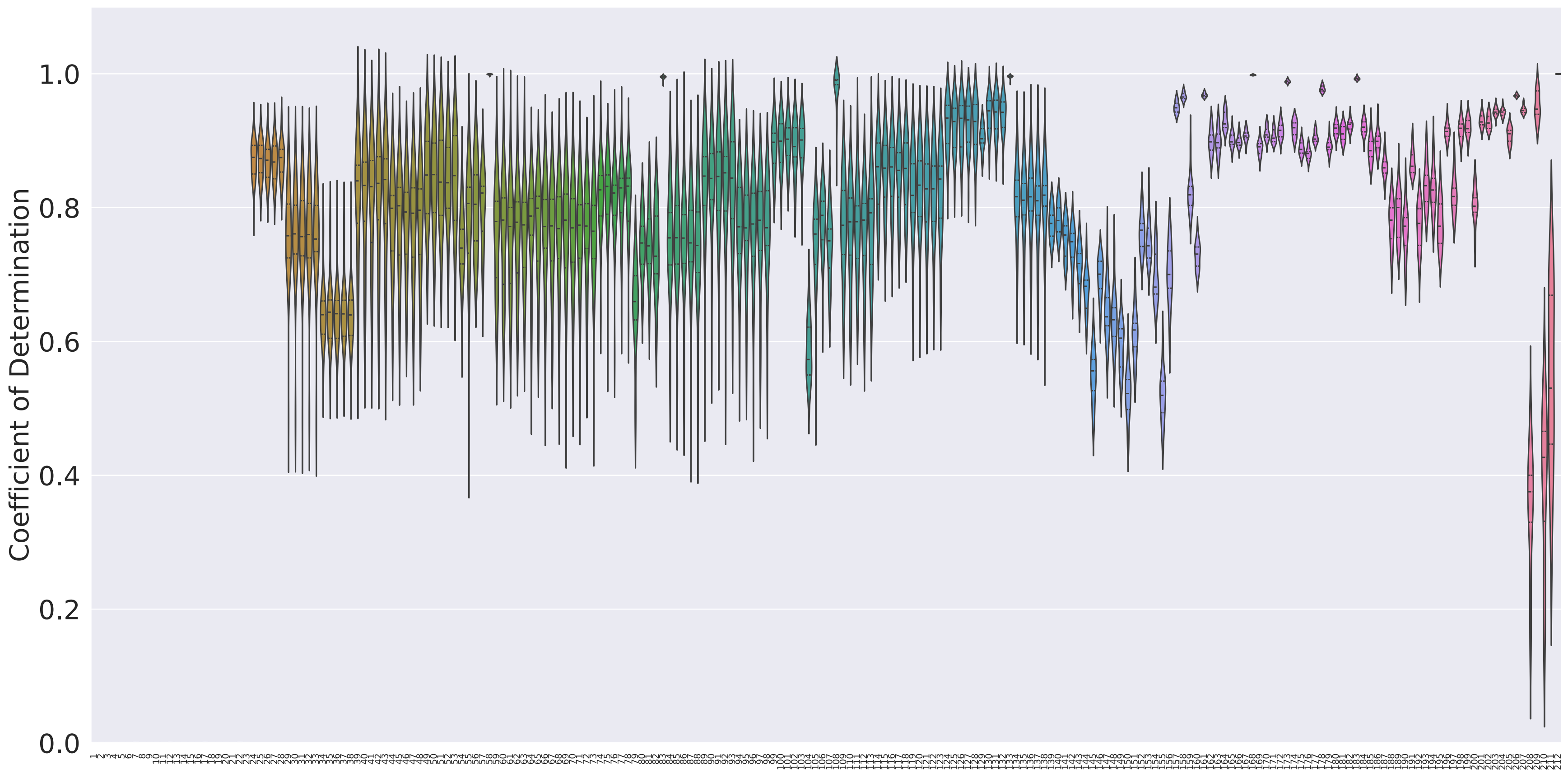

Figure S43. Violin plot of prediction accuracy (R^2) for 25 training sets of the APTC-1 data set property ($\Delta\Delta G^{\ddagger}$) prediction. The order of models is listed in Table S171.

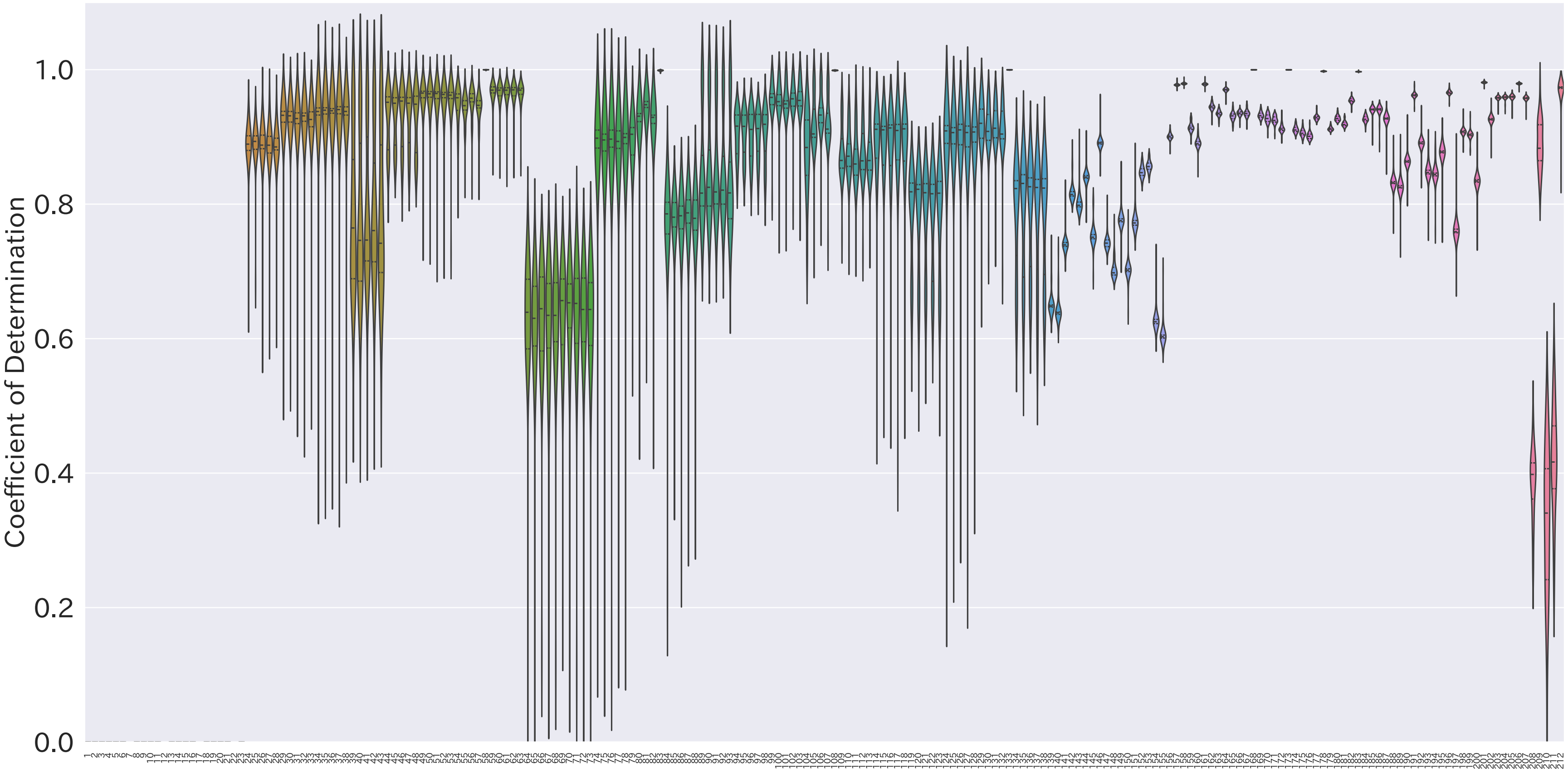

Figure S44. Violin plot of prediction accuracy (R^2) for 40 training sets of the APTC-2 data set property ($\Delta\Delta G^{\ddagger}$) prediction. The order of models is listed in Table S171.

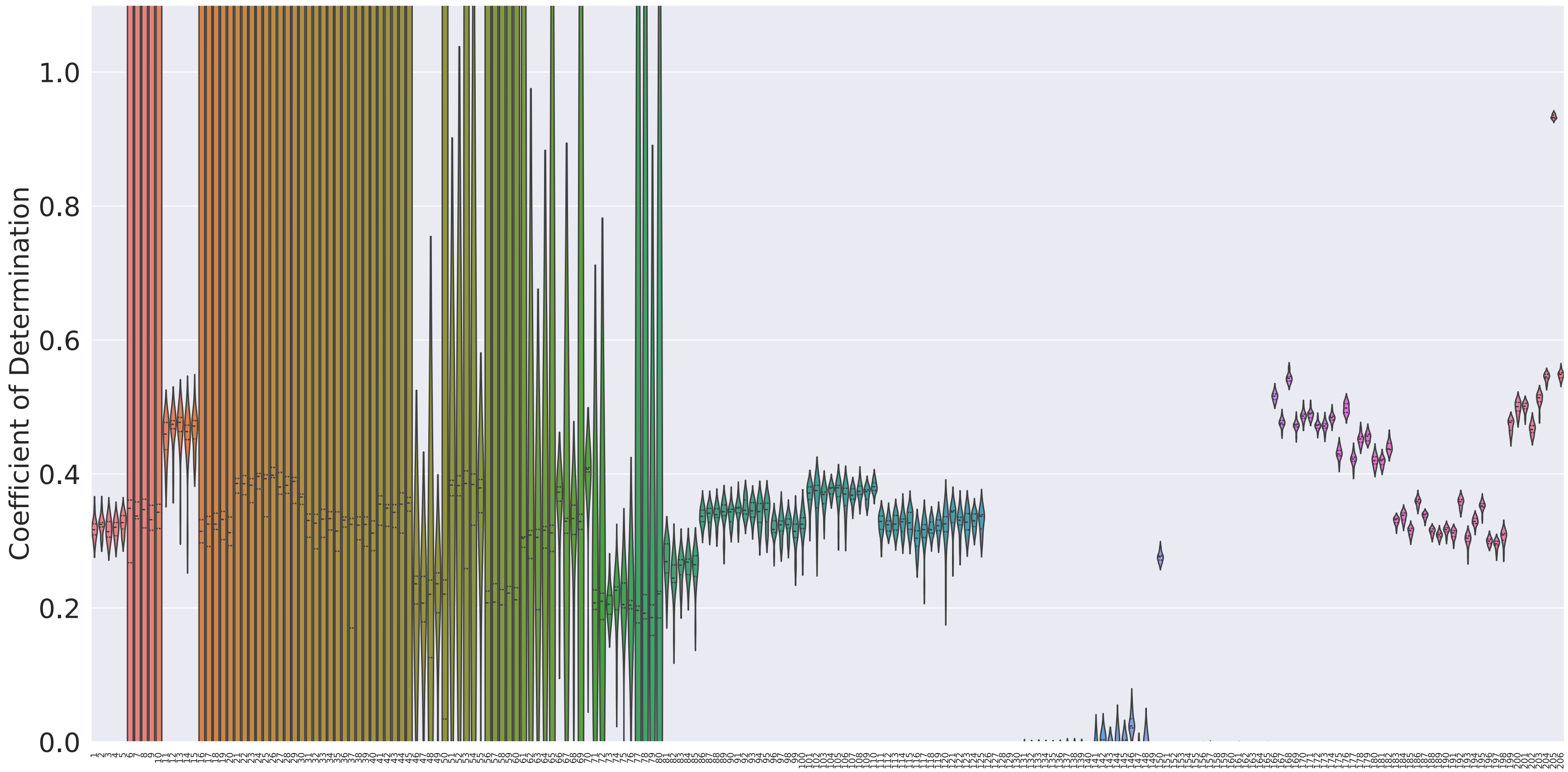

Figure S45. Violin plot of prediction accuracy (R^2) for 15 test sets of the PQC data set dipole moment prediction. The order of models is listed in Table S171.

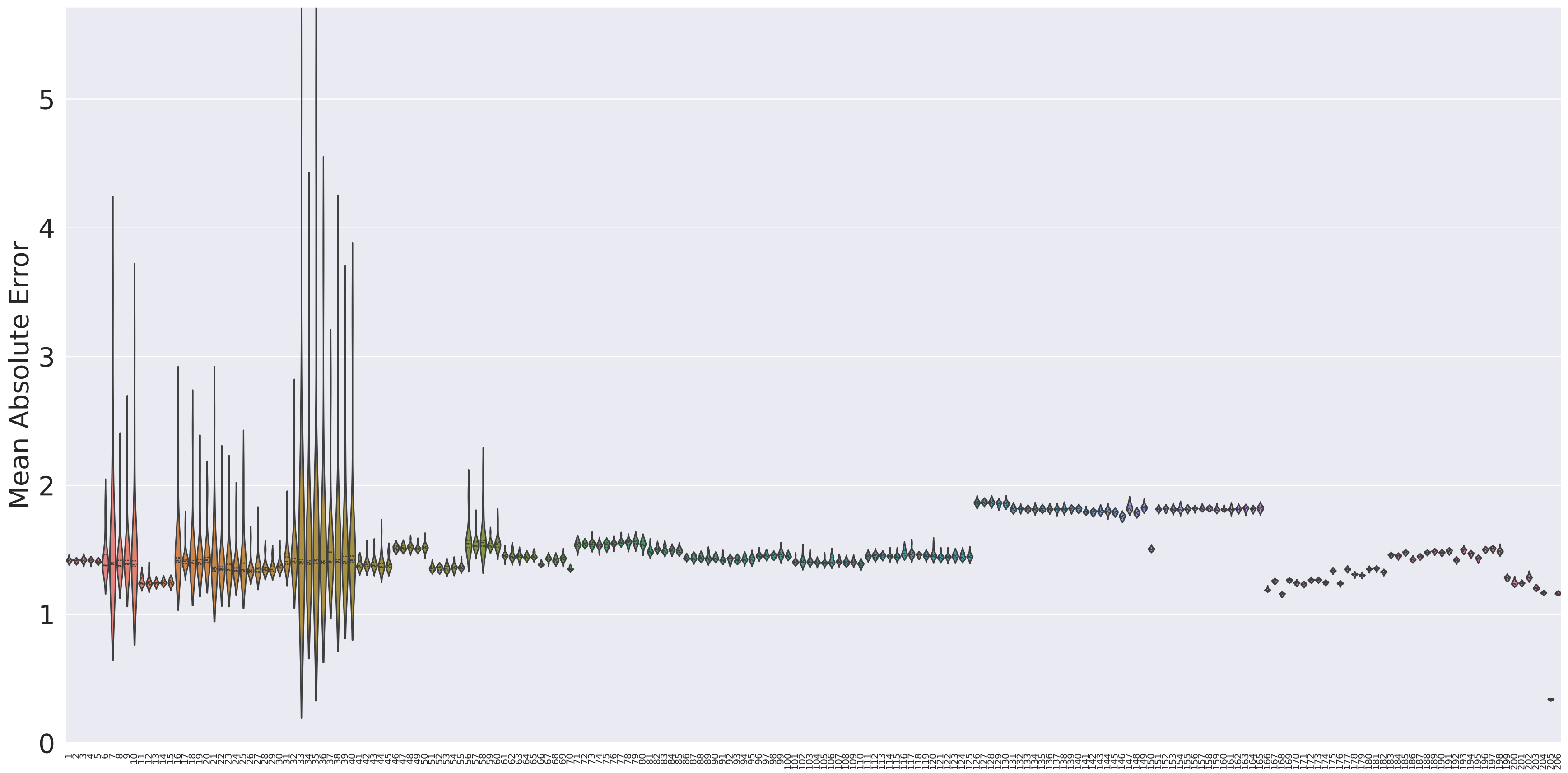

Figure S46. Violin plot of prediction accuracy (MAE) for 15 test sets of the PQC data set dipole moment prediction. The order of models is listed in Table S171.

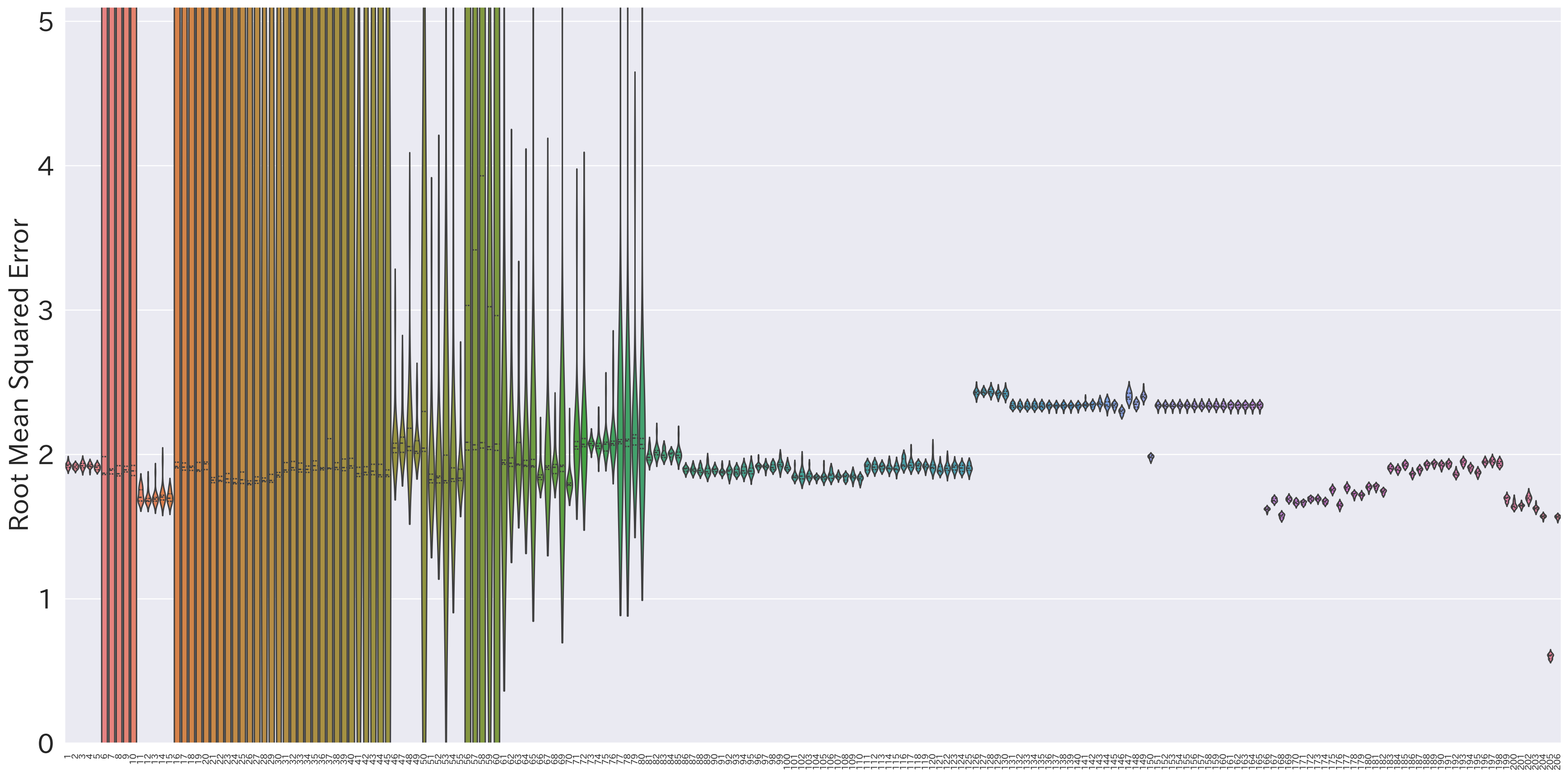

Figure S47. Violin plot of prediction accuracy (RMSE) for 15 test sets of the PQC data set dipole moment prediction. The order of models is listed in Table S171.

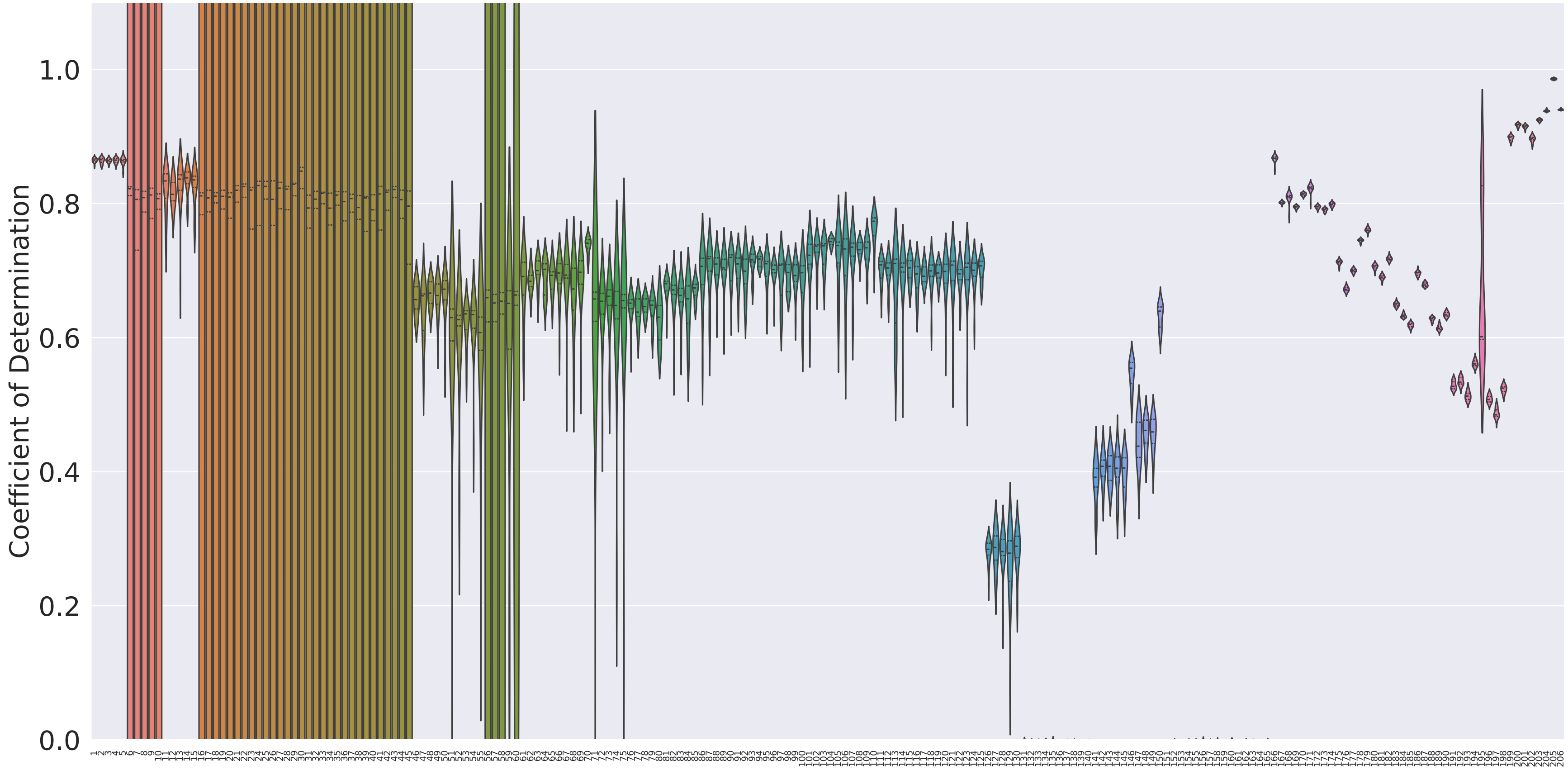

Figure S48. Violin plot of prediction accuracy (R^2) for 15 test sets of the PQC data set HOMO prediction. The order of models is listed in Table S171.

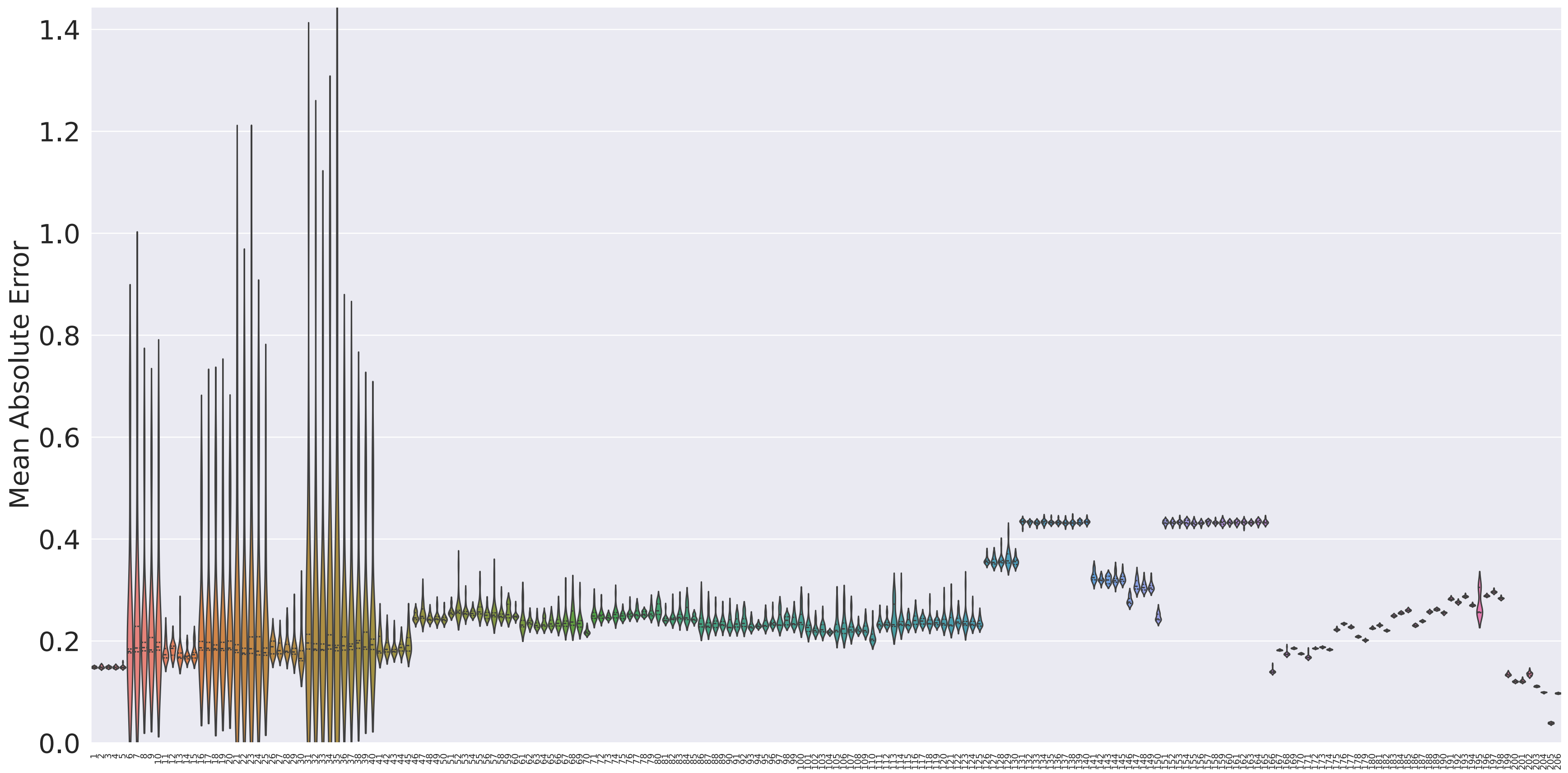

Figure S49. Violin plot of prediction accuracy (MAE) for 15 test sets of the PQC data set HOMO prediction. The order of models is listed in Table S171.

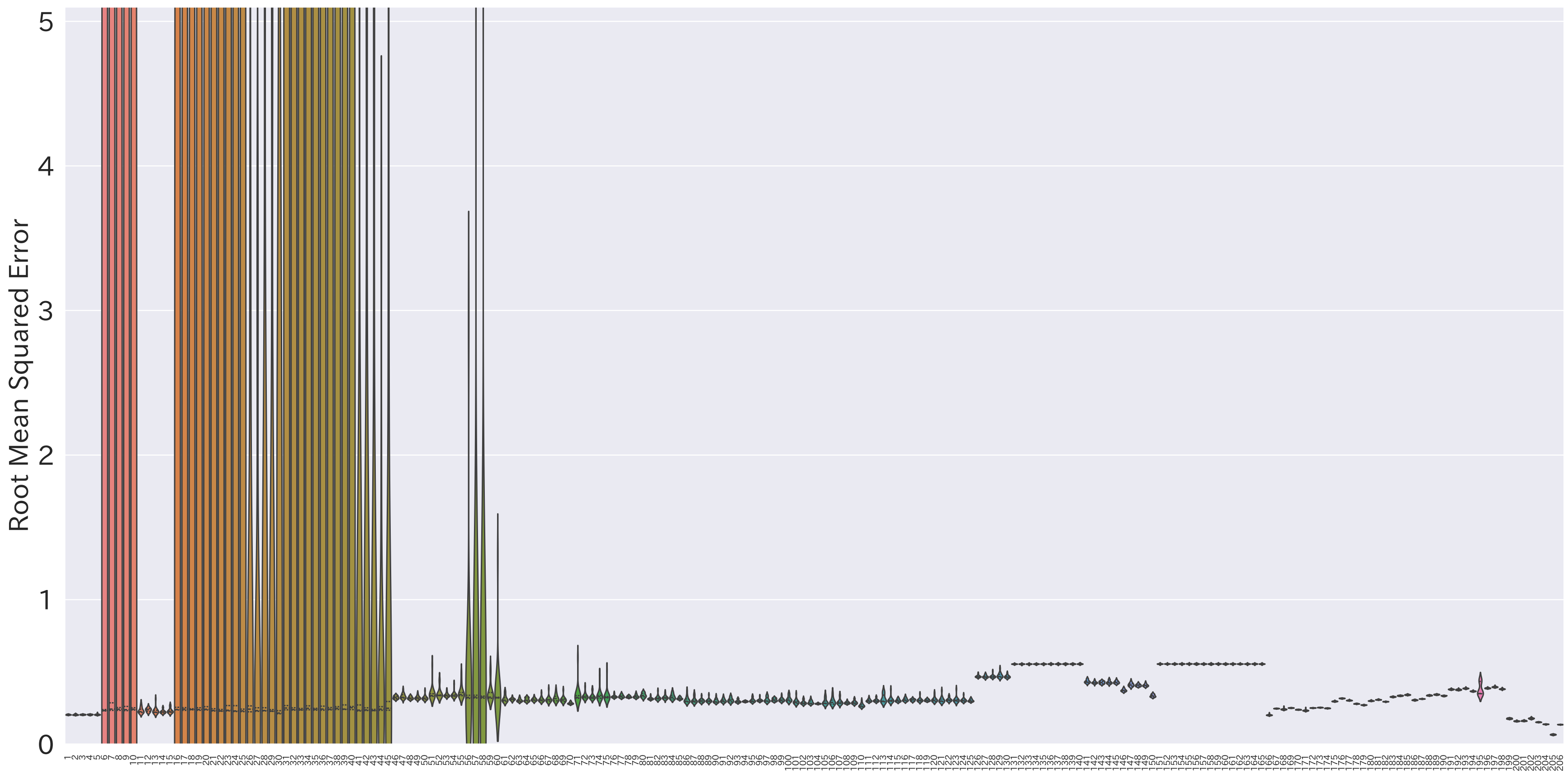

Figure S50. Violin plot of prediction accuracy (RMSE) for 15 test sets of the PQC data set HOMO prediction. The order of models is listed in Table S171.

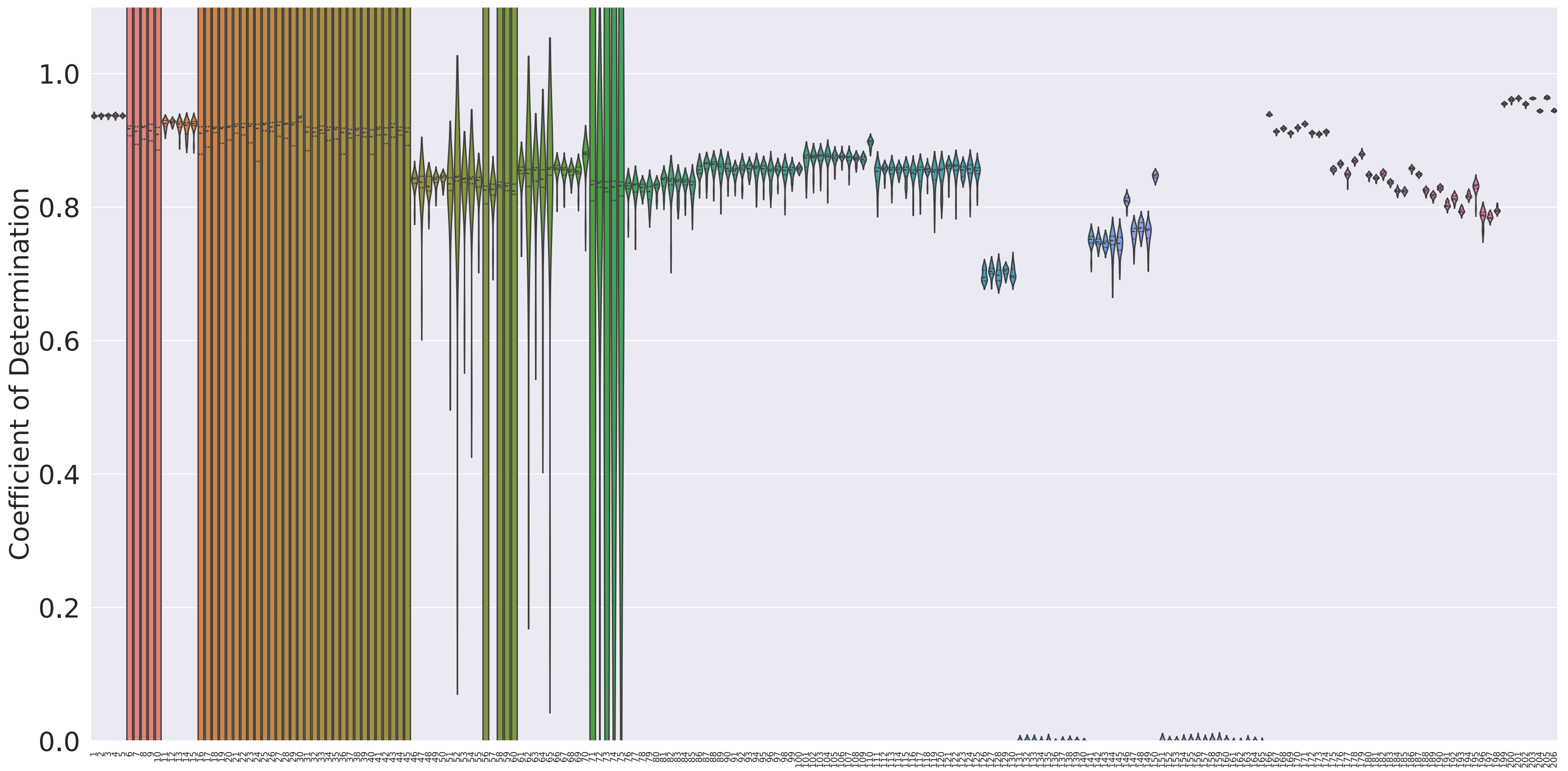

Figure S51. Violin plot of prediction accuracy (R^2) for 15 test sets of the PQC data set HOMO-LUMO gap prediction. The order of models is listed in Table S171.

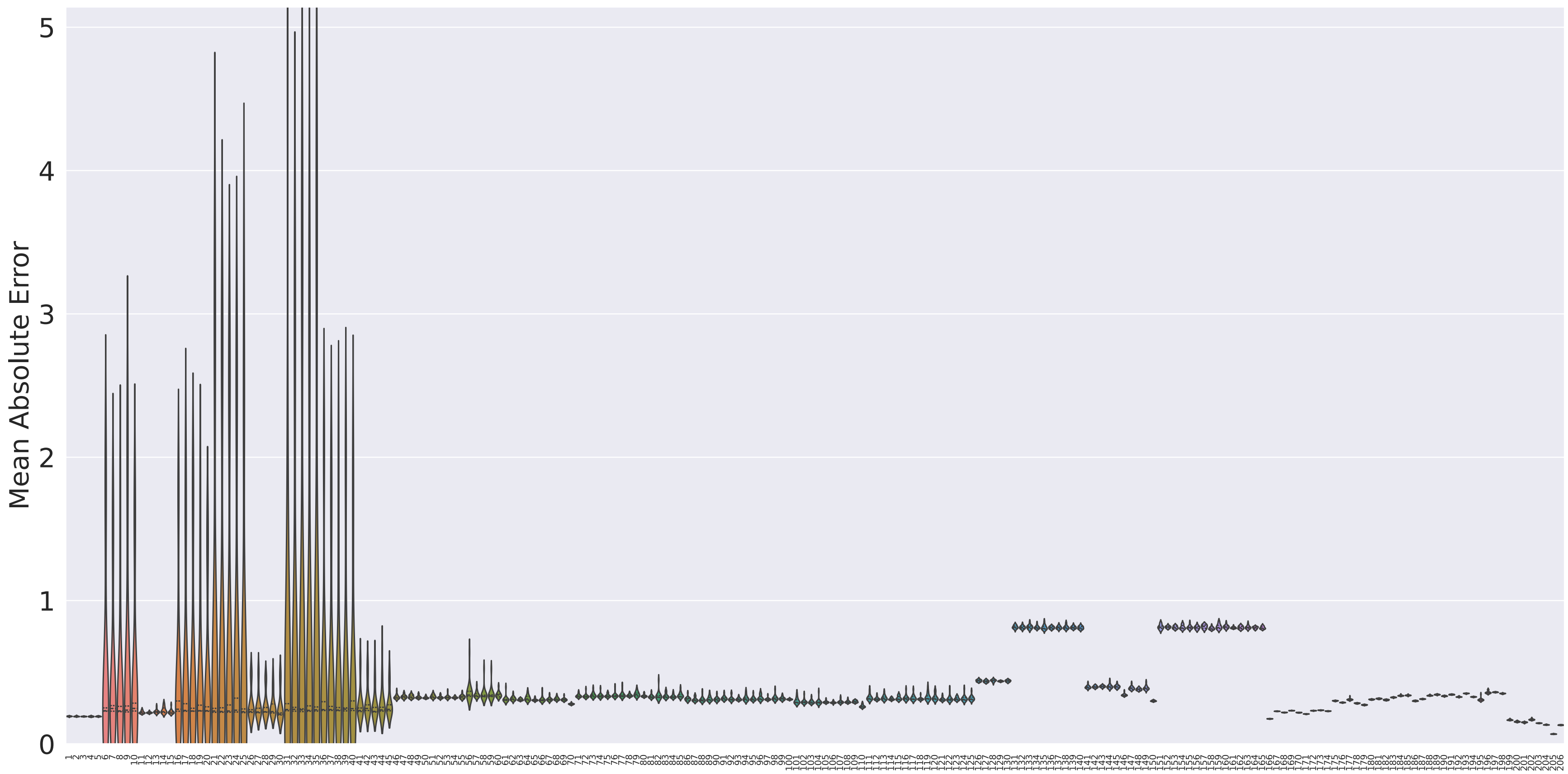

Figure S52. Violin plot of prediction accuracy (MAE) for 15 test sets of the PQC data set HOMO-LUMO gap prediction. The order of models is listed in Table S171.

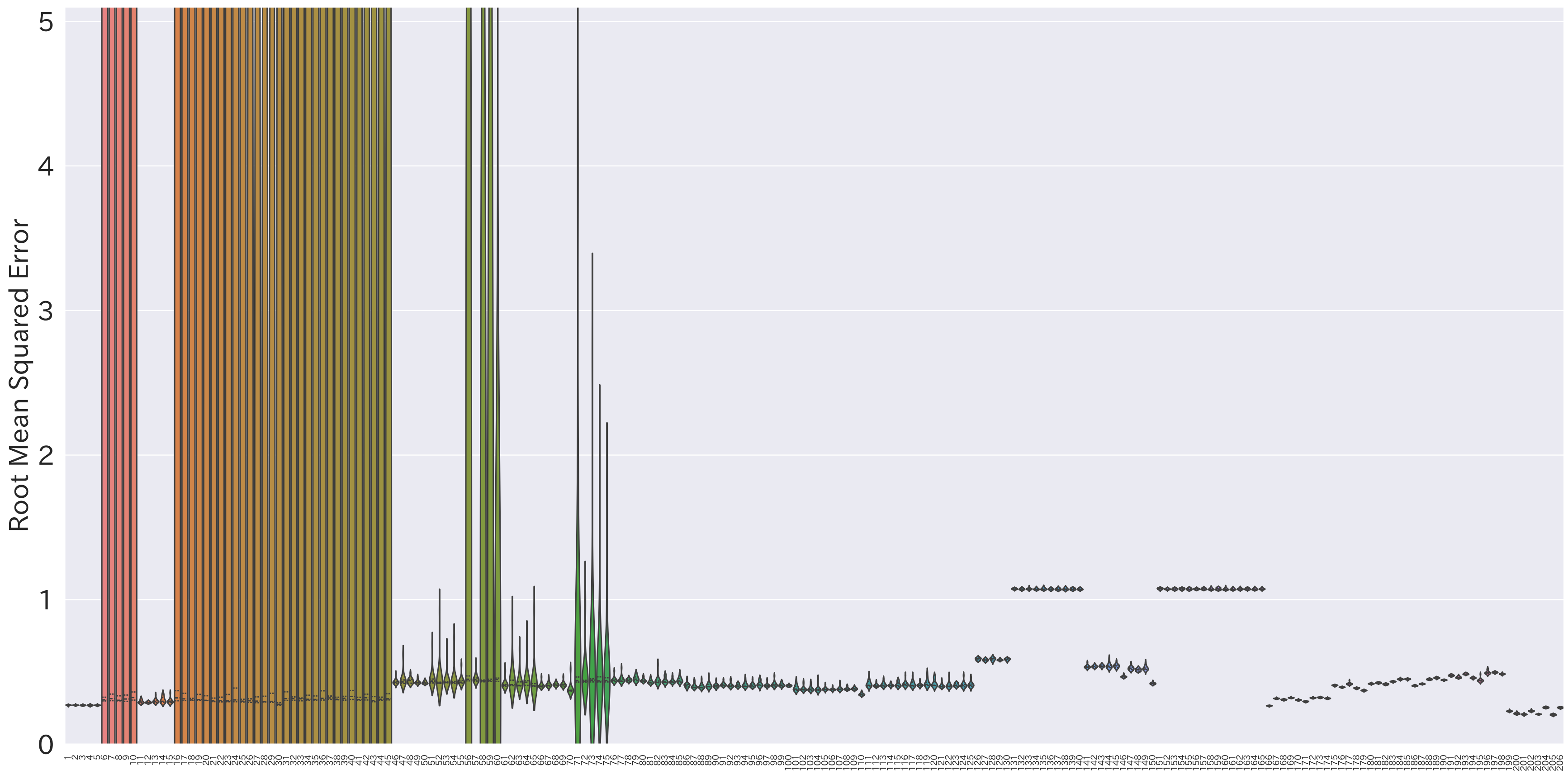

Figure S53. Violin plot of prediction accuracy (RMSE) for 15 test sets of the PQC data set HOMO-LUMO gap prediction. The order of models is listed in Table S171.

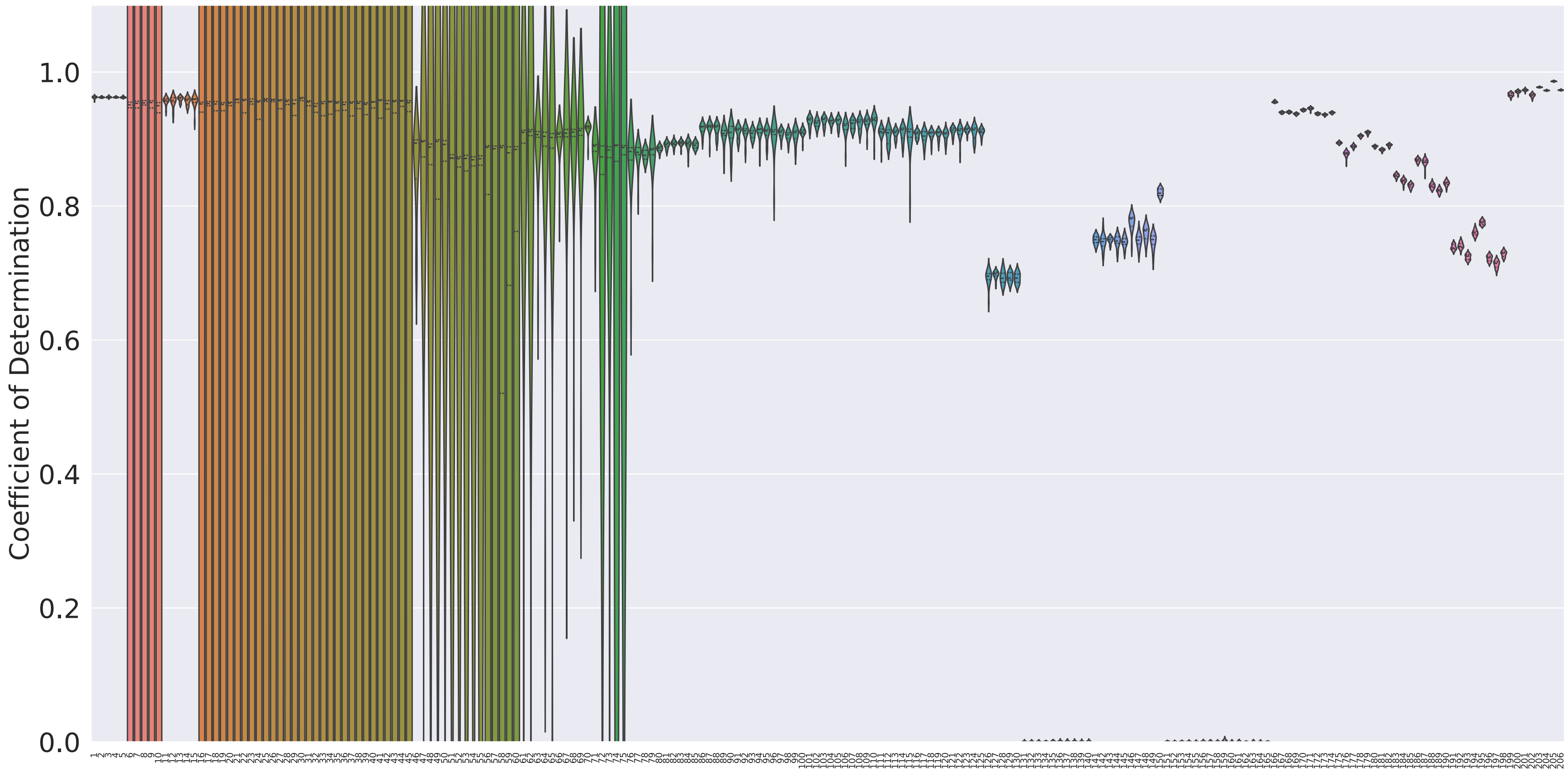

Figure S54. Violin plot of prediction accuracy (R^2) for 15 test sets of the PQC data set LUMO prediction. The order of models is listed in Table S171.

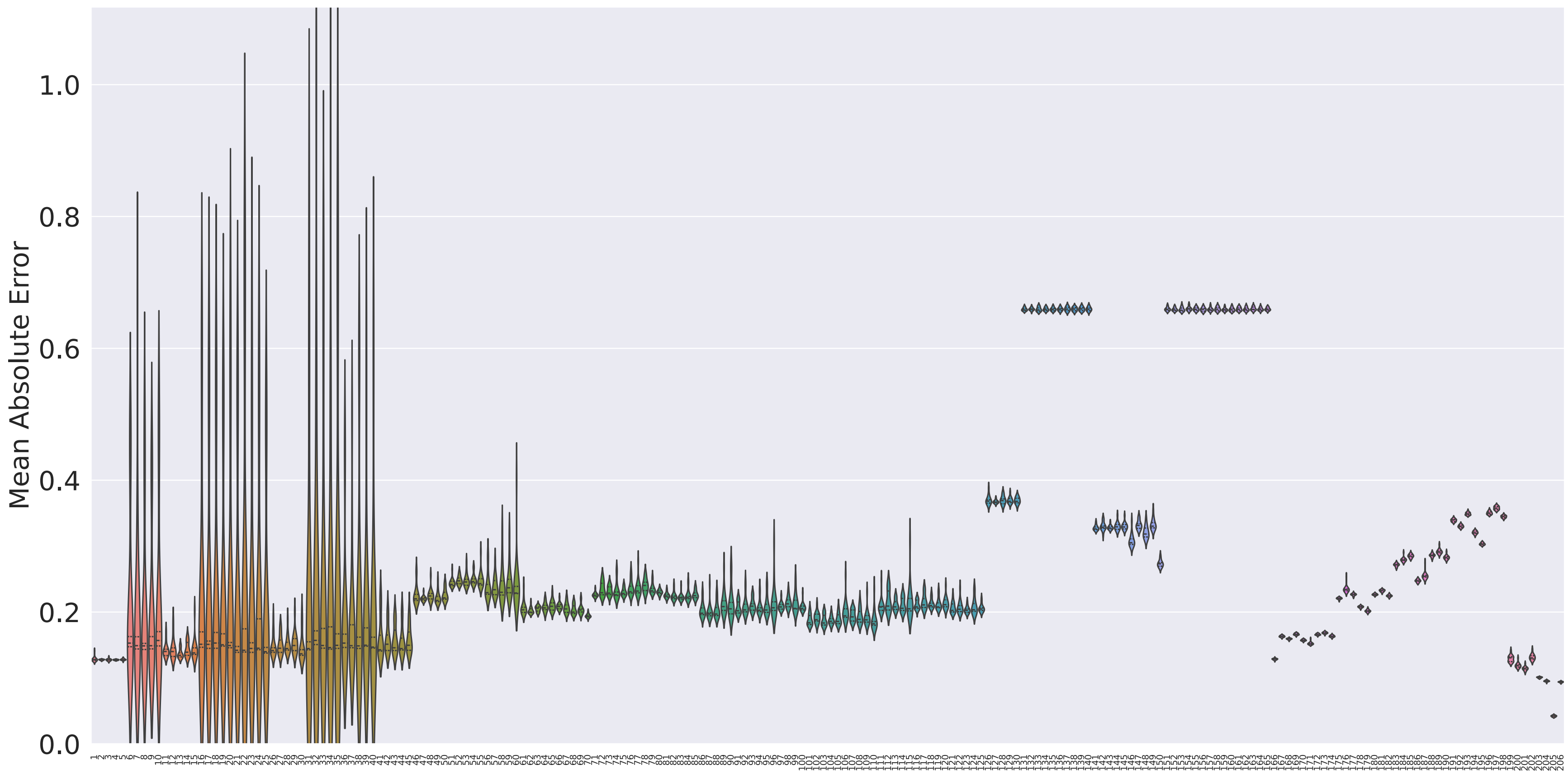

Figure S55. Violin plot of prediction accuracy (MAE) for 15 test sets of the PQC data set LUMO prediction. The order of models is listed in Table S171.

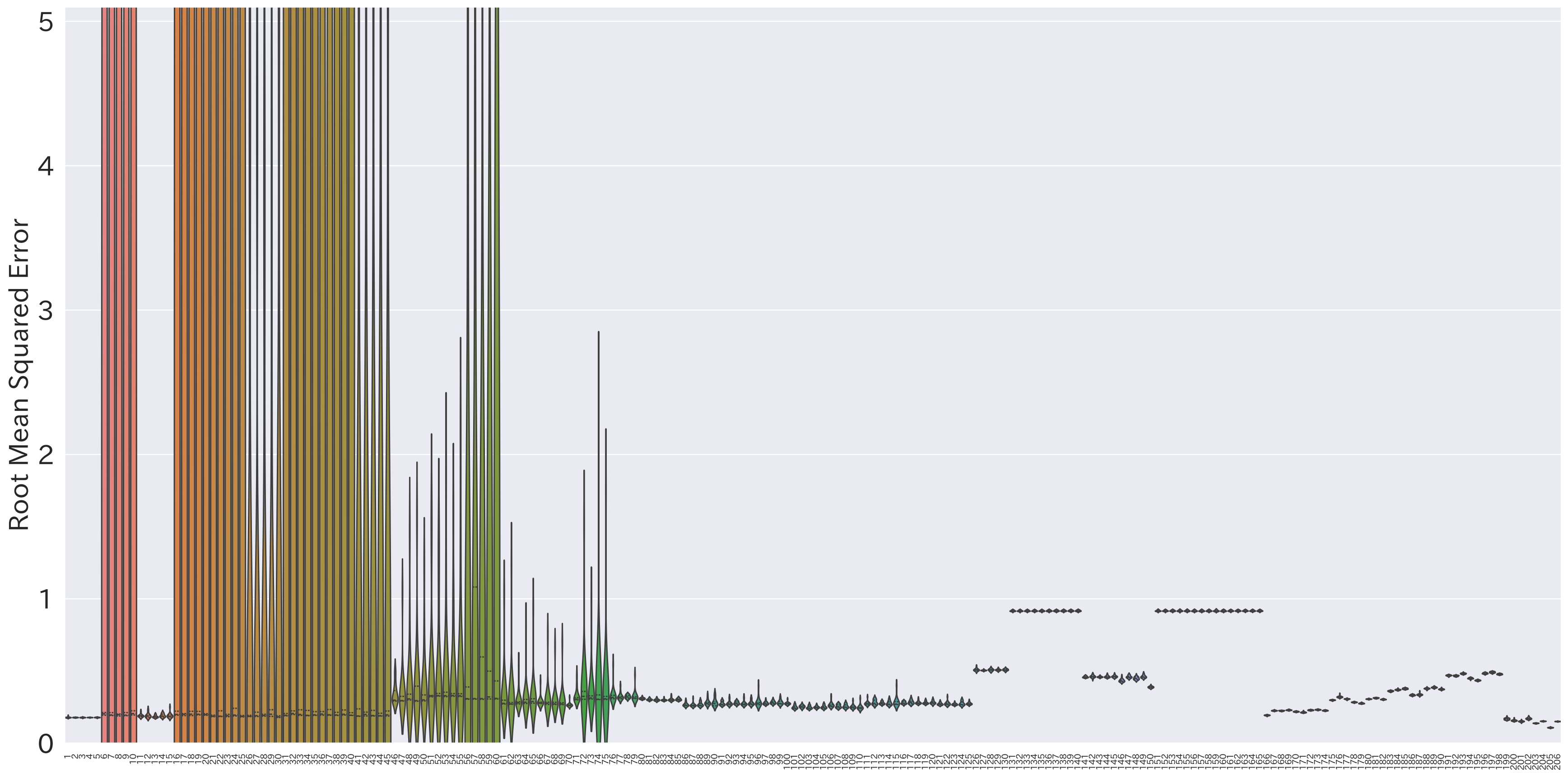

Figure S56. Violin plot of prediction accuracy (RMSE) for 15 test sets of the PQC data set LUMO prediction. The order of models is listed in Table S171.

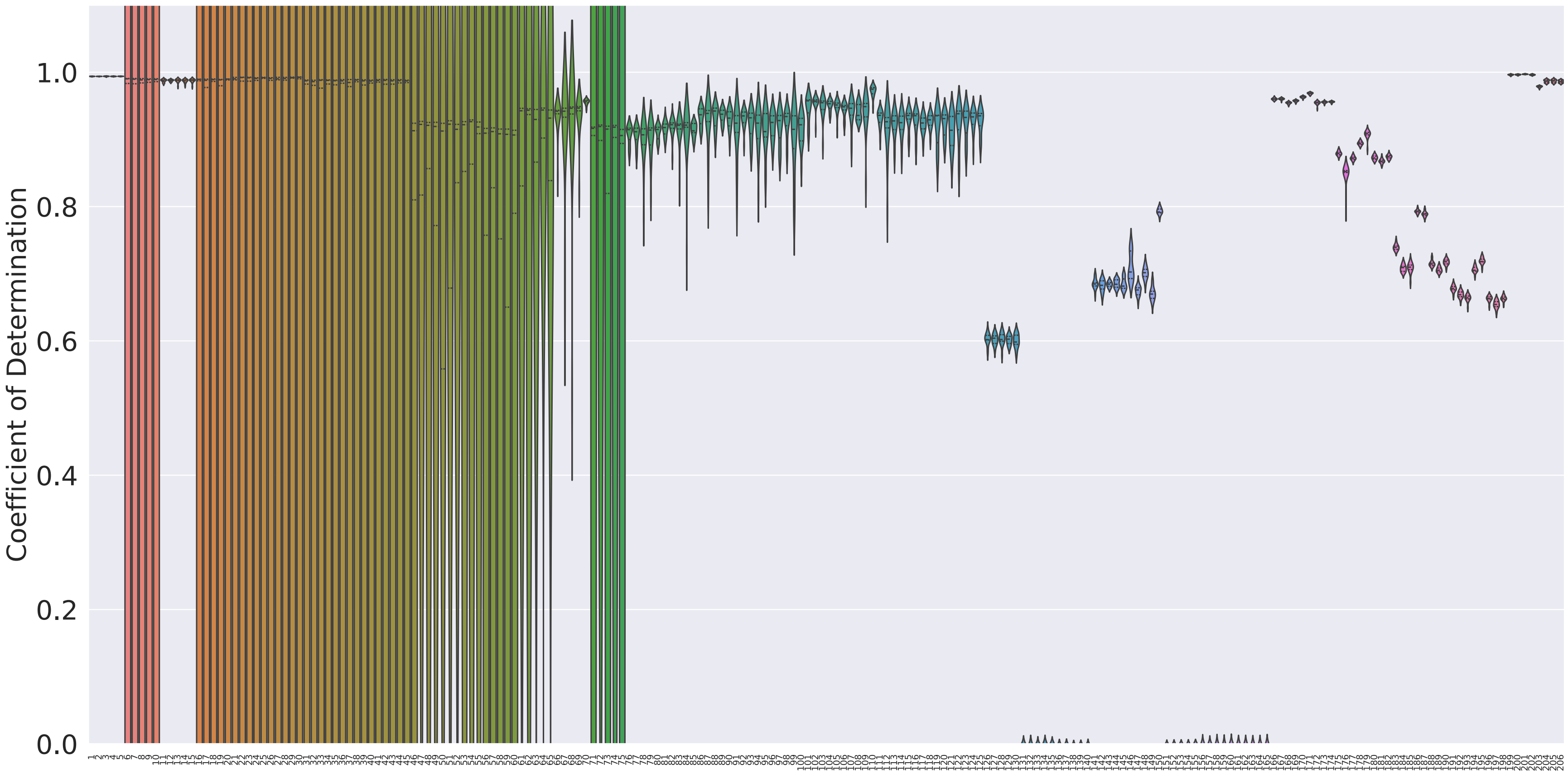

Figure S57. Violin plot of prediction accuracy (R^2) for 15 test sets of the PQC data set energy prediction. The order of models is listed in Table S171.

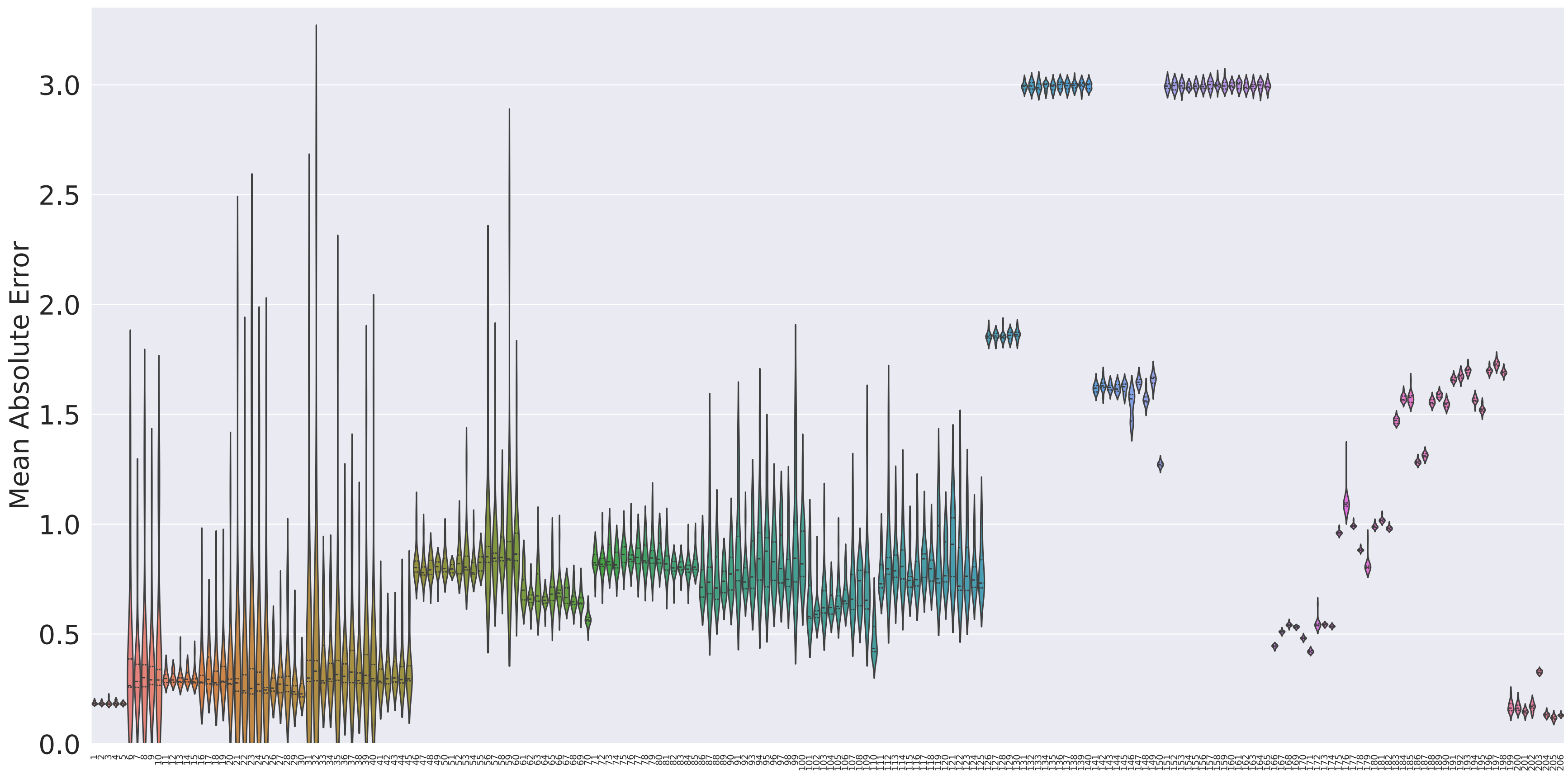

Figure S58. Violin plot of prediction accuracy (MAE) for 15 test sets of the PQC data set energy prediction. The order of models is listed in Table S171.

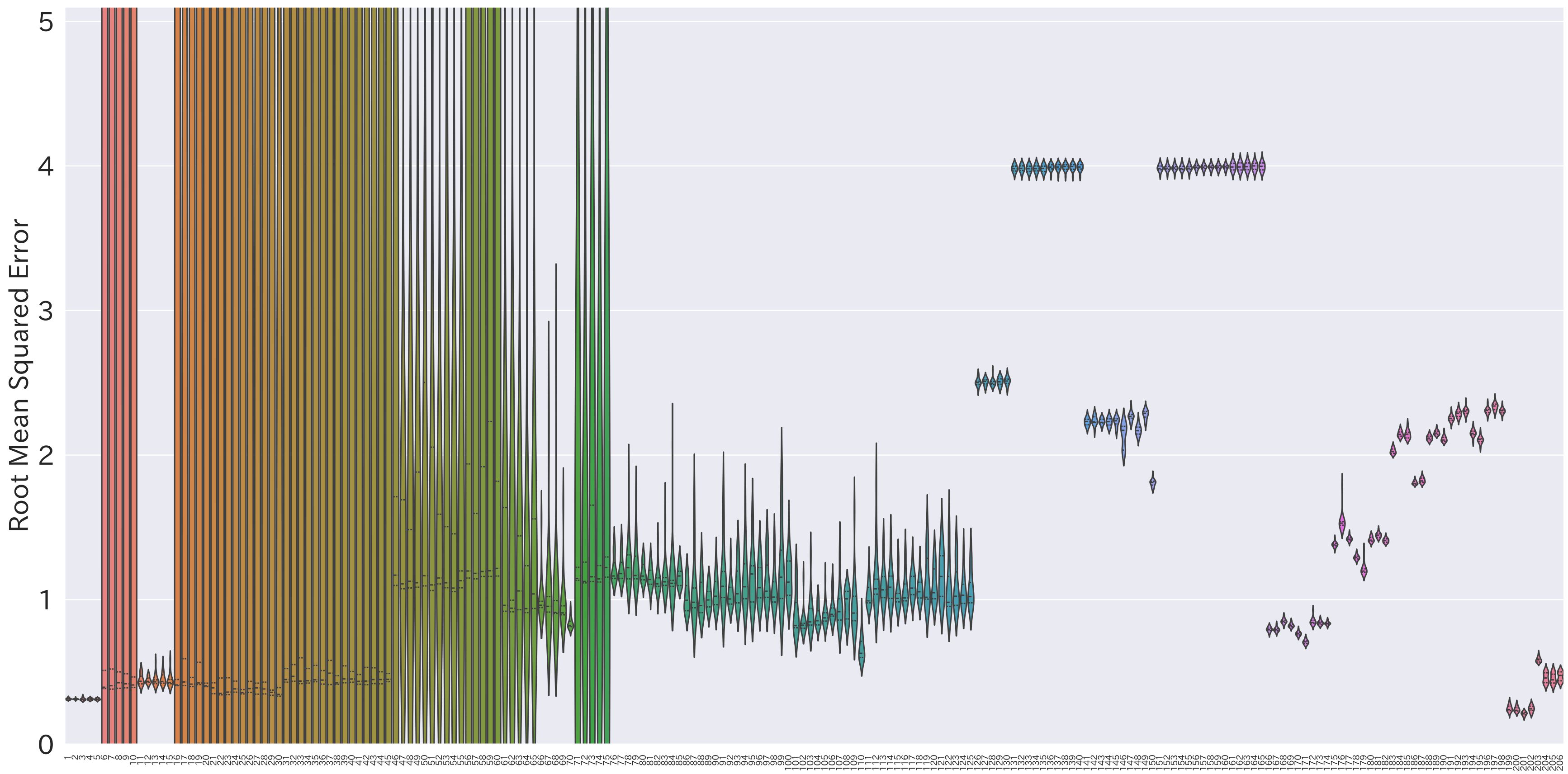

Figure S59. Violin plot of prediction accuracy (RMSE) for 15 test sets of the PQC data set energy prediction. The order of models is listed in Table S171.

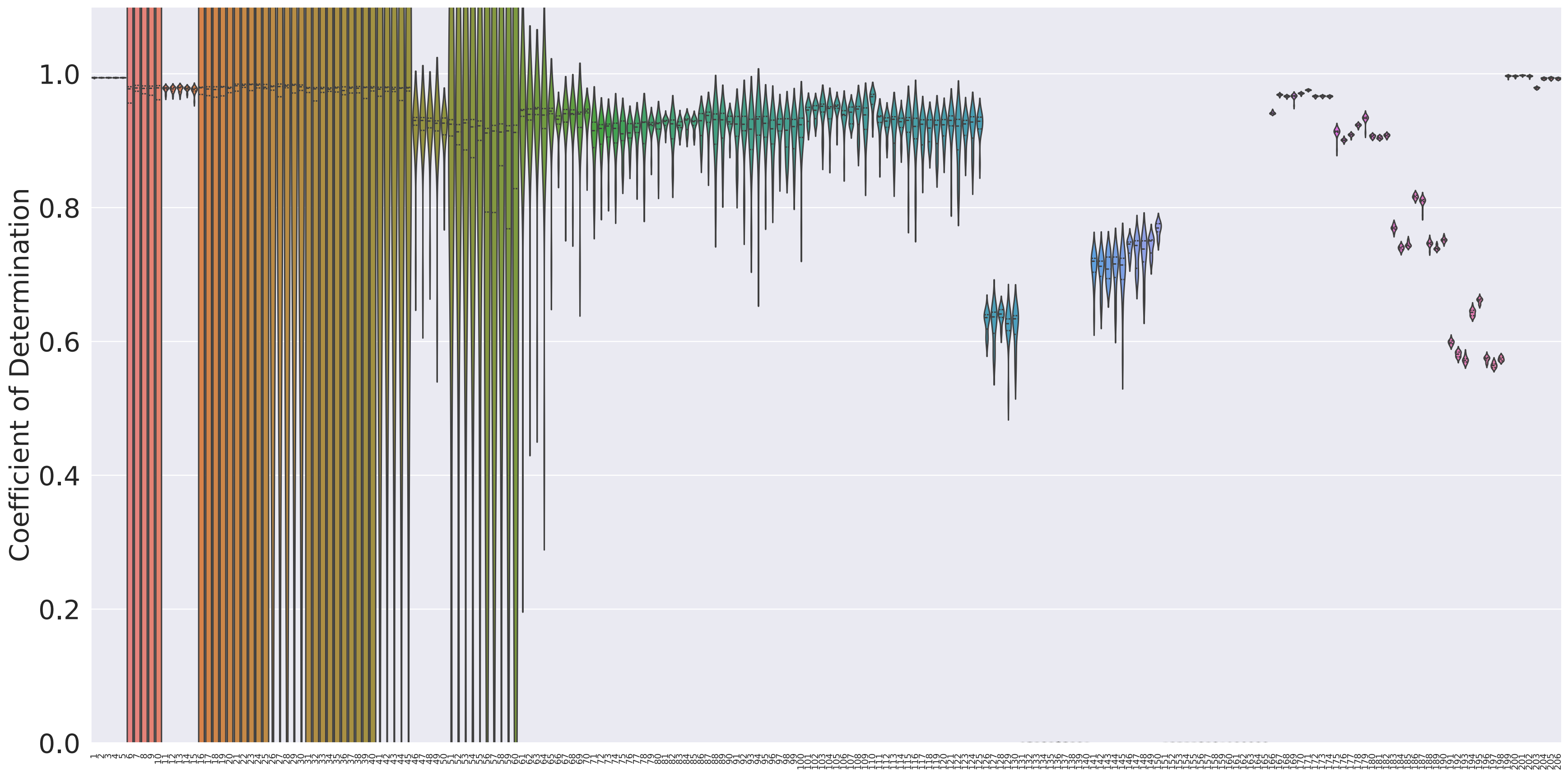

Figure S60. Violin plot of prediction accuracy (R^2) for 15 test sets of the PQC data set enthalpy prediction. The order of models is listed in Table S171.

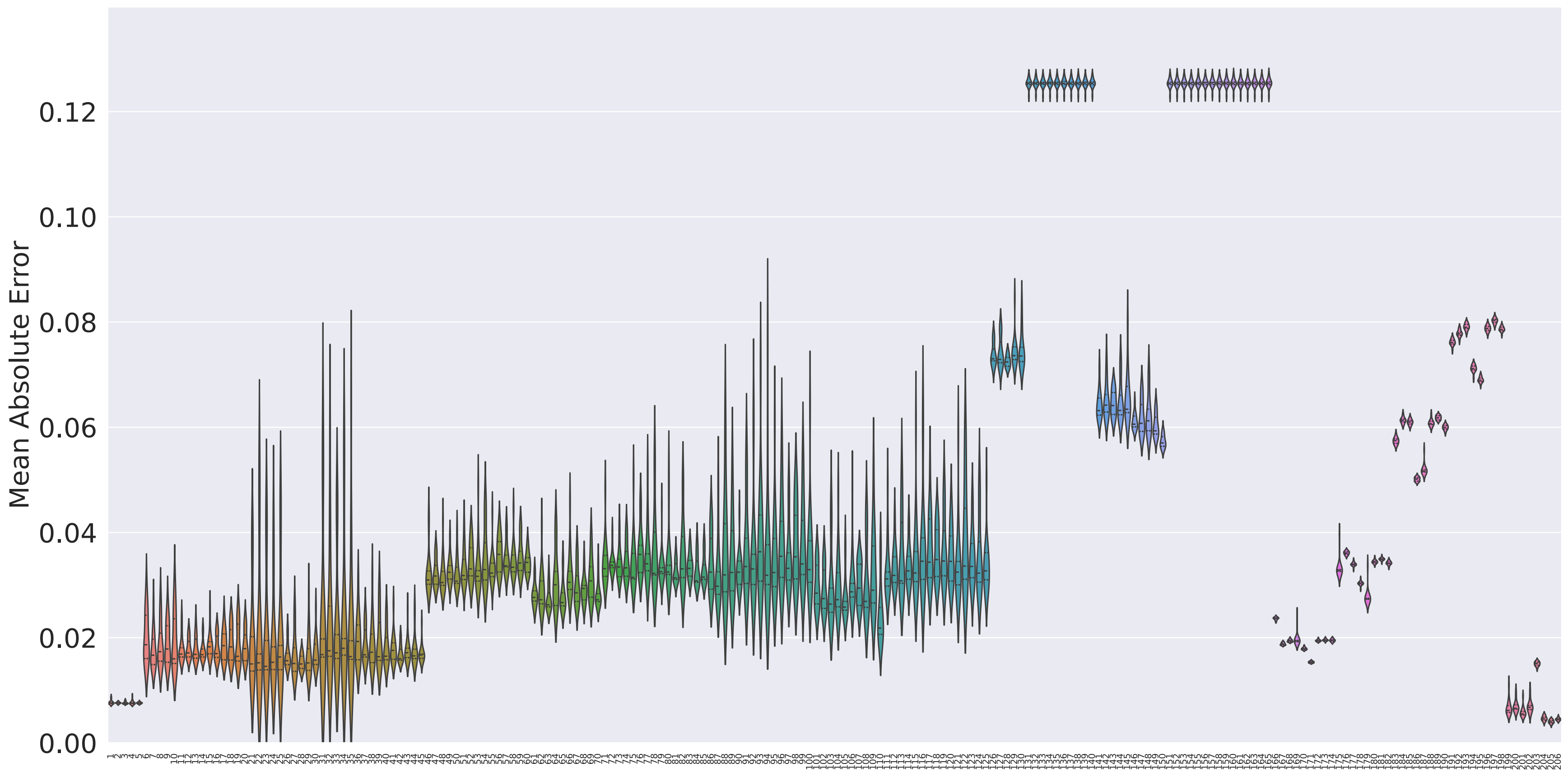

Figure S61. Violin plot of prediction accuracy (MAE) for 15 test sets of the PQC data set enthalpy prediction. The order of models is listed in Table S171.

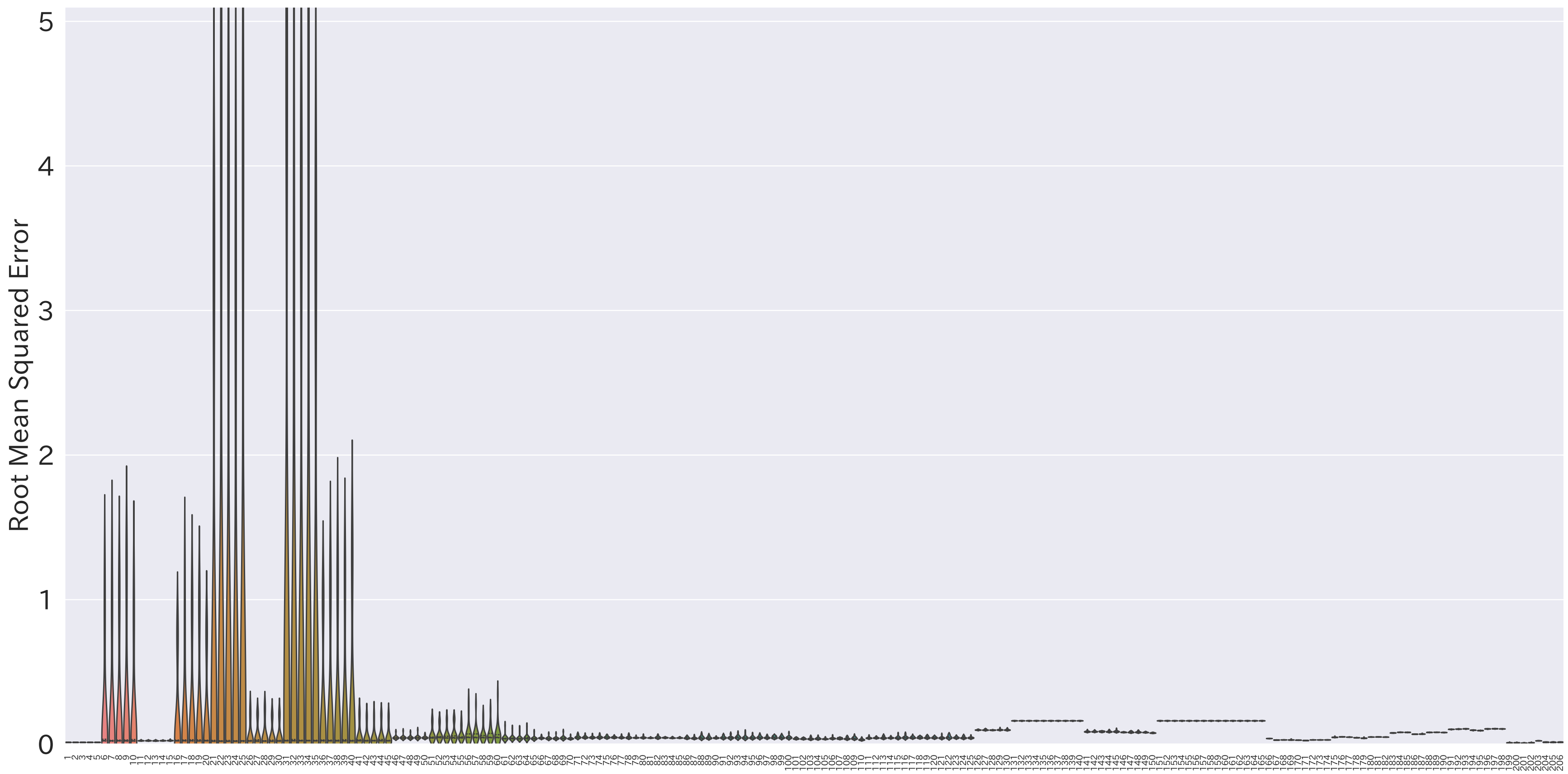

Figure S62. Violin plot of prediction accuracy (RMSE) for 15 test sets of the PQC data set enthalpy prediction. The order of models is listed in Table S171.

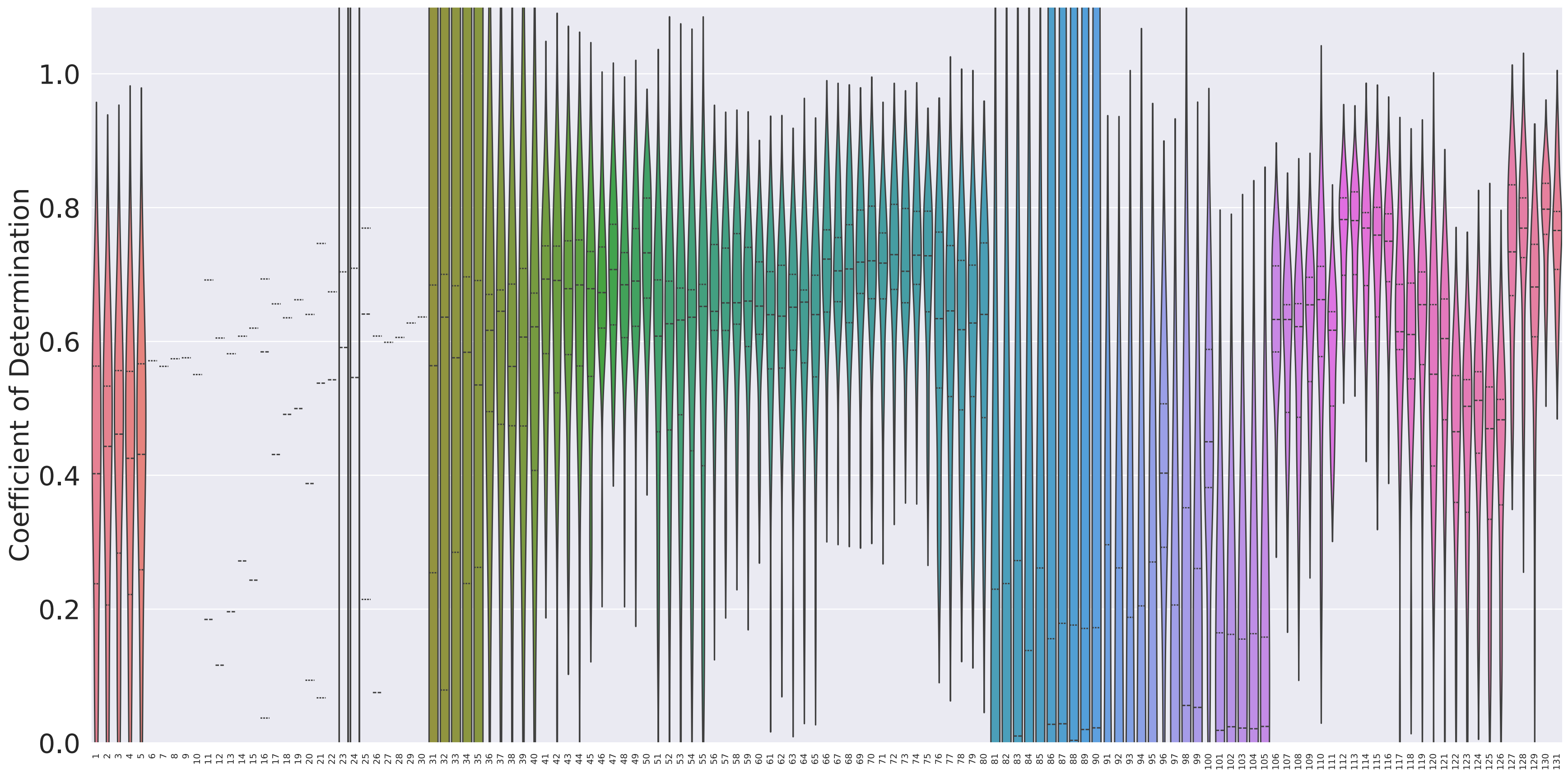

Figure S63. Violin plot of prediction accuracy (R^2) for 25 test sets of the MP data set property (melting point) prediction. The order of models is listed in Table S171.

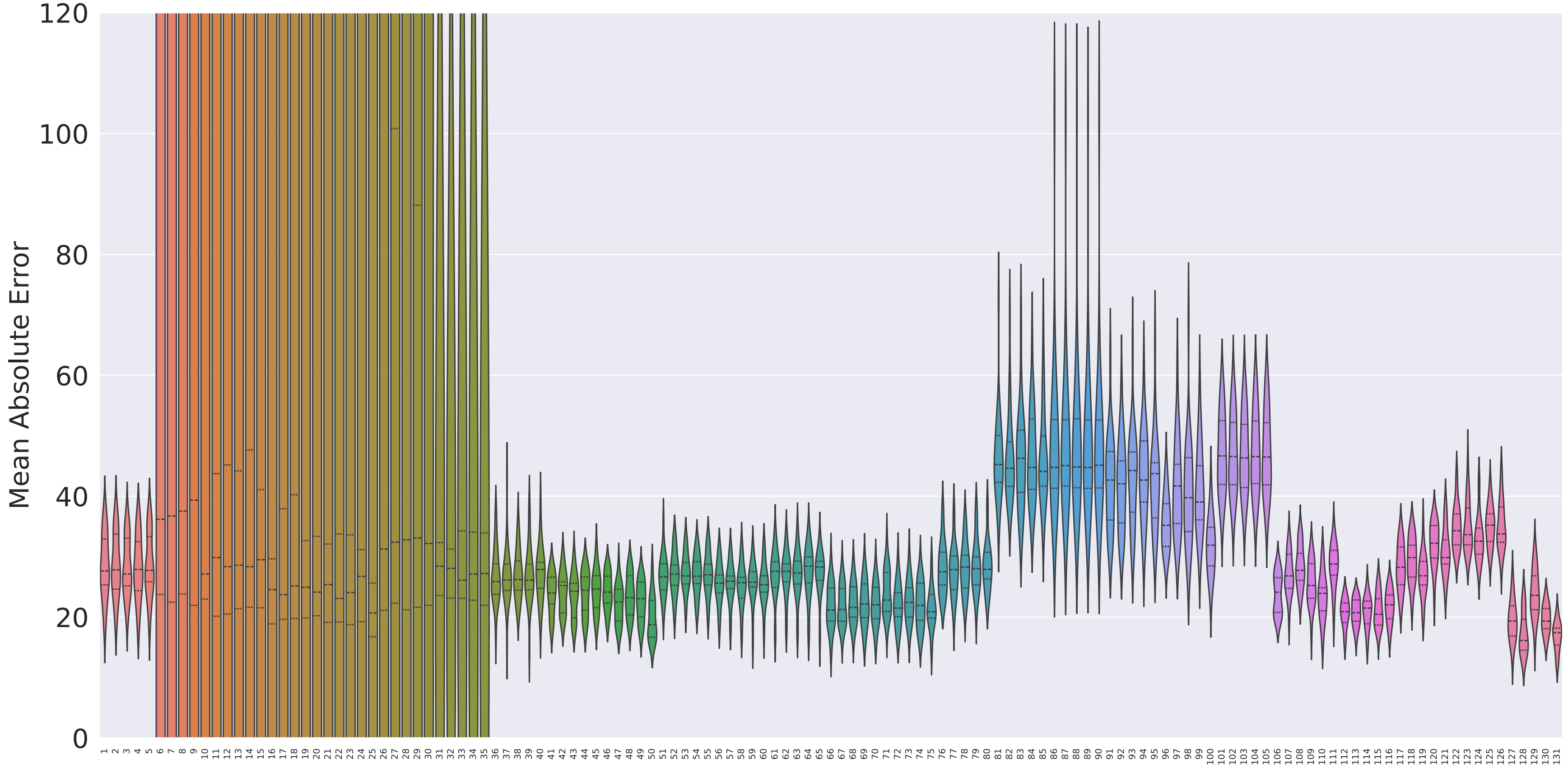

Figure S64. Violin plot of prediction accuracy (MAE) for 25 test sets of the MP data set property (melting point) prediction. The order of models is listed in Table S171.

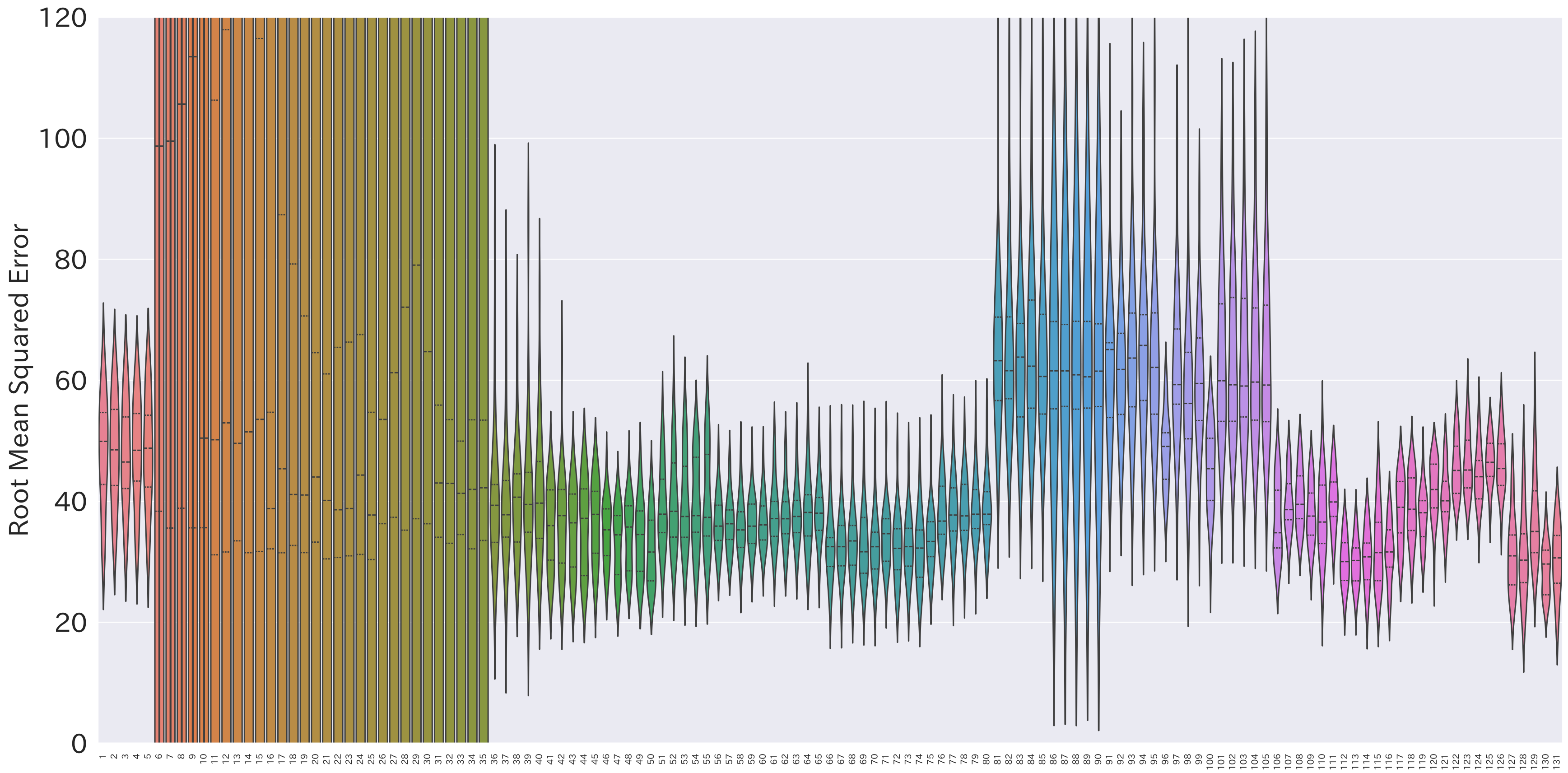

Figure S65. Violin plot of prediction accuracy (RMSE) for 25 test sets of the MP data set property (melting point) prediction. The order of models is listed in Table S171.

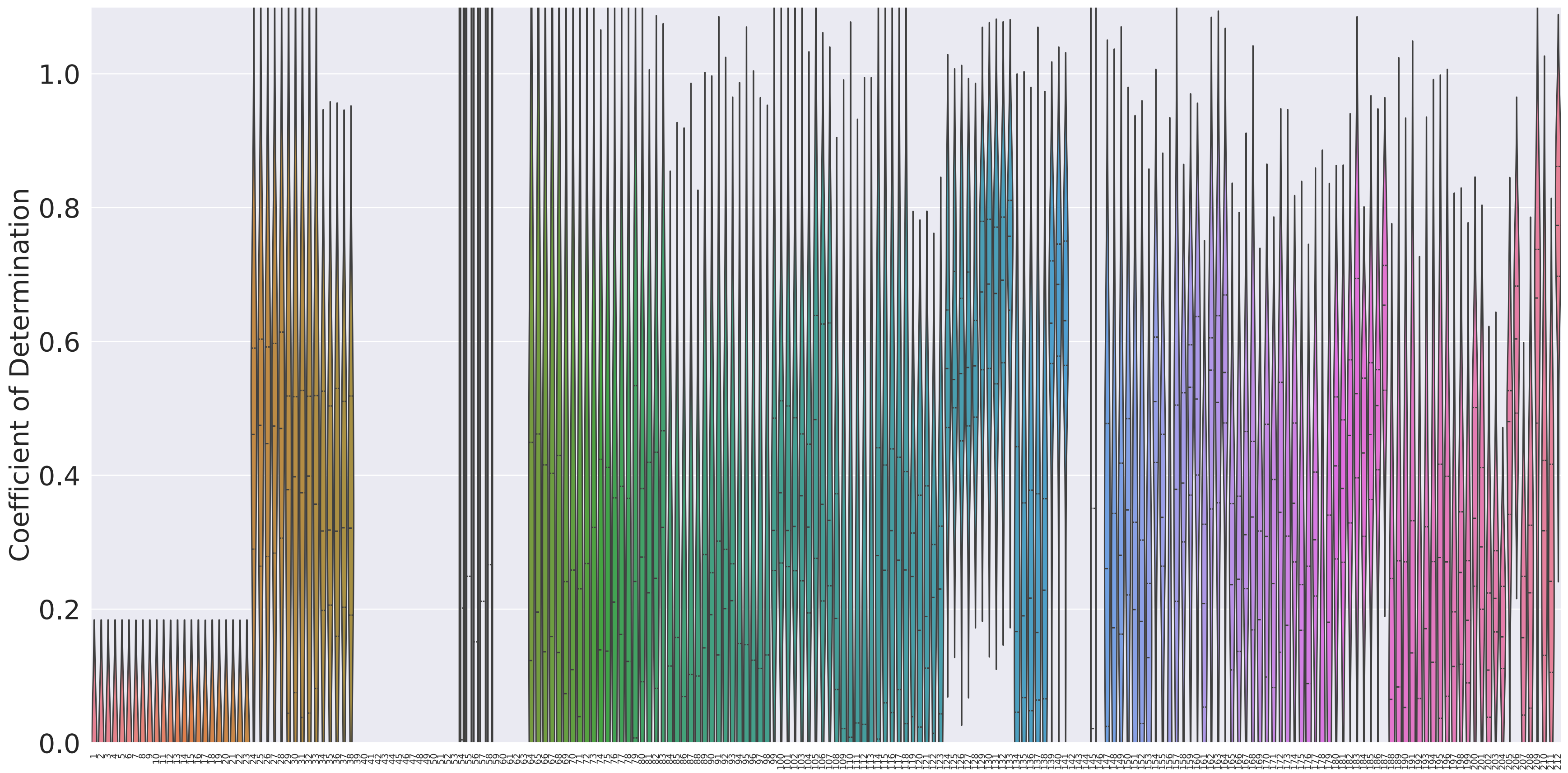

Figure S66. Violin plot of prediction accuracy (R^2) for 25 test sets of the APTC-1 data set property ($\Delta\Delta G^{\ddagger}$) prediction. The order of models is listed in Table S171.

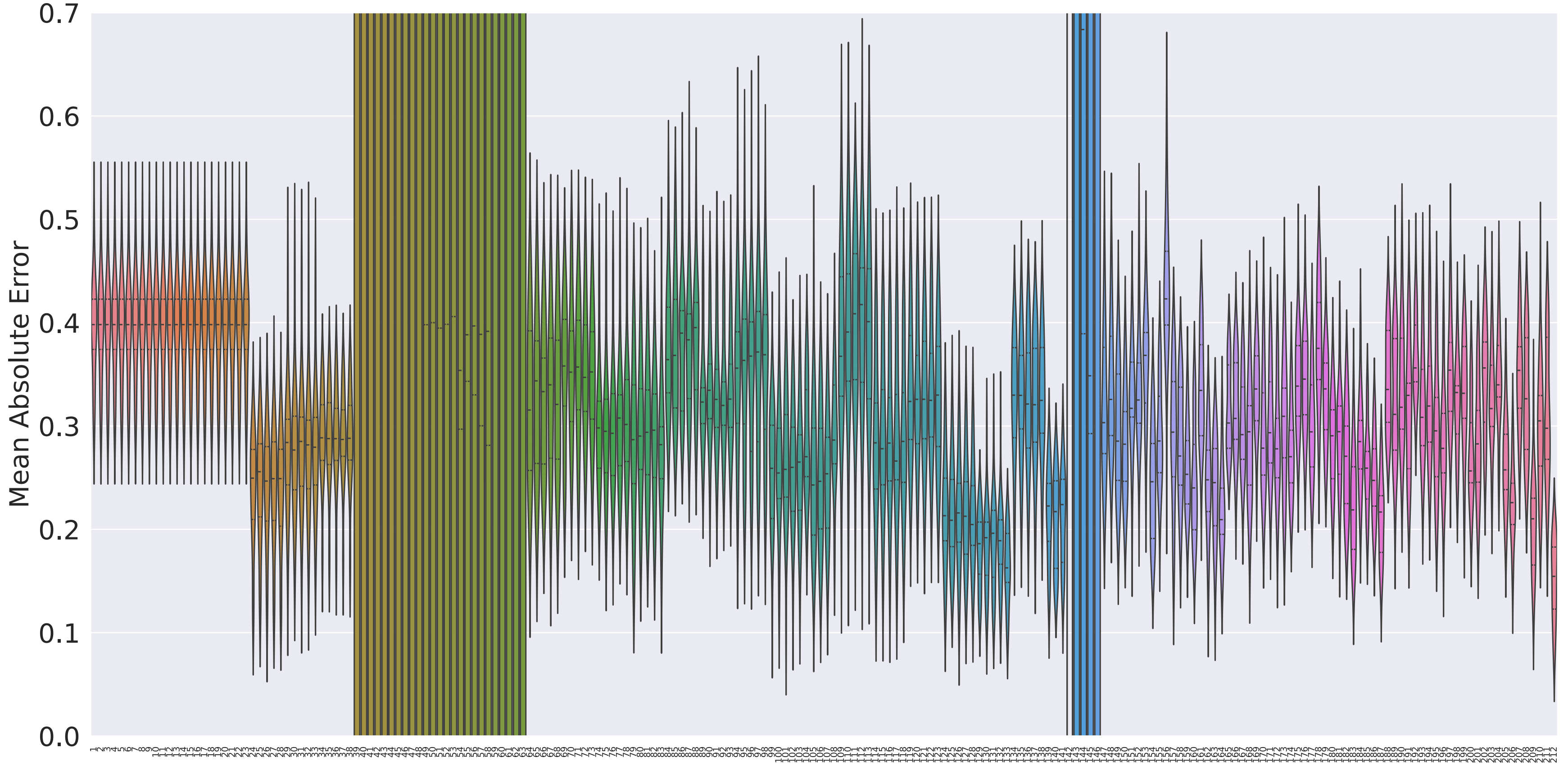

Figure S67. Violin plot of prediction accuracy (MAE) for 25 test sets of the APTC-1 data set property ($\Delta\Delta G^{\ddagger}$) prediction. The order of models is listed in Table S171.

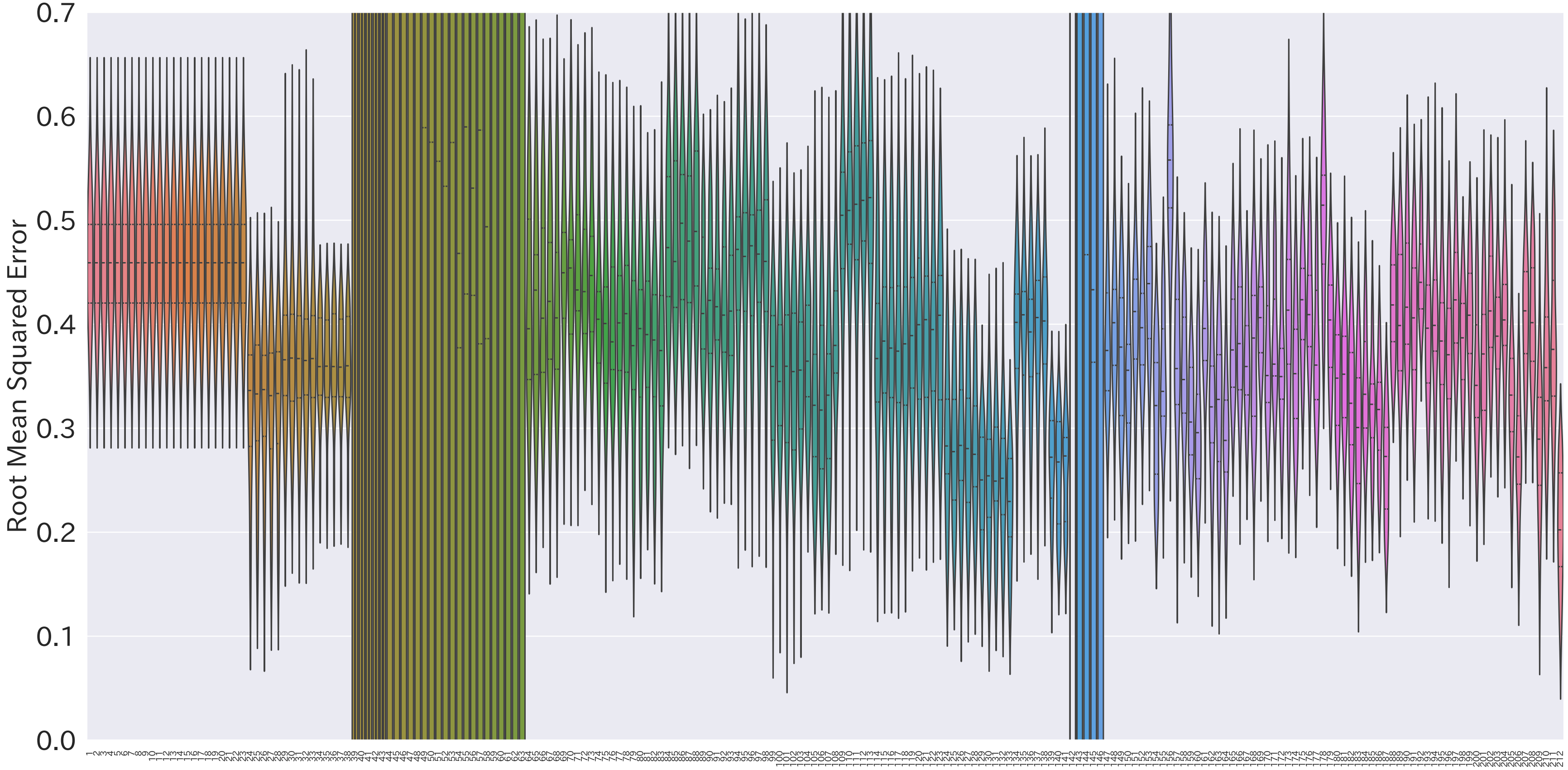

Figure S68. Violin plot of prediction accuracy (RMSE) for 25 test sets of the APTC-1 data set property ($\Delta\Delta G^{\ddagger}$) prediction. The order of models is listed in Table S171.

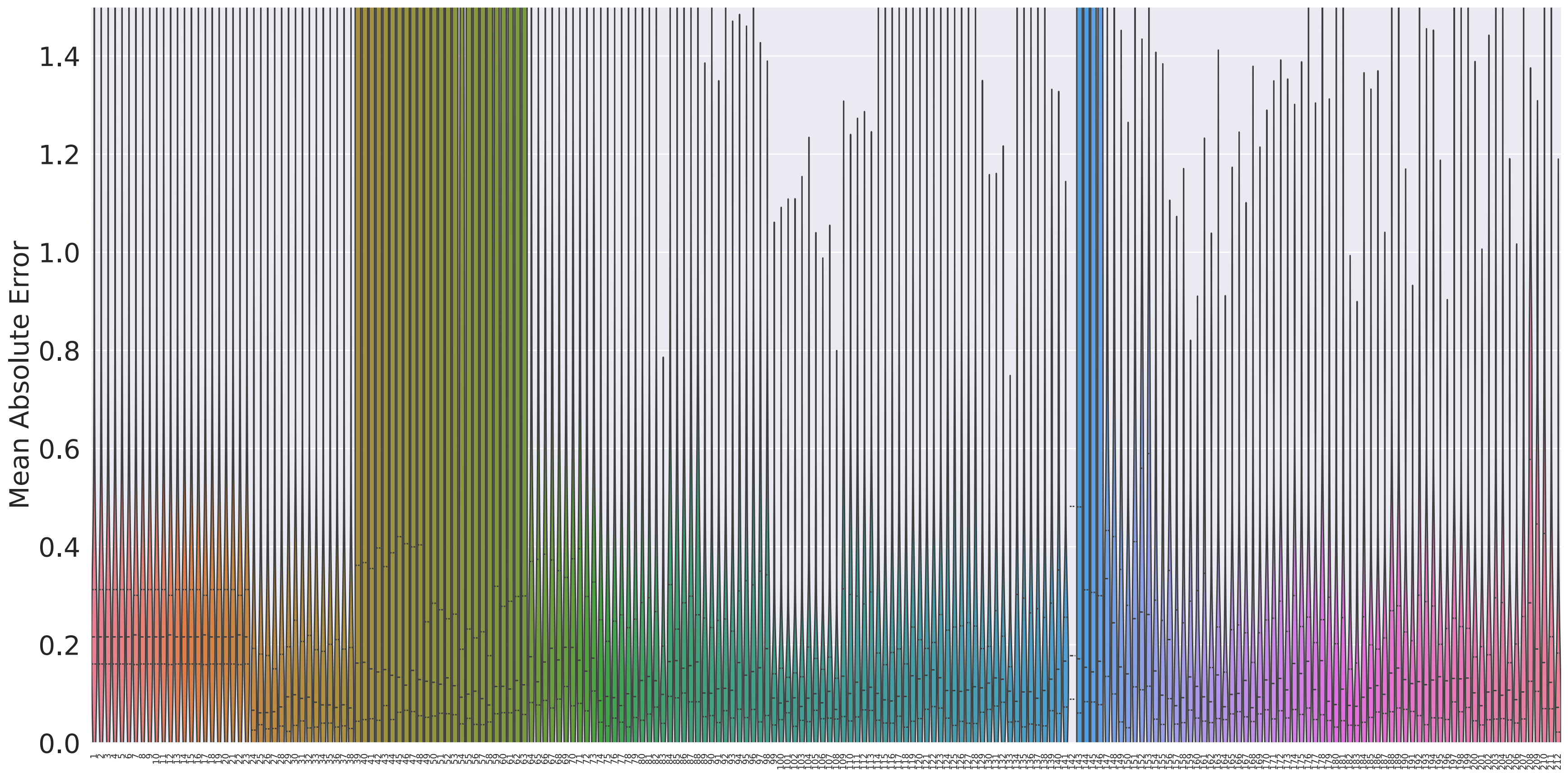

Figure S69. Violin plot of prediction accuracy (MAE) for 40 test points of the APTC-2 data set property ($\Delta\Delta G^{\ddagger}$) prediction. The order of models is listed in Table S171.

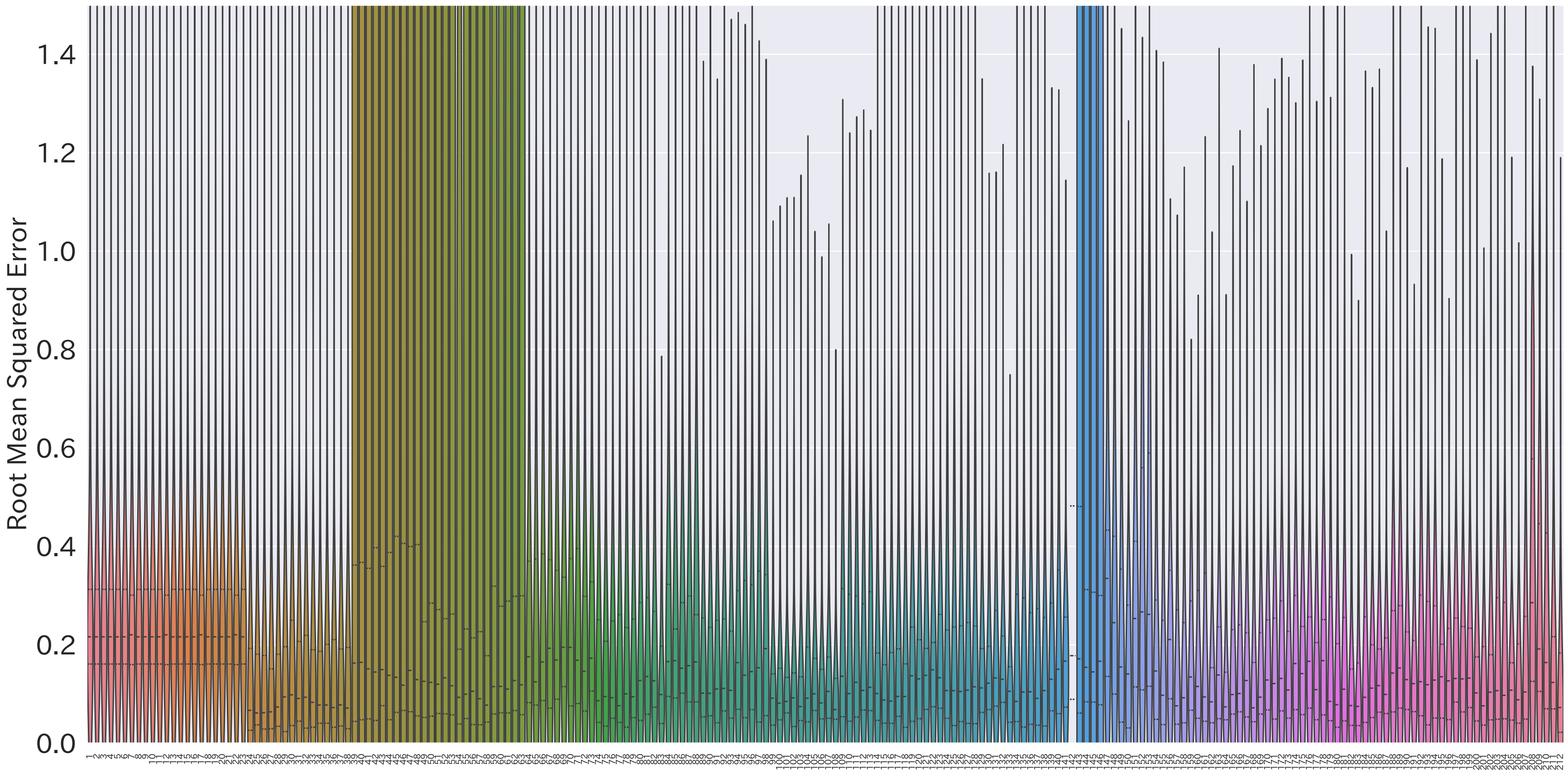

Figure S70. Violin plot of prediction accuracy (RMSE) for 40 test points of the APTC-2 data set property ($\Delta\Delta G^{\ddagger}$) prediction. The order of models is listed in Table S171.
